# Kinetic Resolution of β-Alkyl Phenylethylamine Derivatives through Palladium-Catalyzed, Nosylamide-Directed C−H Olefination

**DOI:** 10.3390/molecules28041852

**Published:** 2023-02-15

**Authors:** Zeng Zhao, Jinxin Wang, Zhiteng Du, Yuzhu Li, Qingyan Sun, Huizi Jin

**Affiliations:** 1 School of Pharmacy, Shanghai jiaotong University, Shanghai 200240, China; 2 School of Pharmacy, Second Military Medical University, Shanghai 200433, China; 3 Department of Pharmacy, Wenzhou Medical University, Wenzhou 325035, China; 4 State Key Laboratory of New Drug and Pharmaceutical Process, Shanghai Institute of Pharmaceutical Industry, China State Institute of Pharmaceutical Industry, Shanghai 201203, China

**Keywords:** C-H activation/alkenylation, *β*-alkyl phenylethylamine, styrene, directing group, enantioselective

## Abstract

Palladium-catalyzed C-H activation reactions have attracted the attention of organic researchers due to their unique high selectivity, broad functional group tolerance, and high efficiency, and they are widely used in natural products and asymmetric synthesis. Here, we report an example of enantioselective C-H alkenylation between *β*-alkyl phenylethylamine compounds and styrenes with Boc-L-lle-OH as the ligand and nosylamide as the directing group. This reaction is applicable to styrene containing various electron-deficient and electron-donating substitutions and may be utilized for the synthesis of benzoazepine compounds.

## 1. Introduction

A transition metal-catalyzed C-H bond activation reaction can directly functionalize the C-H bond, which greatly simplifies the steps of traditional organic synthesis. Therefore, it is widely accepted in drug synthesis, natural product synthesis, and chemical reagent synthesis [1,2,3,4]. Because of the inertness of the C-H bond and the massive existence of such bonds in organic molecules, selectivity and catalytic activity have become the key problems in C-H bond activation reactions. The use of the directing group strategy effectively solves these problems. More specifically, the C-H bonds at certain positions on the substrate can be selectively activated through the coordination of the directing group with the central metal, thus controlling the regio- and stereoselectivity of the reaction [5,6,7,8,9,10,11]. The *Yu* group has conducted extensive and in-depth research on this issue and developed a series of C-H bond activation methods. For example, in 2011, they successfully used carboxyl-directed C-H bond-activated alkenylation reactions to bond two complex fragments and then synthesized (±)-lithospermic acid succinctly and efficiently [12,13,14,15].

In recent years, enantioselective C-H iodination and alkylation have been achieved by combining Palladium (Pd, II) with a mono-N-protected amino acid (MPAA) ligand after kinetic resolutions [11,13,16,17,18,19]. Our research group investigated β-alkyl phenylethylamine compounds with fused bicyclic skeletons, which had been used in the synthesis of the natural product delavastine after Pd-catalyzed, trifluoromethanesulfonate-directed C-H alkylation and kinetic splitting. The structure of alkenylated products is similar to that of cinnamate esters and can be used for the further synthesis of dopamine receptor D1 ligands, α2-adrenergic receptors, 5-HT1A receptor ligands, poly (ADP-ribose) polymerase (PARP) inhibitors, and other drug molecules [20,21,22].

Styrene is an important raw material that is commonly used in chemical industrial processes; however, Pd-catalyzed C−H functionalizations of styrene are challenging due to the low reactivity of the substrates. In the previous study conducted by our research group, we used palladium acetate (Pd(OAc)_2_) as a catalyst to screen ligands, oxidants, and additives. We eventually achieved enantioselective C-H alkenylation between the *β*-alkyl phenyl ethylamine compound and styrene, with the use of Boc-L-lle-OH as a ligand based on higher yield and selectivity. The alkenylated products can be used for the synthesis of benzoazepine compounds, which can bond with dopamine D1 receptors. We also found that the selectivity of the reaction was influenced by the trace amount of water in the solvent, and the selectivity and yield were higher when two equivalents (equiv.) of water were added to the anhydrous solvent.

## 2. Results and Discussion

### 2.1. Preliminary Research and Reaction Optimizations

We started our research by exploring the enantioselective C-H kinetic resolution/alkenylation of racemic Nosyl-protected 2, 3-dihydro-1H-indene-1-methylamine (**rac-1a**). Under the guidance of the C-H alkenylation reaction conditions protected by trifluoromethanesulfonic acid, which were developed by our research group, the alkenylation reaction between **rac-1a** and the coupling agent styrene was carried out under the conditions of Pd(OAc)_2_, Boc-L-t-Leu-OH, AgOAc, K_2_CO_3_, and anhydrous tertiary amyl alcohol (t-AmOH)/1-methyl-2-pyrrolidinone (NMP) (3:1); the test conditions are summarized in Table 1. Under the initial conditions, we obtained the target C-H alkenylated product **2a**, in 30% yield and with an enantiomeric excess (ee) value of 88%, along with the recovered **1a**, in 57% yield and with an ee value of 28% (entry 1; also see the Supplementary Materials). Then, we explored the influence of solvents containing different proportions of water (entries 2 and 3). With two equiv. of water in the anhydrous solvent, the ee value of product **2a** was up to 92%, which was higher than when no water or 4 equiv. of water were added. When the temperature rose to 80 °C, the reaction rate was high, and the yield of product **2a** increased to 41%, but the ee value was only 78% (entry 4). On the contrary, the yield did not change after the oxidant benzoquinone (BQ) was added, but the ee value of product **2a** rose to 93% (entry 5). Encouraged by this result, we screened other mono-protected amino acid ligands (entries 6 and 7). It was found that Boc-L-lle-OH was the most effective chiral ligand, and the ee values of products **2a** and **1a** were 94% and 64%, respectively. A smaller acetyl-protecting group reduced the yield and enantiomeric purity of product **2a** (entry 7). With this preliminary result, we began to test whether an inorganic base could enhance the C-H activation/alkenylation reaction. Unfortunately, we found that other types of inorganic bases (Na_2_CO_3_, entry 8, and Na_3_PO_4_, entry 9) could reduce enantiomeric selectivity and yield.

### 2.2. Substrate Range of Nosylamide-Directed C-H Alkenylation

**Scheme 1:**
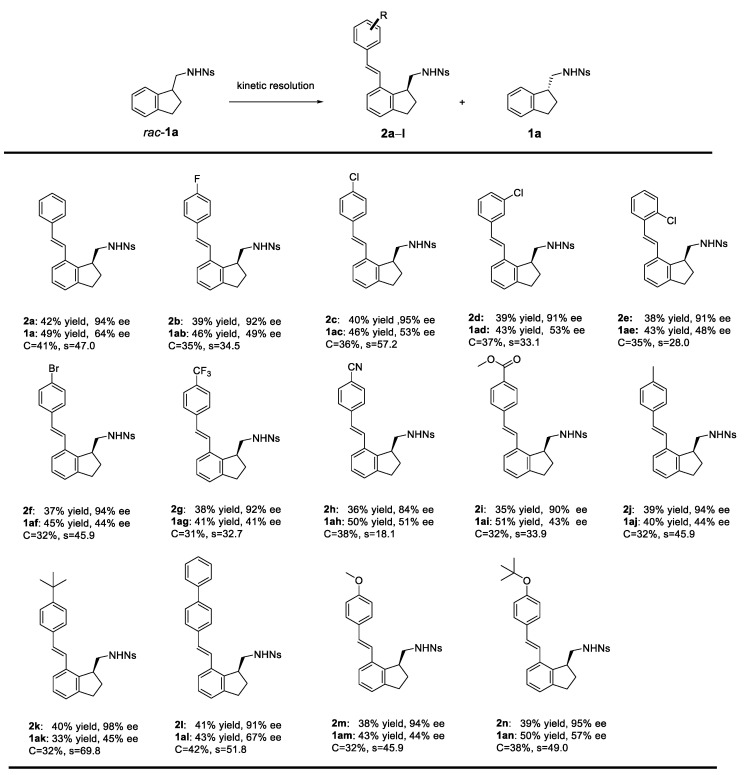
Enantioselective C-H olefination/kinetic resolution of the β-alkyl phenylethylamine derivative. Reaction conditions: anhydrous t-AmOH/NMP (3:1, 0.05 M), rac-1a (0.1 mmol), styrene or substituted styrenes (3.0 equiv.), Pd(OAc)_2_ (20 mol%), Boc-L-lle-OH (40 mol%), AgOAc (2.5 equiv.), K_2_CO_3_ (2.5 equiv.), BQ (0.5 equiv.), H_2_O (2.0 equiv.); 24 h. The enantiomeric excesses were determined by HPLC with a chiral stationary phase. The selectivity factor (s) = (rate of fast-reacting enantiomer)/(rate of slow-reacting enantiomer) = ln[(1-C)(1-ee)]/ln[(1-C)(1+ee)] where C is the conversion [C=ee_SM_/(ee_SM_+ee_PR_)] and ee is the enantiomeric excess of the re-maining starting material [23].

With the optimized reaction conditions, we studied the applicability of a styrene coupling agent. To our satisfaction, the enantioselective C-H alkenylation reaction of racemic ***rac-*1** with various substituted styrenes proceeded smoothly, producing the enantiomer-enriched compounds **2a-n** and **1a**. As shown in Scheme 1, the reaction with p-chlorostyrene generated the alkenylated product **2c** with an ee of 95% and 40% yield, while **1ac** was recovered in 46% yield, which corresponds to a selectivity factor (s) of 57.2 [23]. We also found that the substitution position of chlorine does not greatly affect the kinetic resolution. The fluorine and trifluoromethyl substituted styrenes are also suitable coupling agents, providing the corresponding products **2b** and **2g** with an ee value of 92%. Aryl bromides (**2f**), nitriles (**2h**), and esters (**2i**) with electron-accepting functional groups have good tolerance in this reaction. Coupling agent ligands containing the electron-donating methyl (**2j**), tert-butyl (**2k**), and methoxy groups (**2m**) also performed well and provided alkenylated products with excellent selectivity, especially when the ee value of **2k** reached 98%. The larger substitutional groups on styrene, such as the benzene ring (**2l**) and the tert-butyl group (**2n**), have a slight impact on the selectivity. To sum up, this method is applicable to styrene containing various electron-deficient and electron-donating substitutions. 

**Scheme 2:**
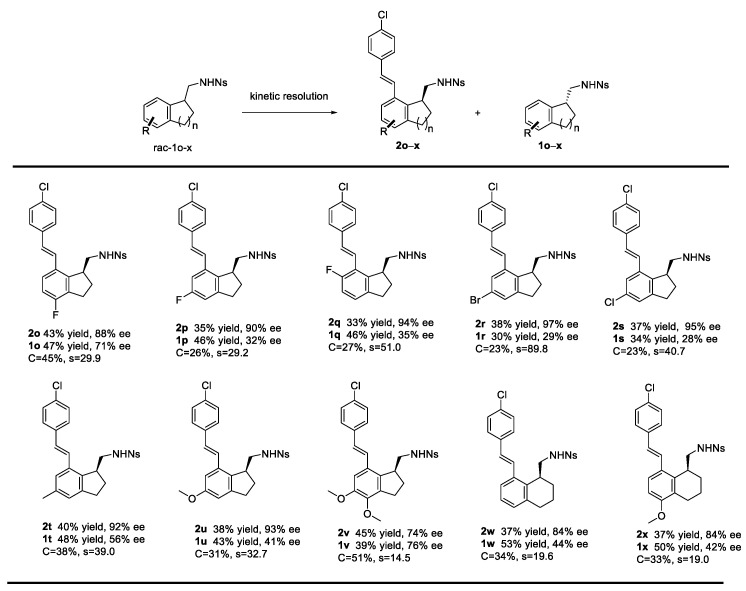
Scope of the *β*-alkyl phenylethylamine substrate.

With these results, we further studied the applicability of amines based on kinetic resolution. We chose chlorostyrene, which has a higher yield and selectivity and is suitable for further modification, as the coupling agent and obtained the enantiomer-enriched compounds **1o-x** and **2o-x** (see Scheme 2 and the Supplementary Materials). We were pleased to find that our method could tolerate various substitutional groups on *β*-alkyl phenethylamine; regardless of the nature of the electrons on the aromatic substrate, the reaction proceeded smoothly. The enantiomeric excess of the fluorine-containing aromatic substrate was 88%, 90%, and 94% (**2o**, **2p** and **2q**, respectively), which indicates that the substitution position of fluorine had some effect on the selectivity. Under the conditions of C-H alkenylation, no direct Heck reaction of aryl bromides with chlorostyrene was observed, although the yield decreased slightly, and the ee value reached 97% (**2r**). Substitutional groups containing chlorine (**2s**), methyl (**2t**), and methoxy (**2u**) were also kinetically resolved to obtain enantiomer-enriched compounds with higher yields, but the two methoxy-substituted substrate (**2v**) had a slightly worse ee value (74%). The kinetic resolution of the tetralin substrates also had good yield and stereoselectivity (**2w** and **2x**). All results indicate that this method provides an opportunity for the orthogonal functionalization of the optically active substituted arenes.

**Scheme 3:**
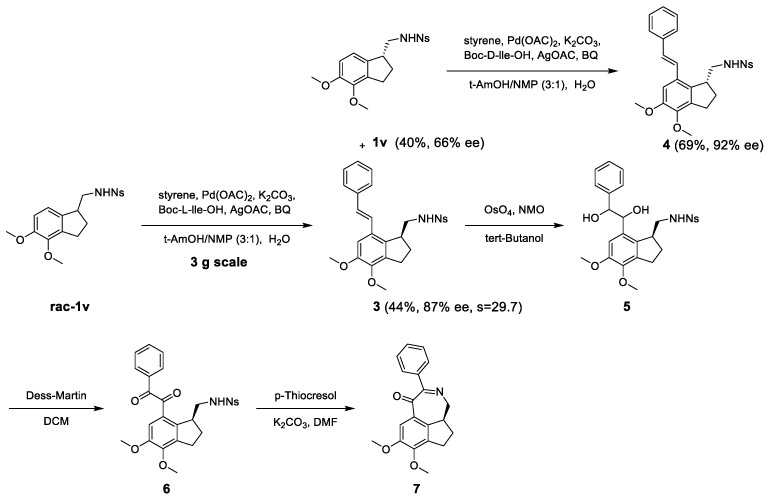
Enantioselective C-H kinetic resolution of the recovered starting material and synthesis of the nitrogen-containing heptatomic ring.

In order to prove the synthesis function of the enantioselective C-H alkenylation reaction, we chose Boc-L-lle-OH as the ligand and performed a gram-scale experiment using ***rac*-1v** and styrene under optimal reaction conditions. As shown in Scheme 3, the solution proceeded smoothly on a 3 g scale, and the alkenylated product **3** was obtained with a yield of 44% and an ee value of 87%. Meanwhile, the raw material **1v** was recovered. Then, we performed the kinetic resolution of the recovered **1v** together with the ligand Boc-D-lle-OH opposite to the enantiomer, and compound **4** was obtained with a yield of 69% and an ee value of 92%. In addition, we oxidized the carbon double bond of compound **3** in the double carbonyl group using OsO_4_ and Dess–Martin periodinane, and then we removed the Nosyl-protecting group to construct a nitrogen-containing heptatomic ring (compound **7**, see Scheme 3). It is worth noting that the indenonitrogen-containing heptatomic ring is an important structural unit in natural products and bioactive molecules.

Reaction conditions: anhydrous t-AmOH/NMP (3:1, 0.05 M), rac-1o-x (0.1 mmol), 4-Chlorostyrene (3.0 equiv), Pd(OAc)_2_ (20 mol%), Boc-L-lle-OH (40 mol%), AgOAc (2.5 equiv.), K_2_CO_3_ (2.5 equiv.), BQ (0.5 equiv.), H_2_O (2.0 equiv.); 24 h. The enantiomeric excesses were determined by HPLC with a chiral stationary phase. The selectivity factor (s) = (rate of fast-reacting enantiomer)/(rate of slow-reacting enantiomer) = ln[(1-C)(1-ee)]/ln[(1-C)(1+ee)] where C is the conversion [C=ee_SM_/(ee_SM_+ee_PR_)] and ee is the enantiomeric excess of the re-maining starting material [23].

**Scheme 4:**
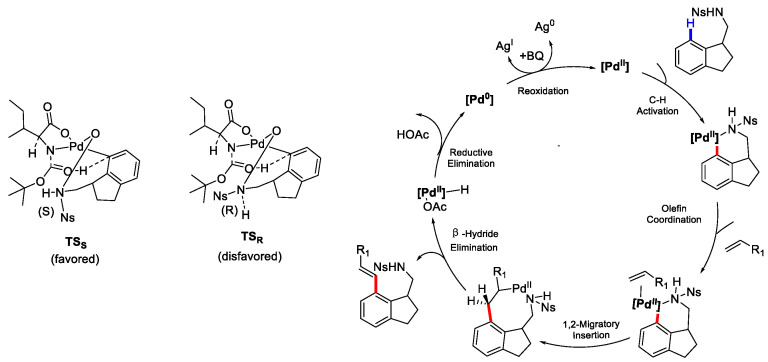
The proposed transition-state model and the catalytic cycle for C-H olefination.

Based on previous, extensive structural and literature research, we proposed two possible transition states, TS_S_ and TS_R_ (Scheme 4). In both TS_S_ and TS_R_, palladium is coordinated with Boc-L-lle-OH and the substrate in a planar manner. The isopentane moves upward, which then pushes the Boc groups below the palladium coordination plane to avoid steric repulsion. In the C-H activation step, the transition state TS_R_ is expected to be disfavored relative to TS_S_ because of the steric repulsion between Boc and Ns in TS_R_, which is consistent with the faster formation of the product with the S configuration [12,18,22,24]. Thus, the Pd(II)-catalyzed C-H olefination of β-alkyl phenylethylamine compounds such as **rac-1** has been proposed to proceed as follows (Scheme 4). After the coordination of **rac-1** with Pd(II), nosylamide-directed ortho-C−H cleavage takes place to form a cyclopalladated intermediate with an olefin. This intermediate undergoes 1,2-migratory insertion, followed by *β*-hydride elimination in order to generate the product. Pd(0), which is presumably stabilized by Boc-L-lle-OH, is then reoxidized to Pd(II) by Ag(I) and BQ, at which point it re-enters the catalytic cycle [17,25].

In conclusion, we realized the enantioselective C-H kinetic resolution of *β*-alkyl phenylethylamine compounds using the Boc-L-lle-OH ligand under the catalysis of Pd(OAc)_2_. It was found that the presence of trace water in solvents could improve the selectivity of reactions. Moreover, the alkenylated products could be used to easily construct a heptatomic ring with a definite spatial configuration, which will provide a new method for the synthesis of benzoazepine compounds.

## 3. Materials and Methods

### 3.1. Chemistry

#### 3.1.1. General Information

All solvents were obtained from Damas-beta, Bidepharm, Alfa-Aesar, and TCI and used directly without further purification. Palladium catalysts and MPAA ligands were purchased from Sigma-Aldrich and Damas-beta; styrene and substituted styrene were obtained from Bidepharm, Damas-beta and TCI. The starting materials and 4-Nitrobenzenesulfonyl chloride used to prepare the Nosyl-protected *β*-alkyl phenylethylamine compounds were purchased from Damas-beta, TCI and Bidepharm. ^1^H NMR and ^13^C NMR spectra were recorded on Bruker-Ascend (400 MHz for ^1^H; 101 MHz for ^13^C, respectively) and Bruker-DRX (500 MHz for ^1^H and 126 MHz for ^13^C) instruments that had been internally referenced to tetramethylsilane or chloroform signals (^1^H: δ7.26, ^13^C: δ77.16). The NMR samples were kept under vacuum before measurement to remove possible solvate molecules. HPLC data were recorded at the Center for Mass Spectrometry, Shanghai Institute of Organic Chemistry, Chinese Academy of Sciences. All reactions were monitored by thin-layer chromatography (TLC) using silica-gel plates (silica gel 60 F254 0.25 mm).

#### 3.1.2. Synthesis

**Scheme 5:**
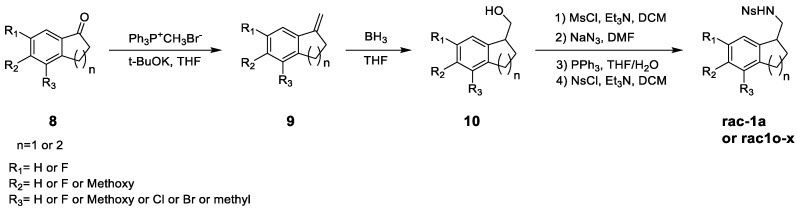
The synthetic routes of **rac-1a** and various substitutional groups on β-alkyl phenethylamine (**rac-1o-x**).

**Scheme 6:**
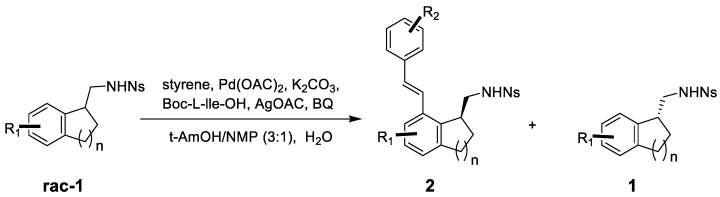
Enantioselective C-H olefination/kinetic resolution of Nosyl-protected β-alkyl phenylethylamine derivatives.

Depiction of the synthesis of the compounds **rac-1a** and **rac-1o-x**. The compounds **rac-1a** or **rac-1o-x** were obtained from 1-indanone or 1-tetralone via the Witting reaction, borane reduction, azide reaction followed by the Staudinger reaction, and amino protection in 20–40% yields (Scheme 5 and Scheme 6) [26,27,28,29].

**rac-1a**: ^1^H NMR (500 MHz, CDCl_3_) δ 8.14–8.10 (m, 1H), 7.88–7.83 (m, 1H), 7.75–7.71 (m, 2H), 7.23–7.11 (m, 4H), 5.32 (t, *J* = 5.9 Hz, 1H), 3.46–3.36 (m, 2H), 3.20 (ddd, *J* = 12.0, 7.1, 5.4 Hz, 1H), 2.99–2.82 (m, 2H), 2.32–2.24 (m, 1H), 1.88 (ddt, *J* = 12.8, 8.7, 6.3 Hz, 1H). ^13^C NMR (126 MHz, CDCl_3_) δ 148.2, 144.5, 142.9, 133.7, 132.9, 131.3, 127.6, 126.6, 125.6, 125.1, 123.8, 47.8, 44.8, 31.2, 29.4. HR-ESI-MS: calcd for C_16_H_16_N_2_O_4_SNa [M + Na]^+^ 355.0723; found 355.0721.

**rac-1o**: ^1^H NMR (400 MHz, CDCl_3_) δ 8.12 (dq, *J* = 7.6, 4.1 Hz, 1H), 7.86 (dt, *J* = 7.4, 3.8 Hz, 1H), 7.74 (dq, *J* = 7.3, 3.9 Hz, 2H), 7.17–7.09 (m, 1H), 6.95 (d, *J* = 7.4 Hz, 1H), 6.87 (t, *J* = 8.6 Hz, 1H), 5.34 (d, *J* = 6.2 Hz, 1H), 3.41 (tt, *J* = 12.4, 6.2 Hz, 2H), 3.28–3.16 (m, 1H), 2.92 (ddq, *J* = 31.4, 16.2, 8.5, 6.8 Hz, 2H), 2.32 (td, *J* = 13.7, 8.2 Hz, 1H), 1.93 (ddd, *J* = 19.3, 8.7, 6.0 Hz, 1H). ^13^C NMR (101 MHz, CDCl_3_) δ 158.8, 146.6, 133.8, 133.0, 131.2, 128.7, 128.7, 125.6, 119.5, 119.5, 114.4, 114.1, 47.5, 45.2, 29.4, 27.1. HR-ESI-MS: calcd for C_16_H_15_FN_2_O_4_SNa [M + Na]^+^ 373.0629; found 373.0632.

**rac-1p**: ^1^H NMR (400 MHz, CDCl_3_) δ 8.11 (dd, *J* = 5.8, 3.4 Hz, 1H), 7.86 (dd, *J* = 5.8, 3.5 Hz, 1H), 7.74 (dq, *J* = 7.4, 4.0 Hz, 2H), 7.08 (dd, *J* = 8.2, 5.2 Hz, 1H), 6.89 (d, *J* = 8.7 Hz, 1H), 6.81 (t, *J* = 8.7 Hz, 1H), 5.31 (d, *J* = 6.4 Hz, 1H), 3.36 (tq, *J* = 12.5, 6.2, 5.8 Hz, 2H), 3.18 (dt, *J* = 11.3, 6.1 Hz, 1H), 2.89 (dtd, *J* = 31.4, 16.0, 7.0 Hz, 2H), 2.31 (td, *J* = 14.3, 8.3 Hz, 1H), 1.96–1.86 (m, 1H). ^13^C NMR (101 MHz, CDCl_3_) δ 164.0, 146.9, 138.4, 133.7, 133.0, 131.2, 125.6, 124.7, 113.7, 113.5, 112.2, 112.0, 47.8, 44.0, 31.3, 29.8. HR-ESI-MS: calcd for C_16_H_15_FN_2_O_4_SNa [M + Na]^+^ 373.0629; found 373.0634.

**rac-1q**: ^1^H NMR (400 MHz, CDCl_3_) δ 8.11 (dq, *J* = 7.6, 4.1 Hz, 1H), 7.85 (dt, *J* = 7.5, 3.7 Hz, 1H), 7.75 (dq, *J* = 7.5, 4.1 Hz, 2H), 7.13 (dd, *J* = 8.1, 5.3 Hz, 1H), 6.88–6.77 (m, 2H), 3.39 (ddt, *J* = 20.1, 12.5, 5.7 Hz, 2H), 3.19 (dt, *J* = 11.6, 6.3 Hz, 1H), 2.86 (dh, *J* = 31.1, 7.2 Hz, 2H), 2.38–2.26 (m, 1H), 1.93 (ddt, *J* = 12.9, 8.6, 6.3 Hz, 1H). ^13^C NMR (101 MHz, CDCl_3_) δ 163.3, 160.9, 148.1, 145.0, 139.8, 133.8, 133.0, 131.2, 126.0, 125.6, 114.6, 114.3, 111.0, 110.7, 47.5, 44.9, 30.5, 29.9. HR-ESI-MS: calcd for C_16_H_15_FN_2_O_4_SNa [M + Na]^+^ 373.0629; found 373.0631.

**rac-1r**: ^1^H NMR (400 MHz, CDCl_3_) δ 8.11–8.07 (m, 1H), 7.89–7.84 (m, 1H), 7.77–7.71 (m, 2H), 7.33 (s, 1H), 7.23 (d, *J* = 8.0 Hz, 1H), 7.03 (d, *J* = 8.0 Hz, 1H), 5.33 (d, *J* = 6.1 Hz, 1H), 3.42–3.30 (m, 2H), 3.20 (dt, *J* = 11.8, 6.4 Hz, 1H), 2.97–2.81 (m, 2H), 2.29 (dtd, *J* = 14.4, 8.3, 6.2 Hz, 1H), 1.89 (ddt, *J* = 13.0, 8.6, 6.1 Hz, 1H). ^13^C NMR (101 MHz, CDCl_3_) δ 146.9, 142.0, 133.8, 133.0, 131.2, 129.6, 128.3, 125.6, 125.3, 121.4, 47.5, 44.4, 31.1, 29.5. HR-ESI-MS: calcd for C_16_H_15_BrN_2_O_4_SNa [M + Na]^+^ 432.9828; found 432.9833.

**rac-1s**: ^1^H NMR (400 MHz, CDCl_3_) δ 8.12–8.07 (m, 1H), 7.89–7.84 (m, 1H), 7.77–7.71 (m, 2H), 7.18 (s, 1H), 7.08 (s, 2H), 5.33 (d, *J* = 6.4 Hz, 1H), 3.37 (tt, *J* = 12.4, 6.4 Hz, 2H), 3.19 (td, *J* = 10.6, 9.2, 5.2 Hz, 1H), 2.97–2.78 (m, 2H), 2.35–2.24 (m, 1H), 1.95–1.85 (m, 1H). ^13^C NMR (101 MHz, CDCl_3_) δ 146.5, 141.5, 133.7, 133.7, 133.3, 133.0, 131.2, 126.8, 125.6, 125.3, 124.9, 47.6, 44.3, 31.1, 29.6. HR-ESI-MS: calcd for C_16_H_15_ClN_2_O_4_SNa [M + Na]^+^ 389.0333; found 389.0330.

**rac-1t**: ^1^H NMR (400 MHz, CDCl_3_) δ 8.11 (dt, *J* = 7.6, 3.8 Hz, 1H), 7.85 (dt, *J* = 7.3, 3.8 Hz, 1H), 7.78–7.70 (m, 2H), 7.03 (d, *J* = 7.3 Hz, 2H), 6.94 (d, *J* = 7.8 Hz, 1H), 5.33 (s, 1H), 3.37 (ddq, *J* = 19.1, 12.7, 5.7 Hz, 2H), 3.18 (dt, *J* = 11.9, 6.7 Hz, 1H), 2.98–2.76 (m, 2H), 2.31 (s, 3H), 2.29–2.21 (m, 1H), 1.86 (ddt, *J* = 12.8, 8.5, 6.2 Hz, 1H). ^13^C NMR (101 MHz, CDCl_3_) δ 148.1, 144.7, 139.9, 137.3, 133.7, 132.9, 131.3, 127.4, 125.8, 125.5, 123.5, 47.8, 44.3, 31.1, 29.6, 21.4. HR-ESI-MS: calcd for C_17_H_18_N_2_O_4_SNa [M + Na]^+^ 369.0879; found 369.0885.

**rac-1u**: ^1^H NMR (400 MHz, CDCl_3_) δ 8.11 (dd, *J* = 5.8, 3.4 Hz, 1H), 7.85 (dt, *J* = 7.5, 3.8 Hz, 1H), 7.76–7.71 (m, 2H), 7.03 (d, *J* = 8.3 Hz, 1H), 6.75 (s, 1H), 6.67 (dd, *J* = 8.3, 2.3 Hz, 1H), 5.35–5.28 (m, 1H), 3.77 (s, 3H), 3.41–3.30 (m, 2H), 3.20–3.13 (m, 1H), 2.86 (ddq, *J* = 31.2, 15.9, 8.3, 7.0 Hz, 2H), 2.33–2.22 (m, 1H), 1.92–1.83 (m, 1H). ^13^C NMR (101 MHz, CDCl_3_) δ 159.6, 146.2, 134.9, 133.7, 132.9, 131.3, 125.6, 124.3, 112.6, 110.4, 55.5, 48.0, 43.9, 31.4, 29.8. HR-ESI-MS: calcd for C_17_H_18_N_2_O_5_SNa [M + Na]^+^ 385.0829; found 385.0832.

**rac-1v**: ^1^H NMR (400 MHz, CDCl_3_) δ 8.11 (dt, *J* = 7.5, 3.8 Hz, 1H), 7.86 (dt, *J* = 7.5, 3.8 Hz, 1H), 7.77–7.69 (m, 2H), 6.83 (d, *J* = 8.1 Hz, 1H), 6.72 (d, *J* = 8.1 Hz, 1H), 5.33 (d, *J* = 6.3 Hz, 1H), 3.83 (s, 6H), 3.34 (tq, *J* = 12.4, 5.7 Hz, 2H), 3.17 (td, *J* = 10.4, 9.3, 5.5 Hz, 1H), 3.01–2.79 (m, 2H), 2.27 (td, *J* = 13.9, 8.2 Hz, 1H), 1.86 (ddd, *J* = 19.0, 8.5, 6.0 Hz, 1H). ^13^C NMR (101 MHz, CDCl_3_) δ 151.9, 145.6, 137.4, 136.7, 133.7, 132.9, 131.3, 125.5, 118.8, 111.4, 60.3, 56.3, 47.8, 44.3, 29.9, 28.2. HR-ESI-MS: calcd for C_18_H_20_N_2_O_6_SNa [M + Na]^+^ 415.0934; found 415.0940.

**rac-1w**: ^1^H NMR (400 MHz, CDCl_3_) δ 8.12 (dt, *J* = 7.4, 3.8 Hz, 1H), 7.86 (dt, *J* = 7.5, 3.8 Hz, 1H), 7.76–7.70 (m, 2H), 7.14–7.05 (m, 4H), 5.38 (t, *J* = 6.1 Hz, 1H), 3.40–3.23 (m, 2H), 3.03 (dq, *J* = 9.8, 4.9 Hz, 1H), 2.74 (t, *J* = 6.8 Hz, 2H), 1.87 (dq, *J* = 10.0, 6.2, 5.5 Hz, 2H), 1.83–1.67 (m, 2H). ^13^C NMR (101 MHz, CDCl_3_) δ 138.1, 136.1, 133.8, 133.7, 133.0, 131.2, 129.7, 128.5, 126.7, 126.0, 125.6, 48.9, 37.8, 29.6, 25.6, 19.4. HR-ESI-MS: calcd for C_17_H_18_N_2_O_4_SNa [M + Na]^+^ 369.0879; found 369.0883.

**rac-1x**: ^1^H NMR (400 MHz, CDCl_3_) δ 8.12 (dt, *J* = 7.5, 3.8 Hz, 1H), 7.86 (dt, *J* = 7.5, 3.8 Hz, 1H), 7.75–7.71 (m, 2H), 7.08 (t, *J* = 7.9 Hz, 1H), 6.70 (dd, *J* = 14.9, 7.9 Hz, 2H), 5.39 (t, *J* = 6.2 Hz, 1H), 3.80 (s, 3H), 3.39–3.23 (m, 2H), 3.01 (dq, *J* = 9.5, 4.8 Hz, 1H), 2.67 (dt, *J* = 17.6, 5.3 Hz, 1H), 2.55 (dt, *J* = 17.5, 7.2 Hz, 1H), 1.89–1.71 (m, 4H). ^13^C NMR (101 MHz, CDCl_3_) δ 157.5, 148.1, 137.4, 133.8, 133.7, 132.9, 131.2, 126.9, 126.3, 125.5, 120.6, 107.9, 55.4, 48.6, 37.9, 25.0, 23.0, 18.6. HR-ESI-MS: calcd for C_18_H_20_N_2_O_5_SNa [M + Na]^+^ 399.0985; found 399.0988.

Nosylamide substrate (0.1 mmol), styrene or substituted styrene (3.0 equiv), Pd(OAc)_2_ (0.2 equiv), Boc-L-lle-OH (0.4 equiv.), AgOAc (2.5 equiv.), K_2_CO_3_ (2.5 equiv.), BQ (0.5 equiv.) and H_2_O (2.0 equiv.) were added to a sealable Schlenk tube and a solution of anhydrous NMP (0.5 mL) in anhydrous t-AmOH (1.5 mL). The Schlenk tube was sealed, and the mixture was stirred vigorously at 60 °C. After 24 h, the reaction was cooled to room temperature, dichloromethane (DCM) was added (5 mL), and the reaction was filtered through a pad of Celite over a plug of silica gel and eluted with DCM (30 mL). The organic layer was washed twice with water, dried over anhydrous sodium sulfate (Na_2_SO_4_), filtered, and concentrated under reduced pressure. The crude material was purified by silica gel column chromatography to obtain the Chiral alkenylation products as white or pale-yellow solids.

**2a**: ^1^H NMR (400 MHz, CDCl_3_) δ 7.92 (d, *J* = 7.8 Hz, 1H), 7.69 (d, *J* = 7.9 Hz, 1H), 7.54–7.50 (m, 3H), 7.40 (q, *J* = 7.7 Hz, 3H), 7.35–7.29 (m, 2H), 7.22 (dd, *J* = 11.9, 4.3 Hz, 2H), 7.15 (d, *J* = 7.4 Hz, 1H), 6.98 (d, *J* = 16.2 Hz, 1H), 5.44 (t, *J* = 5.8 Hz, 1H), 3.80–3.73 (m, 1H), 3.28 (ddd, *J* = 13.1, 5.8, 3.5 Hz, 1H), 3.05–2.89 (m, 3H), 2.29–2.17 (m, 2H). ^13^C NMR (101 MHz, CDCl_3_) δ 147.9, 144.8, 141.0, 137.3, 134.0, 133.5, 133.1, 132.7, 131.4, 130.5, 128.9, 128.2, 128.0, 126.7, 125.5, 125.5, 124.3, 123.1, 47.0, 44.3, 30.9, 28.3. HR-ESI-MS: calcd for C_24_H_22_N_2_O_4_SNa [M + Na]^+^ 457.1192; found 457.1198.

**2b**: ^1^H NMR (400 MHz, CDCl_3_) δ 7.94 (d, *J* = 7.8 Hz, 1H), 7.74 (d, *J* = 7.9 Hz, 1H), 7.57 (t, *J* = 7.8 Hz, 1H), 7.50 (dd, *J* = 8.5, 5.5 Hz, 2H), 7.42 (t, *J* = 8.0 Hz, 2H), 7.21–7.13 (m, 3H), 7.08 (t, *J* = 8.6 Hz, 2H), 6.97 (d, *J* = 16.2 Hz, 1H), 5.59–5.32 (m, 1H), 3.75 (t, *J* = 6.5 Hz, 1H), 3.29 (dd, *J* = 13.2, 3.4 Hz, 1H), 2.96 (ddd, *J* = 29.6, 19.2, 7.4 Hz, 3H), 2.28–2.13 (m, 2H). ^13^C NMR (101 MHz, CDCl_3_) δ 163.8, 161.4, 147.9, 144.8, 141.1, 133.8, 133.6, 132.7, 131.3, 129.2, 128.2, 125.5, 124.3, 123.0, 116.0, 115.8, 46.9, 44.4, 30.8, 28.4. HR-ESI-MS: calcd for C_24_H_21_FN_2_O_4_SNa [M + Na]^+^ 475.1098; found 475.1098.

**2c**: ^1^H NMR (400 MHz, CDCl_3_) δ 7.93 (dd, *J* = 7.9, 1.3 Hz, 1H), 7.73 (dd, *J* = 8.0, 1.2 Hz, 1H), 7.57 (td, *J* = 7.8, 1.4 Hz, 1H), 7.47–7.40 (m, 4H), 7.37–7.32 (m, 2H), 7.24–7.19 (m, 2H), 7.15 (d, *J* = 7.3 Hz, 1H), 6.95 (d, *J* = 16.2 Hz, 1H), 5.45 (t, *J* = 6.0 Hz, 1H), 3.79–3.69 (m, 1H), 3.28 (ddd, *J* = 13.1, 6.2, 3.6 Hz, 1H), 3.04–2.86 (m, 3H), 2.30–2.11 (m, 2H). ^13^C NMR (101 MHz, CDCl_3_) δ 147.9, 144.9, 141.2, 135.9, 133.6, 133.6, 133.4, 132.8, 131.2, 129.1, 128.3, 127.9, 126.2, 125.5, 124.5, 123.0, 47.0, 44.4, 30.8, 28.4. HR-ESI-MS: calcd for C_24_H_21_ClN_2_O_4_SNa [M + Na]^+^ 491.0803; found 491.0798.

**2d**: ^1^H NMR (400 MHz, CDCl_3_) δ 7.96 (dd, *J* = 7.8, 1.2 Hz, 1H), 7.72 (dd, *J* = 8.0, 1.0 Hz, 1H), 7.56 (td, *J* = 7.8, 1.3 Hz, 1H), 7.47–7.38 (m, 4H), 7.31 (t, *J* = 7.7 Hz, 1H), 7.28–7.26 (m, 1H), 7.22 (d, *J* = 5.7 Hz, 1H), 7.20–7.15 (m, 2H), 6.91 (d, *J* = 16.2 Hz, 1H), 5.44 (t, *J* = 5.9 Hz, 1H), 3.79–3.70 (m, 1H), 3.27 (ddd, *J* = 13.0, 6.1, 3.6 Hz, 1H), 3.09–2.88 (m, 3H), 2.29–2.16 (m, 2H). ^13^C NMR (101 MHz, CDCl_3_) δ 147.9, 145.0, 141.2, 139.2, 134.8, 133.6, 133.5, 133.3, 132.7, 131.3, 130.2, 128.9, 128.3, 127.8, 127.0, 126.5, 125.5, 124.9, 124.7, 123.2, 47.0, 44.3, 30.9, 28.4. HR-ESI-MS: calcd for C_24_H_21_ClN_2_O_4_SNa [M + Na]^+^ 491.0803; found 491.0801.

**2e**: ^1^H NMR (400 MHz, CDCl_3_) δ 7.89 (dd, *J* = 7.9, 1.3 Hz, 1H), 7.71 (ddd, *J* = 8.0, 2.9, 1.5 Hz, 2H), 7.55 (td, *J* = 7.8, 1.4 Hz, 1H), 7.45 (d, *J* = 7.6 Hz, 1H), 7.43–7.34 (m, 3H), 7.30 (td, *J* = 7.4, 1.0 Hz, 1H), 7.25–7.15 (m, 4H), 5.41 (t, *J* = 5.9 Hz, 1H), 3.77–3.71 (m, 1H), 3.27 (ddd, *J* = 13.0, 5.9, 3.5 Hz, 1H), 3.06–2.87 (m, 3H), 2.30–2.13 (m, 2H). ^13^C NMR (101 MHz, CDCl_3_) δ 147.9, 144.9, 141.3, 135.4, 133.7, 133.6, 133.2, 132.7, 131.3, 130.0, 129.0, 128.3, 128.1, 127.3, 126.8, 126.3, 125.5, 124.7, 123.5, 47.1, 44.3, 30.9, 28.4. HR-ESI-MS: calcd for C_24_H_21_ClN_2_O_4_SNa [M + Na]^+^ 491.0803; found 491.0805.

**2f**: ^1^H NMR (400 MHz, CDCl_3_) δ 7.93 (dd, *J* = 7.9, 1.3 Hz, 1H), 7.73 (dd, *J* = 8.0, 1.2 Hz, 1H), 7.58 (td, *J* = 7.8, 1.4 Hz, 1H), 7.52–7.47 (m, 2H), 7.45–7.38 (m, 4H), 7.26–7.19 (m, 2H), 7.15 (d, *J* = 7.3 Hz, 1H), 6.93 (d, *J* = 16.2 Hz, 1H), 5.44 (t, *J* = 6.0 Hz, 1H), 3.78–3.71 (m, 1H), 3.28 (ddd, *J* = 13.1, 6.1, 3.6 Hz, 1H), 3.06–2.85 (m, 3H), 2.29–2.12 (m, 2H). ^13^C NMR (101 MHz, CDCl_3_) δ 147.9, 144.9, 141.2, 136.3, 133.6, 133.4, 132.8, 132.0, 131.2, 129.1, 128.2, 126.3, 125.5, 124.5, 123.0, 121.7, 47.0, 44.4, 30.8, 28.4. HR-ESI-MS: calcd for C_24_H_21_BrN_2_O_4_SNa [M + Na]^+^ 535.0298; found 535.0293.

**2g**: ^1^H NMR (400 MHz, CDCl_3_) δ 7.93 (d, *J* = 7.8 Hz, 1H), 7.73 (d, *J* = 7.9 Hz, 1H), 7.63 (s, 4H), 7.56 (t, *J* = 7.7 Hz, 1H), 7.47–7.37 (m, 3H), 7.20 (dd, *J* = 17.8, 7.4 Hz, 2H), 7.04 (d, *J* = 16.2 Hz, 1H), 5.47 (t, *J* = 5.7 Hz, 1H), 3.77 (t, *J* = 6.4 Hz, 1H), 3.29 (dt, *J* = 13.0, 4.8 Hz, 1H), 2.97 (ddd, *J* = 21.4, 17.9, 8.3 Hz, 3H), 2.31–2.15 (m, 2H). ^13^C NMR (101 MHz, CDCl_3_) δ 147.9, 145.0, 141.5, 140.8, 133.6, 133.3, 132.7, 131.2, 128.8, 128.3, 128.1, 126.9, 125.9, 125.5, 124.9, 123.2, 47.0, 44.5, 30.8, 28.5. HR-ESI-MS: calcd for C_25_H_21_F_3_N_2_O_4_SNa [M + Na]^+^ 525.1066; found 525.1065.

**2h**: ^1^H NMR (400 MHz, CDCl_3_) δ 7.95 (dd, *J* = 7.9, 1.4 Hz, 1H), 7.78 (dd, *J* = 8.0, 1.2 Hz, 1H), 7.65 (dd, *J* = 6.9, 1.5 Hz, 4H), 7.61 (dd, *J* = 7.9, 1.4 Hz, 1H), 7.50 (dd, *J* = 7.7, 1.2 Hz, 1H), 7.48–7.41 (m, 2H), 7.21 (dd, *J* = 15.6, 7.4 Hz, 2H), 7.04 (d, *J* = 16.2 Hz, 1H), 5.48 (t, *J* = 6.0 Hz, 1H), 3.77 (td, *J* = 8.2, 4.2 Hz, 1H), 3.28 (dq, *J* = 10.4, 4.2, 3.1 Hz, 1H), 3.05–2.87 (m, 3H), 2.30–2.20 (m, 1H), 2.13 (dd, *J* = 13.3, 7.8 Hz, 1H). ^13^C NMR (101 MHz, CDCl_3_) δ 145.0, 141.9, 141.9, 133.6, 133.0, 132.8, 132.7, 131.0, 129.3, 128.4, 128.3, 127.2, 125.6, 125.2, 123.2, 119.1, 110.9, 47.0, 44.6, 30.8, 28.6. HR-ESI-MS: calcd for C_25_H_21_N_3_O_4_SNa [M + Na]^+^ 482.1145; found 482.1143.

**2i**: ^1^H NMR (400 MHz, CDCl_3_) δ 7.91 (dd, *J* = 7.8, 1.4 Hz, 1H), 7.69 (dd, *J* = 8.0, 1.2 Hz, 1H), 7.53 (td, *J* = 7.8, 1.4 Hz, 1H), 7.51–7.47 (m, 2H), 7.41–7.38 (m, 2H), 7.21 (t, *J* = 7.5 Hz, 1H), 7.16 (d, *J* = 3.6 Hz, 1H), 7.13 (d, *J* = 5.2 Hz, 1H), 7.11–7.07 (m, 2H), 6.93 (d, *J* = 16.1 Hz, 1H), 5.40 (t, *J* = 5.6 Hz, 1H), 3.78–3.70 (m, 1H), 3.25 (dt, *J* = 12.9, 4.4 Hz, 1H), 3.06–2.87 (m, 3H), 2.33 (s, 3H), 2.30–2.19 (m, 2H). ^13^C NMR (101 MHz, CDCl_3_) δ 169.8, 150.4, 144.9, 141.0, 135.1, 133.8, 133.8, 132.9, 132.8, 131.4, 129.4, 128.3, 127.6, 125.8, 125.6, 124.4, 123.1, 122.2, 47.0, 44.4, 30.9, 28.3, 21.3. HR-ESI-MS: calcd for C_26_H_24_N_2_O_6_SNa [M + Na]^+^ 515.1247; found 515.1251.

**2j**: ^1^H NMR (400 MHz, CDCl_3_) δ 7.94 (d, *J* = 7.8 Hz, 1H), 7.70 (d, *J* = 8.0 Hz, 1H), 7.53 (t, *J* = 7.7 Hz, 1H), 7.41 (dd, *J* = 7.6, 3.5 Hz, 3H), 7.35 (t, *J* = 7.7 Hz, 1H), 7.21–7.12 (m, 5H), 6.96 (d, *J* = 16.2 Hz, 1H), 5.43 (t, *J* = 5.9 Hz, 1H), 3.75 (t, *J* = 8.4 Hz, 1H), 3.28 (ddd, *J* = 13.0, 5.8, 3.5 Hz, 1H), 3.04–2.87 (m, 3H), 2.39 (s, 3H), 2.28–2.16 (m, 2H). ^13^C NMR (101 MHz, CDCl_3_) δ 147.9, 144.8, 140.9, 138.0, 134.6, 134.1, 133.5, 132.7, 131.4, 130.4, 129.6, 128.2, 126.7, 125.5, 124.5, 124.1, 122.9, 46.9, 44.3, 30.9, 28.3, 21.4. HR-ESI-MS: calcd for C_25_H_24_N_2_O_4_SNa [M + Na]^+^ 471.1349; found 471.1350.

**2k**: ^1^H NMR (400 MHz, CDCl_3_) δ 7.94 (dd, *J* = 7.9, 1.2 Hz, 1H), 7.68 (dd, *J* = 8.0, 1.1 Hz, 1H), 7.51 (td, *J* = 7.9, 1.3 Hz, 1H), 7.46 (d, *J* = 8.4 Hz, 2H), 7.43–7.39 (m, 3H), 7.32 (td, *J* = 7.8, 1.2 Hz, 1H), 7.22–7.12 (m, 3H), 6.97 (d, *J* = 16.2 Hz, 1H), 5.43 (t, *J* = 6.0 Hz, 1H), 3.78–3.72 (m, 1H), 3.27 (ddd, *J* = 13.0, 5.8, 3.4 Hz, 1H), 3.06–2.87 (m, 3H), 2.30–2.16 (m, 2H), 1.36 (s, 9H). ^13^C NMR (101 MHz, CDCl_3_) δ 151.3, 147.9, 144.8, 140.9, 134.6, 134.2, 133.5, 133.2, 132.7, 131.5, 130.3, 128.2, 126.5, 125.8, 125.5, 124.7, 124.1, 123.0, 46.9, 44.3, 34.8, 31.5, 30.9, 28.3. HR-ESI-MS: calcd for C_28_H_30_N_2_O_4_SNa [M + Na]^+^ 513.1818; found 513. 1815.

**2l**: ^1^H NMR (400 MHz, CDCl_3_) δ 7.96 (dd, *J* = 7.9, 1.4 Hz, 1H), 7.69 (dd, *J* = 8.0, 1.2 Hz, 1H), 7.66–7.62 (m, 4H), 7.59 (d, *J* = 8.4 Hz, 2H), 7.52–7.43 (m, 4H), 7.40–7.33 (m, 2H), 7.26–7.20 (m, 2H), 7.15 (d, *J* = 7.3 Hz, 1H), 7.02 (d, *J* = 16.2 Hz, 1H), 5.44 (t, *J* = 5.7 Hz, 1H), 3.81–3.74 (m, 1H), 3.30 (dt, *J* = 13.0, 4.5 Hz, 1H), 3.06–2.87 (m, 3H), 2.31–2.15 (m, 2H). ^13^C NMR (101 MHz, CDCl_3_) δ 147.9, 144.9, 141.1, 140.7, 140.6, 136.4, 134.0, 133.5, 133.2, 132.7, 131.4, 130.0, 129.1, 128.3, 127.7, 127.5, 127.2, 127.0, 125.6, 125.5, 124.3, 123.1, 47.0, 44.4, 30.9, 28.4. HR-ESI-MS: calcd for C_30_H_26_N_2_O_4_SNa [M + Na]^+^ 533.1505; found 533.1505.

**2m**: ^1^H NMR (400 MHz, CDCl_3_) δ 7.94 (dd, *J* = 7.8, 1.0 Hz, 1H), 7.71 (d, *J* = 8.0 Hz, 1H), 7.57–7.51 (m, 1H), 7.46 (d, *J* = 8.7 Hz, 2H), 7.41–7.34 (m, 2H), 7.19 (t, *J* = 7.5 Hz, 1H), 7.14–7.05 (m, 2H), 6.97–6.90 (m, 3H), 5.43 (t, *J* = 6.0 Hz, 1H), 3.85 (s, 3H), 3.75 (t, *J* = 8.1 Hz, 1H), 3.28 (ddd, *J* = 13.1, 5.9, 3.4 Hz, 1H), 3.07–2.85 (m, 3H), 2.30–2.13 (m, 2H). ^13^C NMR (101 MHz, CDCl_3_) δ 159.6, 147.9, 144.7, 140.8, 134.2, 133.5, 133.2, 132.8, 131.4, 130.1, 130.0, 128.2, 128.0, 125.5, 123.9, 123.3, 122.8, 114.3, 55.5, 46.9, 44.3, 30.9, 28.3. HR-ESI-MS: calcd for C_25_H_24_N_2_O_5_SNa [M + Na]^+^ 487.1298; found 487.1300.

**2n**: ^1^H NMR (400 MHz, CDCl_3_) δ 7.94 (dd, *J* = 7.9, 1.3 Hz, 1H), 7.71 (dd, *J* = 8.0, 1.2 Hz, 1H), 7.53 (td, *J* = 7.8, 1.4 Hz, 1H), 7.43 (d, *J* = 8.5 Hz, 2H), 7.39 (d, *J* = 7.6 Hz, 1H), 7.35 (td, *J* = 7.7, 1.2 Hz, 1H), 7.20 (t, *J* = 7.5 Hz, 1H), 7.12 (dd, *J* = 11.8, 4.4 Hz, 2H), 7.03–7.00 (m, 2H), 6.94 (d, *J* = 16.2 Hz, 1H), 5.43 (t, *J* = 5.9 Hz, 1H), 3.79–3.71 (m, 1H), 3.28 (ddd, *J* = 13.1, 5.9, 3.4 Hz, 1H), 3.04–2.87 (m, 3H), 2.28–2.14 (m, 2H), 1.38 (s, 9H). ^13^C NMR (101 MHz, CDCl_3_) δ 155.6, 147.9, 144.8, 140.8, 134.2, 133.5, 133.1, 132.7, 132.5, 131.4, 130.0, 128.2, 127.3, 125.5, 124.5, 124.2, 124.0, 122.9, 121.9, 79.1, 46.9, 44.3, 30.8, 29.0, 28.3. HR-ESI-MS: calcd for C_28_H_30_N_2_O_5_SNa [M + Na]^+^ 529.1768; found 529.1770.

**2o**: ^1^H NMR (400 MHz, CDCl_3_) δ 7.92 (dd, *J* = 7.9, 1.3 Hz, 1H), 7.75 (dd, *J* = 8.0, 1.2 Hz, 1H), 7.59 (td, *J* = 7.8, 1.4 Hz, 1H), 7.46 (d, *J* = 8.4 Hz, 2H), 7.43–7.38 (m, 2H), 7.37–7.33 (m, 2H), 7.17 (d, *J* = 16.2 Hz, 1H), 6.90 (q, *J* = 8.1 Hz, 2H), 5.47 (t, *J* = 6.1 Hz, 1H), 3.77 (s, 1H), 3.26 (ddd, *J* = 13.4, 6.1, 3.7 Hz, 1H), 3.01–2.91 (m, 3H), 2.36–2.25 (m, 1H), 2.24–2.16 (m, 1H). HR-ESI-MS: calcd for C_24_H_20_ClFN_2_O_4_SNa [M + Na]^+^ 509.0709; found 509.0712.

**2p**: ^1^H NMR (400 MHz, CDCl_3_) δ 7.93 (d, *J* = 7.9 Hz, 1H), 7.76 (d, *J* = 8.0 Hz, 1H), 7.62–7.57 (m, 1H), 7.47 (d, *J* = 8.6 Hz, 2H), 7.45–7.41 (m, 1H), 7.36 (d, *J* = 8.5 Hz, 2H), 7.18 (d, *J* = 16.2 Hz, 1H), 7.09 (dd, *J* = 10.4, 1.9 Hz, 1H), 6.94 (d, *J* = 16.2 Hz, 1H), 6.83 (d, *J* = 8.3 Hz, 1H), 5.47 (t, *J* = 6.0 Hz, 1H), 3.69 (t, *J* = 6.5 Hz, 1H), 3.24 (ddd, *J* = 13.3, 6.0, 3.7 Hz, 1H), 3.03–2.83 (m, 3H), 2.31–2.15 (m, 2H). ^13^C NMR (101 MHz, CDCl_3_) δ 147.9, 147.2, 147.1, 136.9, 135.4, 134.0, 133.6, 132.8, 131.2, 130.1, 129.2, 128.1, 125.5, 125.2, 111.4, 109.3, 47.0, 43.8, 30.9, 28.8. HR-ESI-MS: calcd for C_24_H_20_ClFN_2_O_4_SNa [M + Na]^+^ 509.0709; found 509.0711.

**2q**: ^1^H NMR (400 MHz, CDCl_3_) δ 7.82 (d, *J* = 7.9 Hz, 1H), 7.68 (d, *J* = 8.0 Hz, 1H), 7.52 (t, *J* = 7.8 Hz, 1H), 7.40 (d, *J* = 8.4 Hz, 2H), 7.35 (t, *J* = 7.7 Hz, 1H), 7.29 (d, *J* = 8.4 Hz, 2H), 7.00–6.95 (m, 2H), 6.92–6.83 (m, 2H), 5.40 (t, *J* = 5.8 Hz, 1H), 3.71 (t, *J* = 7.2 Hz, 1H), 3.22 (dt, *J* = 13.1, 4.5 Hz, 1H), 2.96–2.75 (m, 3H), 2.26–2.13 (m, 2H). HR-ESI-MS: calcd for C_24_H_20_ClFN_2_O_4_SNa [M + Na]^+^ 509.0709; found 509.0714.

**2r**: ^1^H NMR (400 MHz, CDCl_3_) δ 7.91 (dd, *J* = 7.8, 1.1 Hz, 1H), 7.77–7.74 (m, 1H), 7.61 (td, *J* = 7.9, 1.2 Hz, 1H), 7.52 (s, 1H), 7.49–7.44 (m, 3H), 7.36 (d, *J* = 8.5 Hz, 2H), 7.25 (s, 1H), 7.15 (d, *J* = 16.2 Hz, 1H), 6.95 (d, *J* = 16.2 Hz, 1H), 5.50 (t, *J* = 6.1 Hz, 1H), 3.72–3.64 (m, 1H), 3.24 (ddd, *J* = 13.4, 5.8, 3.9 Hz, 1H), 3.03–2.83 (m, 3H), 2.27–2.14 (m, 2H). ^13^C NMR (101 MHz, CDCl_3_) δ 147.9, 147.1, 140.9, 135.4, 135.3, 134.0, 133.6, 133.4, 132.8, 131.1, 130.3, 129.2, 128.1, 127.3, 125.8, 125.6, 124.8, 122.1, 47.1, 44.2, 30.7, 28.5. HR-ESI-MS: calcd for C_24_H_20_BrClN_2_O_4_SNa [M + Na]^+^ 568.9908; found 568.9913.

**2s**: ^1^H NMR (400 MHz, CDCl_3_) δ 7.93–7.89 (m, 1H), 7.75 (d, *J* = 8.0 Hz, 1H), 7.63–7.58 (m, 1H), 7.46 (t, *J* = 9.4 Hz, 3H), 7.36 (d, *J* = 8.6 Hz, 3H), 7.18–7.09 (m, 2H), 6.96 (d, *J* = 16.2 Hz, 1H), 5.49 (t, *J* = 6.0 Hz, 1H), 3.73–3.64 (m, 1H), 3.24 (ddd, *J* = 13.4, 5.9, 3.8 Hz, 1H), 3.02–2.84 (m, 3H), 2.29–2.15 (m, 2H). ^13^C NMR (101 MHz, CDCl_3_) δ 147.9, 146.8, 139.8, 135.4, 134.9, 134.0, 134.0, 133.6, 133.4, 132.8, 131.1, 130.3, 129.2, 128.1, 125.6, 124.9, 124.4, 122.9, 46.8, 44.1, 30.7, 28.5. HR-ESI-MS: calcd for C_24_H_20_Cl_2_N_2_O_4_SNa [M + Na]^+^ 525.0413; found 525.0412.

**2t**: ^1^H NMR (400 MHz, CDCl_3_) δ 7.93 (dd, *J* = 7.8, 1.2 Hz, 1H), 7.73 (dd, *J* = 8.0, 1.0 Hz, 1H), 7.57 (td, *J* = 7.8, 1.3 Hz, 1H), 7.47–7.40 (m, 3H), 7.34 (d, *J* = 8.5 Hz, 2H), 7.23–7.14 (m, 2H), 6.99–6.90 (m, 2H), 5.41 (t, *J* = 6.0 Hz, 1H), 3.69 (t, *J* = 6.3 Hz, 1H), 3.26 (ddd, *J* = 13.0, 6.1, 3.6 Hz, 1H), 2.95 (ddt, *J* = 15.2, 8.9, 5.6 Hz, 2H), 2.84 (dd, *J* = 16.3, 7.6 Hz, 1H), 2.34 (s, 3H), 2.29–2.10 (m, 2H). ^13^C NMR (101 MHz, CDCl_3_) δ 147.9, 145.1, 138.4, 138.0, 135.9, 133.5, 133.5, 133.4, 133.3, 132.8, 131.3, 129.1, 128.8, 127.9, 126.2, 125.5, 125.4, 123.7, 47.1, 44.0, 30.7, 28.7, 21.5. HR-ESI-MS: calcd for C_25_H_23_ClN_2_O_4_SNa [M + Na]^+^ 505.0959; found 505.0961.

**2u**: ^1^H NMR (400 MHz, CDCl_3_) δ 7.93 (dd, *J* = 7.8, 1.2 Hz, 1H), 7.73 (dd, *J* = 7.9, 1.0 Hz, 1H), 7.57 (td, *J* = 7.8, 1.3 Hz, 1H), 7.44 (t, *J* = 7.8 Hz, 3H), 7.34 (d, *J* = 8.5 Hz, 2H), 7.16 (d, *J* = 16.2 Hz, 1H), 6.92 (dd, *J* = 9.1, 7.1 Hz, 2H), 6.72 (s, 1H), 5.42 (t, *J* = 6.0 Hz, 1H), 3.82 (s, 3H), 3.66 (t, *J* = 7.3 Hz, 1H), 3.25 (ddd, *J* = 13.0, 6.2, 3.7 Hz, 1H), 2.99–2.81 (m, 3H), 2.28–2.11 (m, 2H). ^13^C NMR (101 MHz, CDCl_3_) δ 160.1, 147.9, 146.5, 135.7, 134.2, 133.7, 133.6, 133.5, 133.4, 132.8, 131.2, 129.2, 129.1, 127.9, 126.1, 125.5, 110.2, 108.5, 55.6, 47.3, 43.6, 31.0, 28.8. HR-ESI-MS: calcd for C_25_H_23_ClN_2_O_5_SNa [M + Na]^+^ 521.0908; found 521.0911.

**2v**: ^1^H NMR (400 MHz, CDCl_3_) δ 7.94 (dd, *J* = 7.9, 1.3 Hz, 1H), 7.76–7.72 (m, 1H), 7.60–7.56 (m, 1H), 7.47–7.42 (m, 3H), 7.36–7.32 (m, 2H), 7.15 (d, *J* = 16.2 Hz, 1H), 6.96 (s, 1H), 6.85 (d, *J* = 16.2 Hz, 1H), 5.45 (t, *J* = 6.0 Hz, 1H), 3.90 (s, 3H), 3.85 (s, 3H), 3.72–3.64 (m, 1H), 3.24 (ddd, *J* = 13.1, 6.1, 3.8 Hz, 1H), 2.95 (ddd, *J* = 13.2, 9.2, 6.7 Hz, 3H), 2.26–2.12 (m, 2H). ^13^C NMR (101 MHz, CDCl_3_) δ 152.4, 147.9, 145.8, 137.4, 135.9, 135.4, 133.6, 133.5, 133.4, 132.8, 131.2, 129.1, 128.8, 127.9, 127.8, 126.0, 125.5, 107.6, 60.4, 56.3, 47.2, 44.1, 28.9, 27.8.; HR-ESI-MS: calcd for C_26_H_25_ClN_2_O_6_SNa [M + Na]^+^ 551.1014; found 551.1017.

**2w**: ^1^H NMR (400 MHz, CDCl_3_) δ 7.75 (dd, *J* = 7.9, 1.1 Hz, 1H), 7.57–7.46 (m, 4H), 7.41–7.30 (m, 4H), 7.15 (t, *J* = 7.7 Hz, 1H), 7.03 (d, *J* = 7.5 Hz, 1H), 6.86 (d, *J* = 15.9 Hz, 1H), 5.48 (t, *J* = 6.1 Hz, 1H), 3.46 (d, *J* = 11.4 Hz, 1H), 3.18 (dt, *J* = 13.5, 4.3 Hz, 1H), 3.00 (ddd, *J* = 13.5, 11.4, 7.1 Hz, 1H), 2.81 (t, *J* = 5.8 Hz, 2H), 2.28–2.21 (m, 1H), 1.85–1.69 (m, 4H). ^13^C NMR (101 MHz, CDCl_3_) δ 147.9, 138.0, 136.6, 136.0, 134.4, 133.6, 133.5, 133.2, 132.7, 131.4, 130.4, 130.0, 129.5, 129.1, 128.1, 127.0, 126.7, 125.5, 124.2, 46.7, 35.4, 29.9, 24.0, 17.6. HR-ESI-MS: calcd for C_25_H_23_ClN_2_O_4_SNa [M + Na]^+^ 505.0959; found 505.0962.

**2x**: ^1^H NMR (400 MHz, CDCl_3_) δ 7.79–7.75 (m, 1H), 7.66 (d, *J* = 7.9 Hz, 1H), 7.57–7.51 (m, 1H), 7.50–7.38 (m, 4H), 7.33 (dd, *J* = 14.7, 8.1 Hz, 3H), 6.80–6.72 (m, 2H), 5.47 (t, *J* = 6.0 Hz, 1H), 3.82 (s, 3H), 3.48–3.41 (m, 1H), 3.20 (dt, *J* = 13.5, 4.4 Hz, 1H), 3.02 (ddd, *J* = 13.4, 11.6, 7.0 Hz, 1H), 2.85 (dd, *J* = 16.9, 4.3 Hz, 1H), 2.48 (ddd, *J* = 17.8, 10.9, 6.8 Hz, 1H), 2.21 (d, *J* = 9.9 Hz, 1H), 1.88–1.81 (m, 1H), 1.69 (ddt, *J* = 19.4, 8.4, 5.1 Hz, 2H). ^13^C NMR (101 MHz, CDCl_3_) δ 157.4, 147.9, 136.3, 135.6, 133.5, 133.2, 133.1, 132.7, 131.4, 129.0, 128.8, 128.2, 127.9, 126.6, 125.4, 124.7, 108.4, 55.5, 46.4, 35.4, 23.4, 23.2, 16.7. HR-ESI-MS: calcd for C_26_H_25_ClN_2_O_5_SNa [M + Na]^+^ 535.1065; found 535.1074.

**3**: ^1^H NMR (400 MHz, CDCl_3_) δ 7.93 (d, *J* = 7.8 Hz, 1H), 7.70 (d, *J* = 7.9 Hz, 1H), 7.52 (dd, *J* = 12.2, 7.6 Hz, 3H), 7.37 (q, *J* = 7.7 Hz, 3H), 7.33–7.28 (m, 1H), 7.15 (d, *J* = 16.2 Hz, 1H), 6.97 (s, 1H), 6.89 (d, *J* = 16.2 Hz, 1H), 5.43 (t, *J* = 5.9 Hz, 1H), 3.91 (s, 3H), 3.85 (s, 3H), 3.74–3.66 (m, 1H), 3.23 (ddd, *J* = 13.0, 5.7, 3.6 Hz, 1H), 2.98–2.93 (m, 2H), 2.31–2.13 (m, 2H). ^13^C NMR (101 MHz, CDCl_3_) δ 152.4, 147.9, 145.5, 137.3, 135.2, 133.5, 133.2, 132.7, 131.4, 129.3, 129.1, 129.0, 127.9, 126.6, 125.5, 125.4, 107.6, 60.4, 56.8, 48.0, 44.0, 29.2, 27.8. HR-ESI-MS: calcd for C_26_H_26_N_2_O_6_SNa [M + Na]^+^ 517.1404; found 517.1408.

**5**: A solution of osmium tetroxide (1.0 mL, 2.5 mg mL^−1^ in t-BuOH, 25 mg, 0.098 mmol, 1 mol%) was added to a stirred solution of **3** (494 mg, 1.0 mmol) and N-methyl-morpholine-N-oxide (354 mg, 3.0 mmol) at room temperature. The layers were shaken and separated, and the aqueous phase was extracted with EtOAc (3x5 mL). The organic layer was dried and concentrated. The residue was purified by silica gel column chromatography to give a pale yellow solid **5** (436 mg, 80%). ^1^H NMR (400 MHz, CDCl_3_) δ 8.04–7.98 (m, 1H), 7.84–7.79 (m, 1H), 7.70 (q, *J* = 5.4, 4.3 Hz, 2H), 7.15 (q, *J* = 5.6 Hz, 3H), 6.99 (d, *J* = 5.3 Hz, 3H), 5.48 (s, 1H), 4.73–4.61 (m, 2H), 3.86 (s, 3H), 3.77 (s, 3H), 3.57 (d, *J* = 12.3 Hz, 1H), 3.31 (s, 1H), 2.94 (d, *J* = 5.7 Hz, 2H), 2.72–2.63 (m, 2H), 2.37 (q, *J* = 7.1 Hz, 1H). ^13^C NMR (100 MHz, CDCl_3_) δ 152.1, 147.9, 145.1, 139.6, 136.2, 135.7, 133.7, 133.0, 131.4, 131.0, 128.1, 127.9, 126.9, 125.4, 109.3, 79.3, 76.0, 60.3, 56.2, 46.6, 43.0, 29.6, 27.3. HR-ESI-MS: calcd for C_26_H_28_N_2_O_8_SNa [M + Na]^+^ 551.1459; found 551.1463.

**6**: Dess–Martin periodinane (254 mg, 0.6 mmol) was added to a solution of **5** (54 mg, 0.1 mmol) in DCM at 0^o^C. Then, the resulting reaction mixture was stirred at room temperature for 2 h and monitored by TLC. Upon completion, the reaction was neutralized with sodium sulfite solution and sodium bicarbonate solution at 0^o^C and extracted with EtOAc. The organic layer was dried and concentrated. The residue was purified by silica gel column chromatography to present the target molecule as a pale-yellow solid **6** (38 mg, 70%). ^1^H NMR (400 MHz, CDCl_3_) δ 8.13–8.08 (m, 1H), 7.94–7.89 (m, 2H), 7.86–7.82 (m, 1H), 7.72–7.63 (m, 3H), 7.51 (t, *J* = 7.7 Hz, 2H), 7.00 (s, 1H), 5.61 (t, *J* = 6.1 Hz, 1H), 4.03–3.98 (m, 1H), 3.97 (s, 3H), 3.72 (s, 3H), 3.35 (dt, *J* = 10.8, 5.3 Hz, 1H), 3.22 (ddd, *J* = 12.4, 7.8, 6.6 Hz, 1H), 2.98 (dqt, *J* = 14.3, 8.8, 4.8 Hz, 2H), 2.31–2.17 (m, 2H). ^13^C NMR (100 MHz, CDCl_3_) δ 194.9, 194.8, 151.4, 151.1, 141.5, 139.2, 135.0, 133.7, 133.5, 133.1, 132.9, 131.3, 130.1, 129.2, 125.5, 123.8, 116.0, 60.5, 56.4, 46.9, 44.8, 29.5, 27.6. HR-ESI-MS: calcd for C_26_H_24_N_2_O_8_SNa [M + Na]^+^ 547.1146; found 547.1144.

**7**: K_2_CO_3_ (28 mg, 0.2 mmol) and p-Thiocresol (25 mg, 0.2 mmol) were added to a solution of **6** (54 mg, 0.1 mmol) in DMF (1 mL). The reaction solution was stirred at room temperature and monitored by TLC. Upon completion, the reaction was then poured into 10 mL of water and extracted with EtOAc. The organic layer was combined and concentrated under reduced pressure, and the crude product was purified by silica gel column chromatography using petroleum ether/EtOAc (10→20%) as an eluent to give a pale yellow solid **7** (26 mg, 81%). ^1^H NMR (400 MHz, CDCl_3_) δ 8.04 (d, *J* = 7.4 Hz, 2H), 7.63 (t, *J* = 7.4 Hz, 1H), 7.49 (t, *J* = 7.7 Hz, 2H), 6.91 (s, 1H), 4.57 (d, *J* = 8.7 Hz, 1H), 4.01 (s, 3H), 4.01–3.94 (m, 1H), 3.79 (s, 3H), 3.29 (d, *J* = 7.2 Hz, 2H), 3.20 (dd, *J* = 15.8, 8.6 Hz, 1H), 3.10–3.00 (m, 1H), 2.54 (d, *J* = 6.2 Hz, 1H). HR-ESI-MS: calcd for C_20_H_19_NO_3_Na [M + Na]^+^ 344.1257; found 344.1253.

## Figures and Tables

**Table 1:** Optimization of conditions for the kinetic resolution of β-alkylphenyl ethylamine.

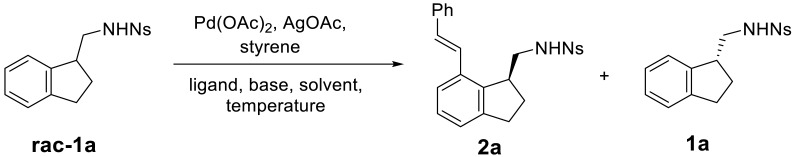					
Entry	Conditions	Yield of 2	ee of 2	Yield of 1	ee of 1
1	Boc-L-t-Leu-OH, K_2_CO_3_, t-AmOH/NMP(3:1), 60 °C	30%	88%	57%	28%
2	Boc-L-t-Leu-OH, K_2_CO_3_, H_2_O(2.0 equiv.), 60 °C t-AmOH/NMP(3:1)	36%	92%	55%	45%
3	Boc-L-t-Leu-OH, K_2_CO_3_, H_2_O(4.0 equiv.), 60 °C t-AmOH/NMP(3:1)	35%	91%	58%	64%
4	Boc-L-t-Leu-OH, K_2_CO_3_, H_2_O(2.0 equiv.), 80 ^o^C t-AmOH/NMP(3:1)	41%	78%	41%	20%
5	Boc-L-t-Leu-OH, K_2_CO_3_, H_2_O(2.0 equiv.), 60 °C t-AmOH/NMP(3:1), BQ	36%	93%	56%	62%
**6**	**Boc-L-lle-OH**, **K_2_CO_3_**, **H_2_O(2.0 equiv.)**, **60 °C** **t-AmOH/NMP(3:1)**, **BQ**	**42%**	**94%**	**49%**	**64%**
7	Ac-L-lle-OH, K_2_CO_3_, H_2_O(2.0 equiv.), 60 °C t-AmOH/NMP(3:1), BQ	36%	90%	53%	48%
8	Boc-L-lle-OH, Na_2_CO_3_, H_2_O(2.0 equiv.), 60 °C t-AmOH/NMP(3:1), BQ	38%	92%	56%	62%
9	Boc-L-lle-OH, Na_3_PO_4_, H_2_O(2.0 equiv.), 60 °C t-AmOH/NMP(3:1), BQ	31%	90%	60%	53%

Reactions were carried out in the anhydrous t-AmOH/NMP (3:1, 0.05 M) solvent at the indicated temperature for 24 h, in the presence of 20 mol% of Pd(OAc)_2_, 40 mol% of the indicated MPAA ligand, 3.0 equiv. of styrene, 2.5 equiv. of AgOAc, 2.5 equiv. of the indicated inorganic base, the indicated 0.5 equiv. of BQ and the indicated equiv. of H_2_O. The enantiomeric excesses were determined by HPLC with a chiral stationary phase.

## Data Availability

Not applicable.

## Supplementary Materials

The following supporting information can be downloaded at: https://www.mdpi.com/article/10.3390/molecules28041852/s1, copies of the ^1^H NMR and ^13^C NMR spectra and HPLC data for all newly synthesized compounds.

## Sample Availability

Samples of the compounds are available from the authors.
