# Supplementary material for: Kinetic Resolution of β-Alkyl Phenylethylamine Derivatives through Palladium-Catalyzed, Nosylamide-Directed C−H Olefination
Source: Molecules. 2023 Feb 15;28(4):1852. doi: 10.3390/molecules28041852 (PMC9967062; doi:10.3390/molecules28041852)
Supplement: Supplementary file 1 [file molecules-28-01852-s001.zip › molecules-2187702-supplementary.pdf]

Table of Contents

1. Figure S1 to Figure S22 The ^{1}H spectrum and ^{13}C spectrum of **rac-1a** and **rac-1o-x**.
2. Figure S23 to Figure S68 The ^{1}H spectrum and ^{13}C spectrum of **2a-x**.
3. Figure S69 to Figure S75 The ^{1}H spectrum and ^{13}C spectrum of **3**, **5**, **6** and **7**.
4. Figure S76 and Figure S77 The HPLC data of **1a** and **2a** of entry 6 in Table 1.
5. Figure S78 to Figure S103 The HPLC Data of **1ab-an** and **2b-n** in Scheme 1.
6. Figure S104 to Figure S123 The HPLC Data of **1o-x** and **2o-x** in Scheme 2.
7. Figure S124 to Figure S126 The HPLC data of **3, 1v** and **4** in Scheme 3.

1 The ^{1}H spectrum and ^{13}C spectrum of **rac-1a** and **rac-1o-x**.

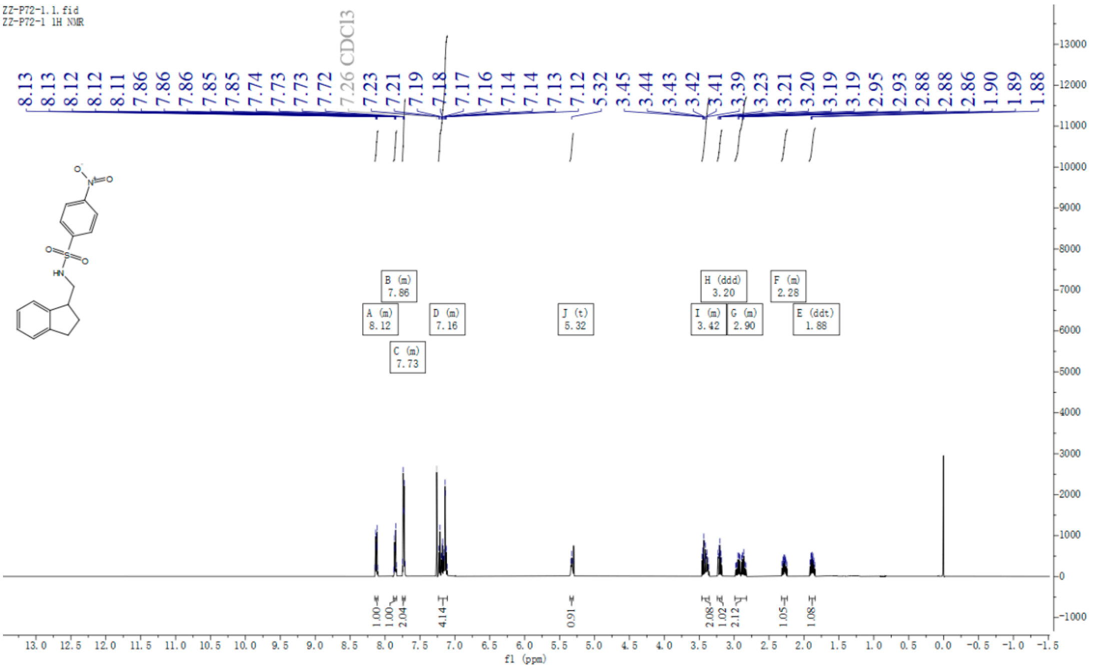

Figure S1. The ^{1}H spectrum (500MHz, $CDCl_3$) of **rac-1a**.

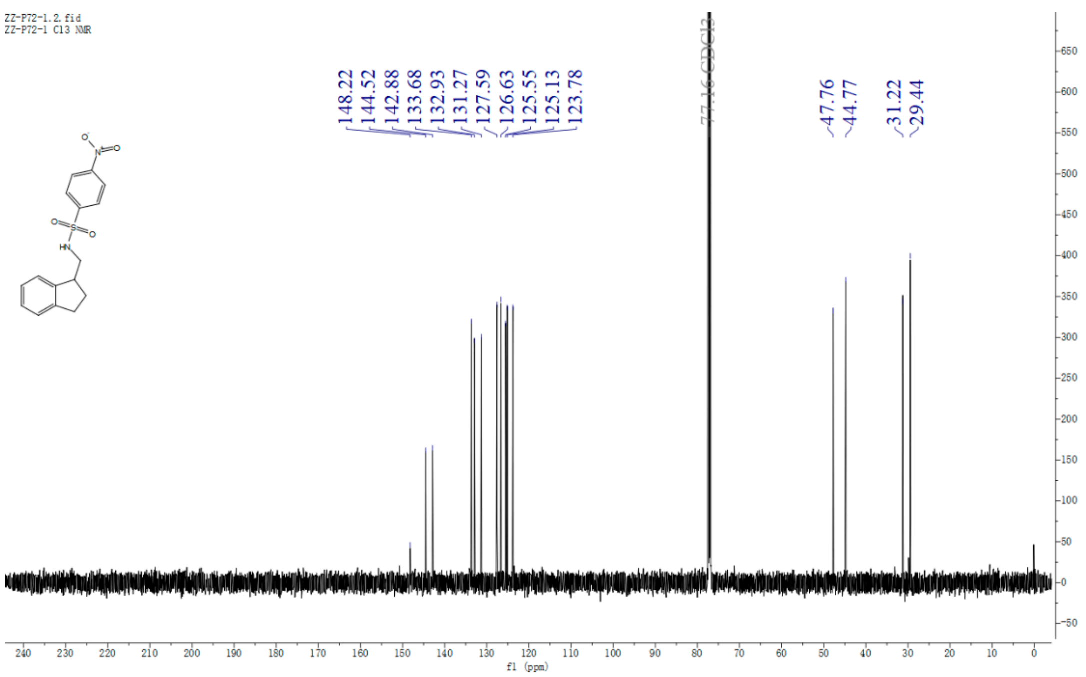

Figure S2. The ^{13}C spectrum (126MHz, $CDCl_3$) of **rac-1a**.

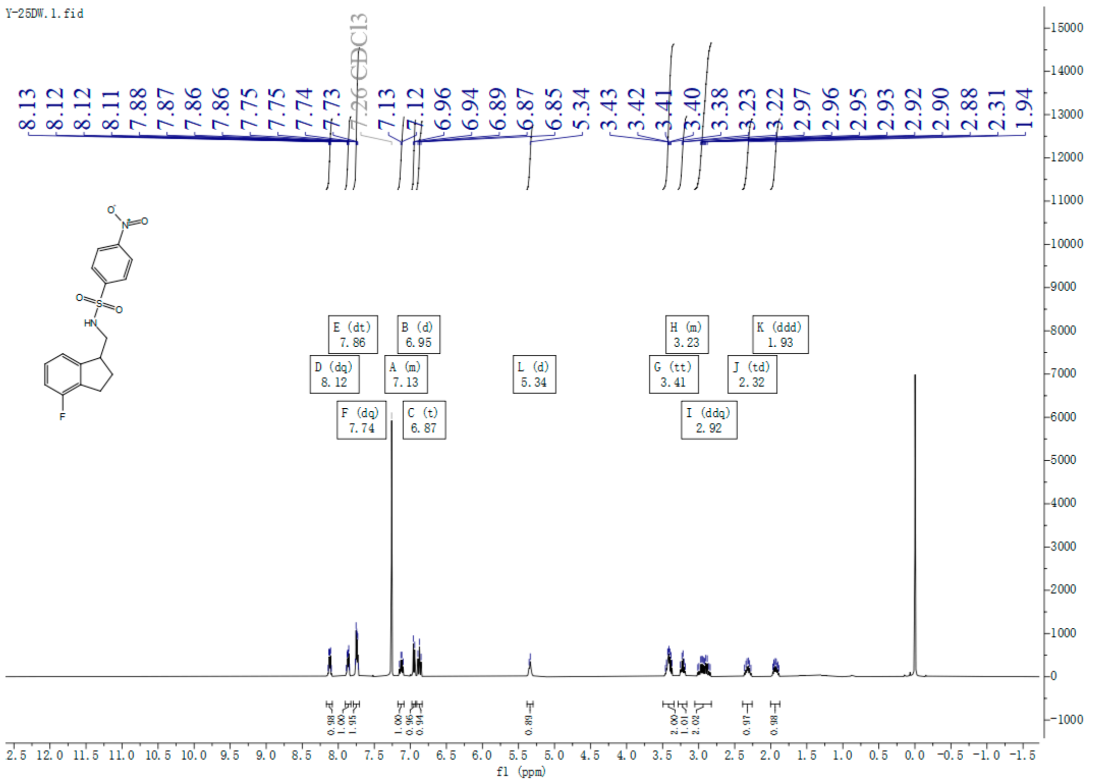

Figure S3. The ^{1}H spectrum (400MHz, $CDCl_3$) of **rac-1o**.

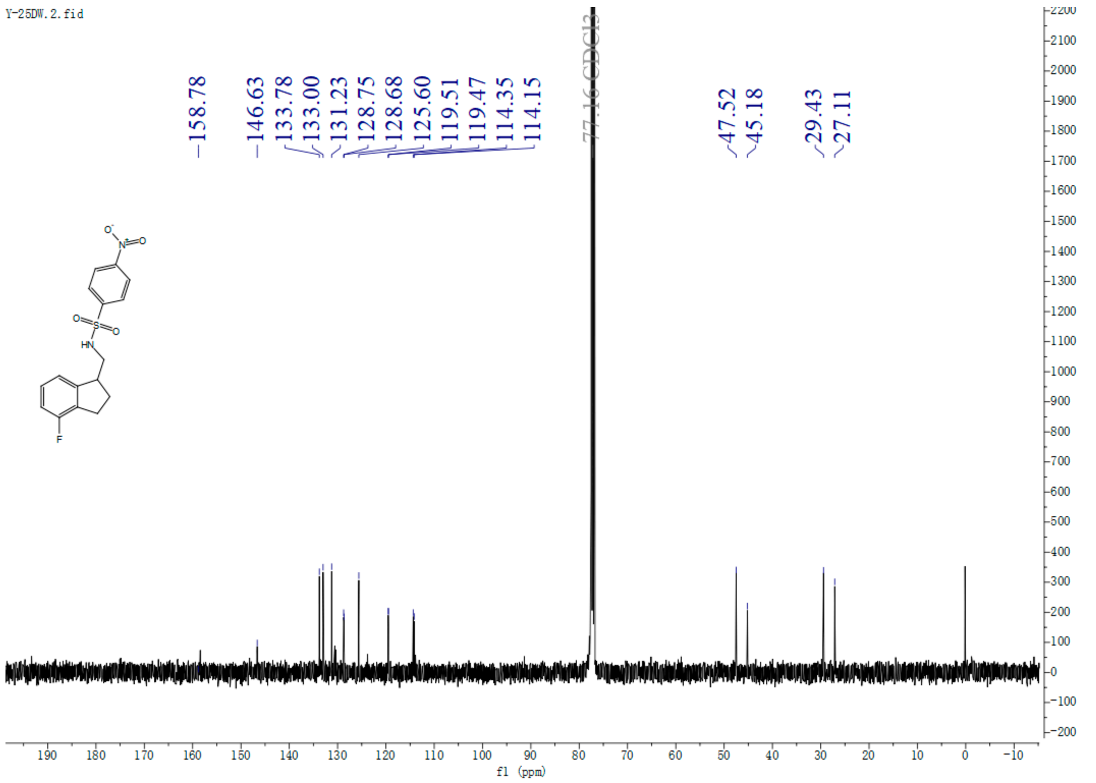

Figure S4. The ^{13}C spectrum (101MHz, $CDCl_3$) of **rac-1o**.

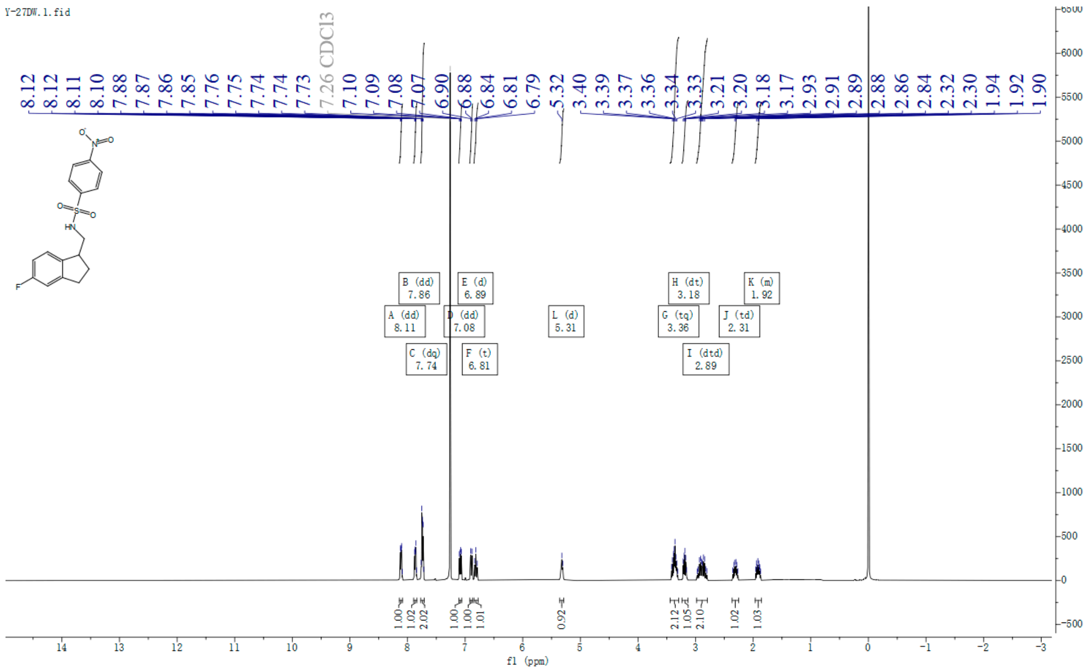

Figure S5. The ^{1}H spectrum (400MHz, $CDCl_3$) of **rac-1p**.

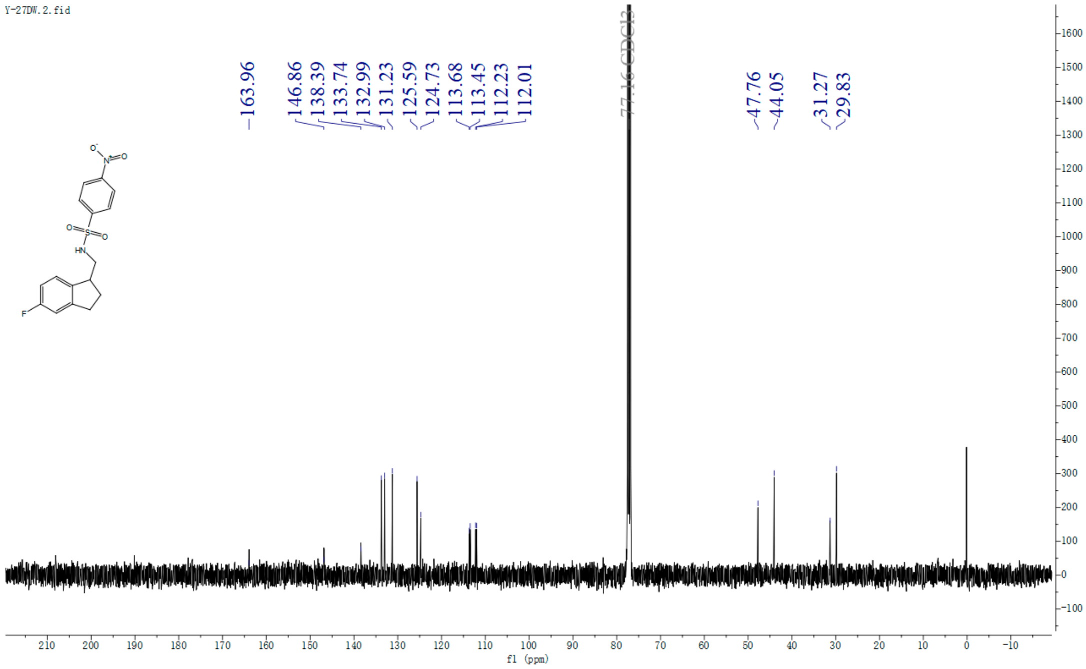

Figure S6. The ^{13}C spectrum (101MHz, $CDCl_3$) of **rac-1p**.

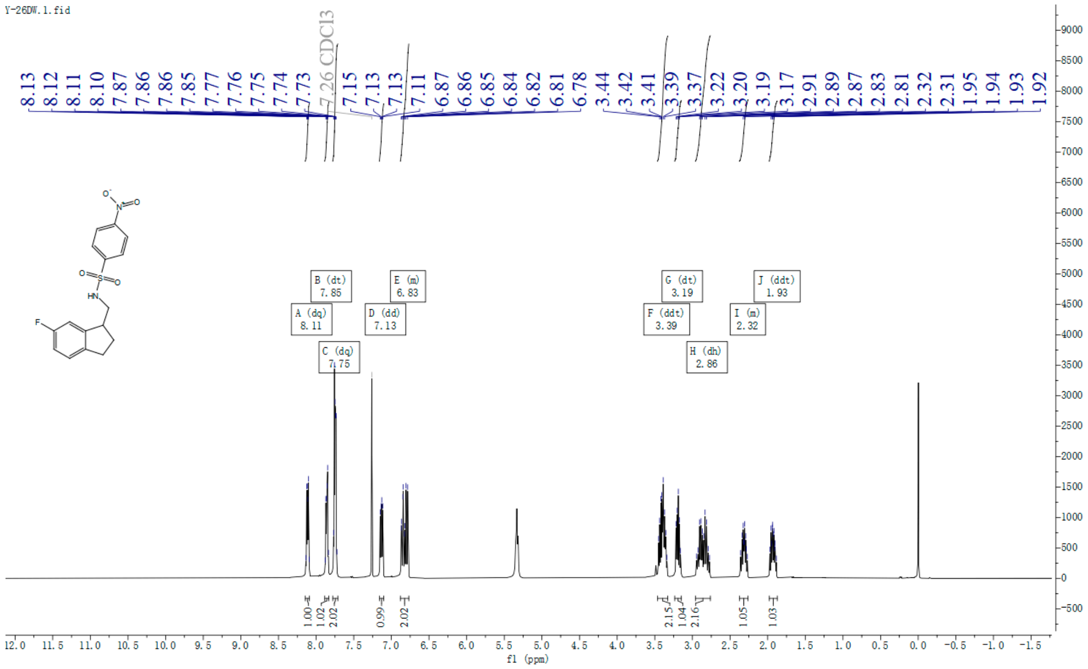

Figure S7. The ^{1}H spectrum (400MHz, $CDCl_3$) of **rac-1q**.

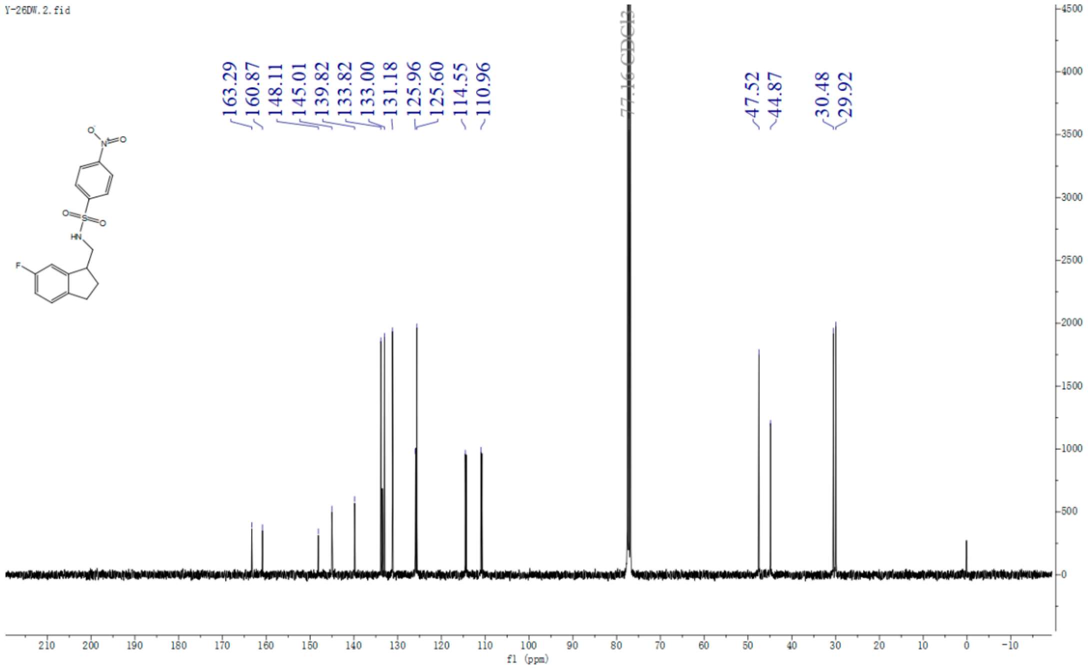

Figure S8. The ^{13}C spectrum (101MHz, $CDCl_3$) of **rac-1q**.

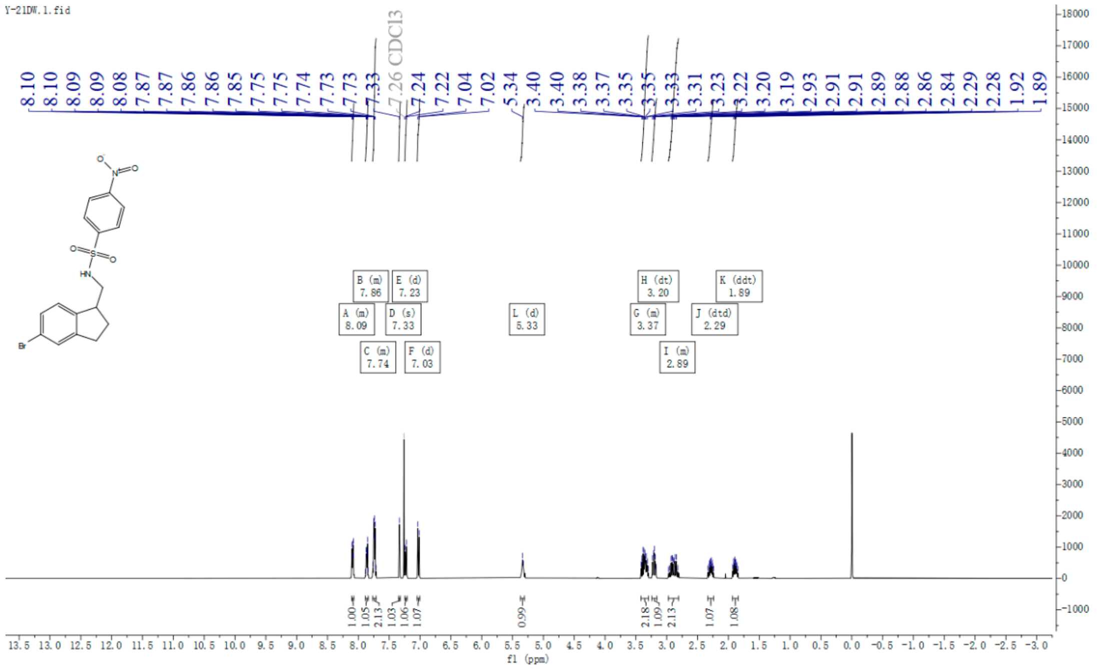

Figure S9. The ¹H spectrum (400MHz, $CDCl_3$) of **rac-1r**.

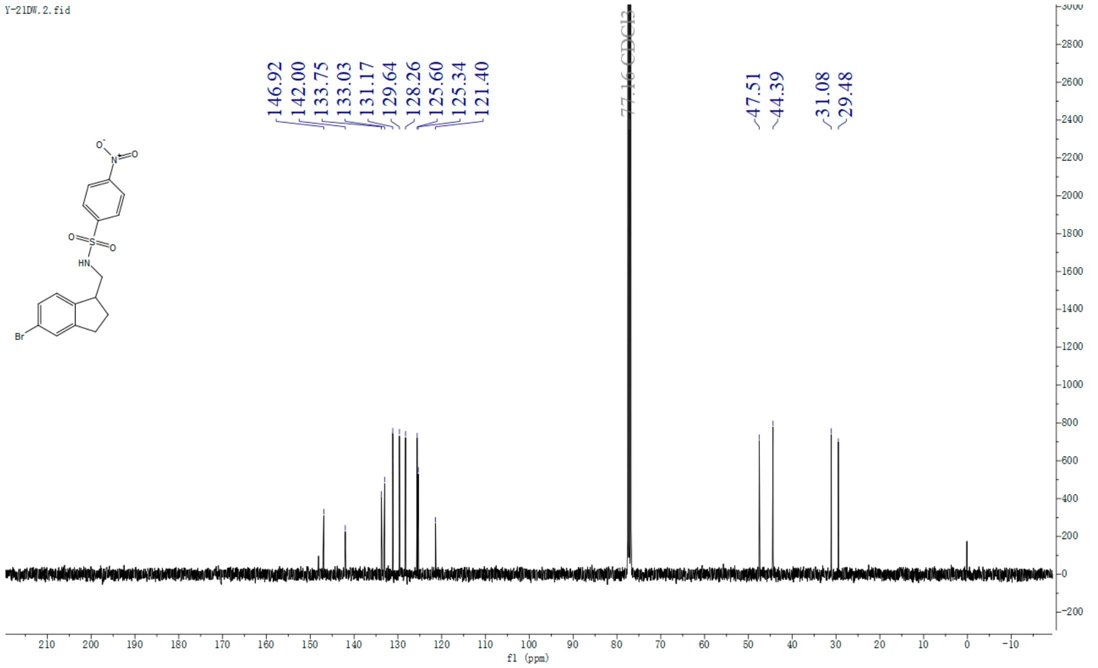

Figure S10. The ¹³C spectrum (101MHz, $CDCl_3$) of **rac-1r**.

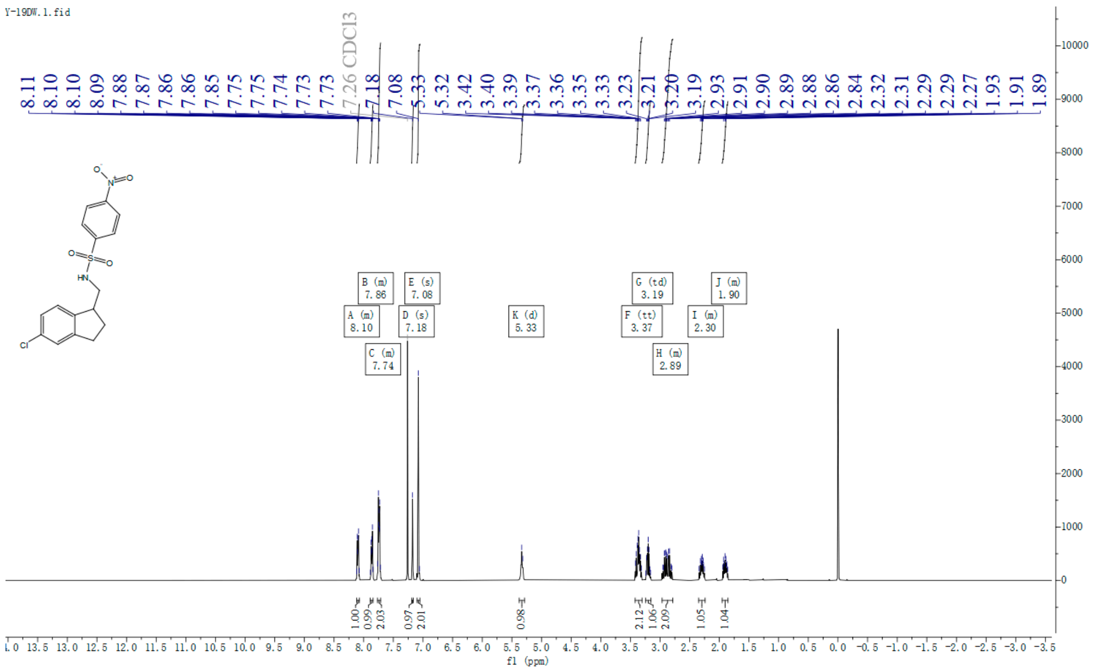

Figure S11. The ^{1}H spectrum (400MHz, $CDCl_3$) of **rac-1s**.

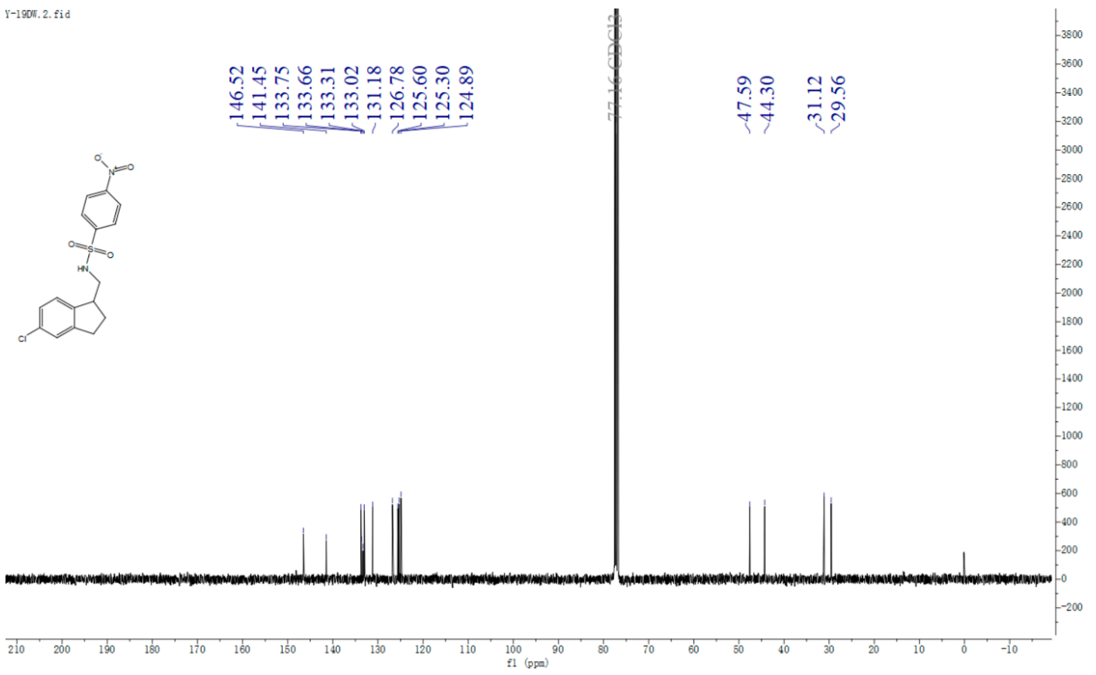

Figure S12. The ^{13}C spectrum (101MHz, $CDCl_3$) of **rac-1s**.

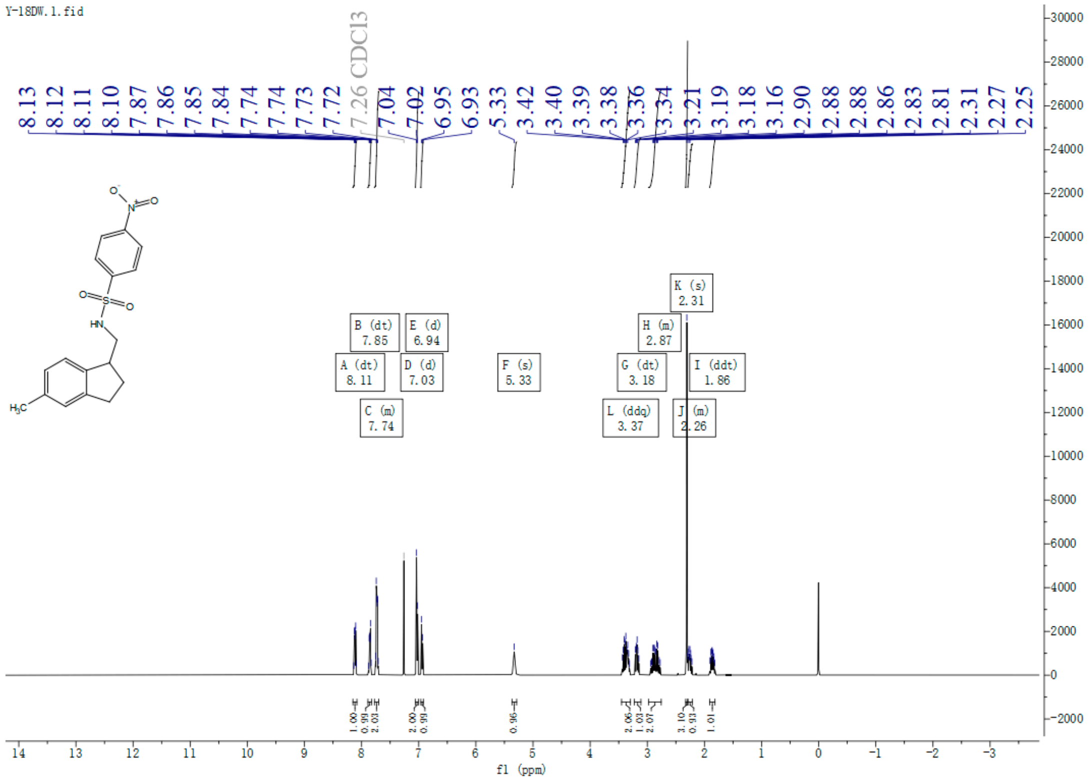

Figure S13. The ^{1}H spectrum (400MHz, $CDCl_3$) of **rac-1t**.

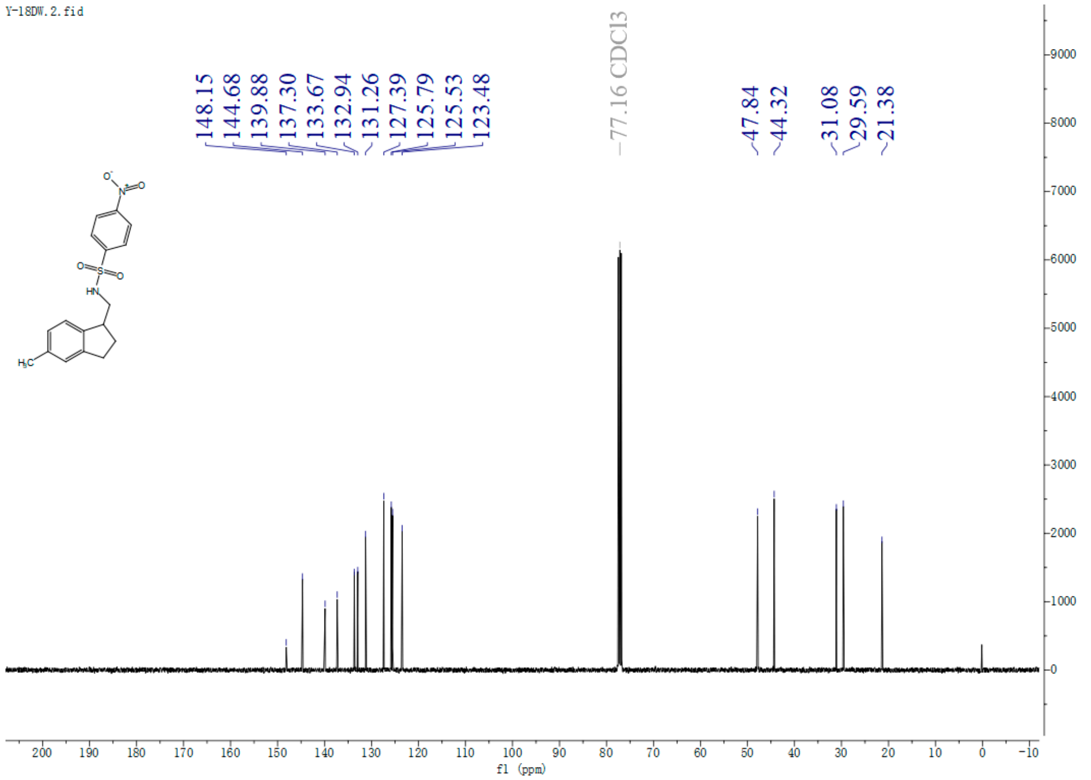

Figure S14. The ^{13}C spectrum (101MHz, $CDCl_3$) of **rac-1t**.

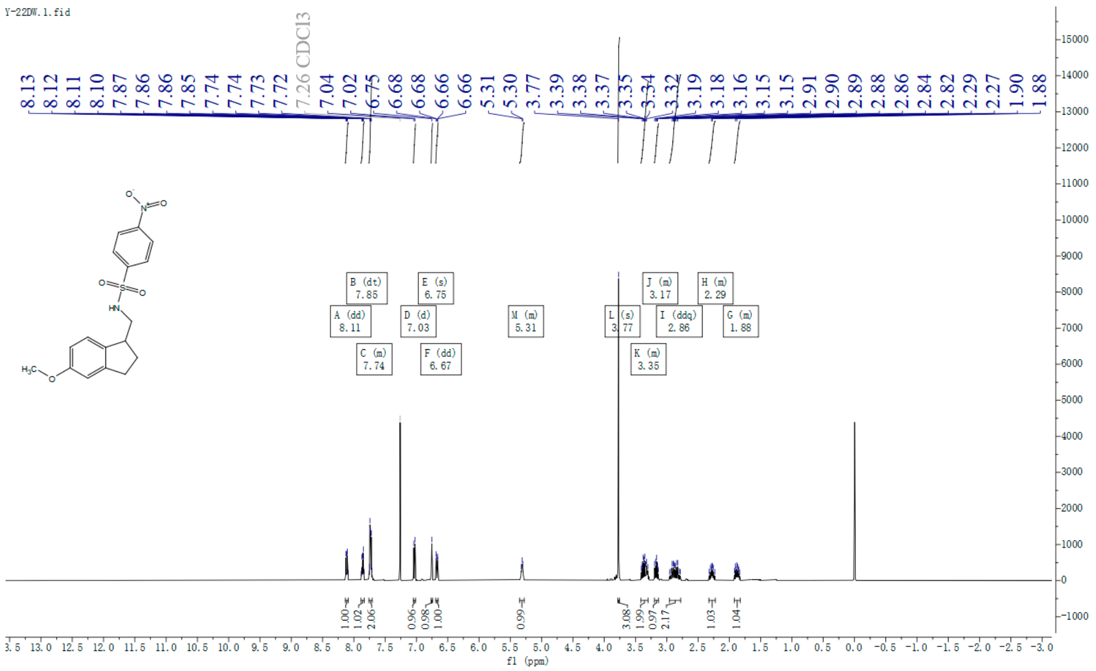

Figure S15. The ¹H spectrum (400MHz, $CDCl_3$) of **rac-1u**.

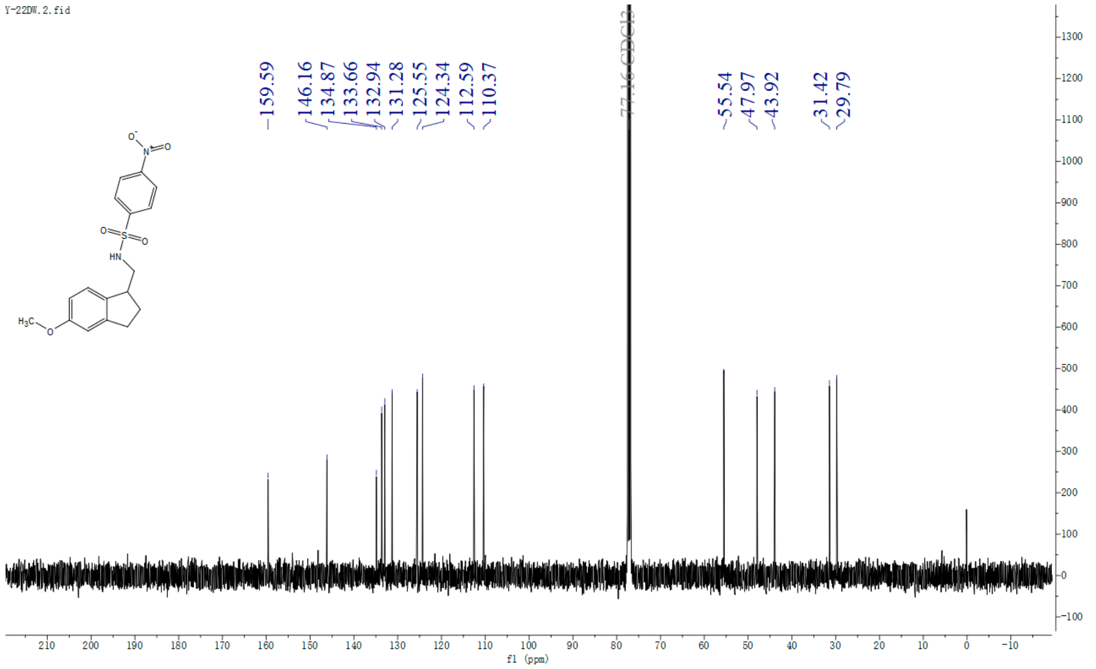

Figure S16. The ¹³C spectrum (101MHz, $CDCl_3$) of **rac-1u**.

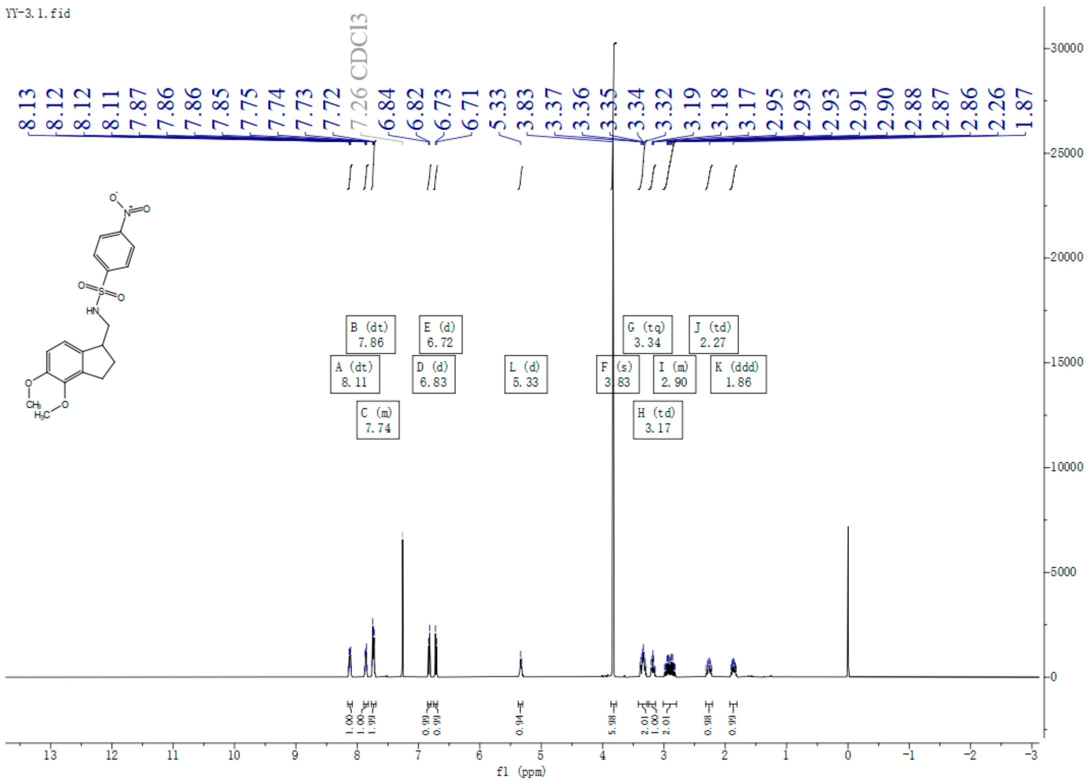

Figure S17. The ^{1}H spectrum (400MHz, $CDCl_3$) of **rac-1v**.

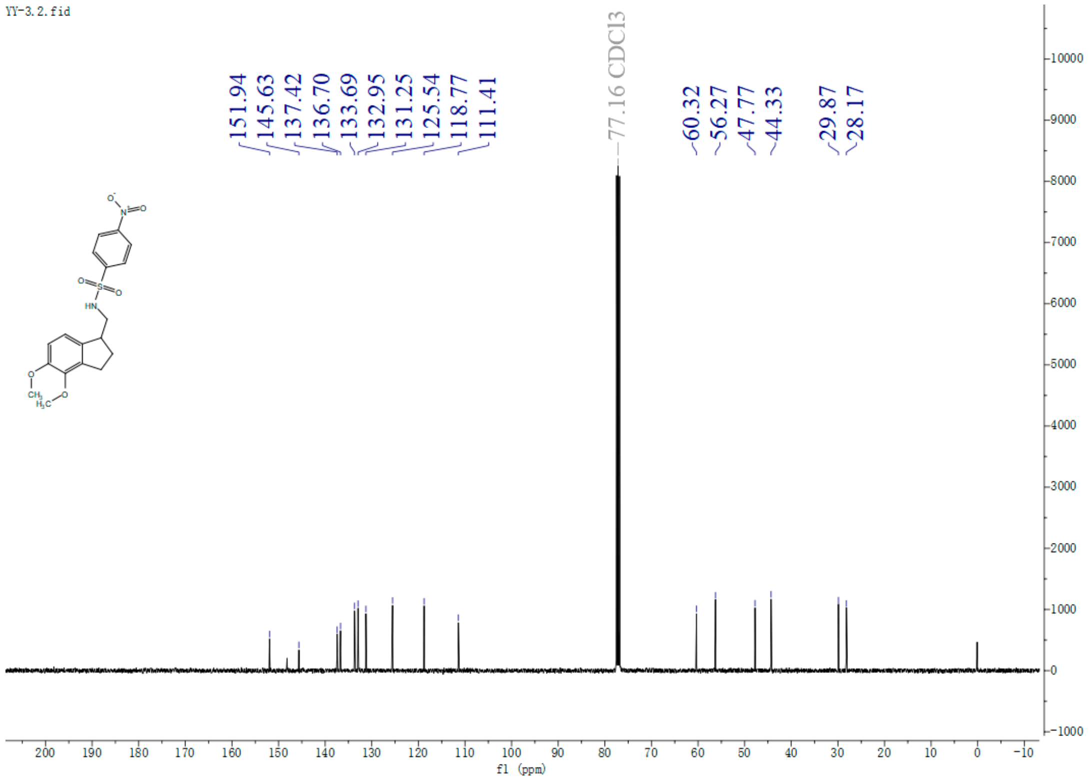

Figure S18. The ^{13}C spectrum (101MHz, $CDCl_3$) of **rac-1v**.

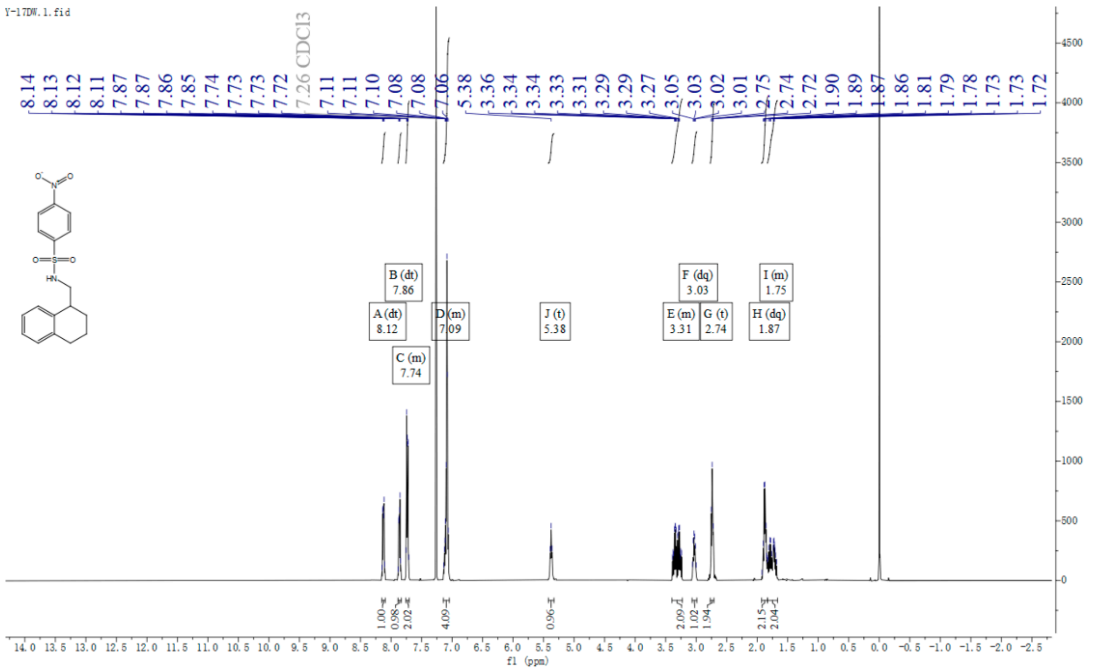

Figure S19. The ^{1}H spectrum (400MHz, $CDCl_3$) of **rac-1w**.

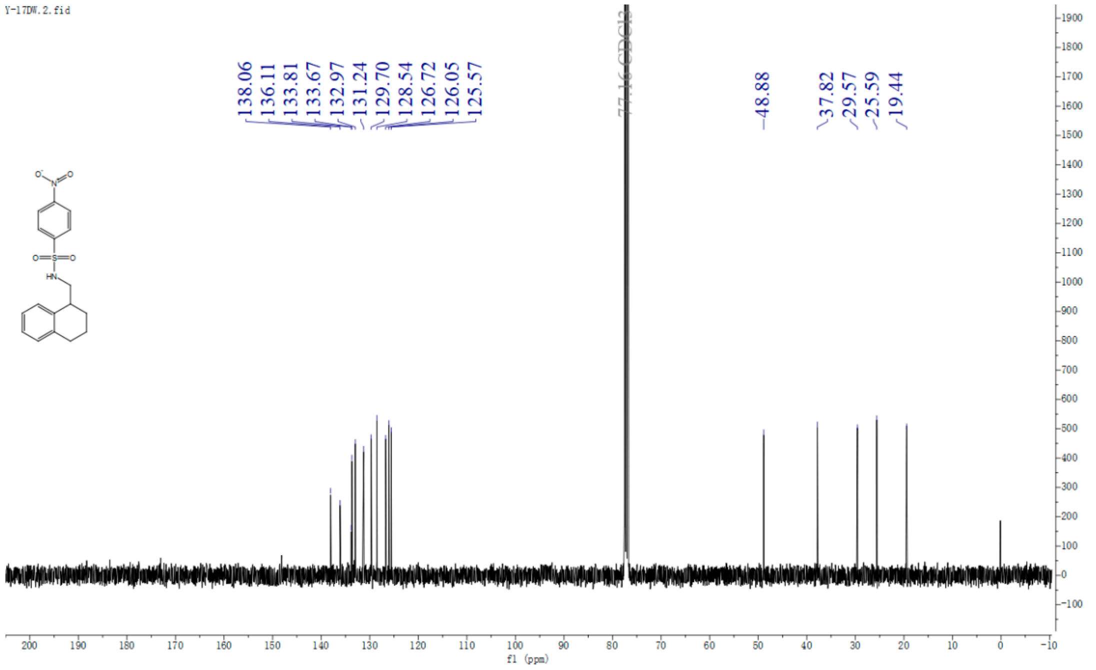

Figure S20. The ^{13}C spectrum (101MHz, $CDCl_3$) of **rac-1w**.

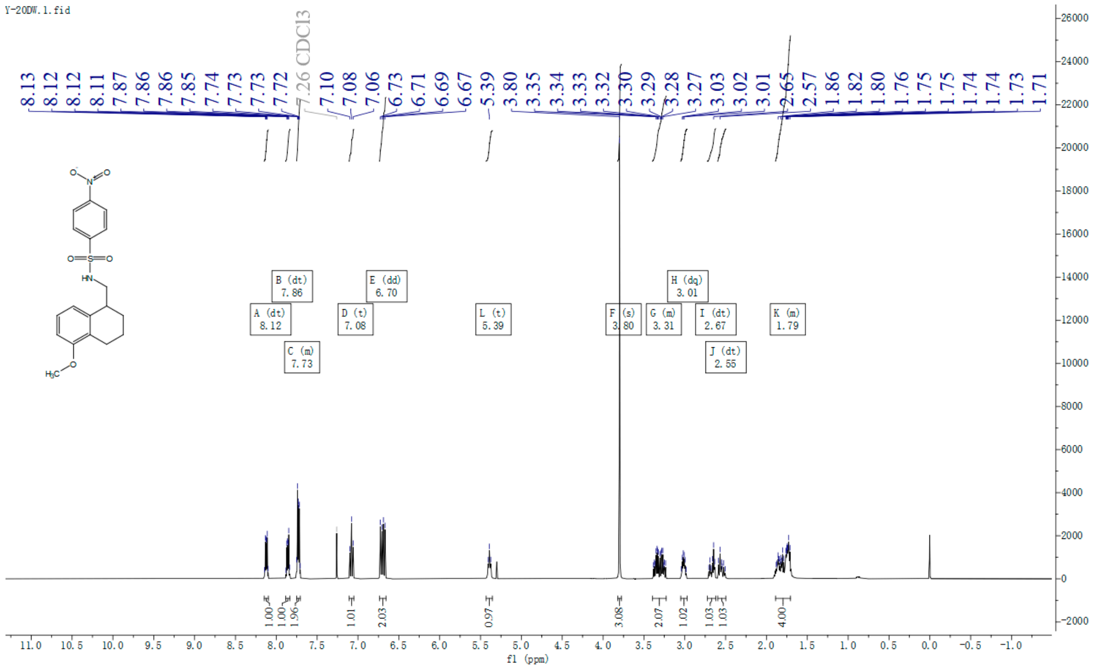

Figure S21. The ^{1}H spectrum (400MHz, $CDCl_3$) of **rac-1x**.

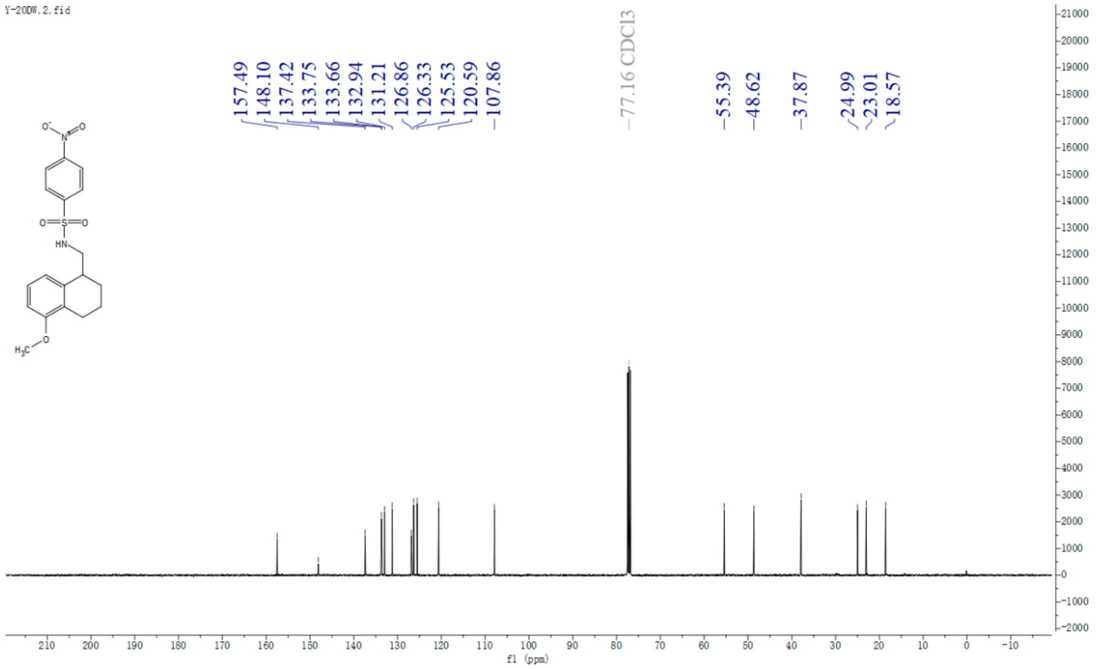

Figure S22. The ^{13}C spectrum (101MHz, $CDCl_3$) of **rac-1x**.

2 The ^{1}H spectrum and ^{13}C spectrum of **2a-x** and **3**.

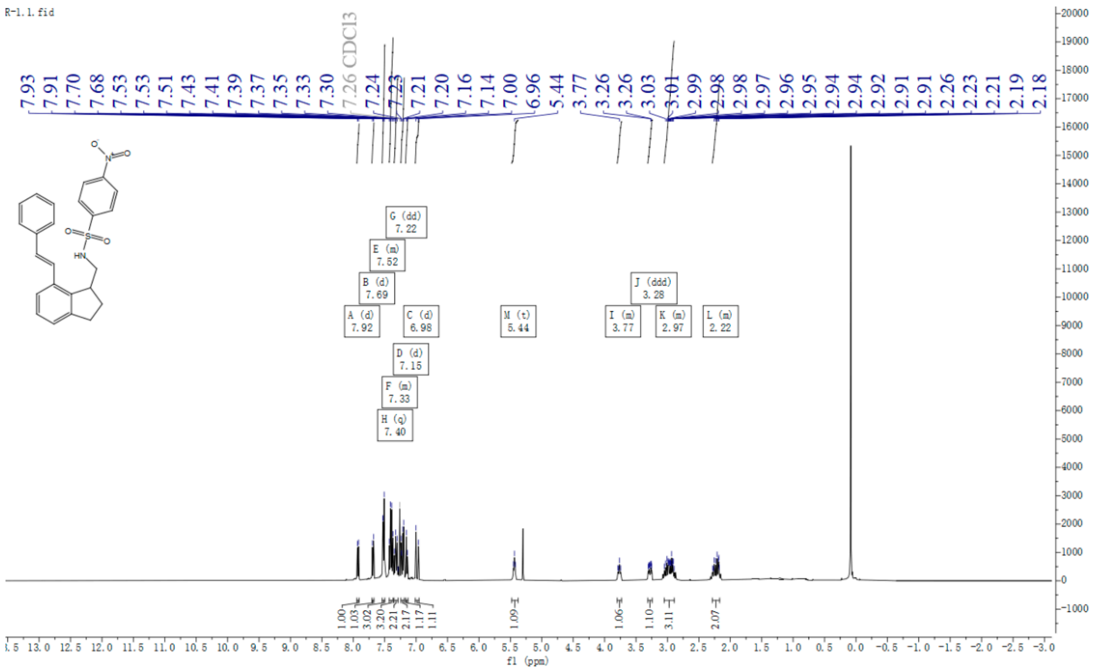

Figure S23. The ^{1}H spectrum (400MHz, $CDCl_3$) of **2a**.

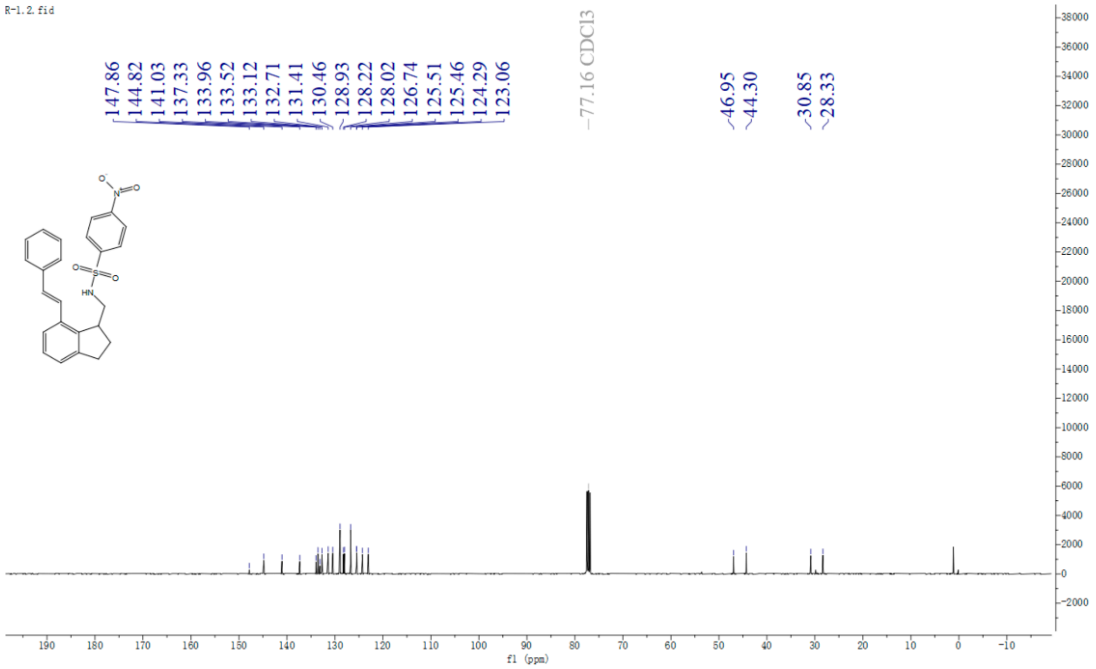

Figure S24. The ^{13}C spectrum (101MHz, $CDCl_3$) of **2a**.

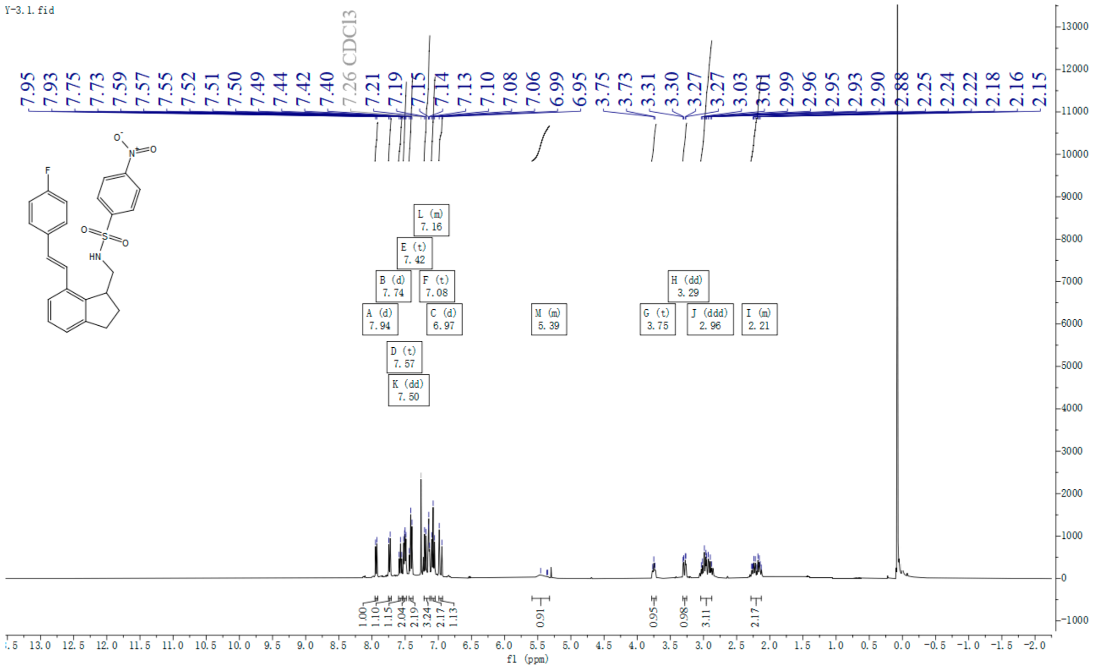

Figure S25. The ^{1}H spectrum (400MHz, $CDCl_3$) of **2b**.

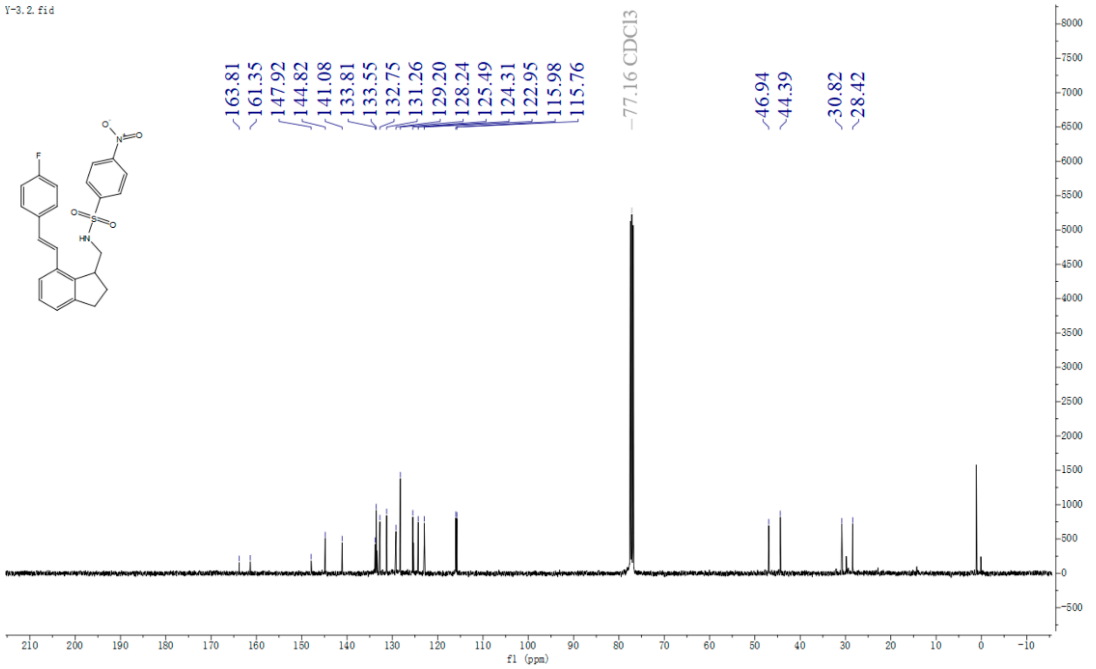

Figure S26. The ^{13}C spectrum (101MHz, $CDCl_3$) of **2b**.

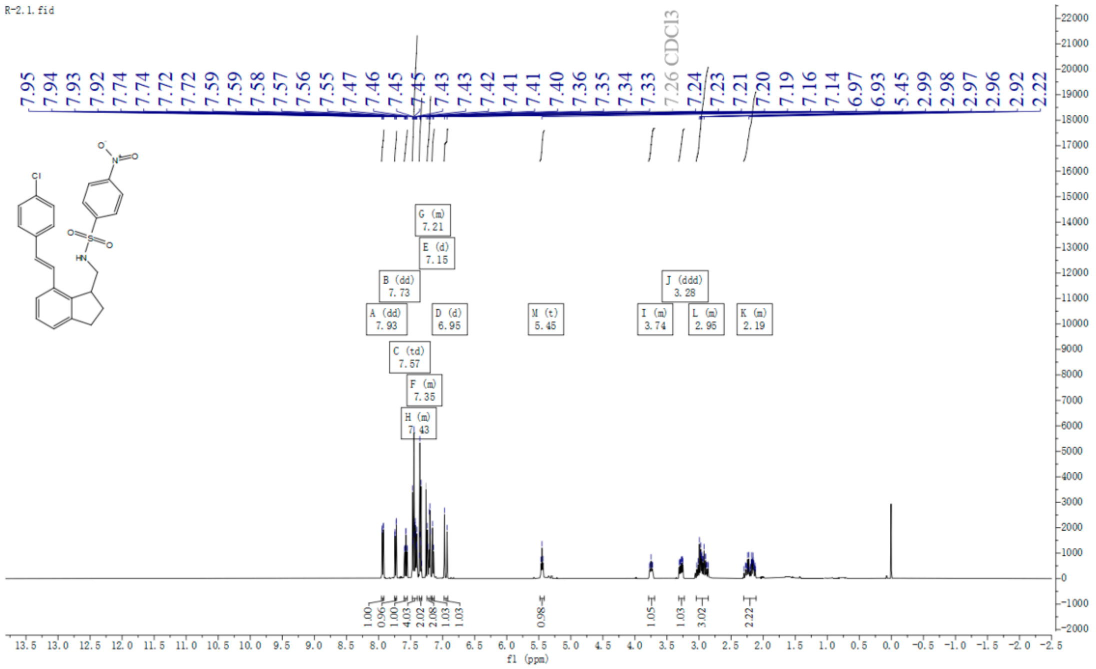

Figure S27. The ^{1}H spectrum (400MHz, $CDCl_3$) of **2c**.

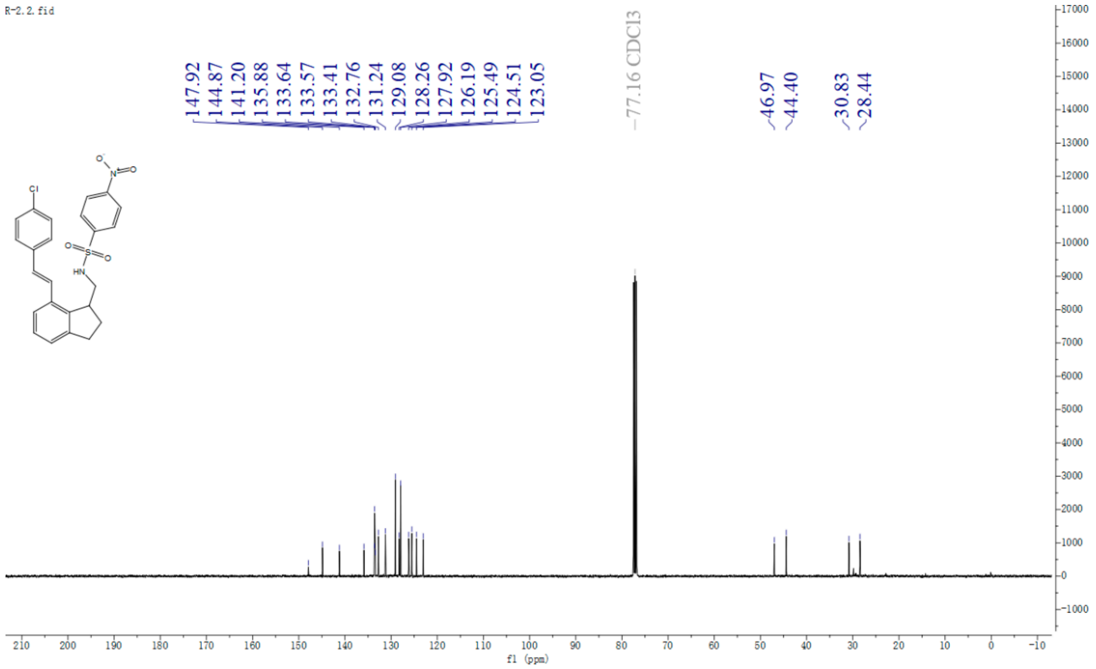

Figure S28. The ^{13}C spectrum (101MHz, $CDCl_3$) of **2c**.

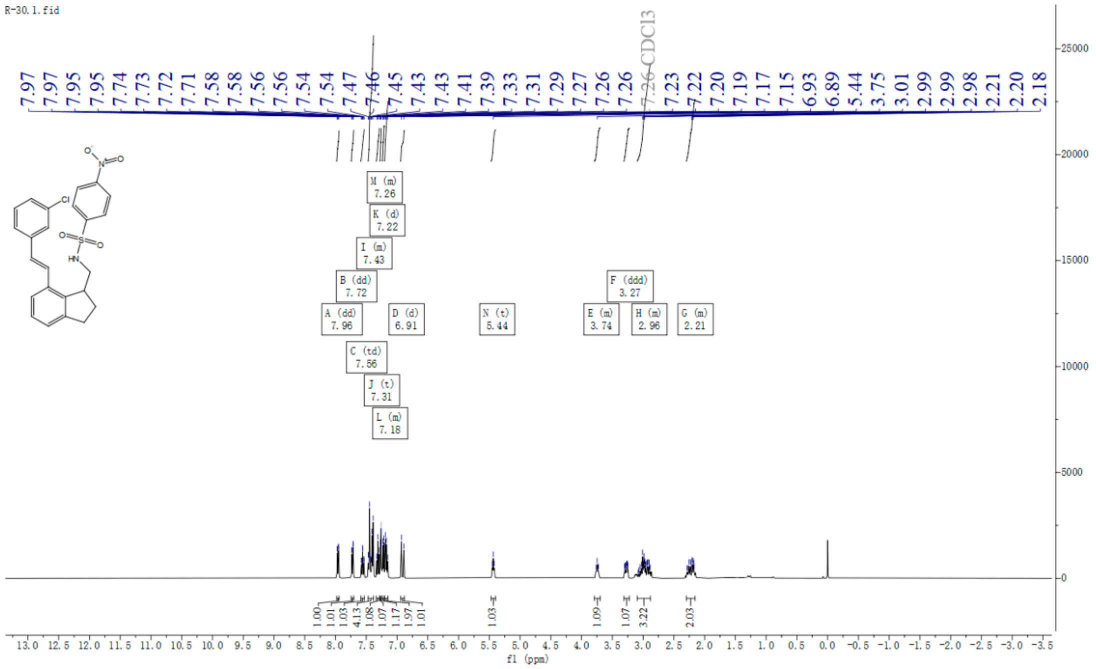

Figure S29. The ^{1}H spectrum (400MHz, $CDCl_3$) of **2d**.

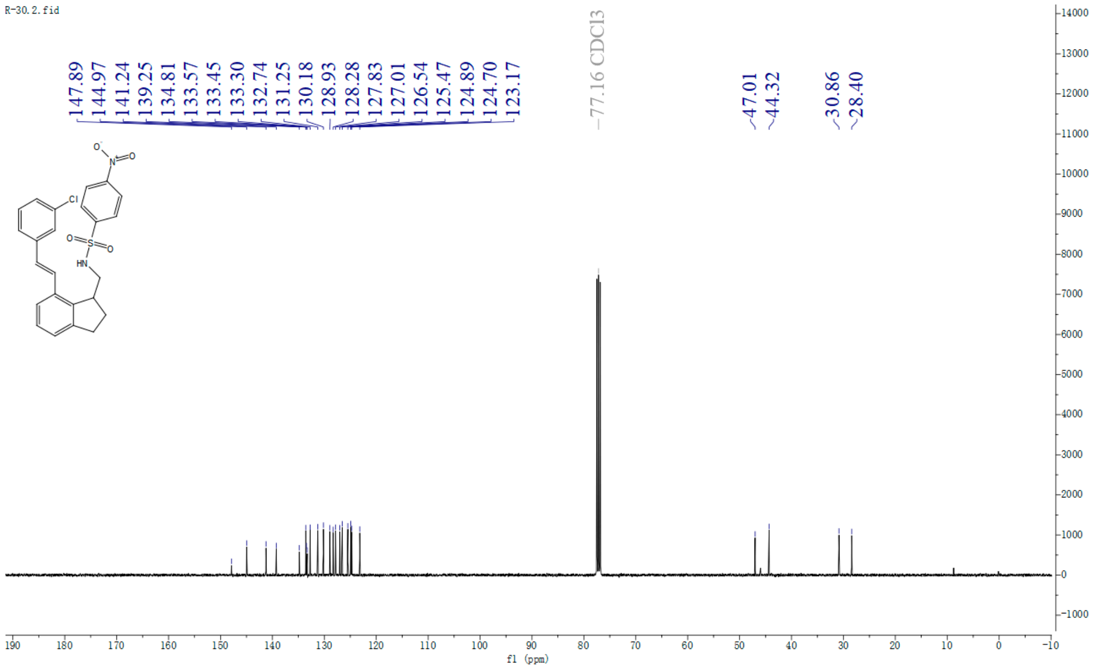

Figure S30. The ^{13}C spectrum (101MHz, $CDCl_3$) of **2d**.

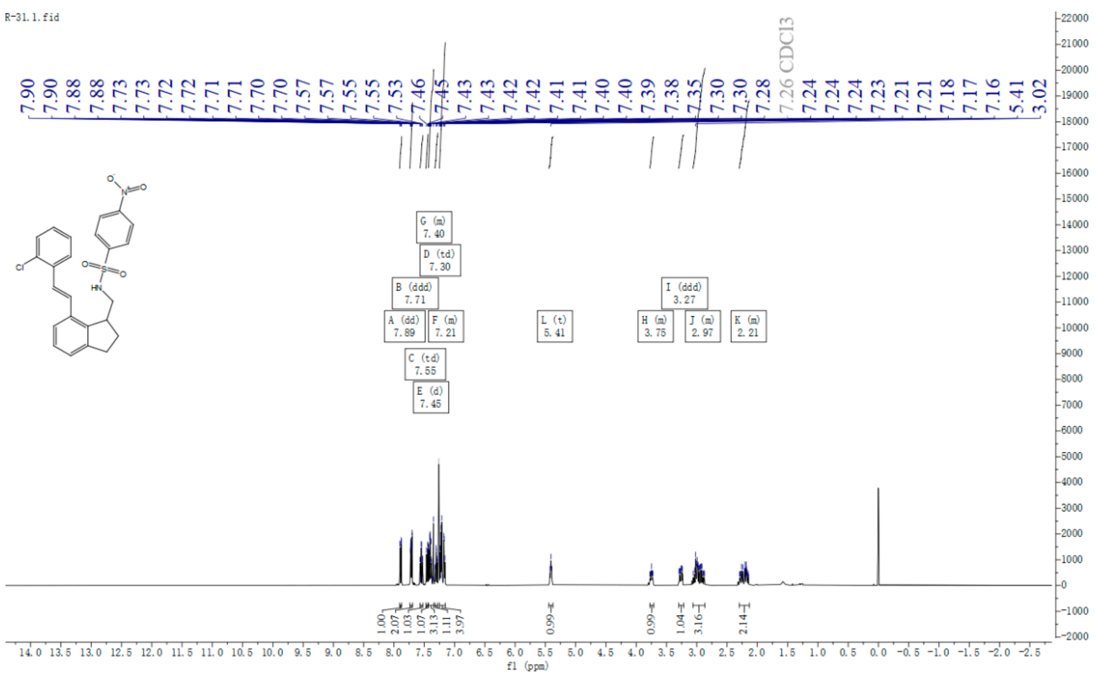

Figure S31. The ^{1}H spectrum (400MHz, $CDCl_3$) of **2e**.

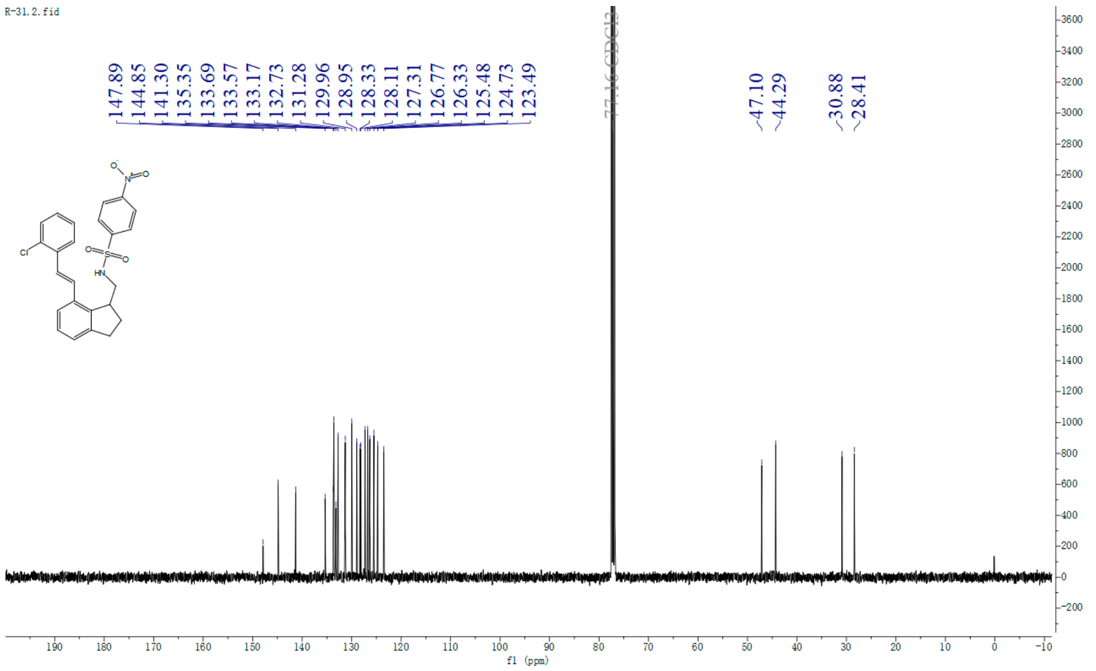

Figure S32. The ^{13}C spectrum (101MHz, $CDCl_3$) of **2e**.

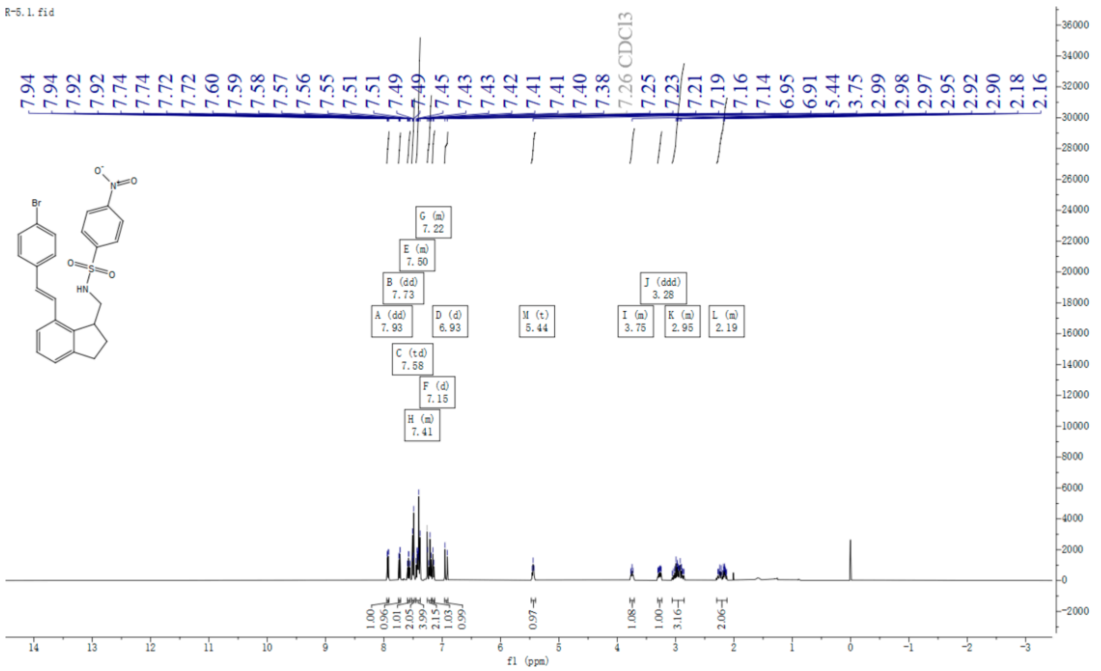

Figure S33. The ¹H spectrum (400MHz, CDCl₃) of **2f**.

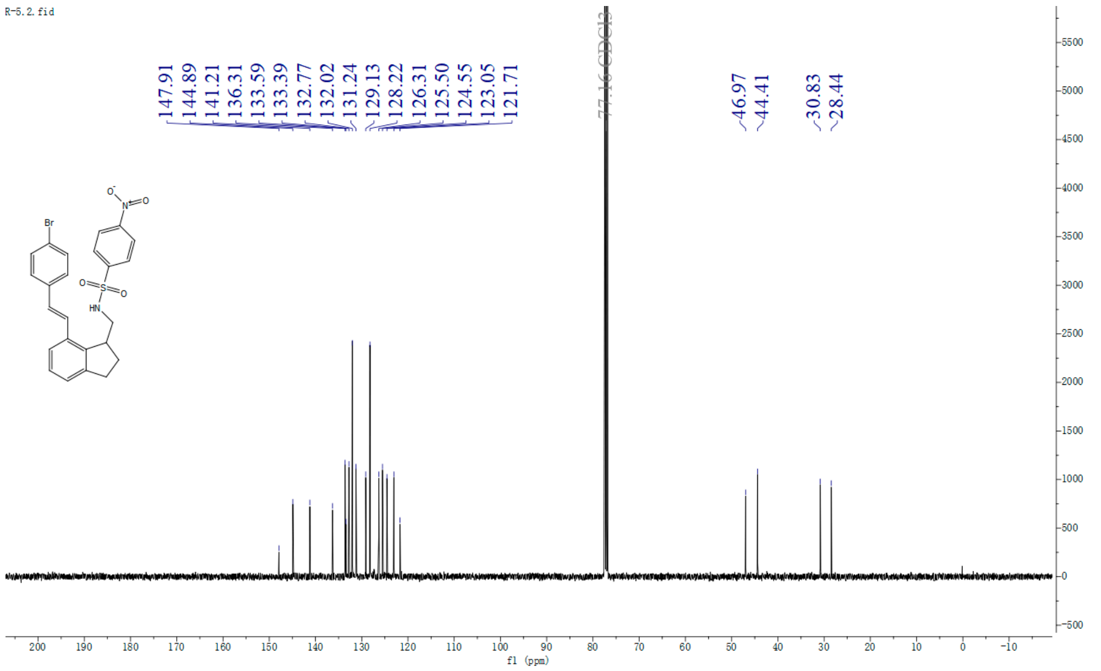

Figure S34. The ¹³C spectrum (101MHz, CDCl₃) of **2f**.

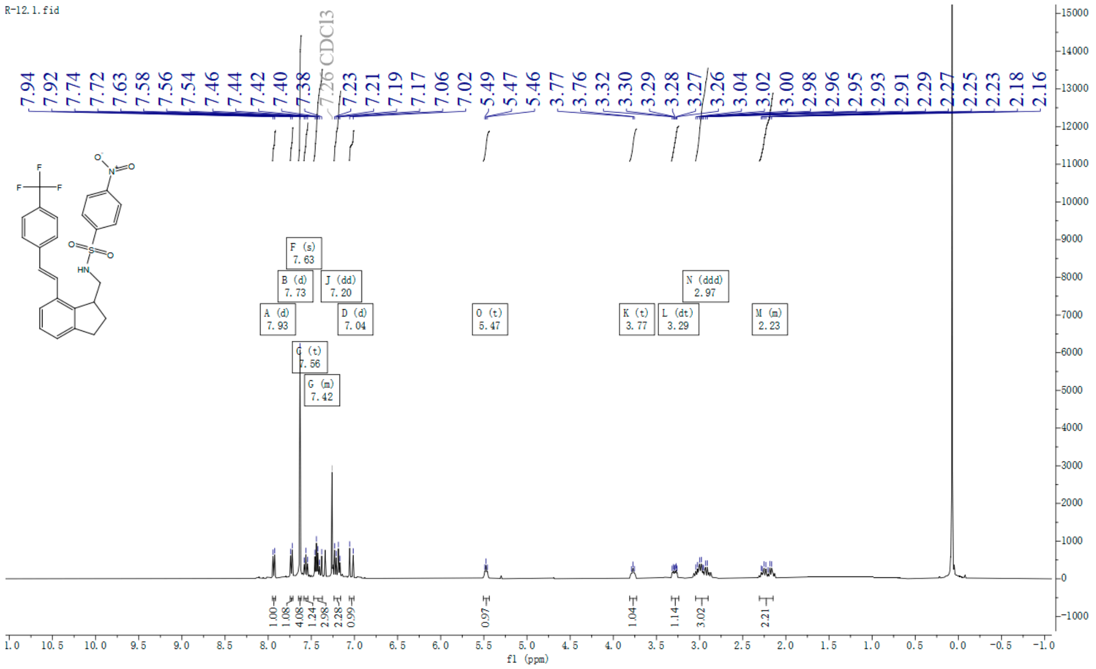

Figure S35. The ^{1}H spectrum (400MHz, $CDCl_3$) of **2g**.

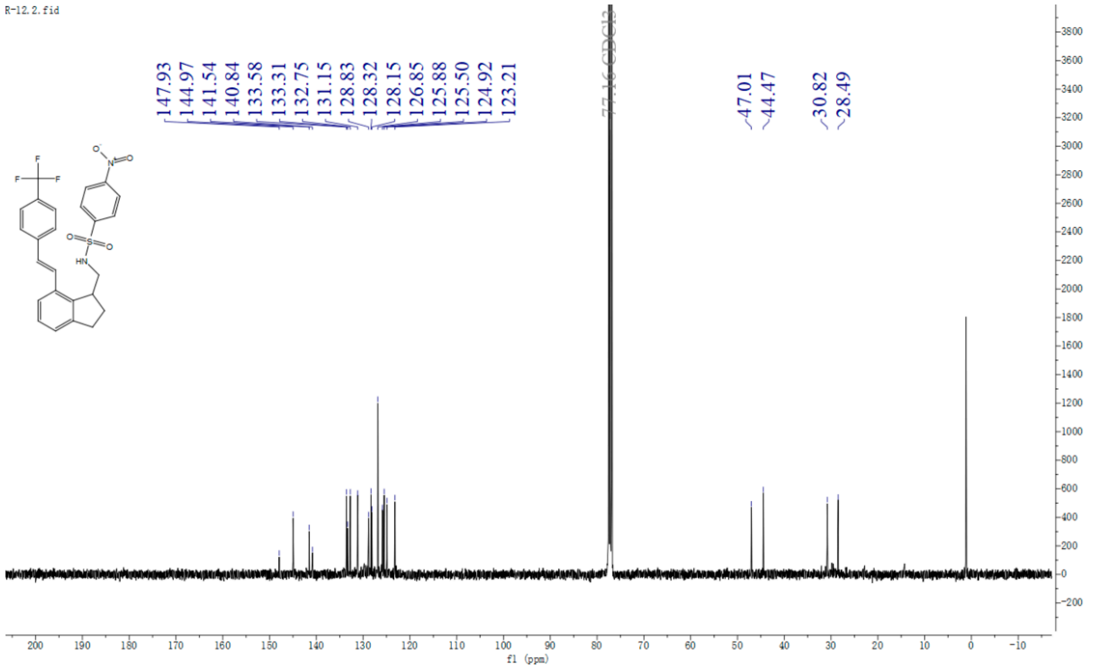

Figure S36. The ^{13}C spectrum (101MHz, $CDCl_3$) of **2g**.

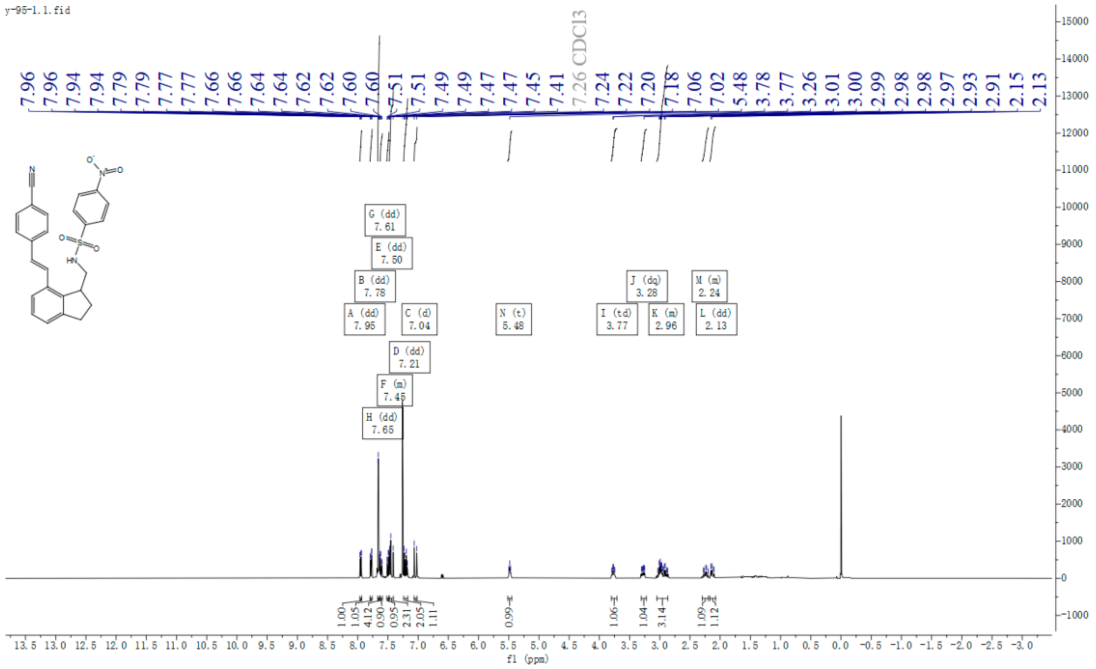

Figure S37. The ¹H spectrum (400MHz, $CDCl_3$) of **2h**.

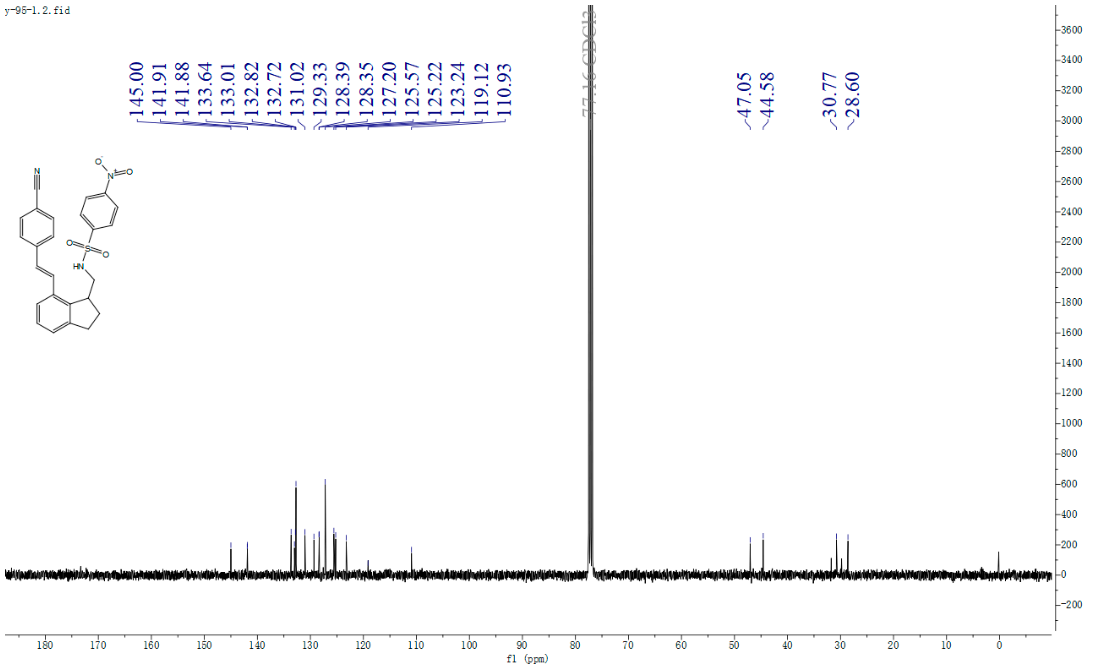

Figure S38. The ¹³C spectrum (101MHz, $CDCl_3$) of **2h**.

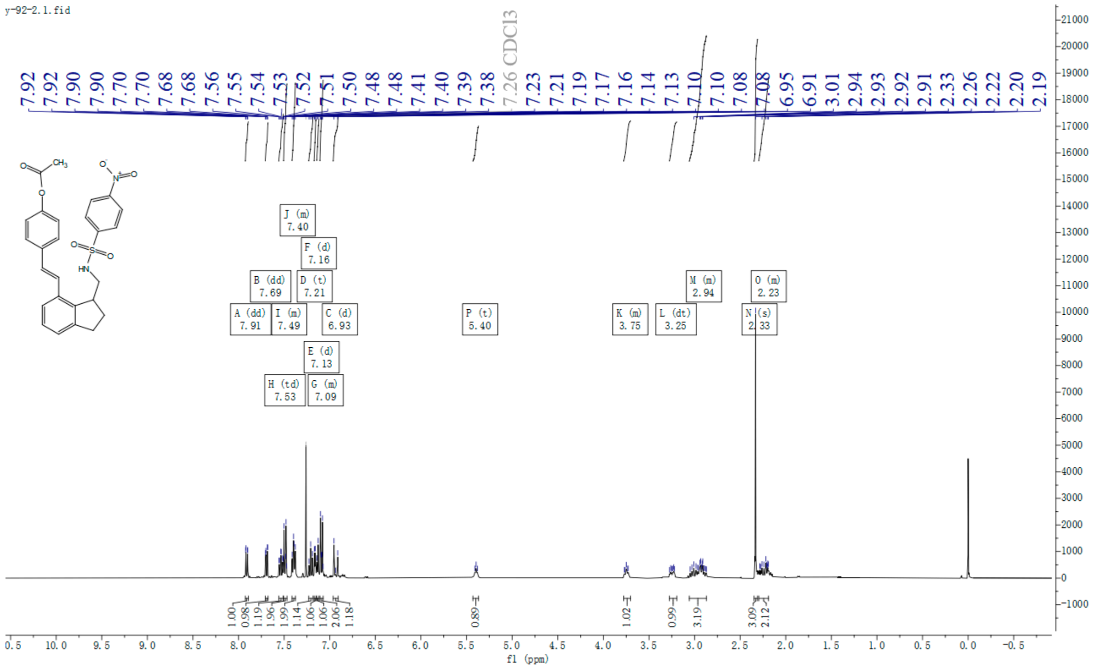

Figure S39. The ¹H spectrum (400MHz, $CDCl_3$) of **2i**.

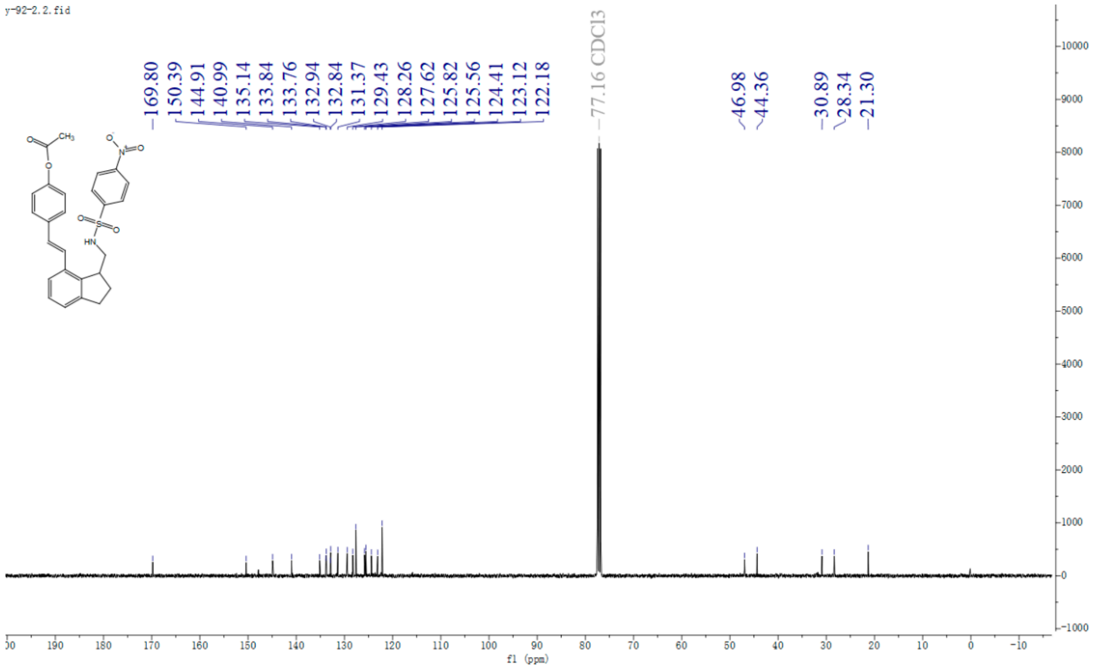

Figure S40. The ¹³C spectrum (101MHz, $CDCl_3$) of **2i**.

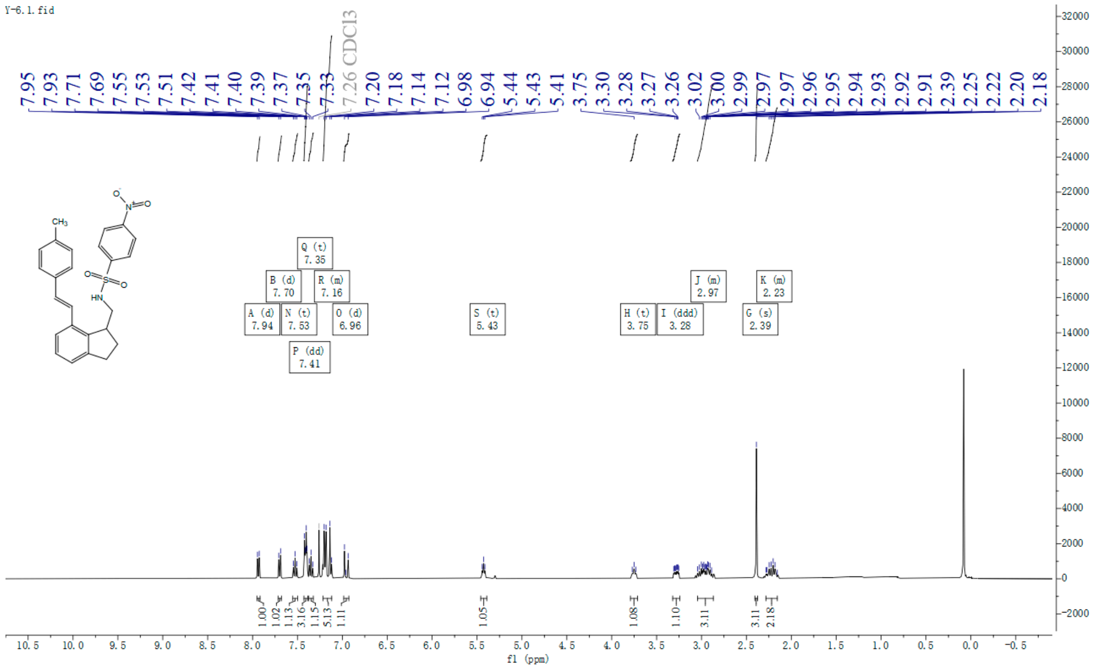

Figure S41. The ^{1}H spectrum (400MHz, $CDCl_3$) of **2j**.

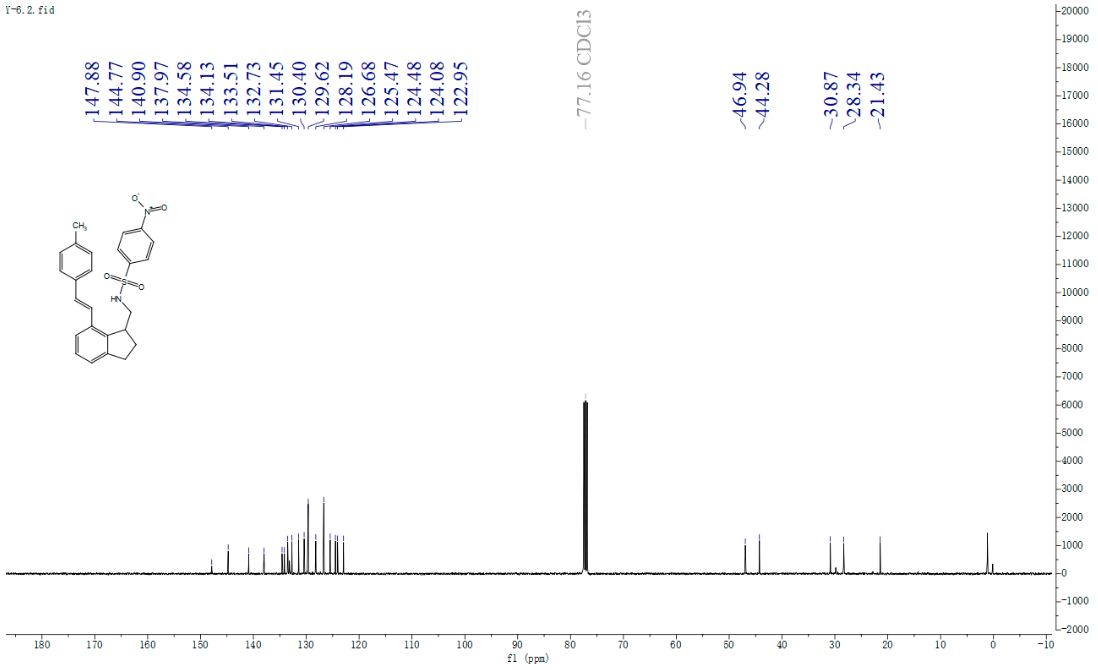

Figure S40. The ^{13}C spectrum (101MHz, $CDCl_3$) of **2j**.

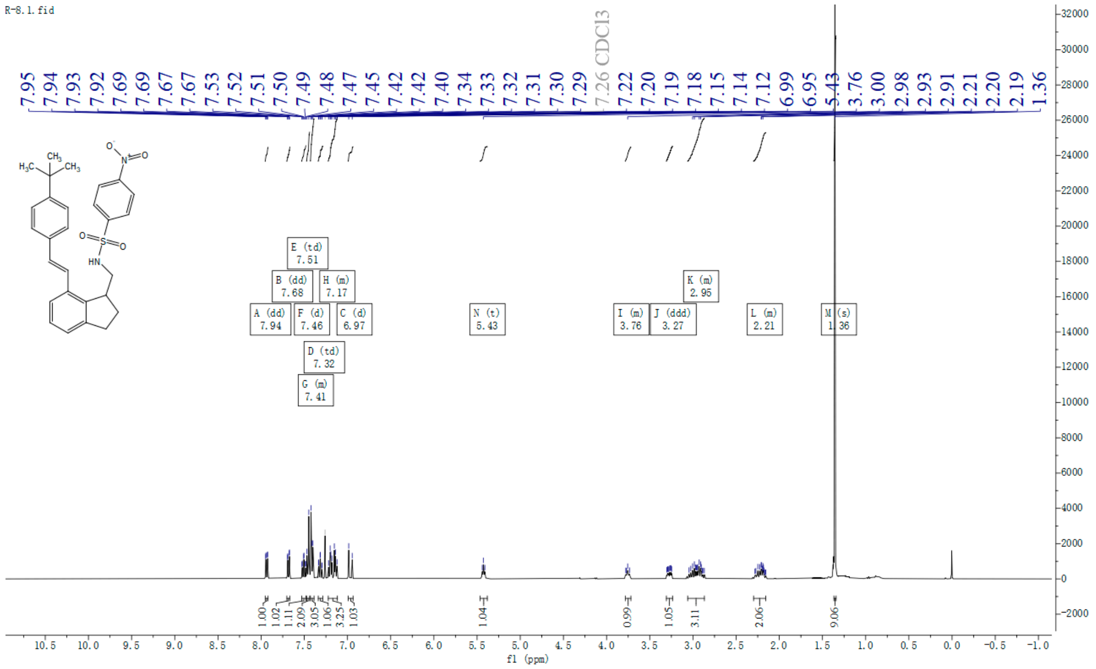

Figure S43. The ^{1}H spectrum (400MHz, $CDCl_3$) of **2k**.

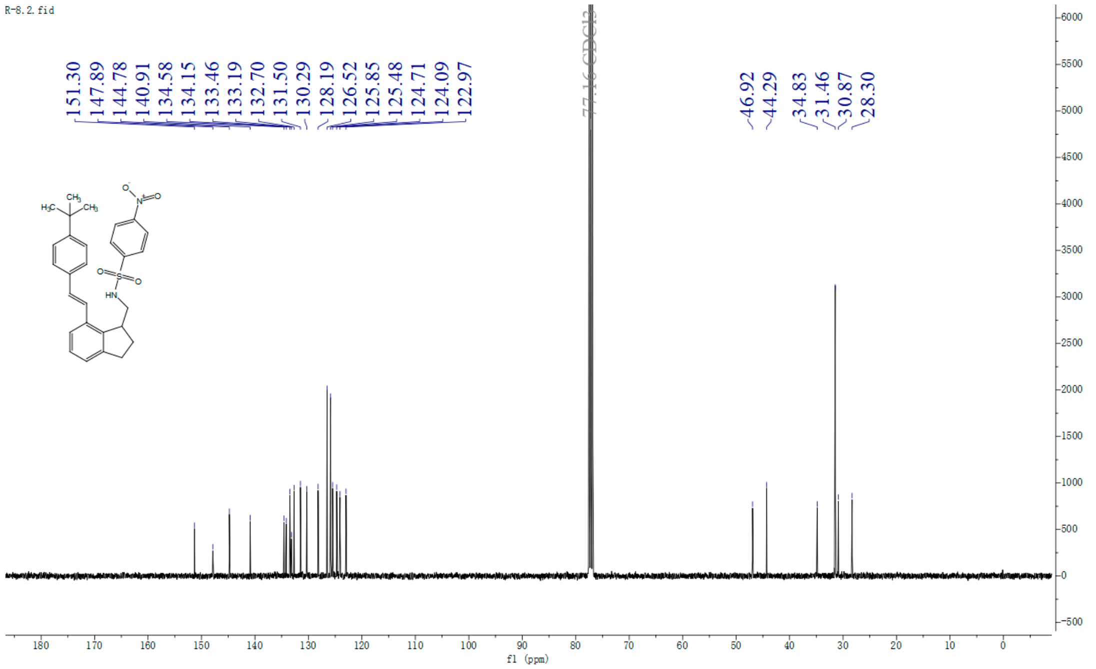

Figure S44. The ^{13}C spectrum (101MHz, $CDCl_3$) of **2k**.

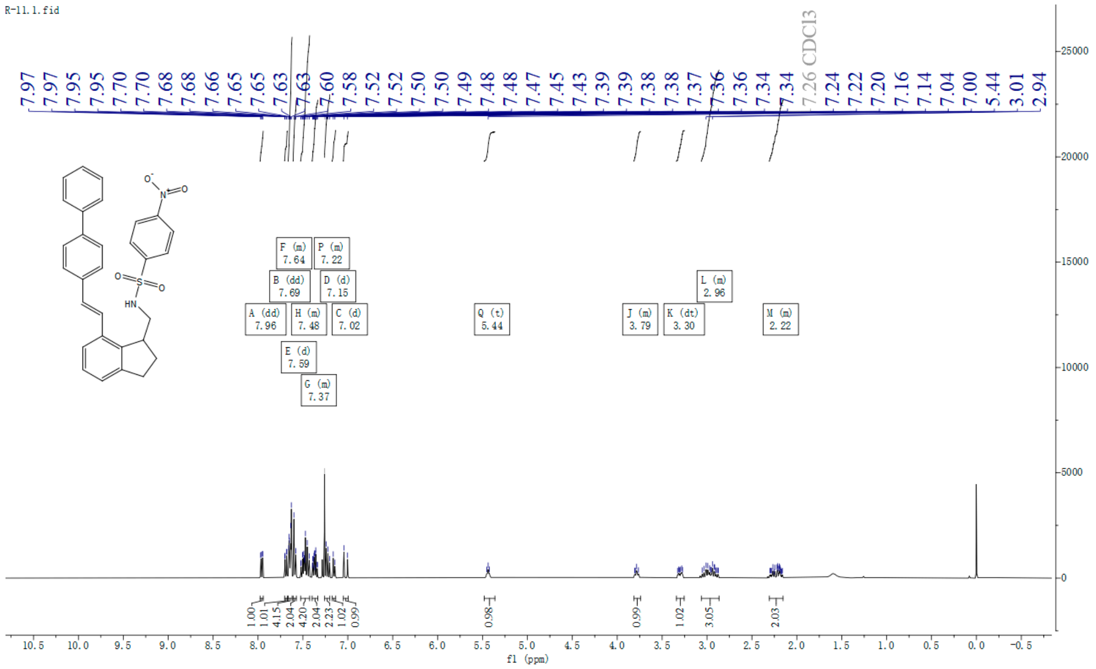

Figure S45. The ^{1}H spectrum (400MHz, $CDCl_3$) of **2l**.

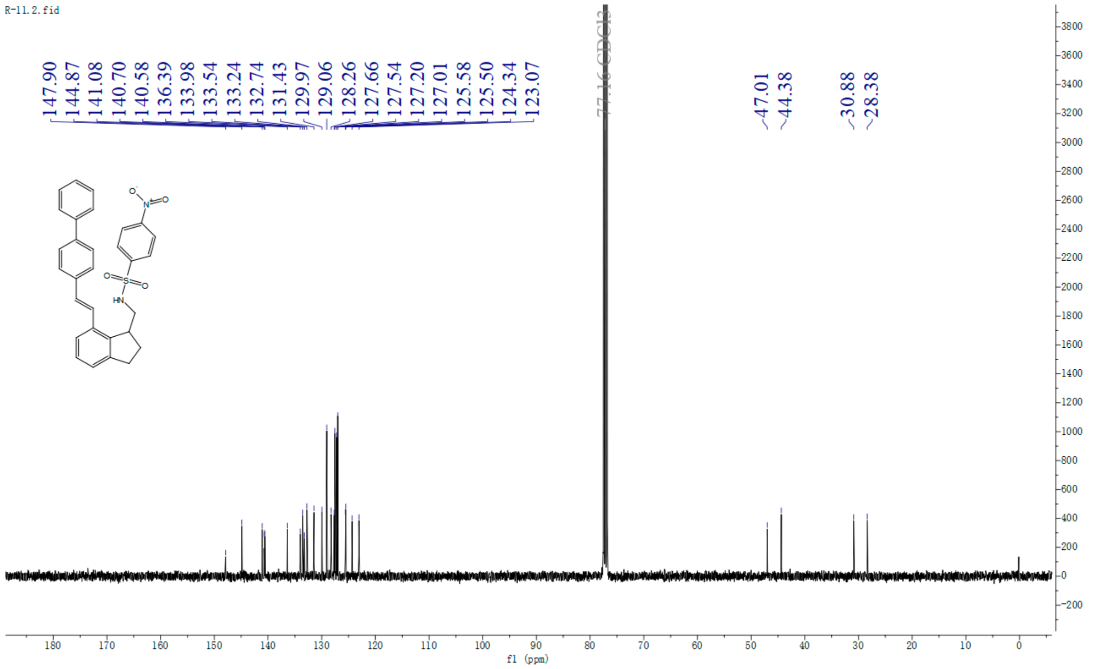

Figure S46. The ^{13}C spectrum (101MHz, $CDCl_3$) of **2l**.

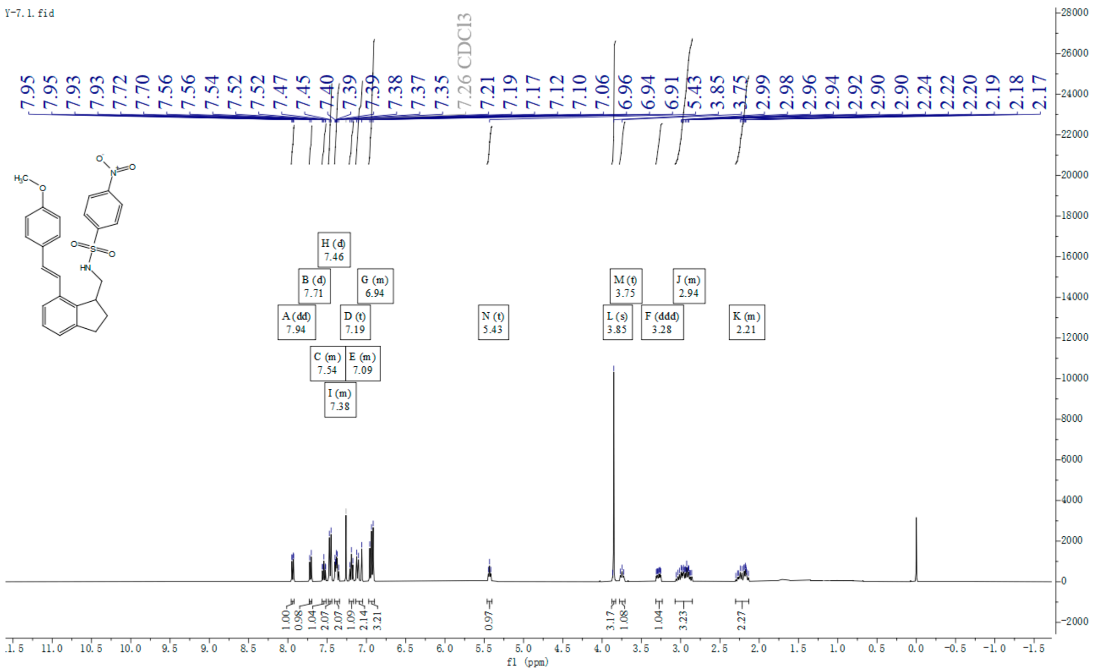

Figure S47. The ^{1}H spectrum (400MHz, $CDCl_3$) of **2m**.

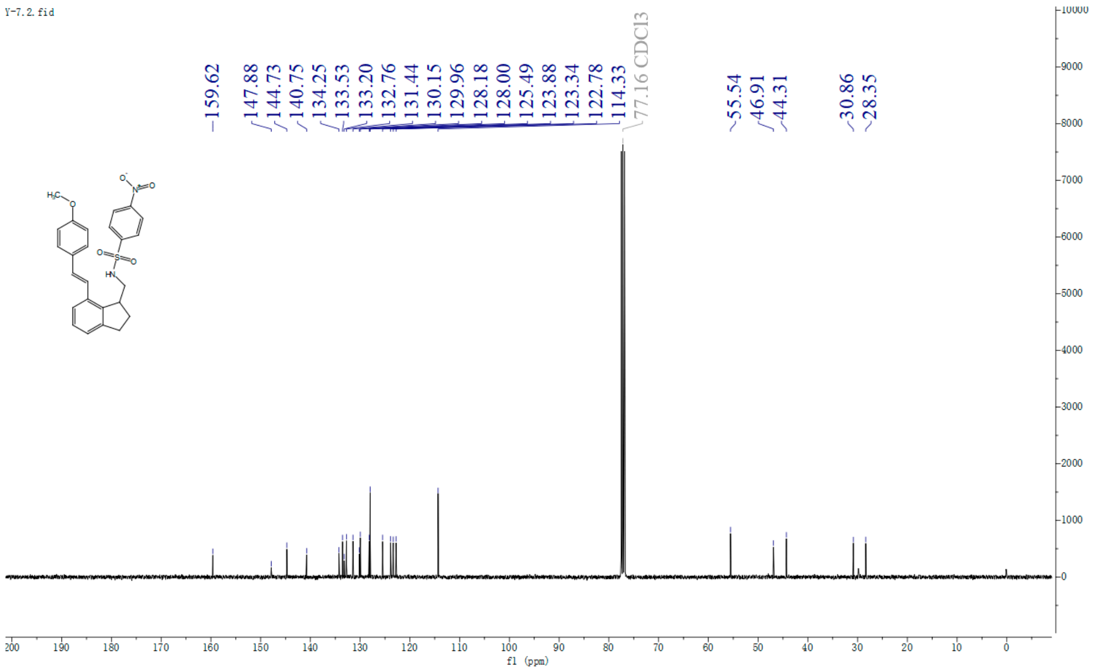

Figure S48. The ^{13}C spectrum (101MHz, $CDCl_3$) of **2m**.

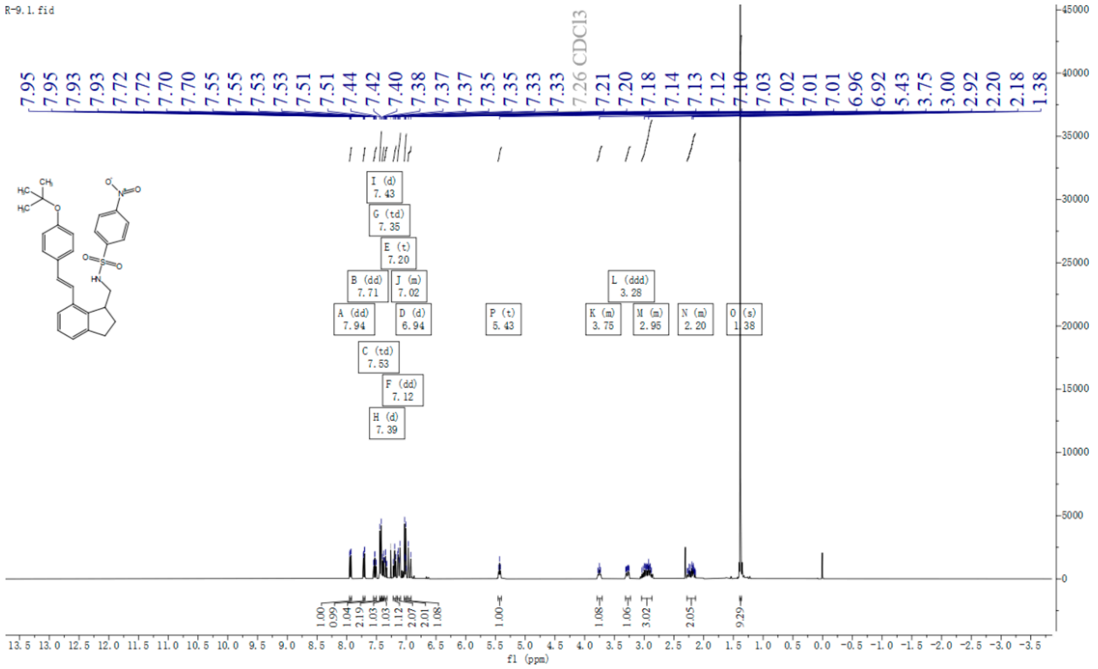

Figure S49. The ^{1}H spectrum (400MHz, $CDCl_3$) of **2n**.

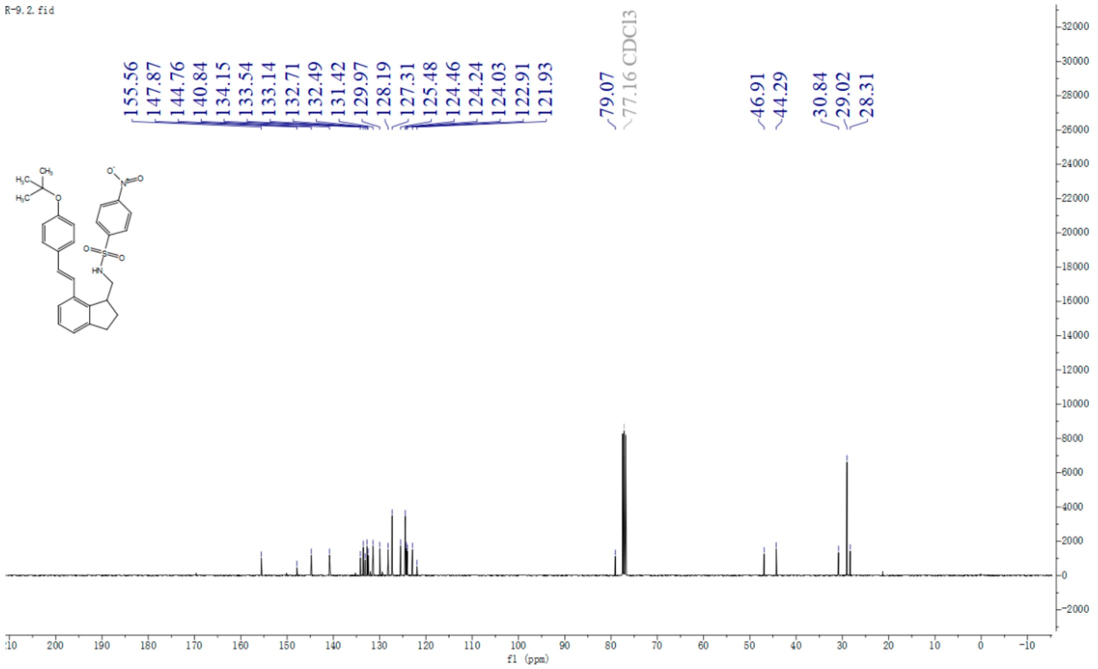

Figure S50. The ^{13}C spectrum (101MHz, $CDCl_3$) of **2n**.

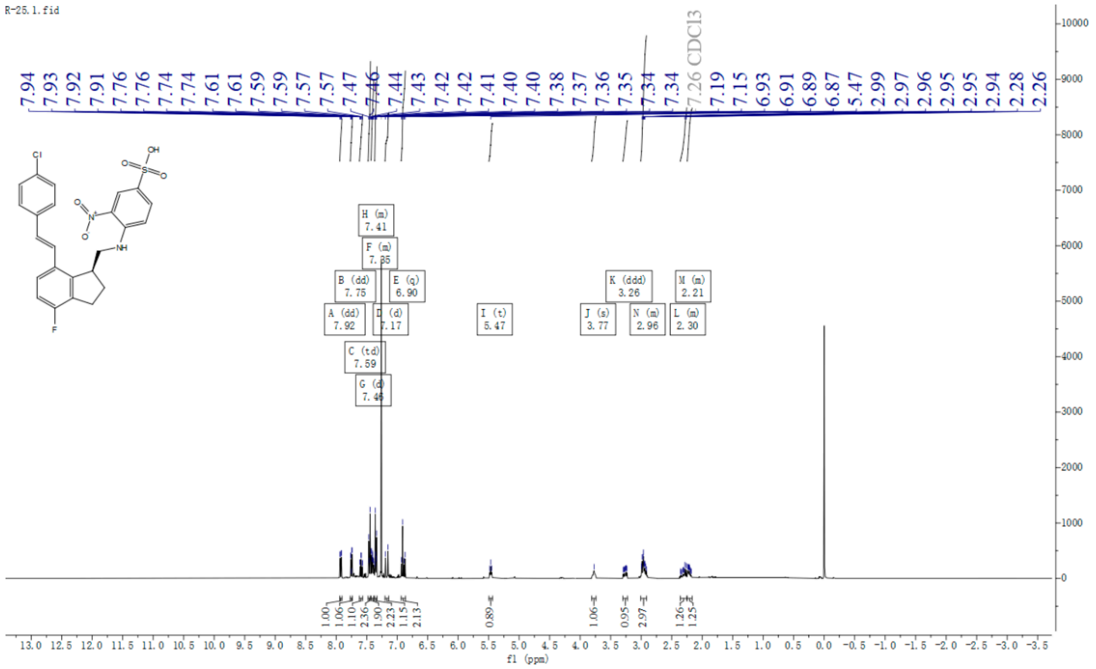

Figure S51. The ¹H spectrum (400MHz, $CDCl_3$) of **2o**.

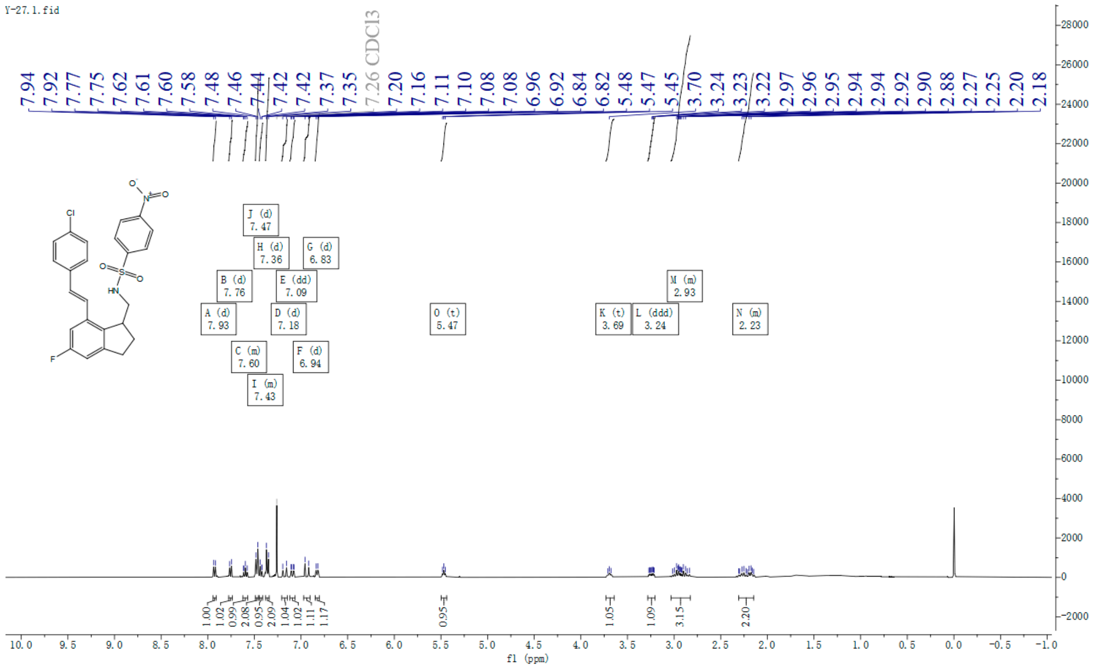

Figure S52. The ¹H spectrum (400MHz, $CDCl_3$) of **2p**.

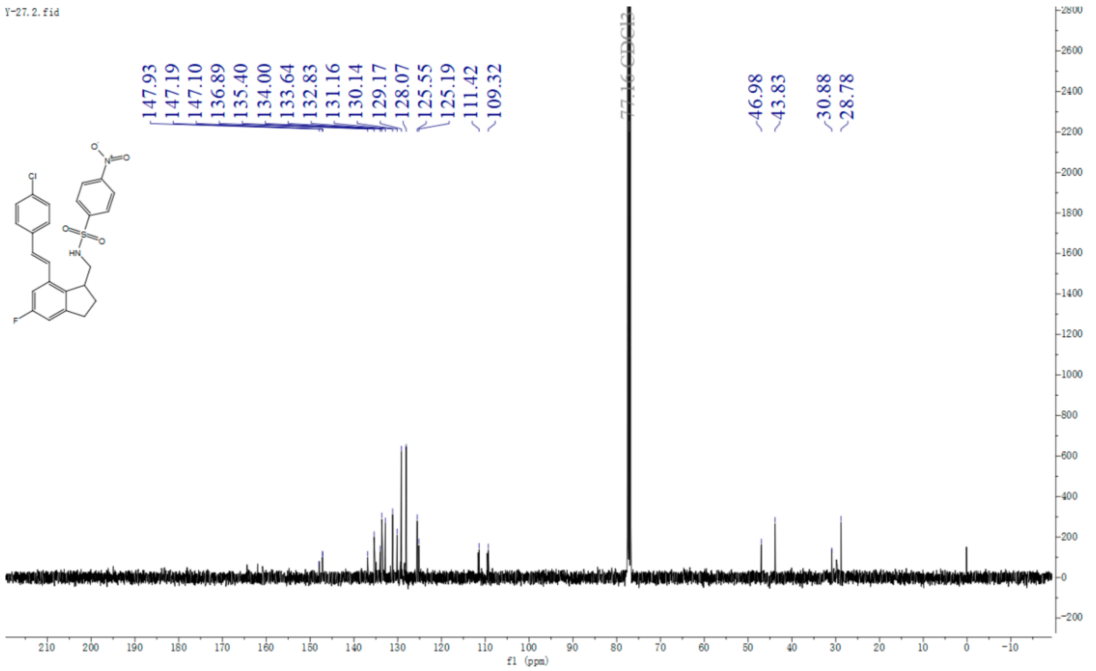

Figure S53. The ^{13}C spectrum (101MHz, $CDCl_3$) of **2p**.

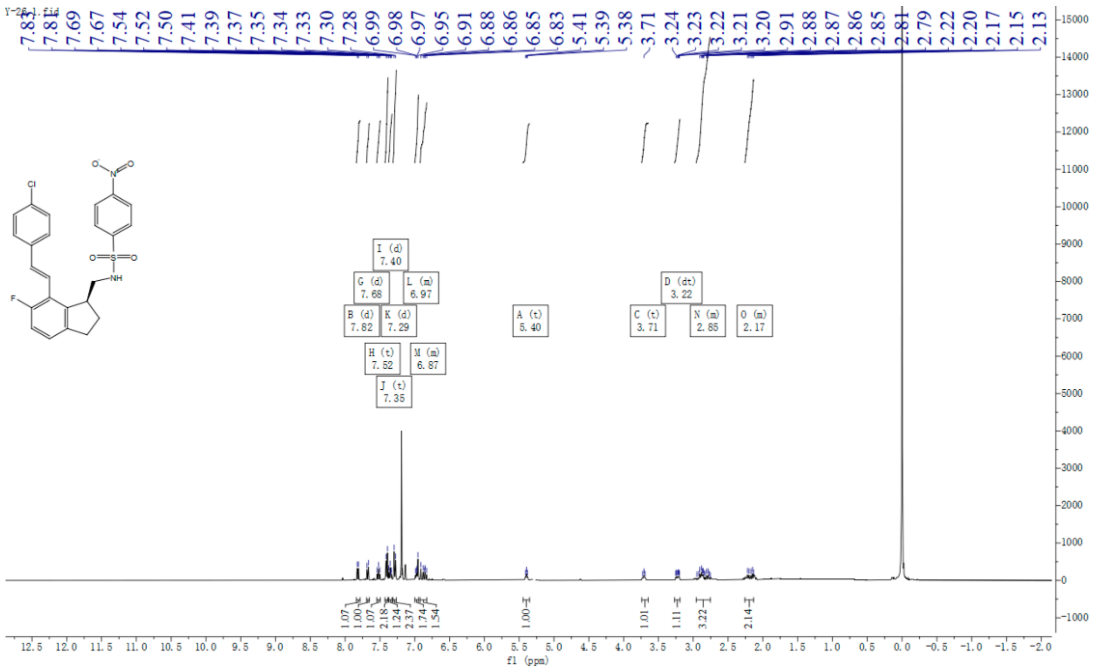

Figure S54. The ^{1}H spectrum (400MHz, $CDCl_3$) of **2q**.

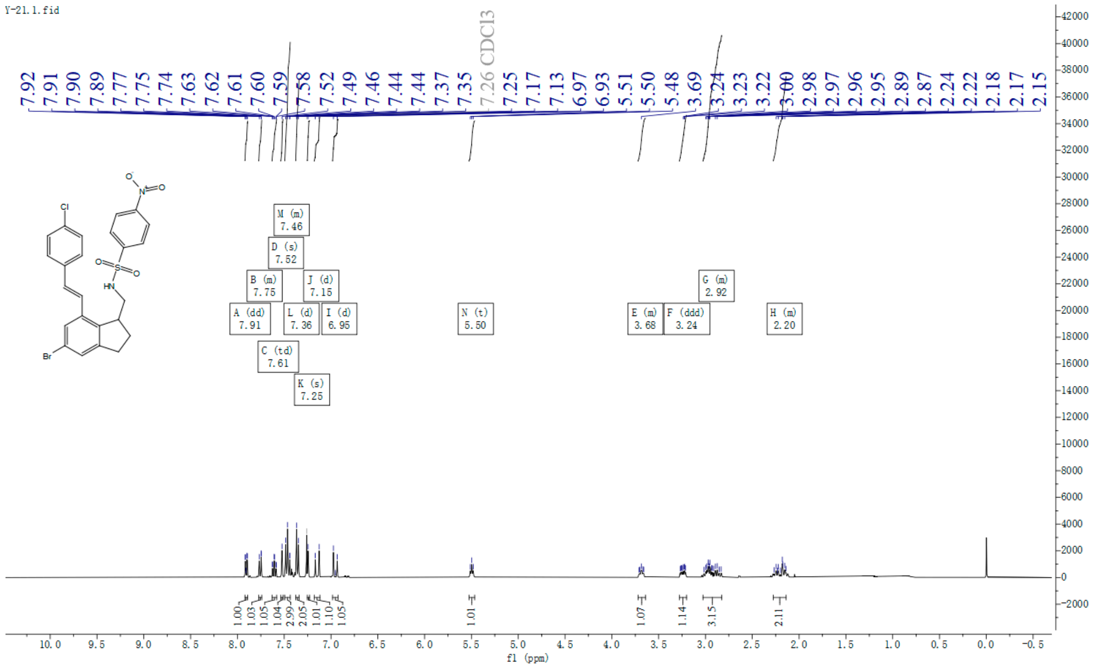

Figure S55. The ^1H spectrum (400MHz, $CDCl_3$) of **2r**.

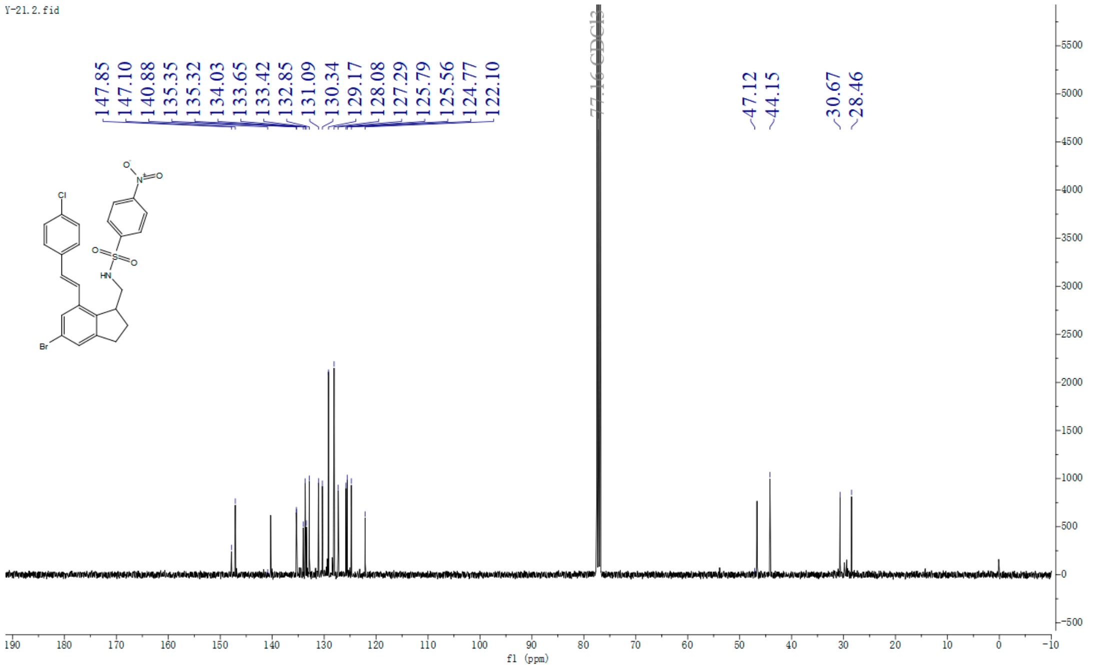

Figure S56. The ^{13}C spectrum (101MHz, $CDCl_3$) of **2r**.

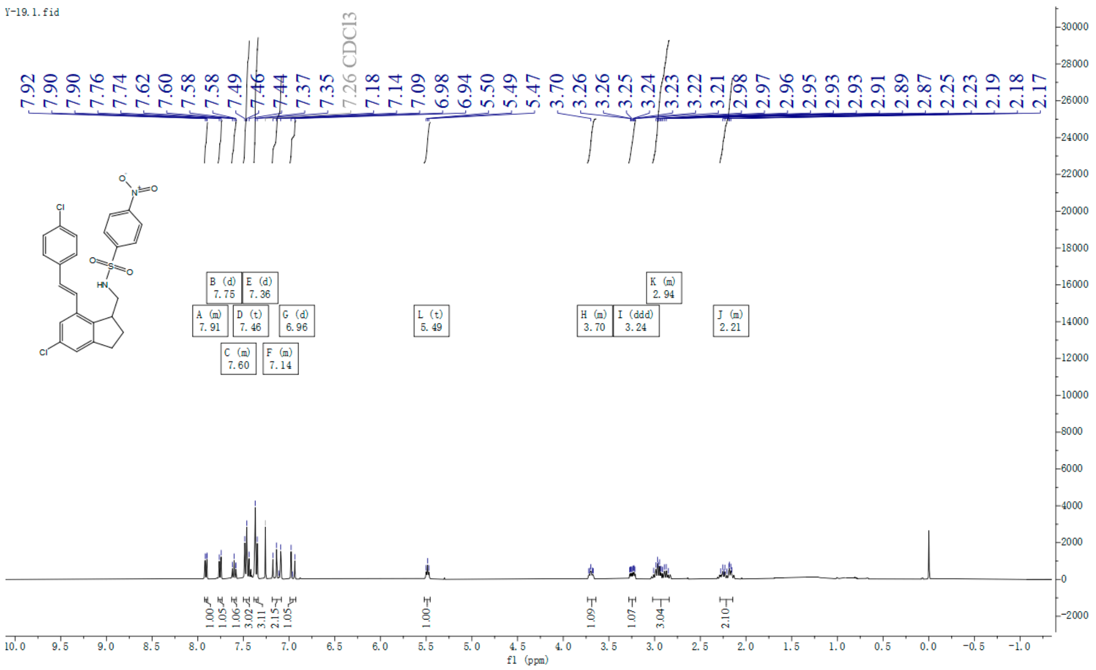

Figure S57. The ^{1}H spectrum (400MHz, CDCl3) of **2s**.

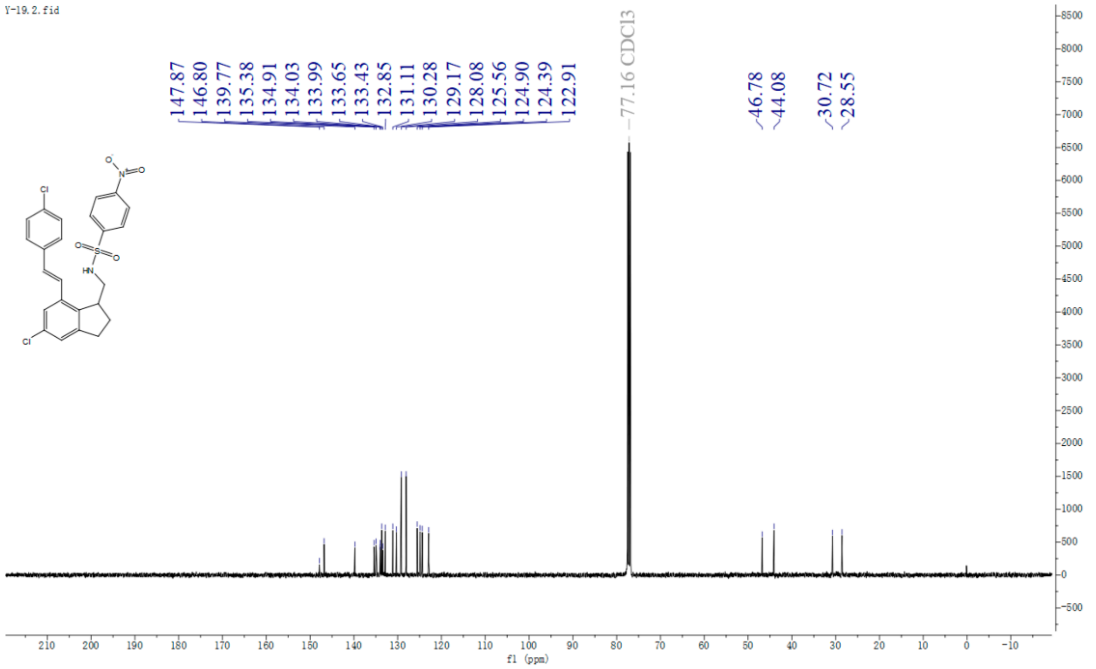

Figure S58. The ^{13}C spectrum (101MHz, CDCl3) of **2s**.

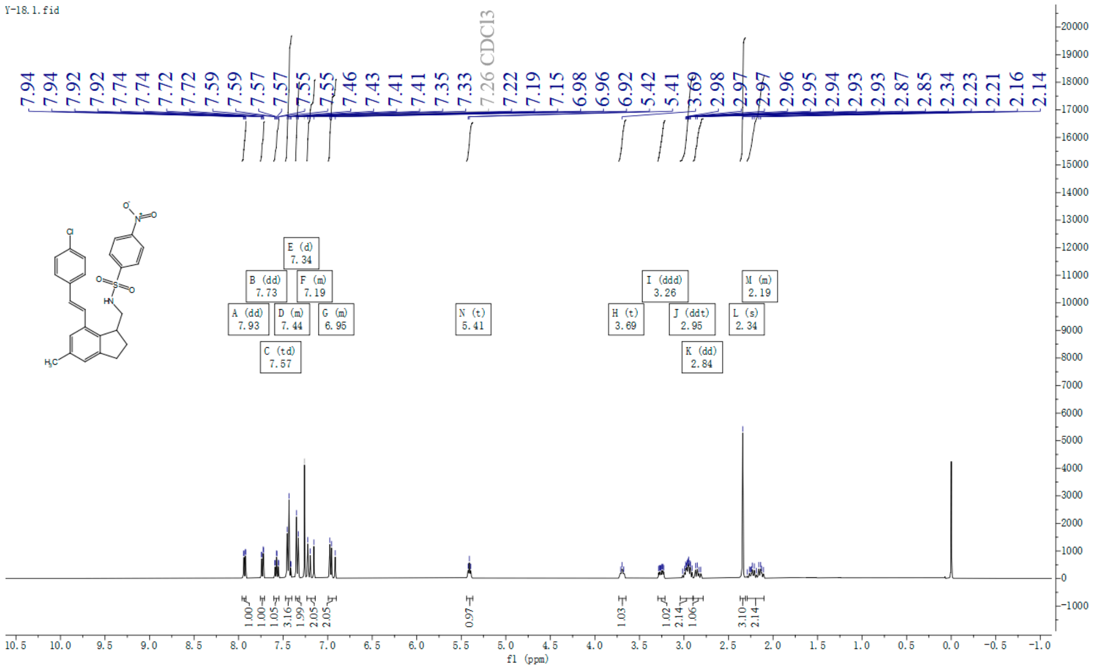

Figure S59. The ^{1}H spectrum (400MHz, $CDCl_3$) of **2t**.

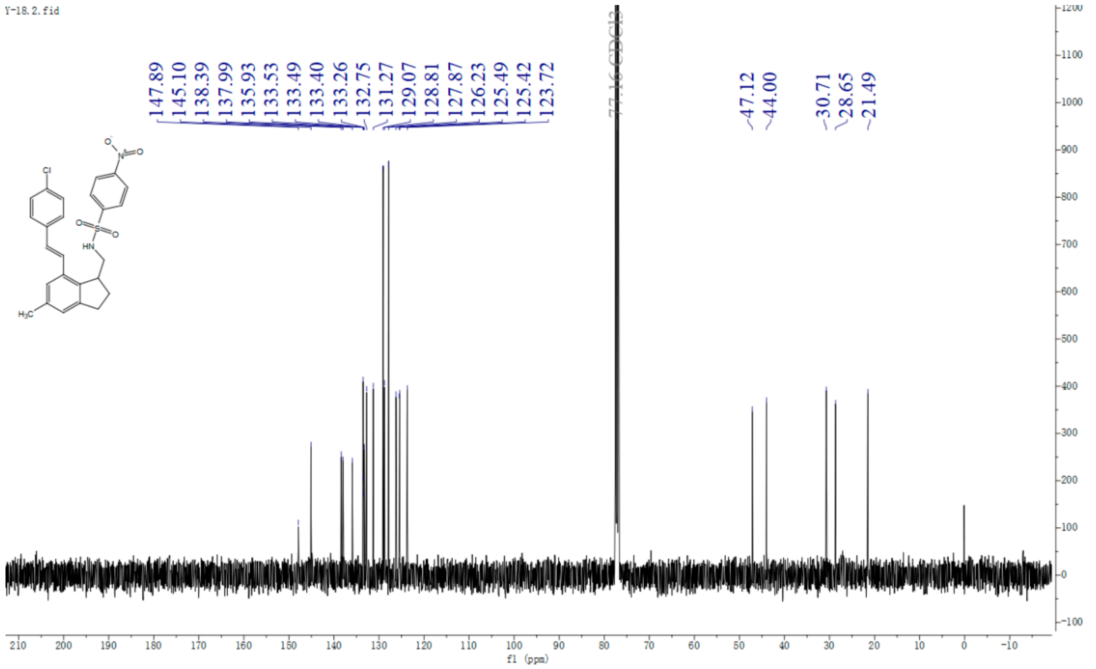

Figure S60. The ^{13}C spectrum (101MHz, $CDCl_3$) of **2t**.

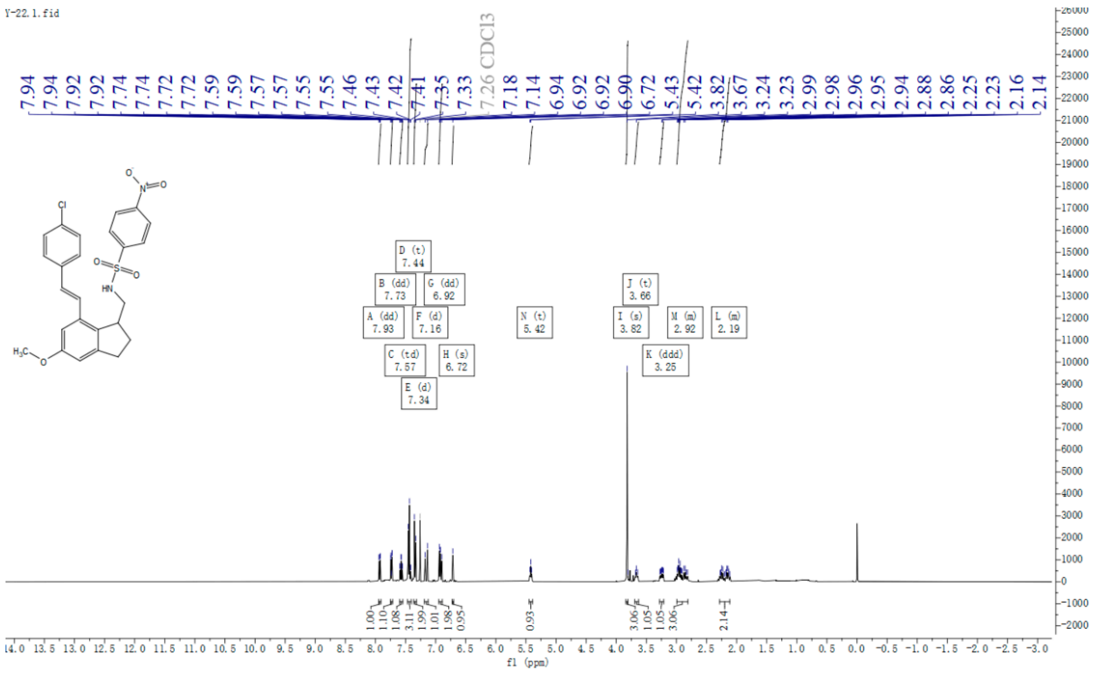

Figure S61. The ^{1}H spectrum (400MHz, $CDCl_3$) of **2u**.

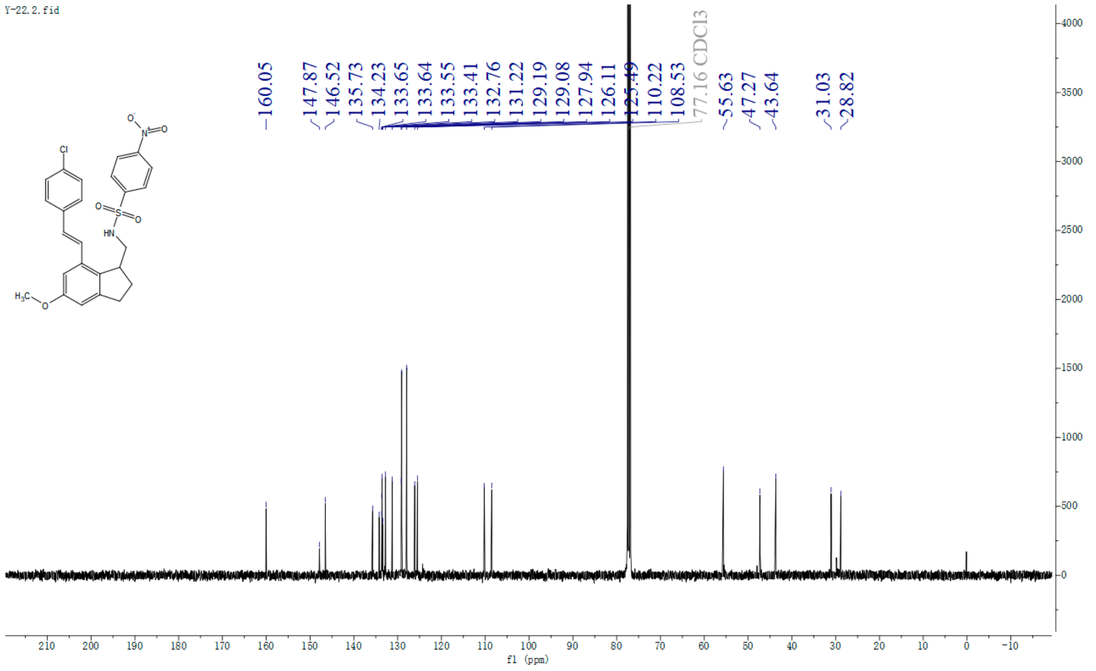

Figure S62. The ^{13}C spectrum (101MHz, $CDCl_3$) of **2u**.

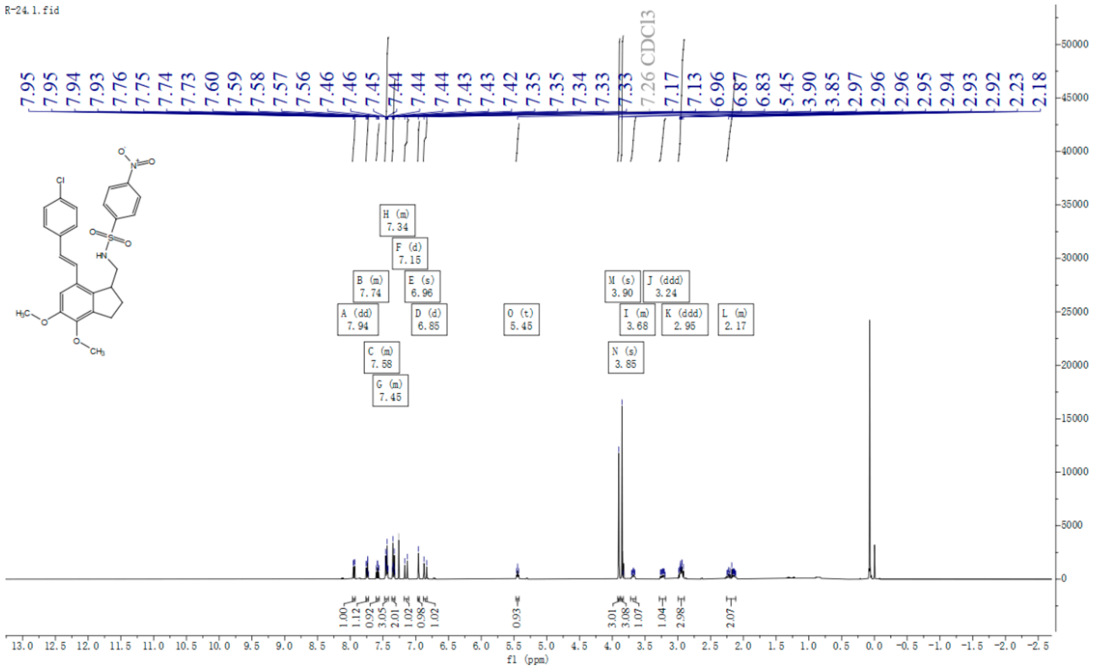

Figure S63. The ^{1}H spectrum (400MHz, $CDCl_3$) of **2v**.

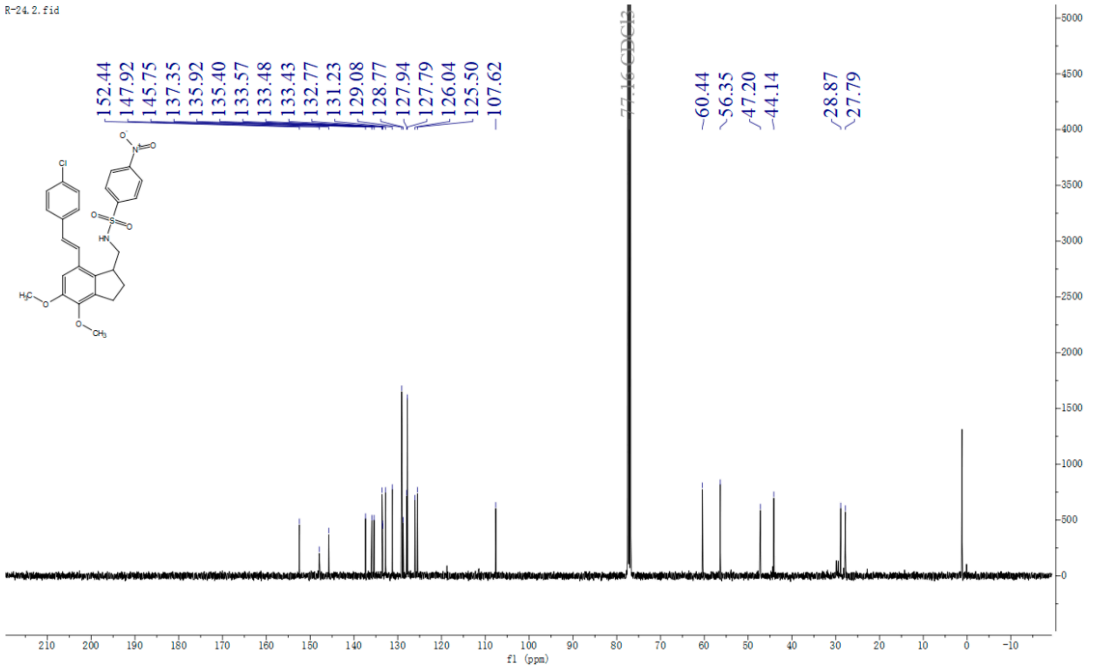

Figure S64. The ^{13}C spectrum (101MHz, $CDCl_3$) of **2v**.

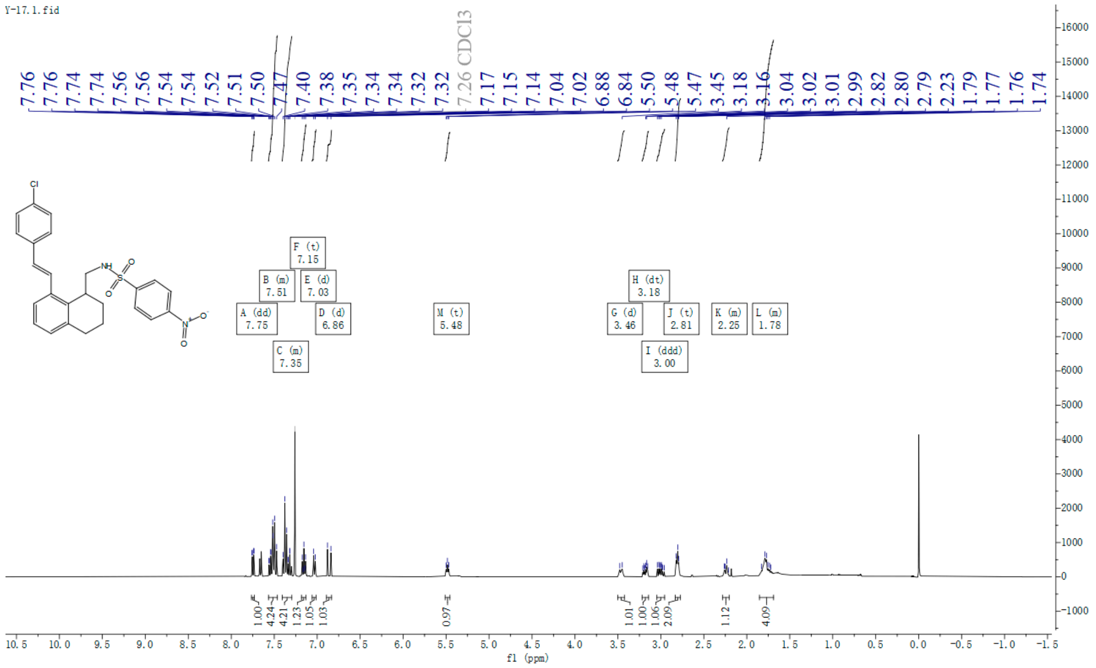

Figure S65. The ^{1}H spectrum (400MHz, $CDCl_3$) of **2w**.

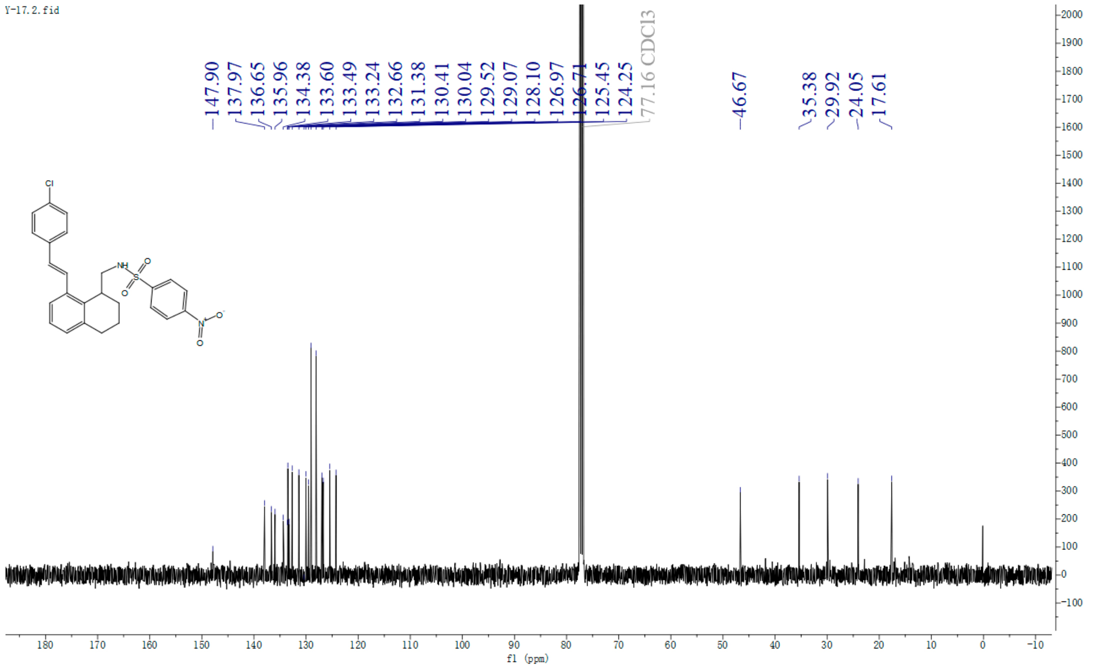

Figure S66. The ^{13}C spectrum (101MHz, $CDCl_3$) of **2w**.

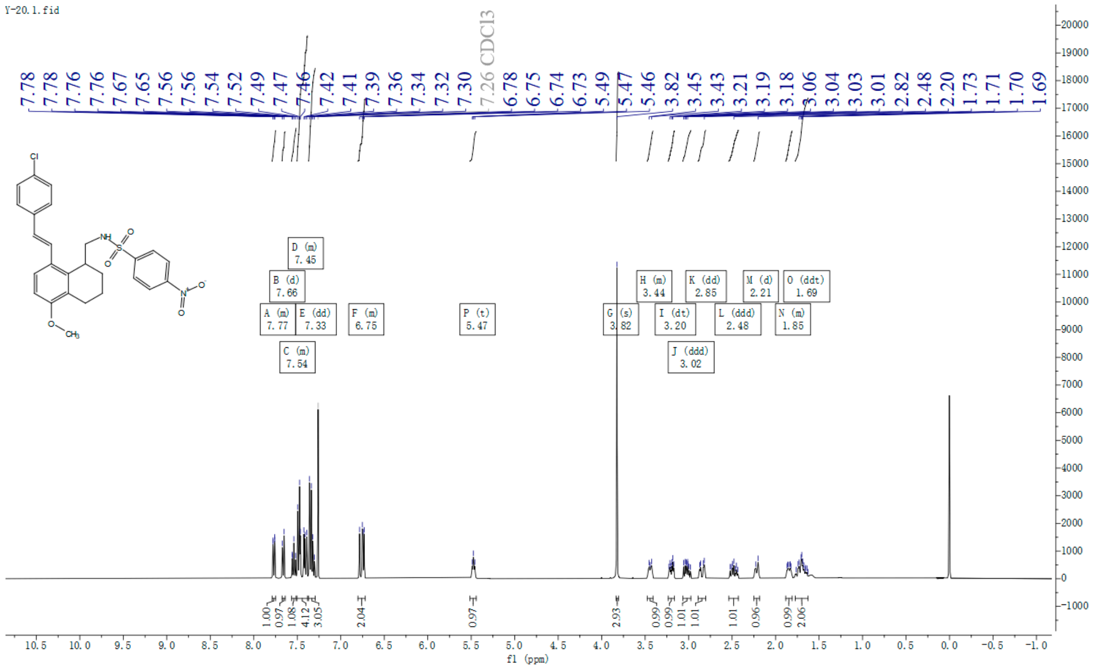

Figure S67. The ^{1}H spectrum (400MHz, $CDCl_3$) of **2x**.

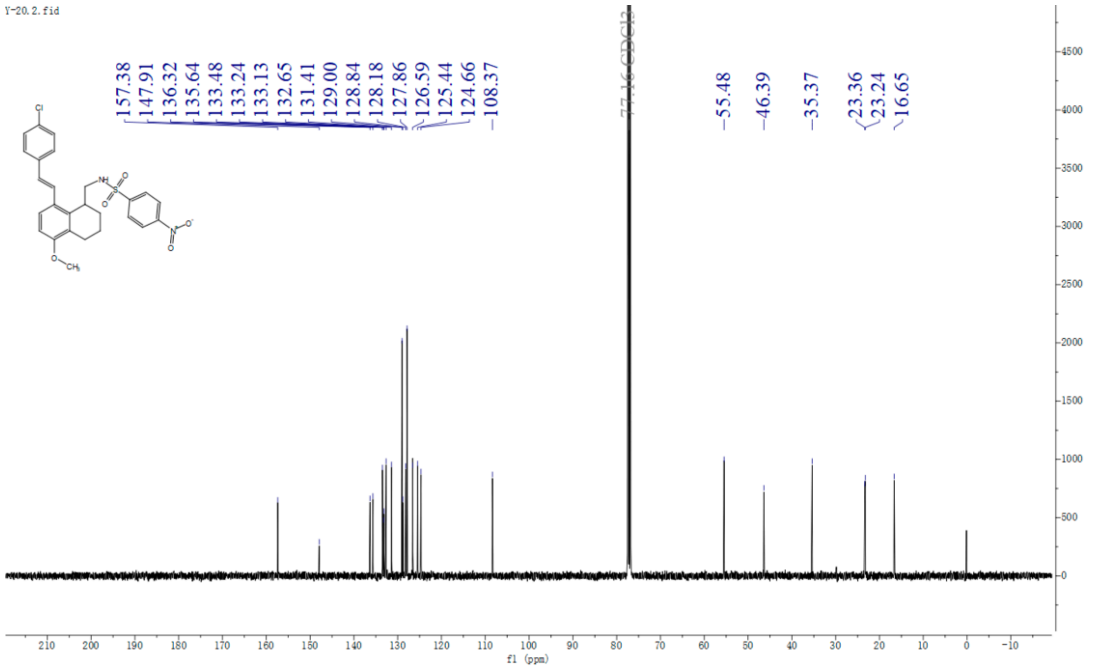

Figure S68. The ^{13}C spectrum (101MHz, $CDCl_3$) of **2x**.

3 The ^{1}H spectrum and ^{13}C spectrum of **3**, **5**, **6** and **7**.

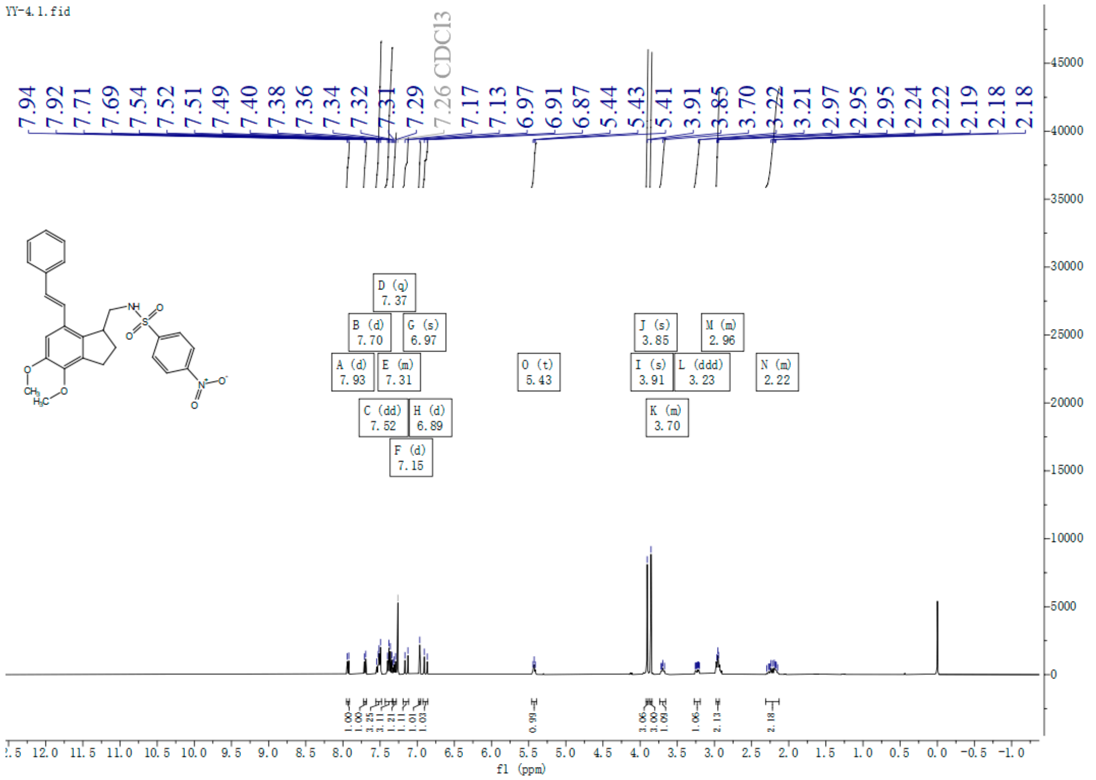

Figure S69. The ^{1}H spectrum (400MHz, $CDCl_3$) of **3**.

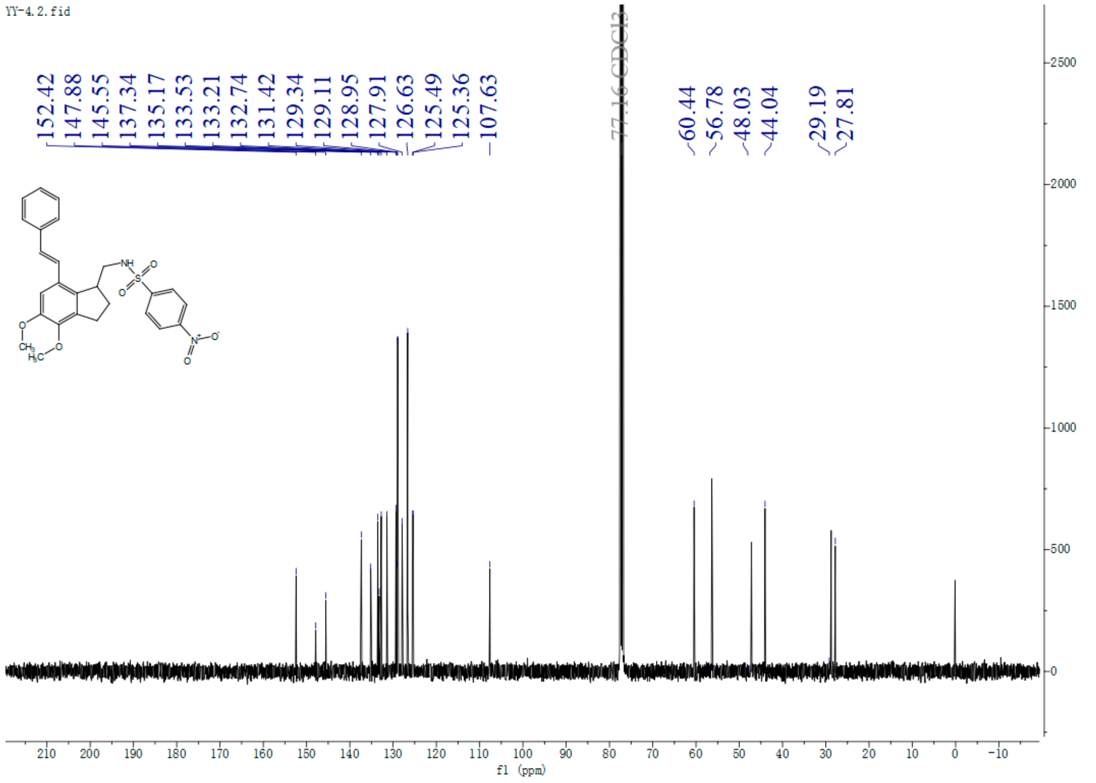

Figure S70. The ^{13}C spectrum (101MHz, $CDCl_3$) of **3**.

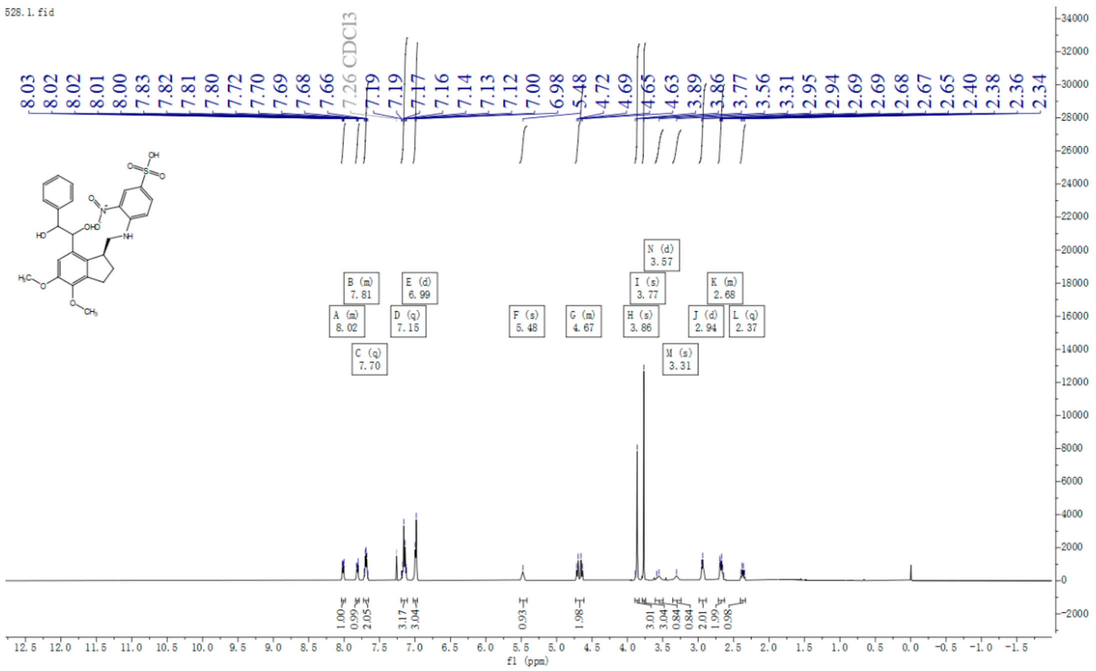

Figure S71. The ^{1}H spectrum (400MHz, $CDCl_3$) of **5**.

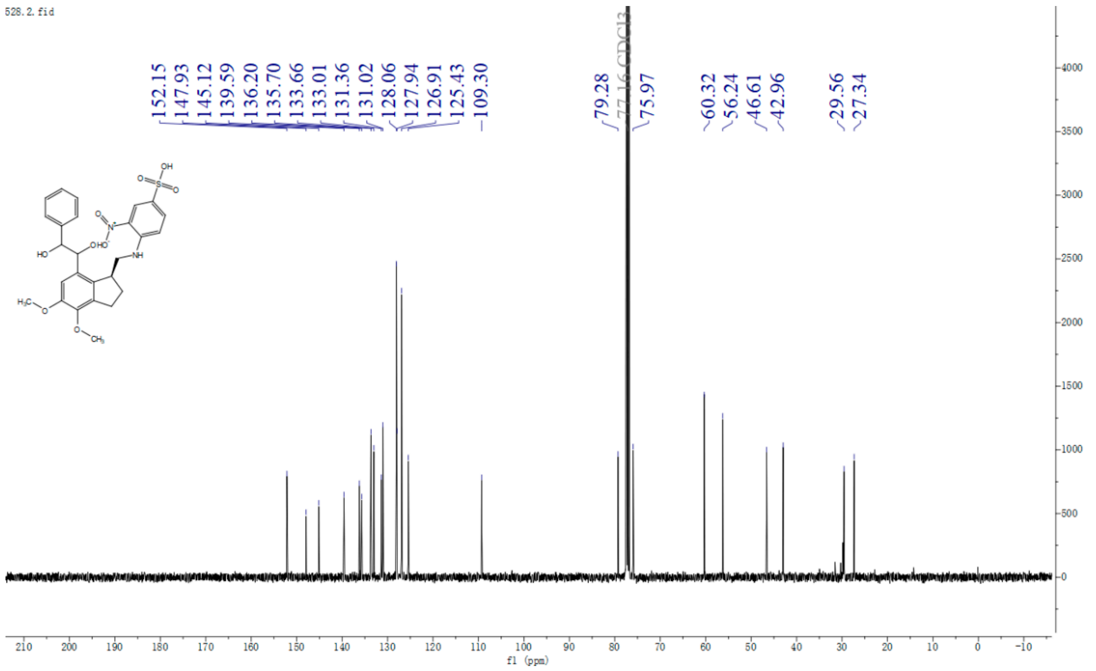

Figure S72. The ^{13}C spectrum (101MHz, $CDCl_3$) of **5**.

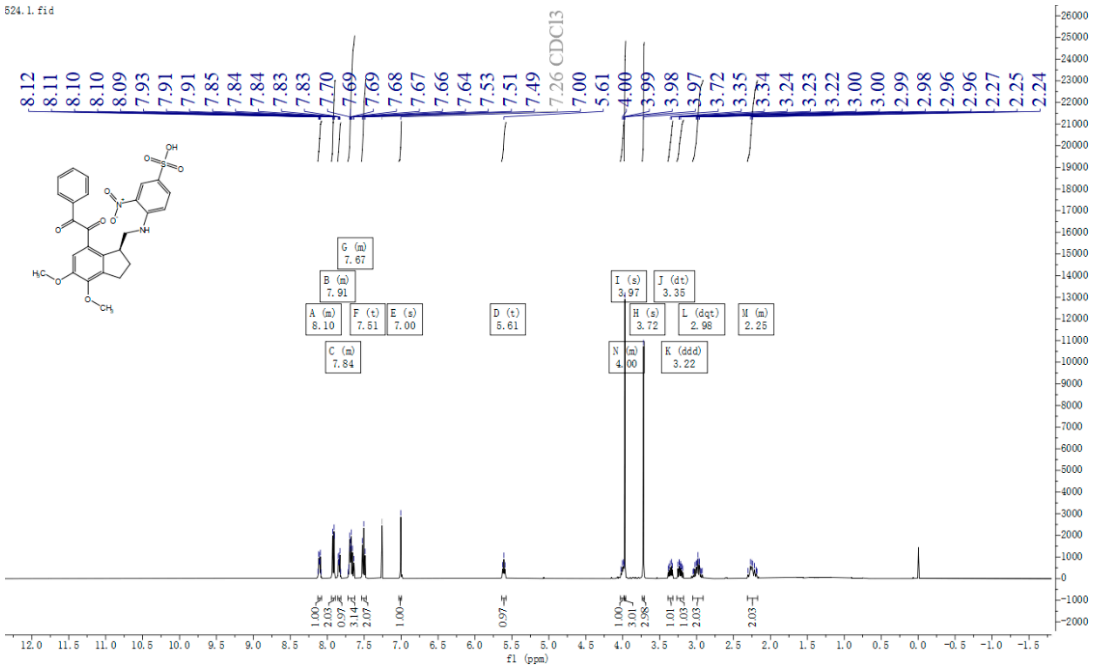

Figure S73. The ^{1}H spectrum (400MHz, $CDCl_3$) of **6**.

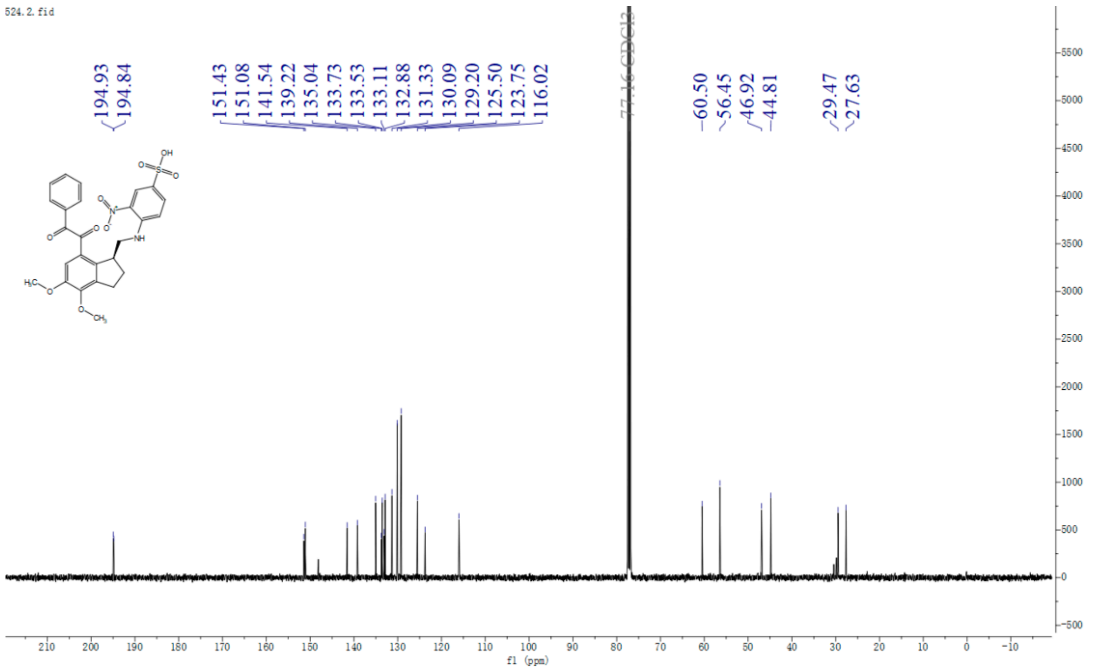

Figure S74. The ^{13}C spectrum (101MHz, $CDCl_3$) of **6**.

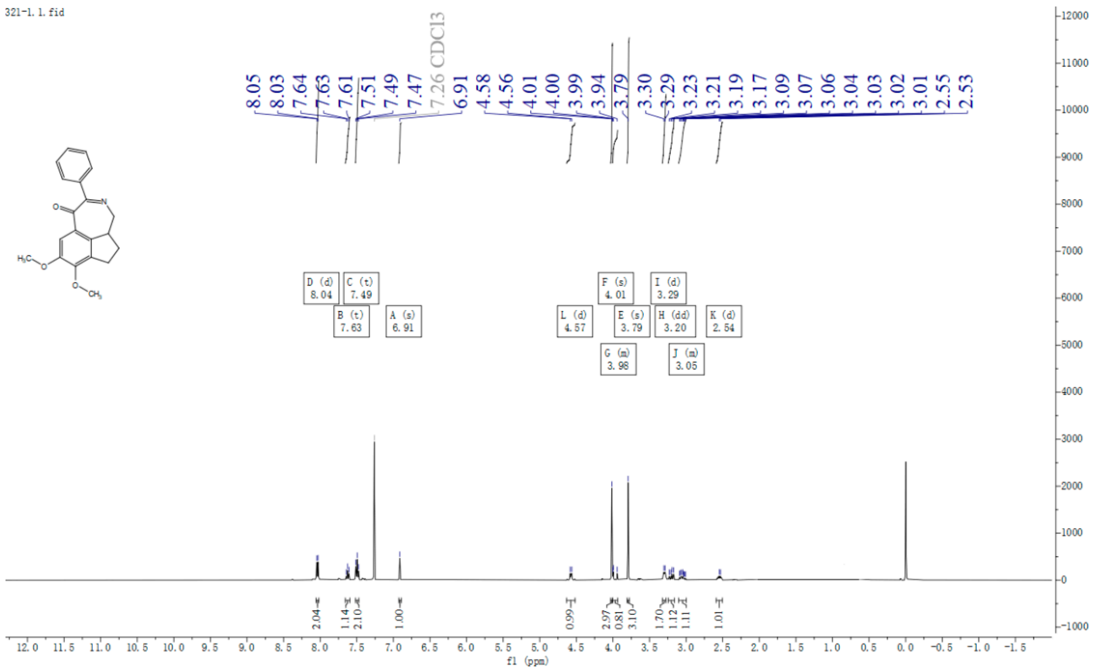

Figure S75. The ^{1}H spectrum (400MHz, $CDCl_3$) of **7**.

4. The HPLC data of **1a** and **2a** in Table 1.

a

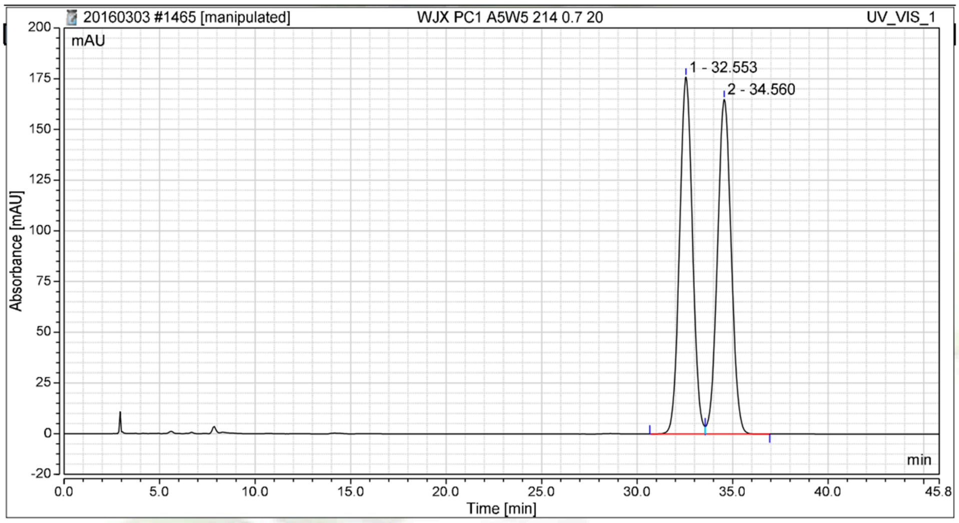

Integration Results				
No.	Retention Time min	Area mAU*min	Height mAU	Relative Area %
1	32.553	137.655	175.951	50.00
2	34.560	137.635	165.000	50.00
**Total:**		**275.290**	**340.951**	**100.00**

b

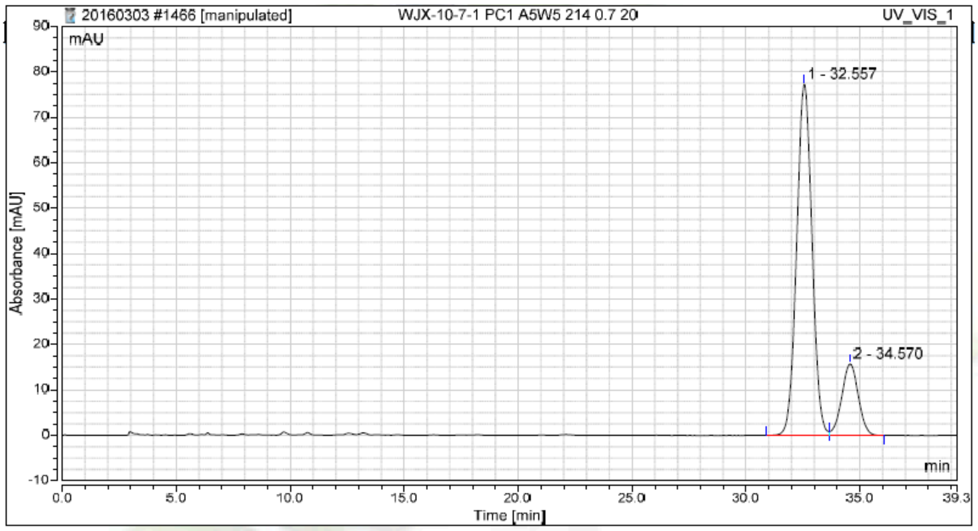

Integration Results				
No.	Retention Time min	Area mAU*min	Height mAU	Relative Area %
1	32.557	60.389	77.338	81.94
2	34.570	13.309	15.781	18.06
**Total:**		**73.699**	**93.119**	**100.00**

Figure S76. The HPLC data of **1a** of entry 6 in Table 1.

a

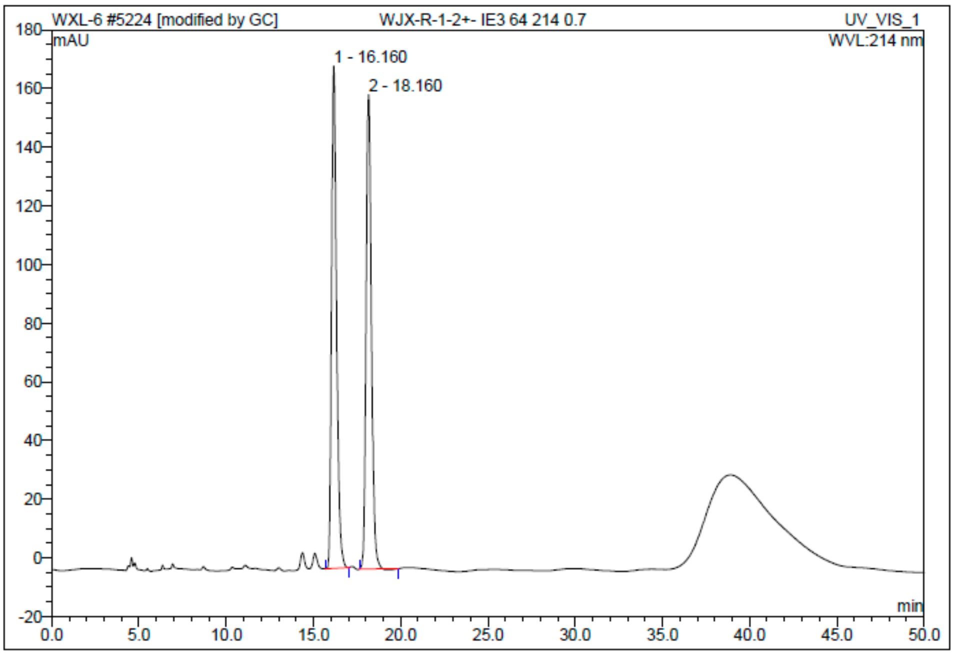

No.	Ret.Time min	Peak Name	Height mAU	Area mAU*min	Rel.Area %	Amount	Type
1	16.16	n.a.	171.396	57.015	50.05	n.a.	BMB*
2	18.16	n.a.	161.771	56.898	49.95	n.a.	BMB*
Total:			333.167	113.913	100.00	0.000	

b

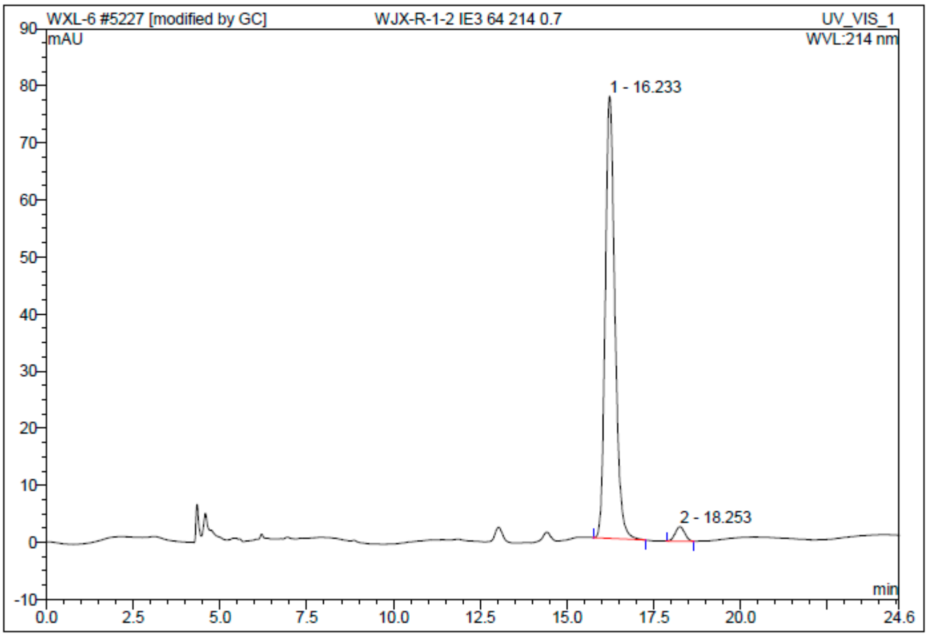

No.	Ret.Time min	Peak Name	Height mAU	Area mAU*min	Rel.Area %	Amount	Type
1	16.23	n.a.	77.488	25.036	96.90	n.a.	BMB
2	18.25	n.a.	2.522	0.801	3.10	n.a.	BMB
Total:			80.010	25.837	100.00	0.000	

Figure S77. The HPLC data of **2a** of entry 6 in Table 1.

5 The HPLC Data of **1ab-an** and **2b-n** in **Scheme 1**.

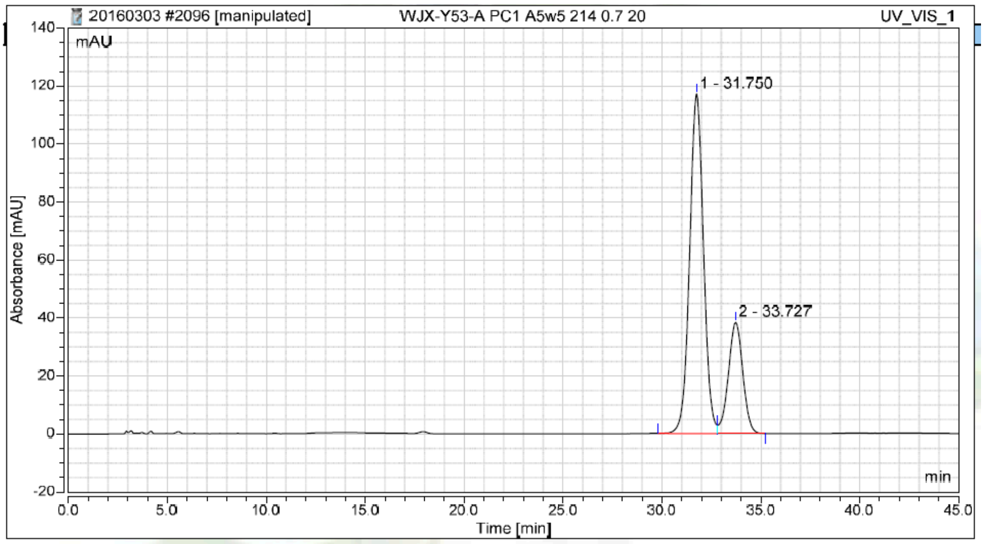

Integration Results					
No.	Retention Time min	Retention Time min	Area mAU*min	Height mAU	Relative Area %
1	32	31.750	95.066	117.091	74.53
2	34	33.727	32.491	38.414	25.47
**Total:**		**127.557**	**155.505**	**100.00**	

Figure S78. The HPLC data of **1ab** in Scheme 1.

a

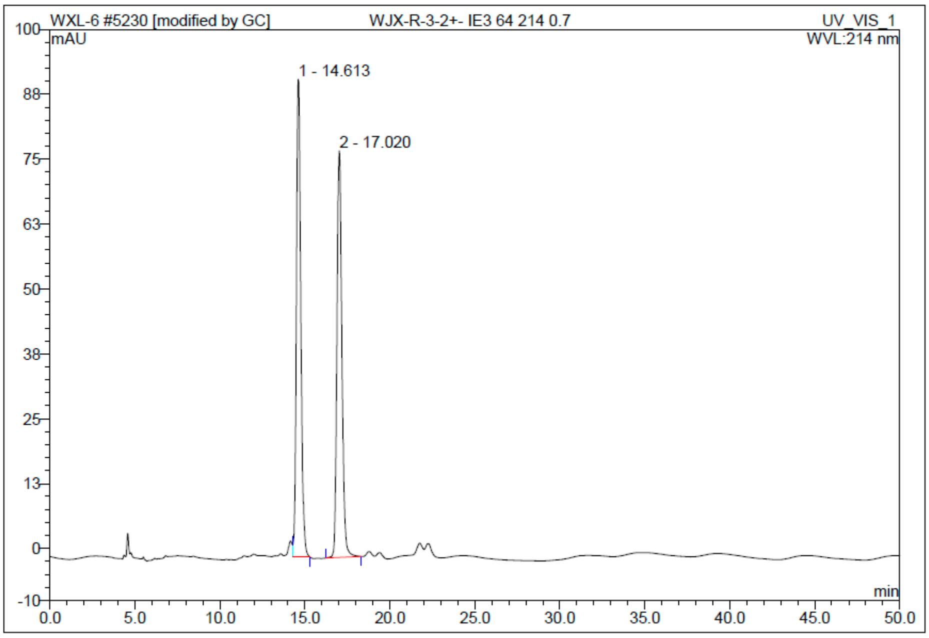

No.	Ret.Time min	Peak Name	Height mAU	Area mAU*min	Rel.Area %	Amount	Type
1	14.61	n.a.	92.089	27.747	51.16	n.a.	MB*
2	17.02	n.a.	78.352	26.487	48.84	n.a.	BMB
Total:			170.441	54.233	100.00	0.000	

b

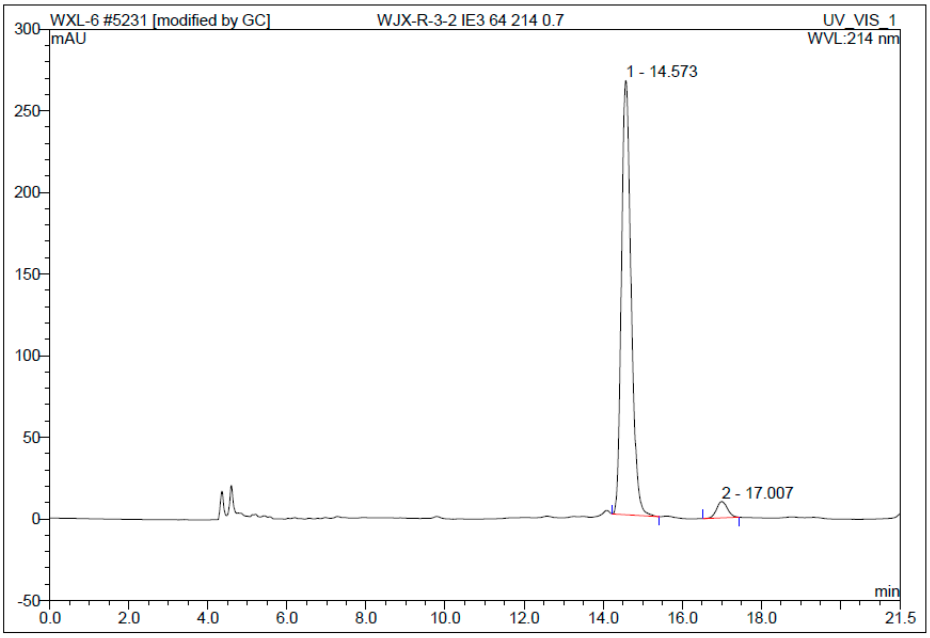

No.	Ret.Time min	Peak Name	Height mAU	Area mAU*min	Rel.Area %	Amount	Type
1	14.57	n.a.	266.005	75.194	95.96	n.a.	BMB*
2	17.01	n.a.	9.935	3.166	4.04	n.a.	BMB*
Total:			275.940	78.360	100.00	0.000	

Figure S79. The HPLC data of **2b** in Scheme 1.

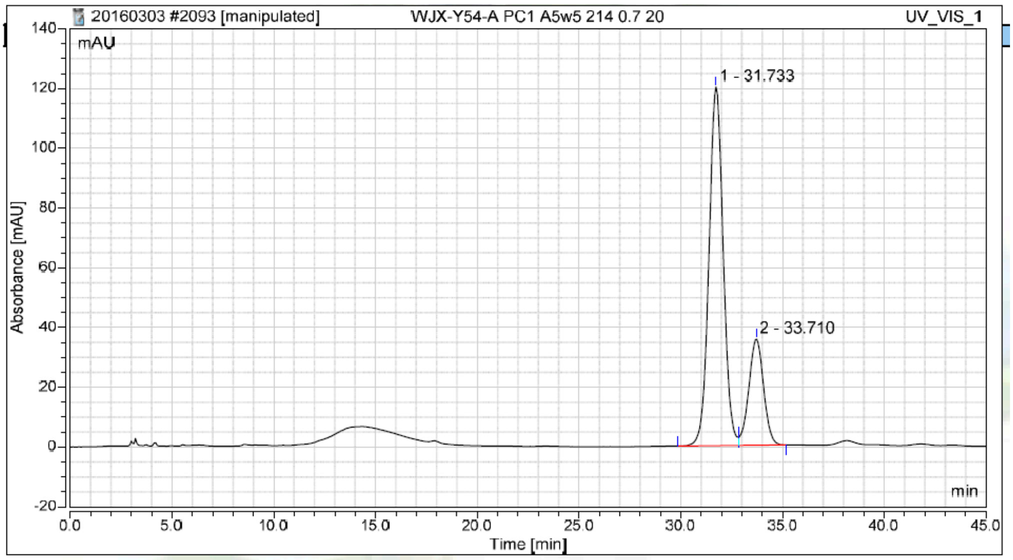

Integration Results					
No.	Retention Time min	Retention Time min	Area mAU*min	Height mAU	Relative Area %
1	32	31.733	97.154	120.188	76.42
2	34	33.710	29.971	35.659	23.58
Total:		127.125	155.848	100.00	

Figure S80. The HPLC data of **1ac** in Scheme 1.

a

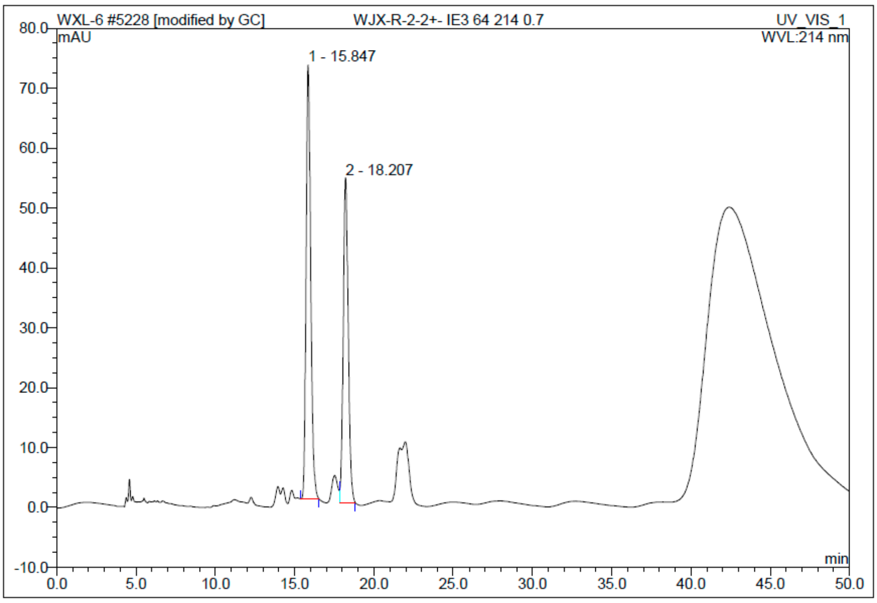

No.	Ret.Time min	Peak Name	Height mAU	Area mAU*min	Rel.Area %	Amount	Type
1	15.85	n.a.	72.510	24.421	55.23	n.a.	BMB*
2	18.21	n.a.	54.260	19.799	44.77	n.a.	MB*
Total:			126.770	44.219	100.00	0.000	

b

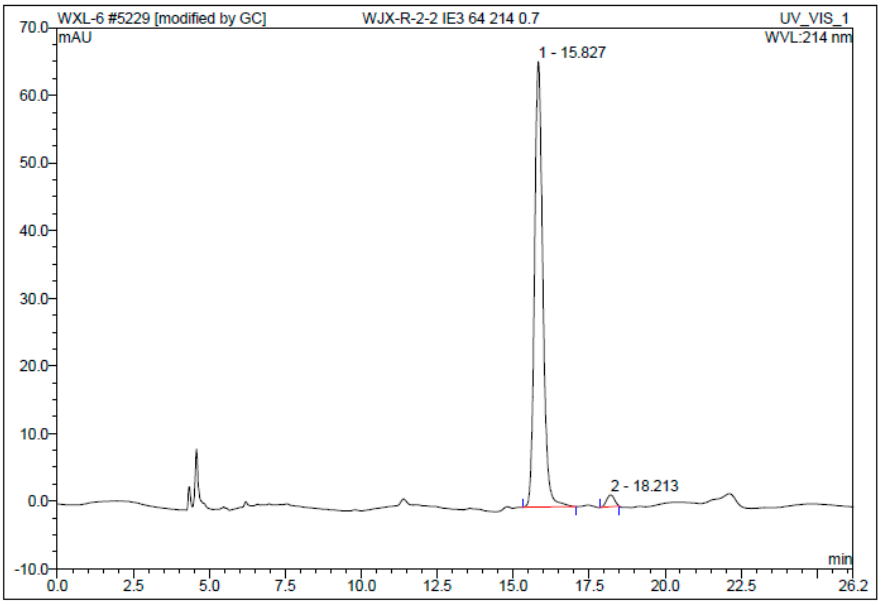

No.	Ret.Time min	Peak Name	Height mAU	Area mAU*min	Rel.Area %	Amount	Type
1	15.83	n.a.	65.875	20.908	97.55	n.a.	BMB
2	18.21	n.a.	1.753	0.526	2.45	n.a.	BMB*
Total:			67.628	21.434	100.00	0.000	

Figure S81. The HPLC data of **2c** in Scheme 1.

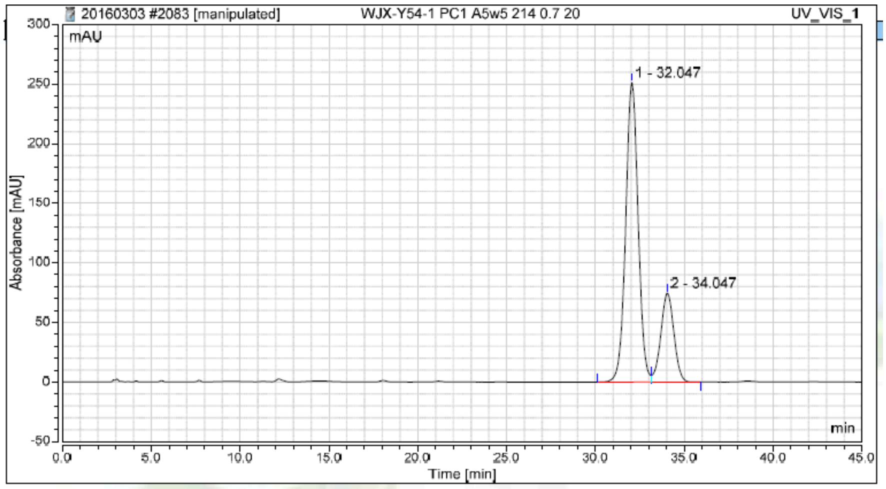

Integration Results					
No.	Retention Time min	Retention Time min	Area mAU*min	Height mAU	Relative Area %
1	32	32.047	204.506	251.342	76.27
2	34	34.047	63.641	74.709	23.73
Total:		268.147	326.051	100.00	

Figure S82. The HPLC data of **1ad** in Scheme 1.

a

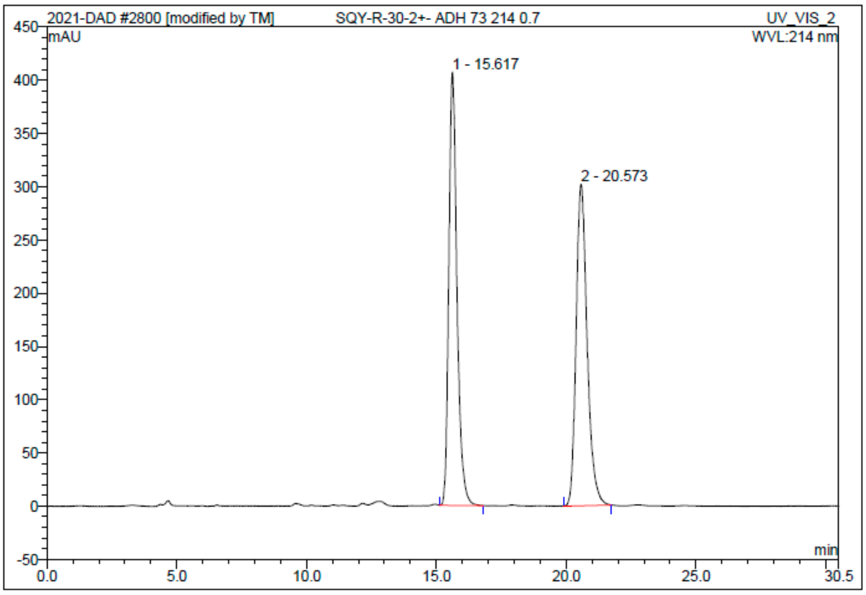

No.	Ret.Time min	Peak Name	Height mAU	Area mAU*min	Rel.Area %	Amount	Type
1	15.62	n.a.	407.077	148.270	49.95	n.a.	BMB*
2	20.57	n.a.	301.935	148.571	50.05	n.a.	BMB
Total:			709.012	296.841	100.00	0.000	

b

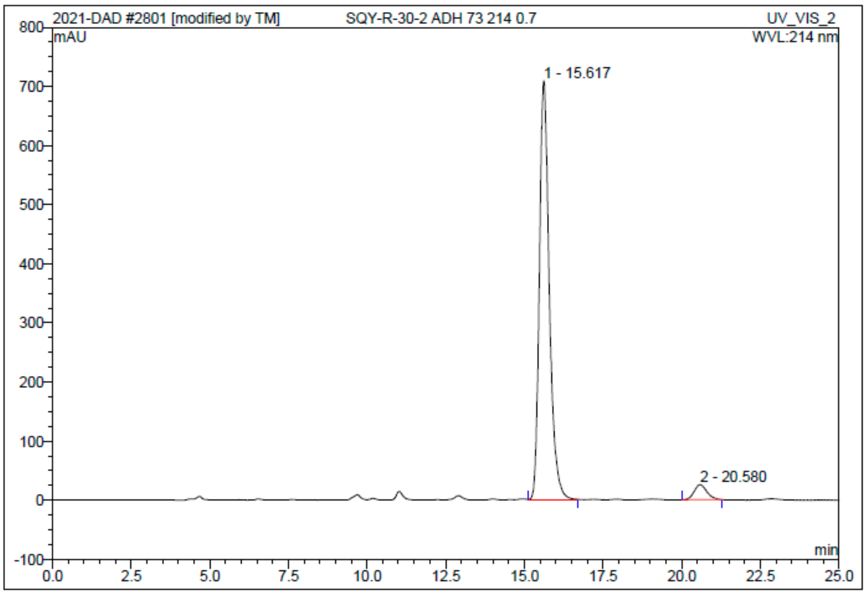

No.	Ret.Time min	Peak Name	Height mAU	Area mAU*min	Rel.Area %	Amount	Type
1	15.62	n.a.	708.601	258.496	95.55	n.a.	BMB
2	20.58	n.a.	25.390	12.034	4.45	n.a.	BMB
Total:			733.991	270.530	100.00	0.000	

Figure S83. The HPLC data of **2d** in Scheme 1.

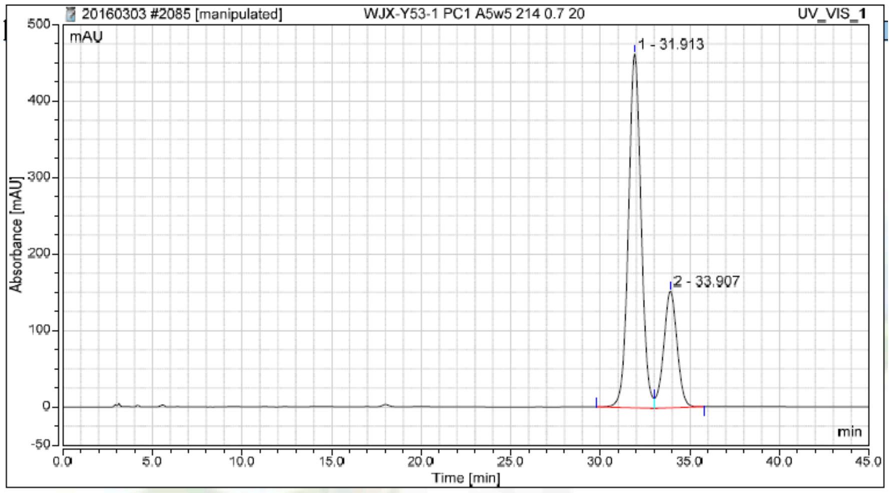

Integration Results					
No.	Retention Time min	Retention Time min	Area mAU*min	Height mAU	Relative Area %
1	32	31.913	378.811	462.695	74.16
2	34	33.907	131.987	152.756	25.84
Total:		510.798	615.451	100.00	

Figure S84. The HPLC data of **1ae** in Scheme 1.

a

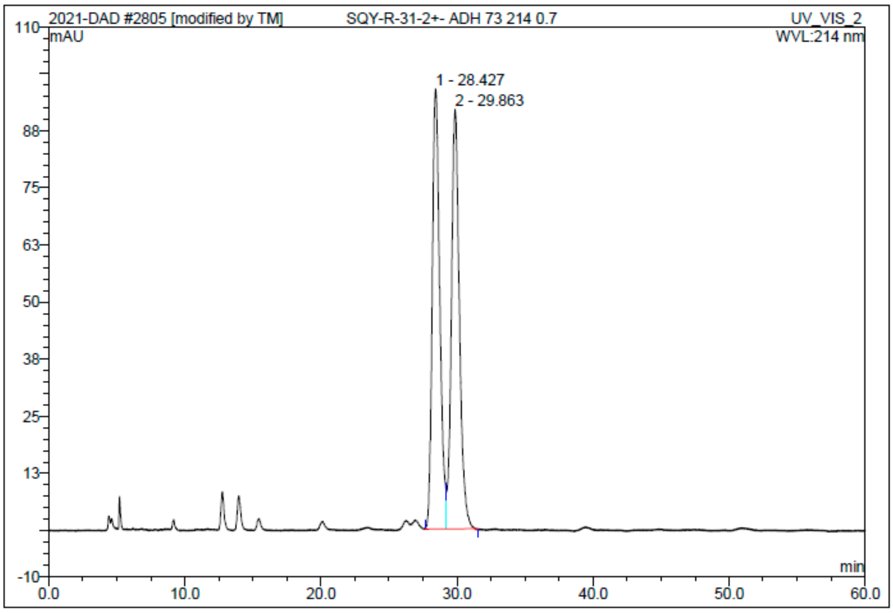

No.	Ret.Time min	Peak Name	Height mAU	Area mAU*min	Rel.Area %	Amount	Type
1	28.43	n.a.	96.336	61.657	49.69	n.a.	BM *
2	29.86	n.a.	91.844	62.436	50.31	n.a.	MB*
Total:			188.179	124.093	100.00	0.000	

b

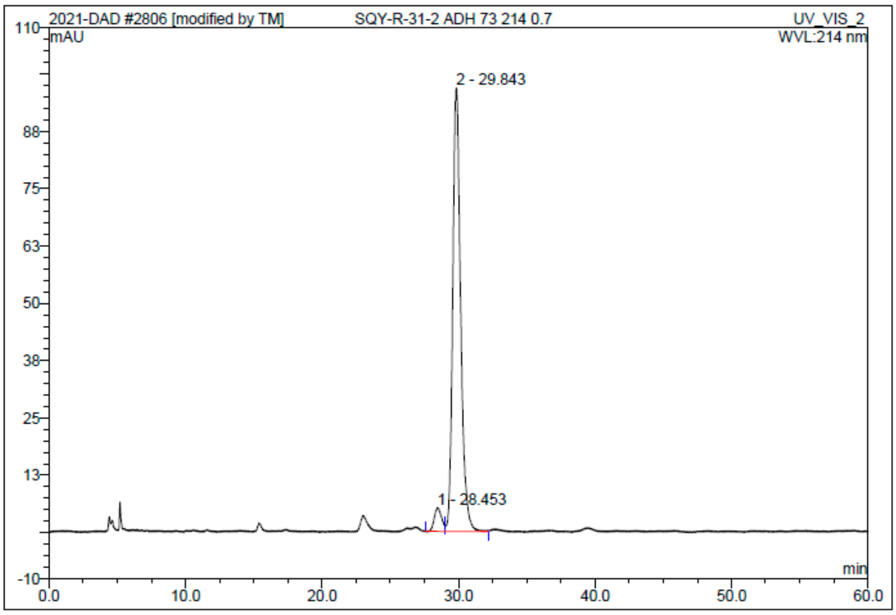

No.	Ret.Time min	Peak Name	Height mAU	Area mAU*min	Rel.Area %	Amount	Type
1	28.45	n.a.	5.188	3.150	4.63	n.a.	BM *
2	29.84	n.a.	96.727	64.928	95.37	n.a.	MB*
Total:			101.915	68.078	100.00	0.000	

Figure S85. The HPLC data of **2e** in Scheme 1.

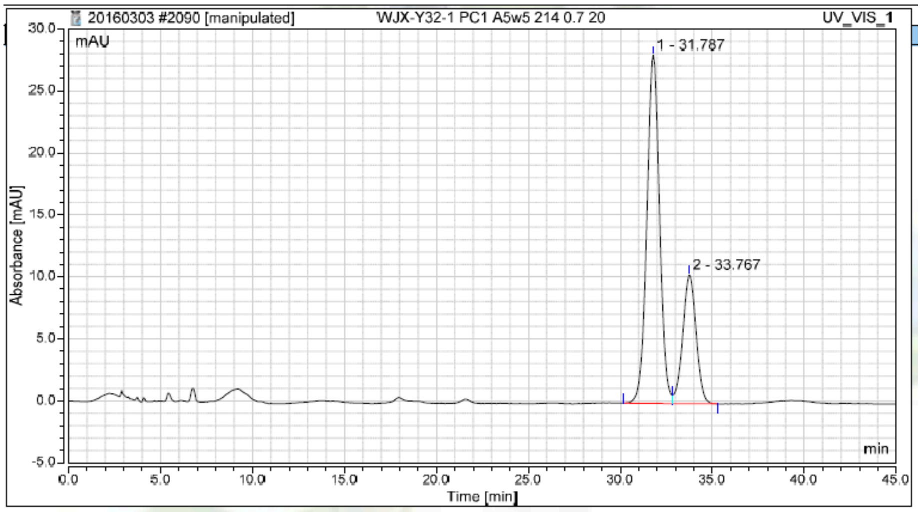

Integration Results					
No.	Retention Time min	Retention Time min	Area mAU*min	Height mAU	Relative Area %
1	32	31.787	22.506	28.087	71.97
2	34	33.767	8.764	10.399	28.03
**Total:**		**31.271**	**38.486**	**100.00**	

Figure S86. The HPLC data of **1af** in Scheme 1.

a

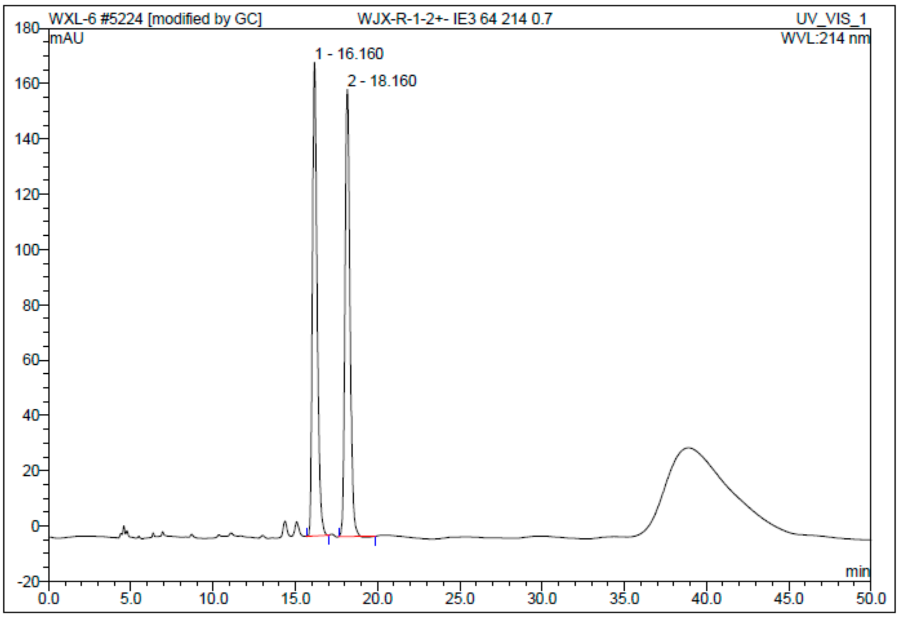

No.	Ret.Time min	Peak Name	Height mAU	Area mAU*min	Rel.Area %	Amount	Type
1	16.16	n.a.	171.396	57.015	50.05	n.a.	BMB*
2	18.16	n.a.	161.771	56.898	49.95	n.a.	BMB*
Total:			333.167	113.913	100.00	0.000	

b

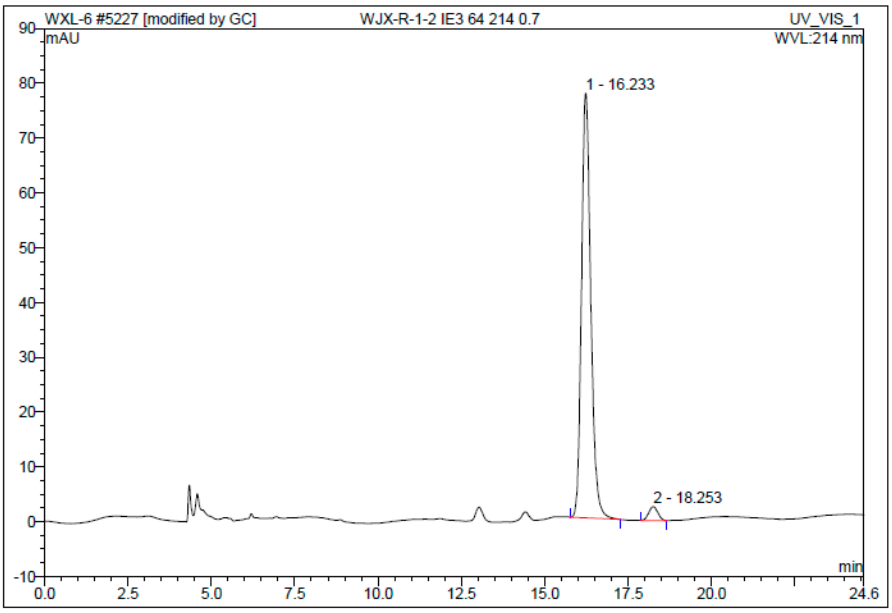

No.	Ret.Time min	Peak Name	Height mAU	Area mAU*min	Rel.Area %	Amount	Type
1	16.23	n.a.	77.488	25.036	96.90	n.a.	BMB
2	18.25	n.a.	2.522	0.801	3.10	n.a.	BMB
Total:			80.010	25.837	100.00	0.000	

Figure S87. The HPLC data of **2f** in Scheme 1.

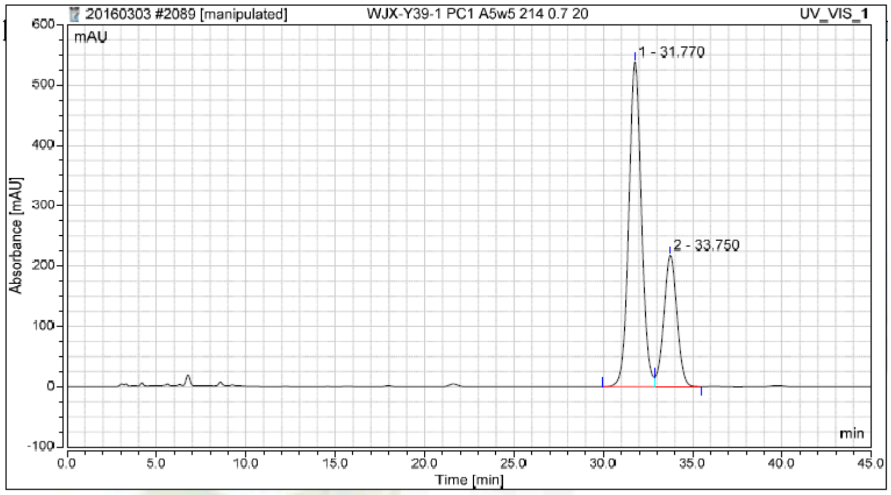

Integration Results					
No.	Retention Time min	Retention Time min	Area mAU*min	Height mAU	Relative Area %
1	32	31.770	440.427	538.262	70.46
2	34	33.750	184.674	217.817	29.54
Total:		625.102	756.078	100.00	

Figure S88. The HPLC data of **1ag** in Scheme 1.

a

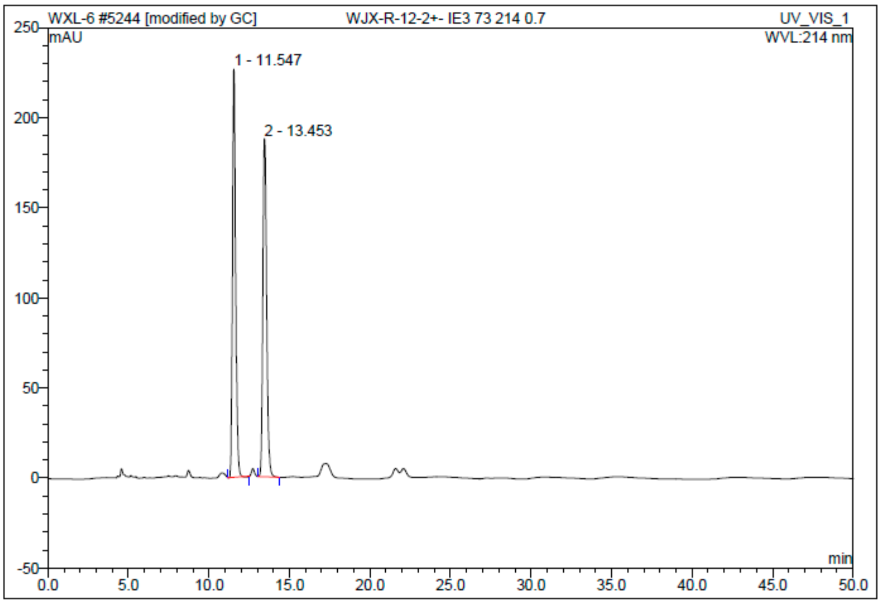

No.	Ret.Time min	Peak Name	Height mAU	Area mAU*min	Rel.Area %	Amount	Type
1	11.55	n.a.	226.585	52.036	50.49	n.a.	BMB*
2	13.45	n.a.	187.758	51.029	49.51	n.a.	BMB*
Total:			414.343	103.065	100.00	0.000	

b

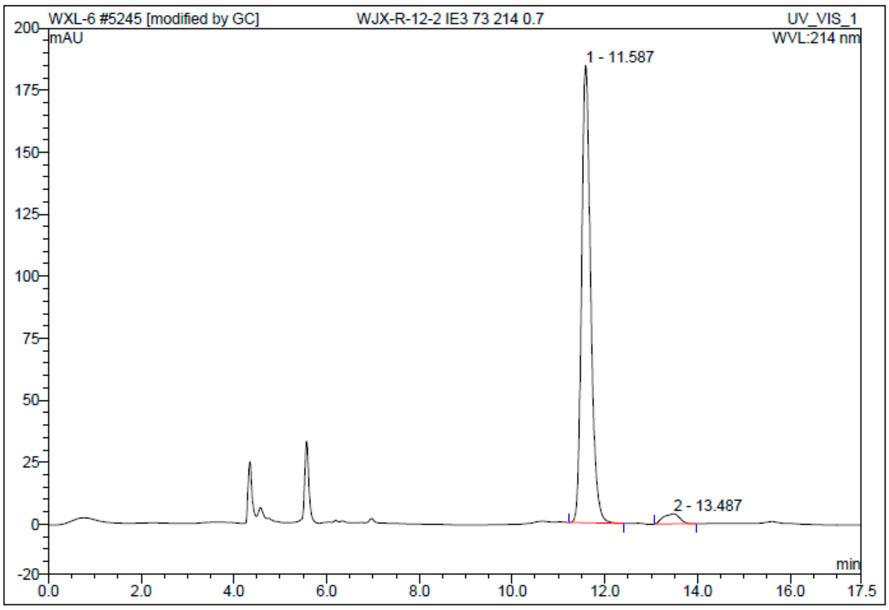

No.	Ret.Time min	Peak Name	Height mAU	Area mAU*min	Rel.Area %	Amount	Type
1	11.59	n.a.	184.389	41.907	96.20	n.a.	BMB
2	13.49	n.a.	4.034	1.657	3.80	n.a.	BMB*
Total:			188.423	43.564	100.00	0.000	

Figure S89. The HPLC data of **2g** in Scheme 1.

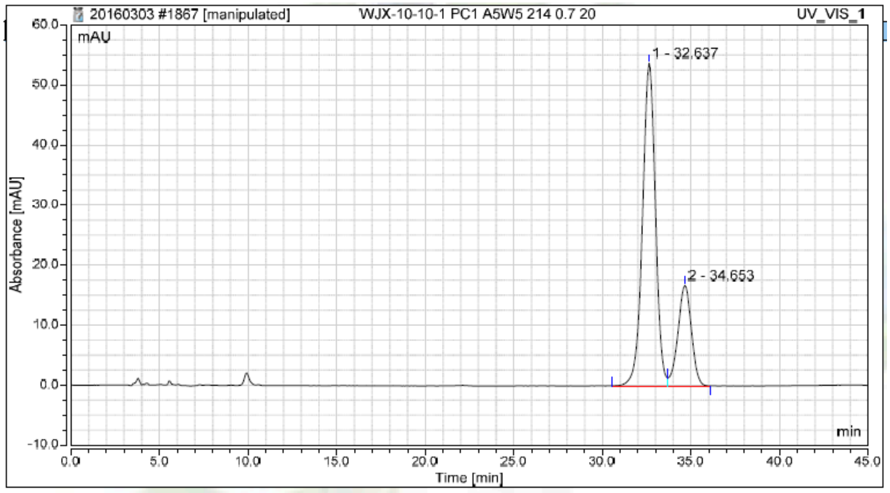

Integration Results					
No.	Retention Time min	Retention Time min	Area mAU*min	Height mAU	Relative Area %
1	33	32.637	45.424	53.781	75.44
2	35	34.653	14.791	16.760	24.56
Total:		60.216	70.541	100.00	

Figure S90. The HPLC data of **1ah** in Scheme 1.

a

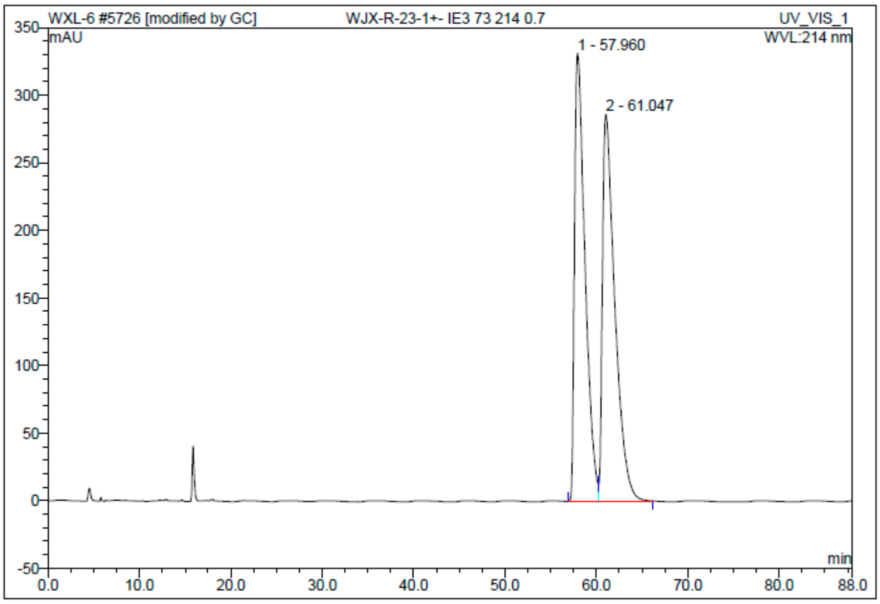

No.	Ret.Time min	Peak Name	Height mAU	Area mAU*min	Rel.Area %	Amount	Type
1	57.96	n.a.	331.591	442.612	49.58	n.a.	BM *
2	61.05	n.a.	286.148	450.117	50.42	n.a.	MB*
Total:			617.739	892.729	100.00	0.000	

b

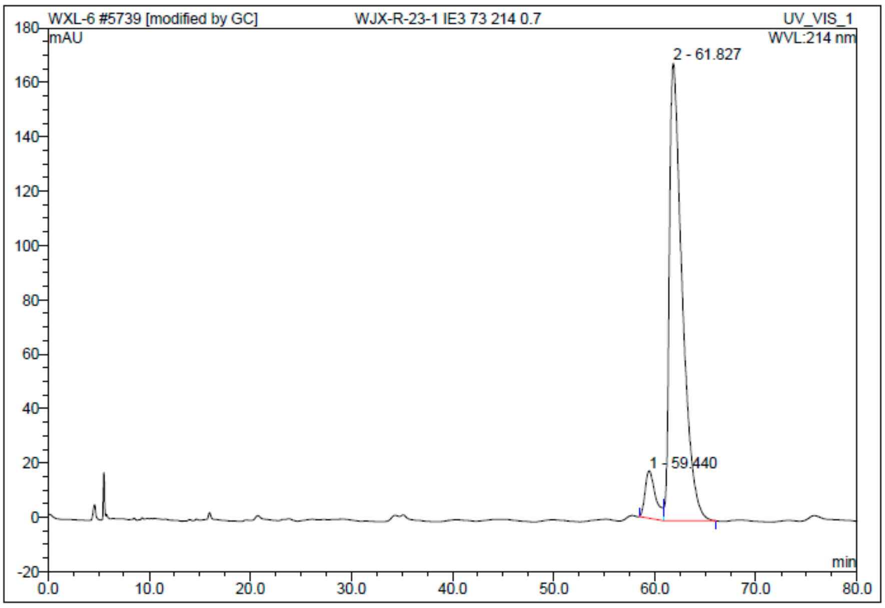

No.	Ret.Time min	Peak Name	Height mAU	Area mAU*min	Rel.Area %	Amount	Type
1	59.44	n.a.	17.472	20.980	7.79	n.a.	BM *
2	61.83	n.a.	168.376	248.168	92.21	n.a.	MB*
Total:			185.848	269.148	100.00	0.000	

Figure S91. The HPLC data of **2h** in Scheme 1.

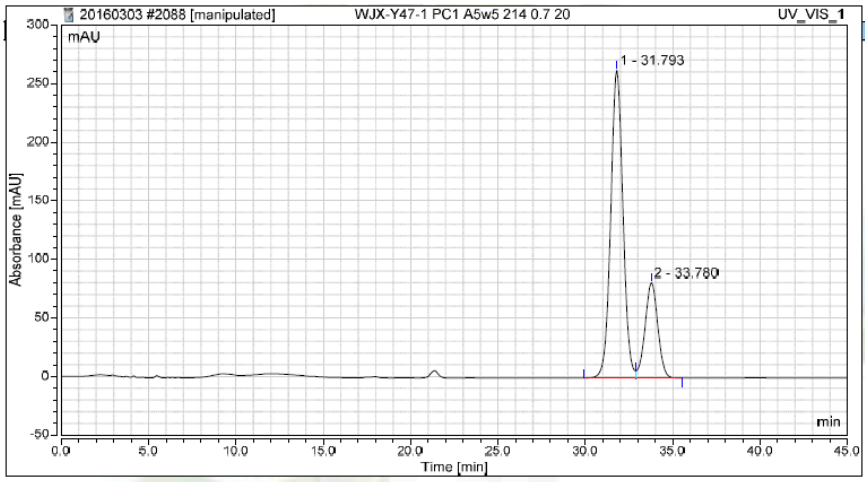

Integration Results					
No.	Retention Time min	Retention Time min	Area mAU*min	Height mAU	Relative Area %
1	32	31.793	213.273	263.063	75.54
2	34	33.780	69.072	81.512	24.46
Total:		282.346	344.576	100.00	

Figure S92. The HPLC data of **1ai** in Scheme 1.

a

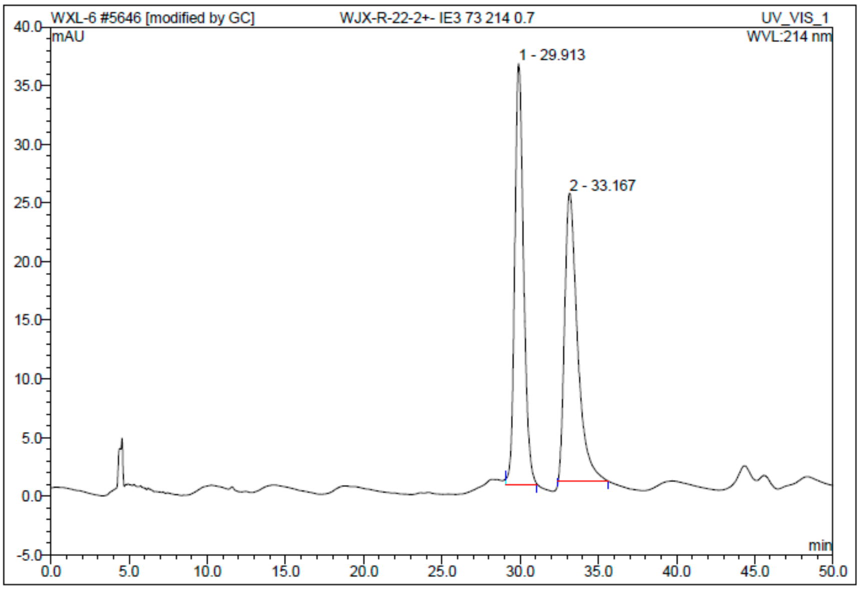

No.	Ret.Time min	Peak Name	Height mAU	Area mAU*min	Rel.Area %	Amount	Type
1	29.91	n.a.	35.858	23.484	50.12	n.a.	MB*
2	33.17	n.a.	24.521	23.371	49.88	n.a.	MB*
Total:			60.379	46.856	100.00	0.000	

b

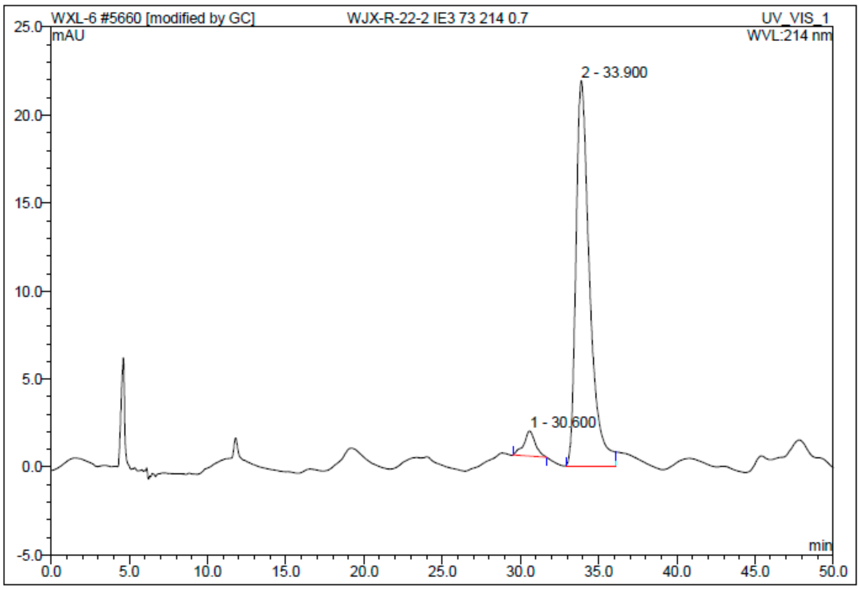

No.	Ret.Time min	Peak Name	Height mAU	Area mAU*min	Rel.Area %	Amount	Type
1	30.60	n.a.	1.431	1.235	5.27	n.a.	BMB
2	33.90	n.a.	21.895	22.203	94.73	n.a.	BM *
Total:			23.326	23.438	100.00	0.000	

Figure S93. The HPLC data of **2i** in Scheme 1.

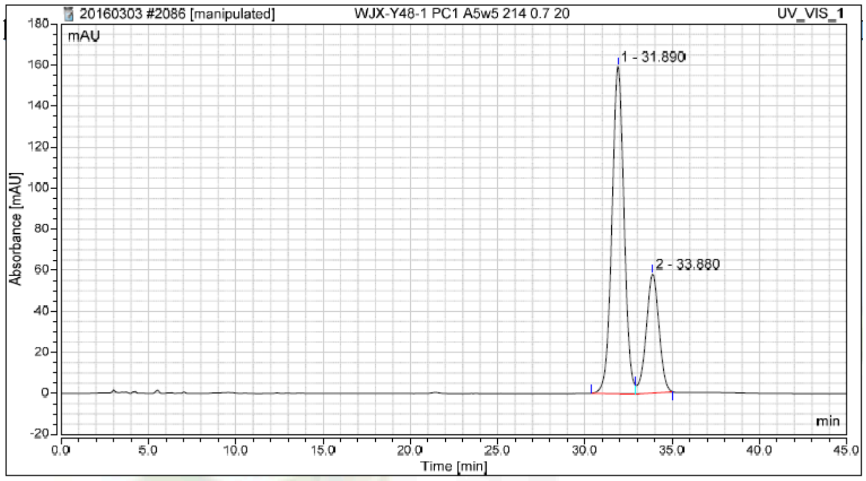

Integration Results					
No.	Retention Time min	Retention Time min	Area mAU*min	Height mAU	Relative Area %
1	32	31.890	127.921	159.693	72.08
2	34	33.880	49.558	58.029	27.92
Total:		177.479	217.722	100.00	

Figure S94. The HPLC data of **1aj** in Scheme 1.

a

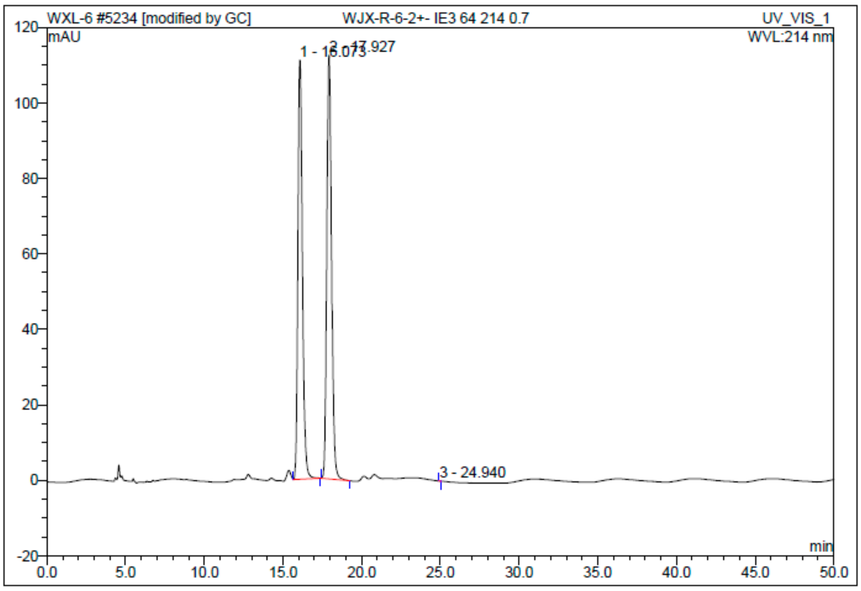

No.	Ret.Time min	Peak Name	Height mAU	Area mAU*min	Rel.Area %	Amount	Type
1	16.07	n.a.	111.085	36.946	48.36	n.a.	BMB*
2	17.93	n.a.	112.280	39.458	51.64	n.a.	BMB
3	24.94	n.a.	0.006	0.000	0.00	n.a.	BMB*
Total:			223.371	76.405	100.00	0.000	

b

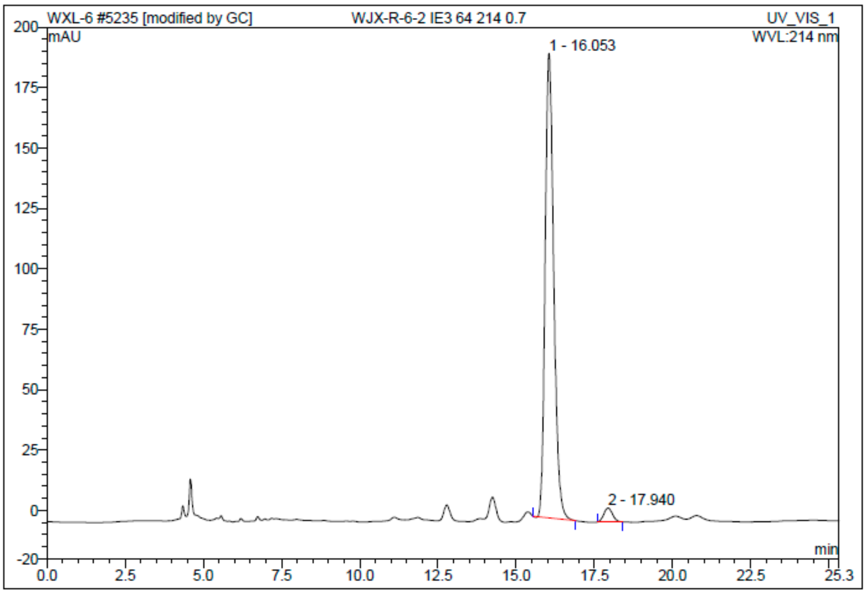

No.	Ret.Time min	Peak Name	Height mAU	Area mAU*min	Rel.Area %	Amount	Type
1	16.05	n.a.	192.285	61.001	97.04	n.a.	BMB*
2	17.94	n.a.	5.746	1.859	2.96	n.a.	BMB*
Total:			198.030	62.860	100.00	0.000	

Figure S95. The HPLC data of **2j** in Scheme 1.

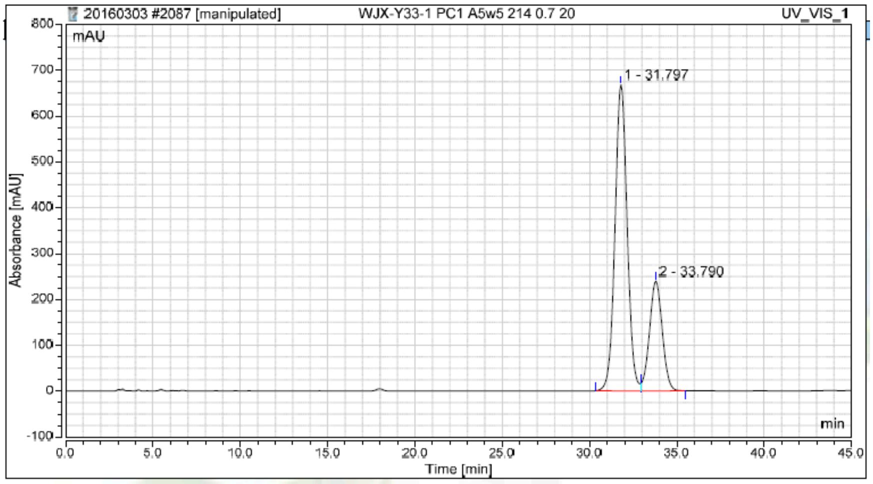

Integration Results					
No.	Retention Time min	Retention Time min	Area mAU*min	Height mAU	Relative Area %
1	32	31.797	539.825	667.940	72.66
2	34	33.790	203.168	239.790	27.34
Total:		742.993	907.730	100.00	

Figure S96. The HPLC data of **1ak** in Scheme 1.

a

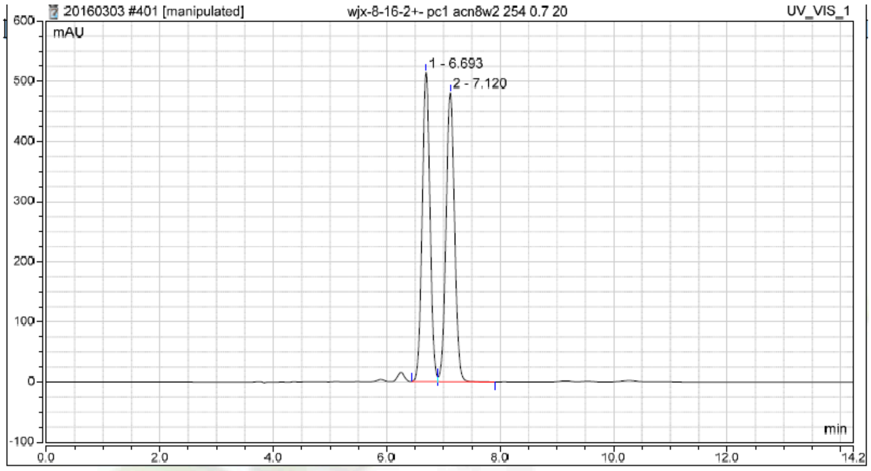

Integration Results				
No.	Retention Time min	Area mAU*min	Height mAU	Relative Area %
1	6.693	82.030	514.254	49.54
2	7.120	83.562	480.307	50.46
**Total:**		**165.591**	**994.561**	**100.00**

b

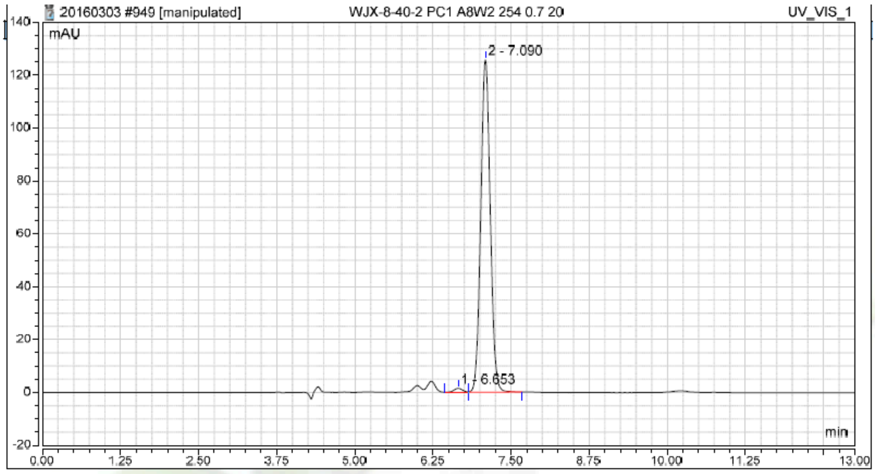

Integration Results				
No.	Retention Time min	Area mAU*min	Height mAU	Relative Area %
1	6.653	0.222	1.435	0.99
2	7.090	22.120	125.734	99.01
**Total:**		**22.342**	**127.169**	**100.00**

Figure S97. The HPLC data of **2k** in Scheme 1.

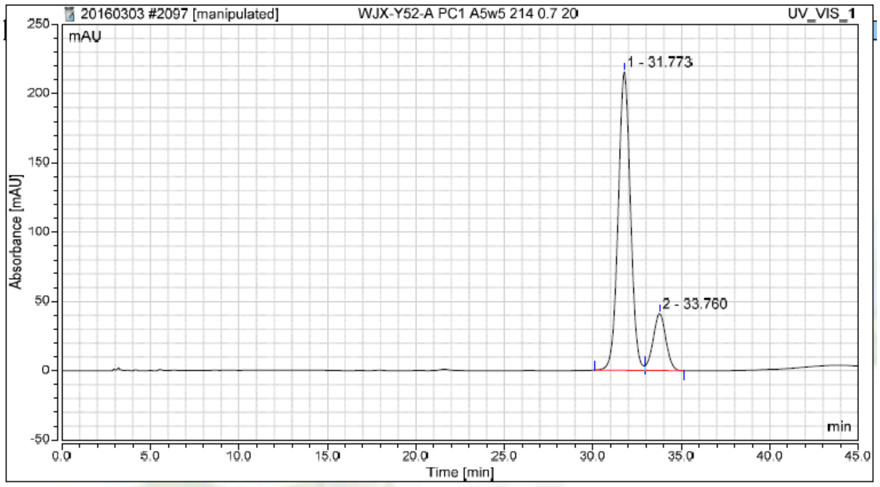

Integration Results					
No.	Retention Time min	Retention Time min	Area mAU*min	Height mAU	Relative Area %
1	32	31.773	172.983	215.444	83.44
2	34	33.760	34.340	41.234	16.56
Total:		207.323	256.678	100.00	

Figure S98. The HPLC data of **1al** in Scheme 1.

a

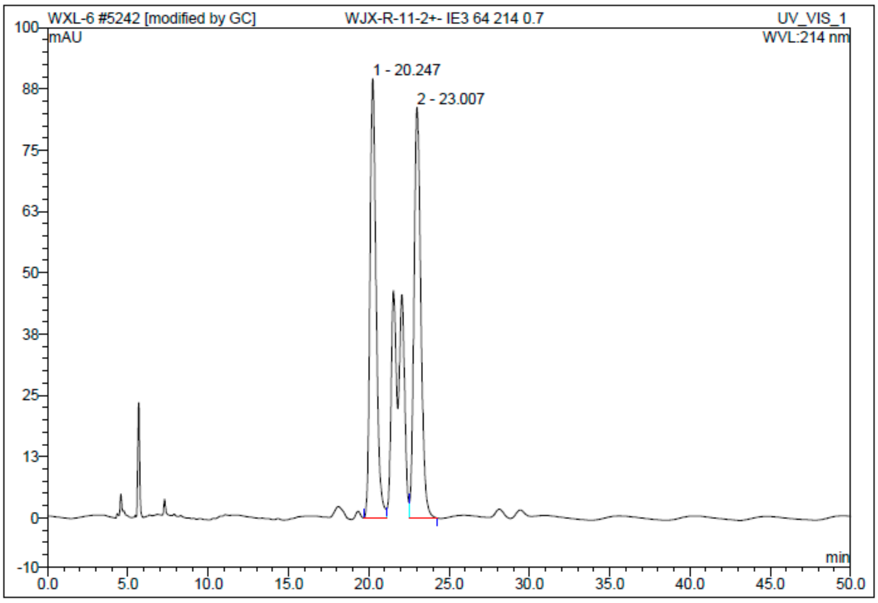

No.	Ret.Time min	Peak Name	Height mAU	Area mAU*min	Rel.Area %	Amount	Type
1	20.25	n.a.	89.565	40.034	49.01	n.a.	BM *
2	23.01	n.a.	83.768	41.657	50.99	n.a.	MB*
Total:			173.334	81.691	100.00	0.000	

b

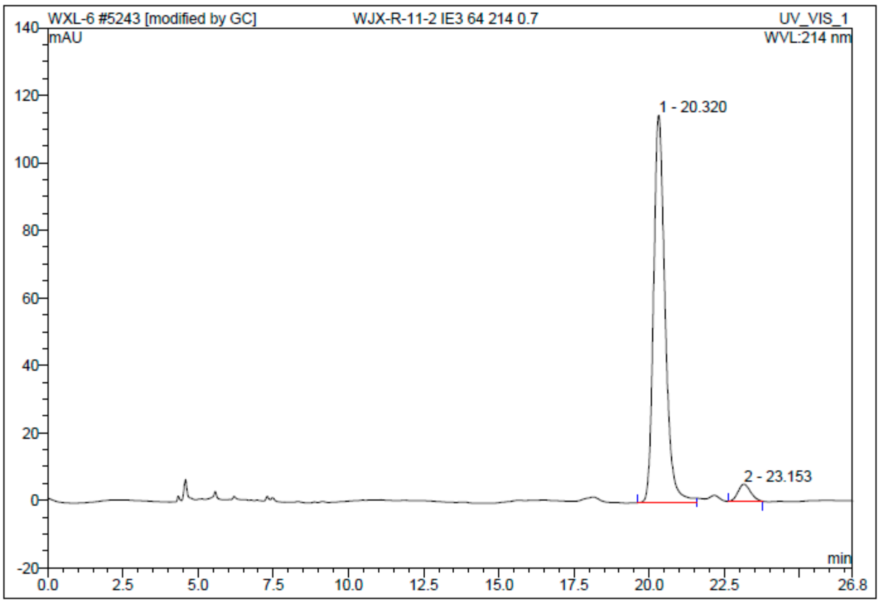

No.	Ret.Time min	Peak Name	Height mAU	Area mAU*min	Rel.Area %	Amount	Type
1	20.32	n.a.	114.805	51.004	95.44	n.a.	BM *
2	23.15	n.a.	5.044	2.435	4.56	n.a.	BMB*
Total:			119.849	53.439	100.00	0.000	

Figure S99. The HPLC data of **2l** in Scheme 1.

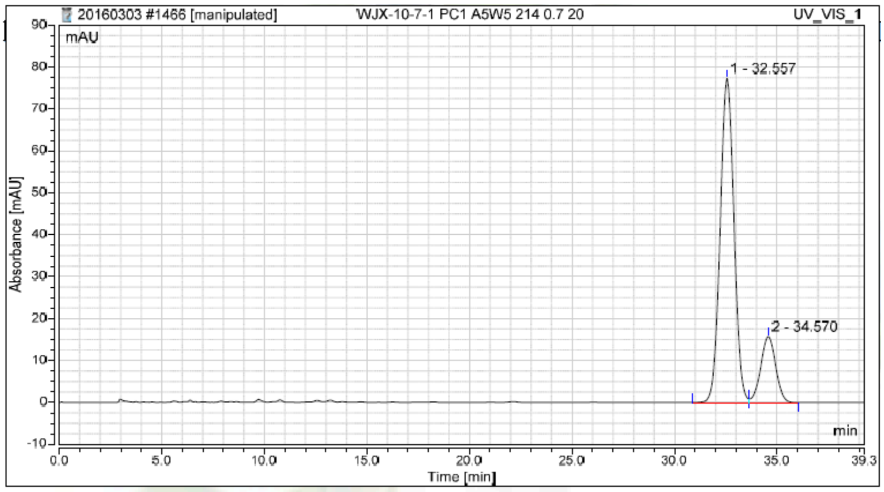

Integration Results					
No.	Retention Time min	Retention Time min	Area mAU*min	Height mAU	Relative Area %
1	33	32.557	60.389	77.338	81.94
2	35	34.570	13.309	15.781	18.06
Total:		73.699	93.119	100.00	

Figure S100. The HPLC data of **1am** in Scheme 1.

a

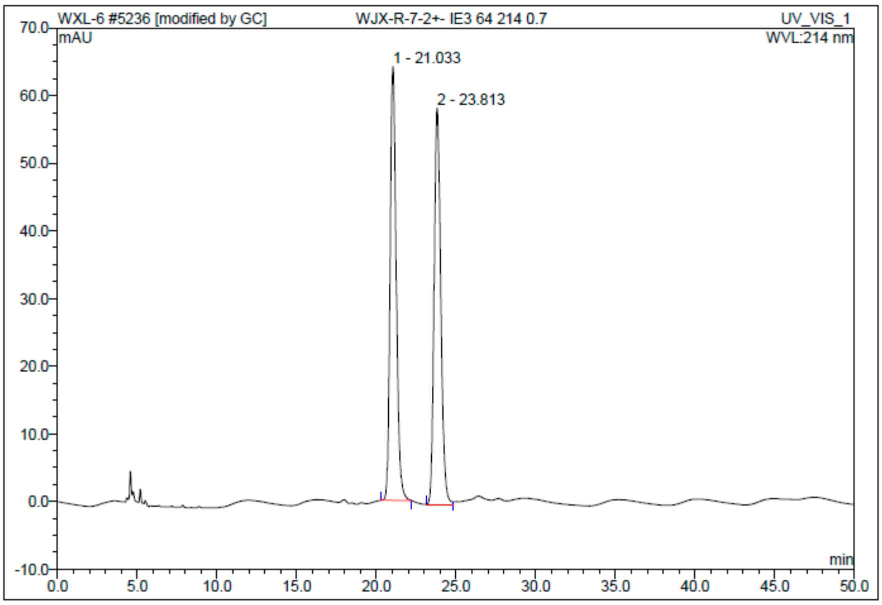

No.	Ret.Time min	Peak Name	Height mAU	Area mAU*min	Rel.Area %	Amount	Type
1	21.03	n.a.	64.158	28.490	50.06	n.a.	BMB*
2	23.81	n.a.	58.625	28.423	49.94	n.a.	BM *
Total:			122.783	56.912	100.00	0.000	

b

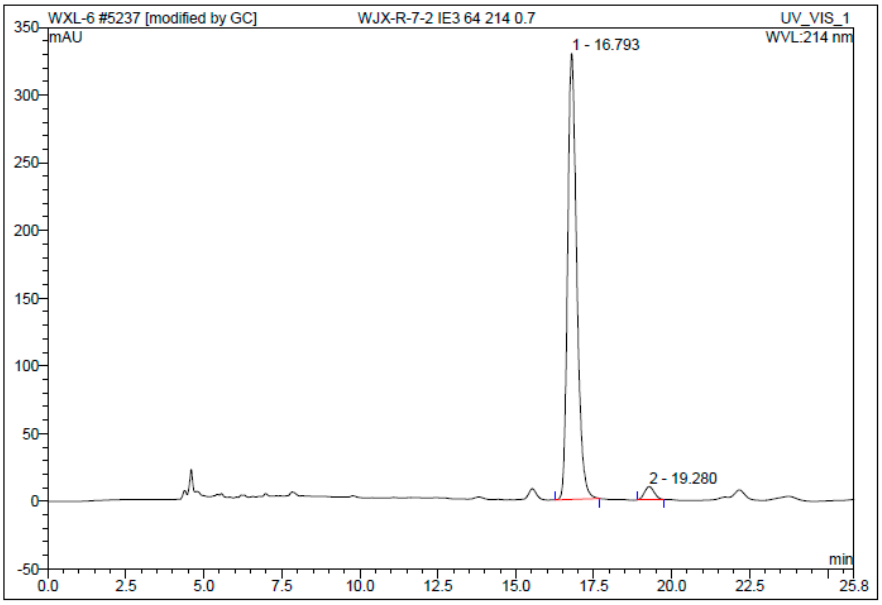

No.	Ret.Time min	Peak Name	Height mAU	Area mAU*min	Rel.Area %	Amount	Type
1	16.79	n.a.	329.385	111.937	97.03	n.a.	BMB*
2	19.28	n.a.	9.564	3.429	2.97	n.a.	BMB*
Total:			338.949	115.366	100.00	0.000	

Figure S101. The HPLC data of **2m** in Scheme 1.

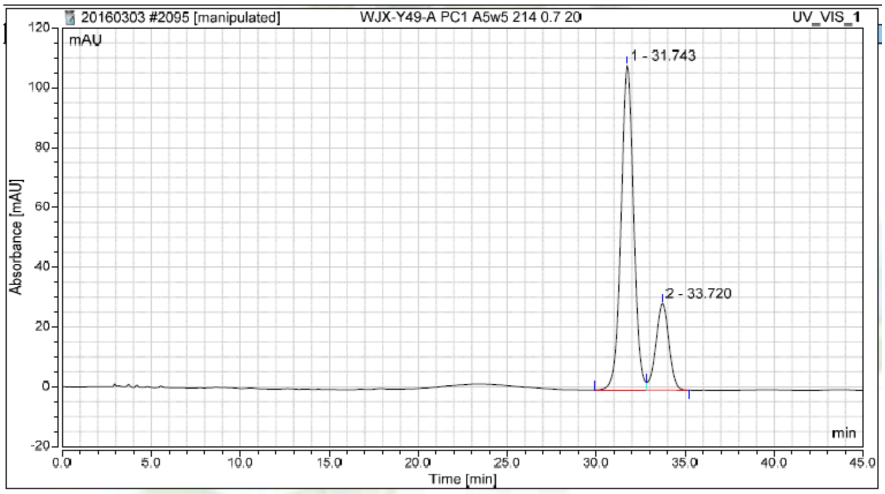

Integration Results					
No.	Retention Time min	Retention Time min	Area mAU*min	Height mAU	Relative Area %
1	32	31.743	88.880	108.647	78.27
2	34	33.720	24.674	29.095	21.73
Total:		113.554	137.742	100.00	

Figure S102. The HPLC data of **1am** in Scheme 1.

a

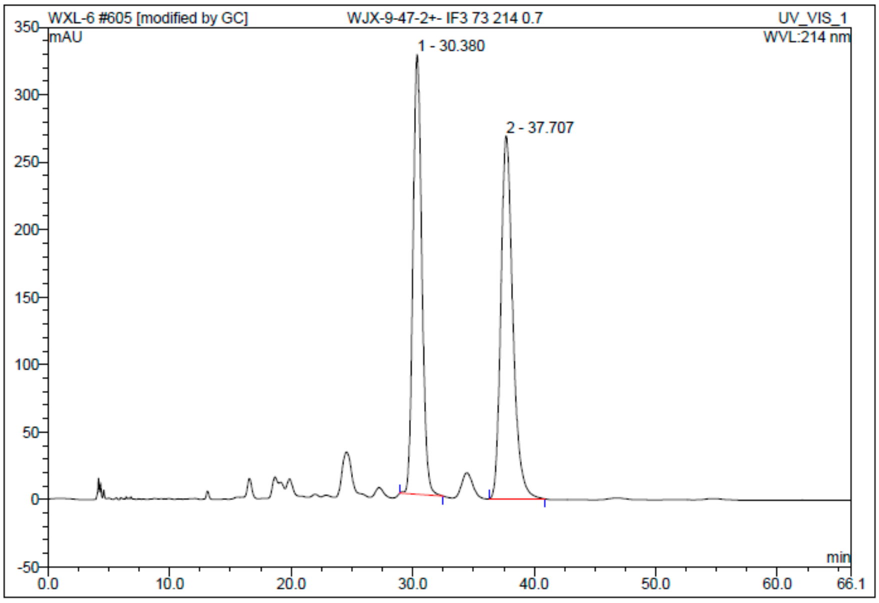

No.	Ret.Time min	Peak Name	Height mAU	Area mAU*min	Rel.Area %	Amount	Type
1	30.38	n.a.	325.972	275.270	47.24	n.a.	BMB*
2	37.71	n.a.	269.212	307.494	52.76	n.a.	BMB*
Total:			595.184	582.764	100.00	0.000	

b

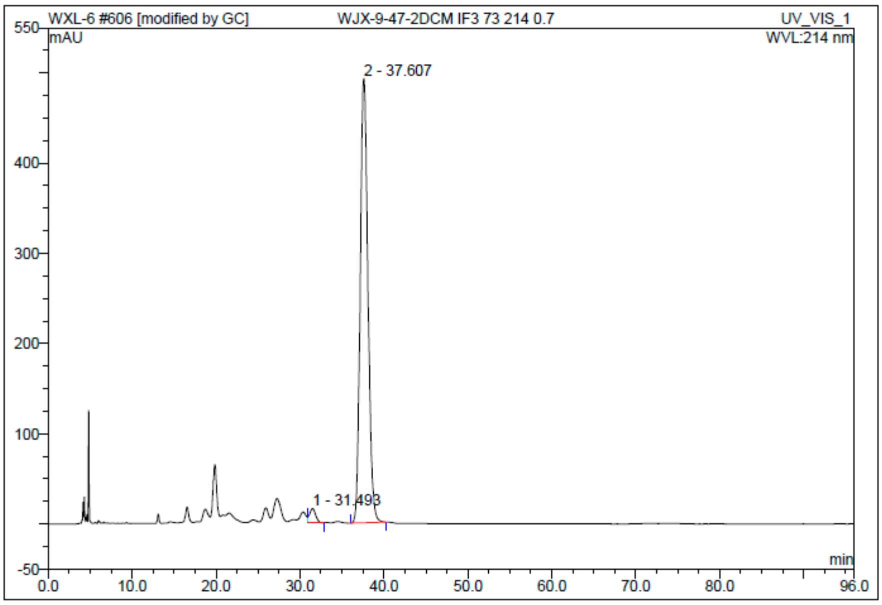

No.	Ret.Time min	Peak Name	Height mAU	Area mAU*min	Rel.Area %	Amount	Type
1	31.49	n.a.	15.529	13.612	2.53	n.a.	MB*
2	37.61	n.a.	492.445	524.468	97.47	n.a.	BMB*
Total:			507.974	538.081	100.00	0.000	

Figure S103. The HPLC data of **2n** in Scheme 1.

6. The HPLC Data of **1o-x** and **2o-x** in Scheme 2.

a

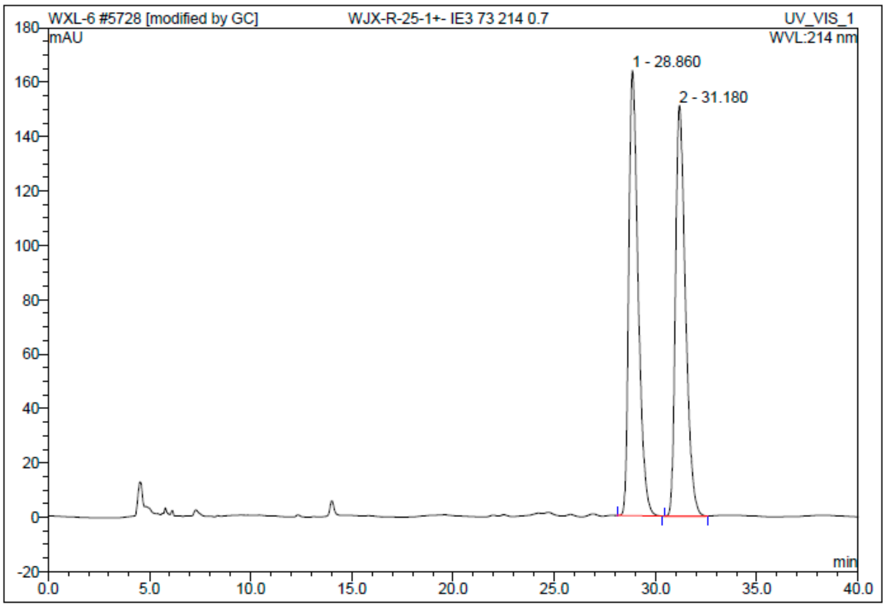

No.	Ret.Time min	Peak Name	Height mAU	Area mAU*min	Rel.Area %	Amount	Type
1	28.86	n.a.	163.779	87.964	50.29	n.a.	BMB
2	31.18	n.a.	151.111	86.960	49.71	n.a.	BMB
Total:			314.890	174.925	100.00	0.000	

b

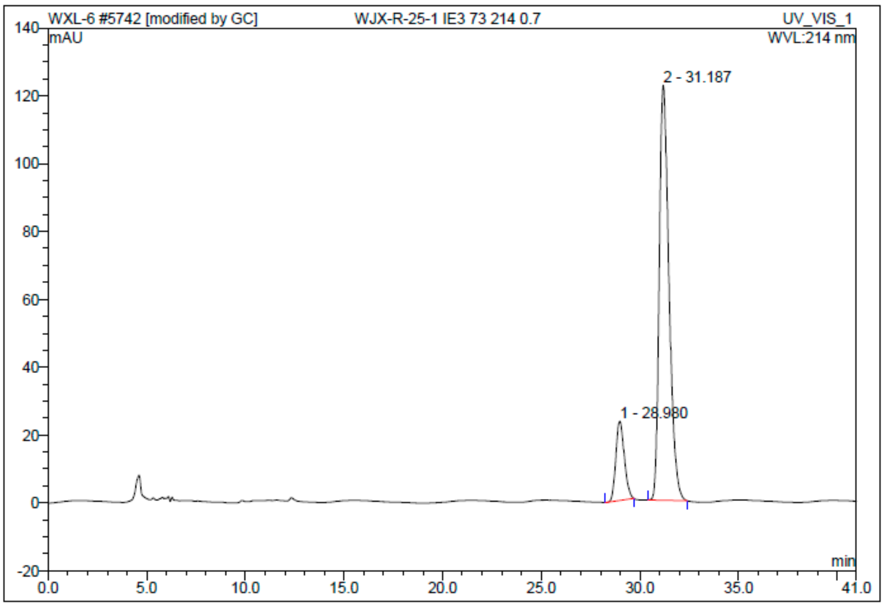

No.	Ret.Time min	Peak Name	Height mAU	Area mAU*min	Rel.Area %	Amount	Type
1	28.98	n.a.	23.381	11.660	14.31	n.a.	BMB*
2	31.19	n.a.	122.344	69.838	85.69	n.a.	BMB*
Total:			145.725	81.498	100.00	0.000	

Figure S104. The HPLC data of **1o** in Scheme 2.

a

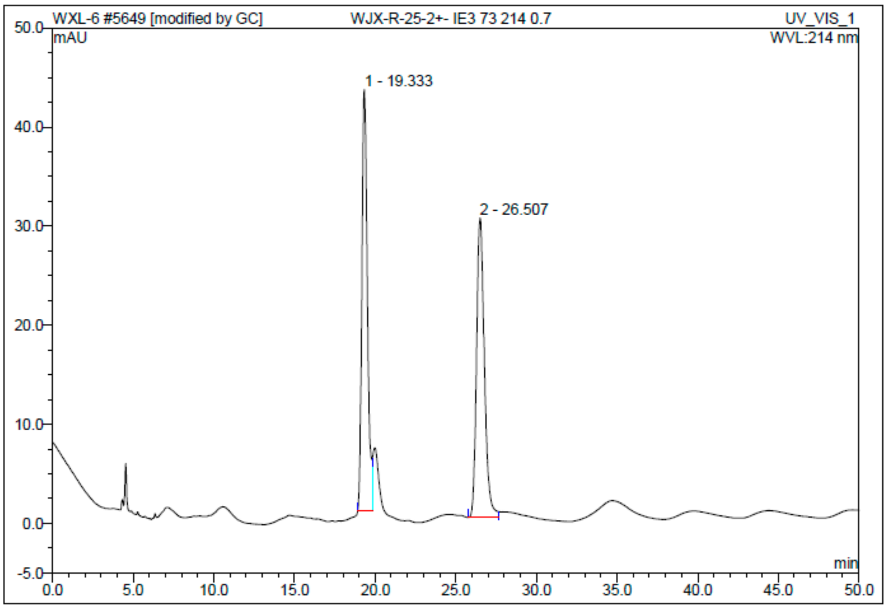

No.	Ret.Time min	Peak Name	Height mAU	Area mAU*min	Rel.Area %	Amount	Type
1	19.33	n.a.	42.546	16.795	49.61	n.a.	BM *
2	26.51	n.a.	30.215	17.061	50.39	n.a.	BM *
Total:			72.761	33.855	100.00	0.000	

b

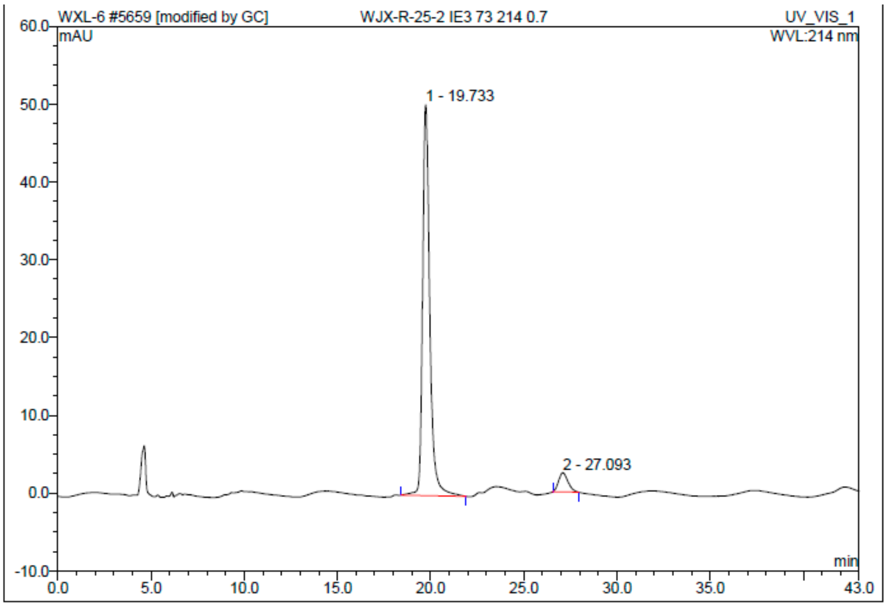

No.	Ret.Time min	Peak Name	Height mAU	Area mAU*min	Rel.Area %	Amount	Type
1	19.73	n.a.	50.233	22.331	93.90	n.a.	BMB*
2	27.09	n.a.	2.477	1.450	6.10	n.a.	BMB*
Total:			52.710	23.781	100.00	0.000	

Figure S105. The HPLC data of **2o** in Scheme 2.

a

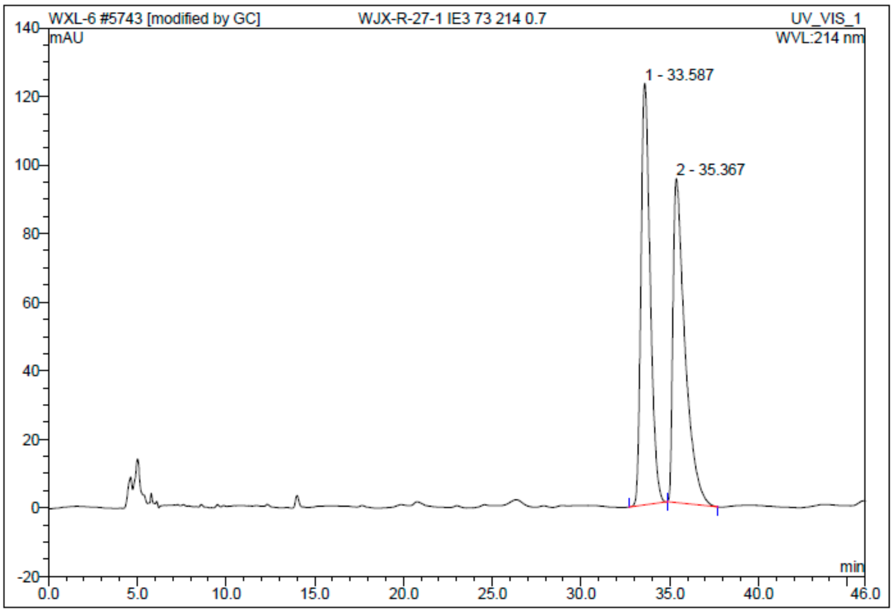

No.	Ret.Time min	Peak Name	Height mAU	Area mAU*min	Rel.Area %	Amount	Type
1	33.59	n.a.	123.021	74.070	49.94	n.a.	BMb*
2	35.37	n.a.	94.452	74.248	50.06	n.a.	bMB*
Total:			217.473	148.319	100.00	0.000	

b

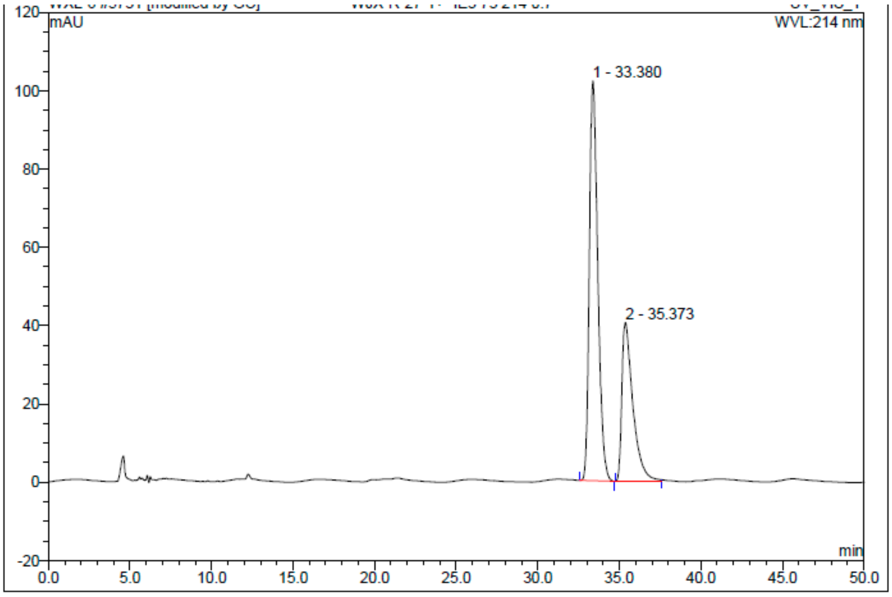

No.	Ret.Time min	Peak Name	Height mAU	Area mAU*min	Rel.Area %	Amount	Type
1	33.38	n.a.	102.141	61.432	65.88	n.a.	BMB
2	35.37	n.a.	40.562	31.810	34.12	n.a.	BM *
Total:			142.702	93.243	100.00	0.000	

Figure S106. The HPLC data of **1p** in Scheme 2.

a

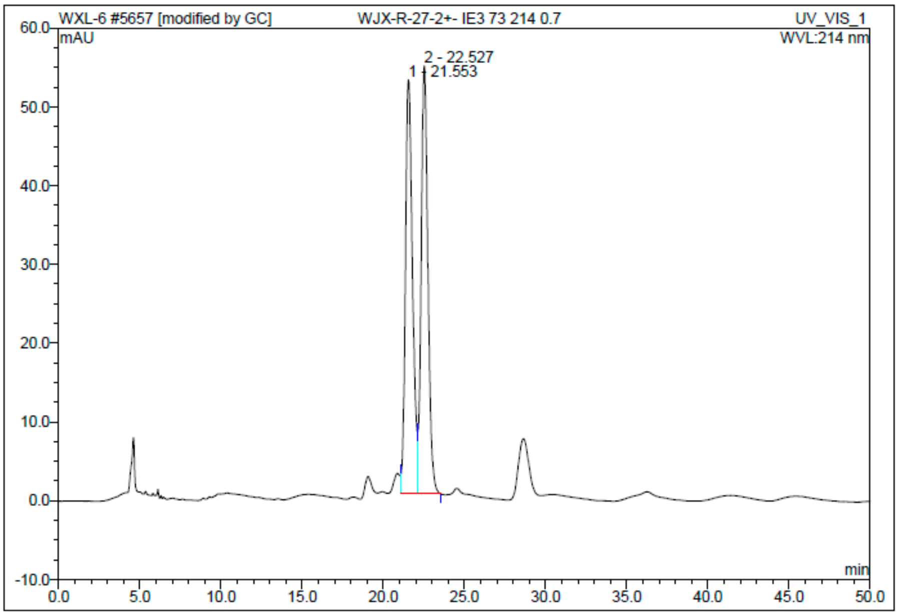

No.	Ret.Time min	Peak Name	Height mAU	Area mAU*min	Rel.Area %	Amount	Type
1	21.55	n.a.	52.571	26.529	50.32	n.a.	M *
2	22.53	n.a.	54.338	26.193	49.68	n.a.	MB*
Total:			106.909	52.722	100.00	0.000	

b

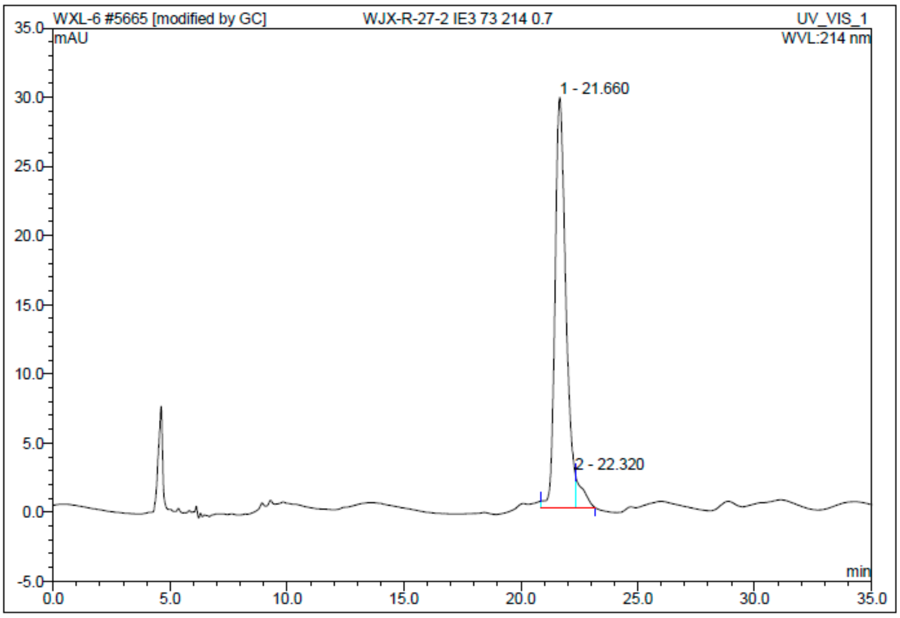

No.	Ret.Time min	Peak Name	Height mAU	Area mAU*min	Rel.Area %	Amount	Type
1	21.66	n.a.	29.663	15.813	94.76	n.a.	M *
2	22.32	n.a.	2.515	0.874	5.24	n.a.	MB*
Total:			32.178	16.687	100.00	0.000	

Figure S107. The HPLC data of **2p** in Scheme 2.

a

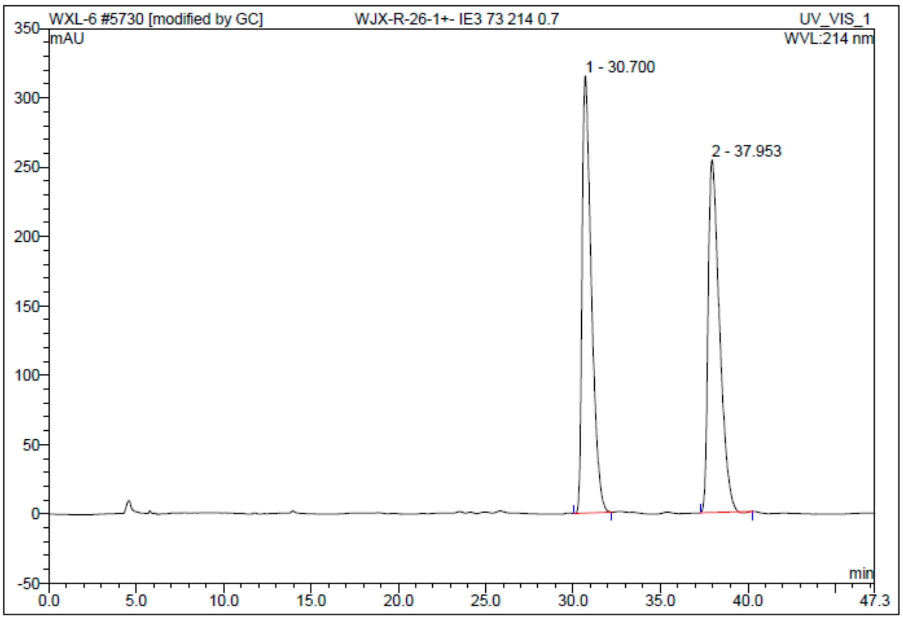

No.	Ret.Time min	Peak Name	Height mAU	Area mAU*min	Rel.Area %	Amount	Type
1	30.70	n.a.	315.435	194.329	50.23	n.a.	BMB*
2	37.95	n.a.	254.445	192.566	49.77	n.a.	BMB*
Total:			569.880	386.894	100.00	0.000	

b

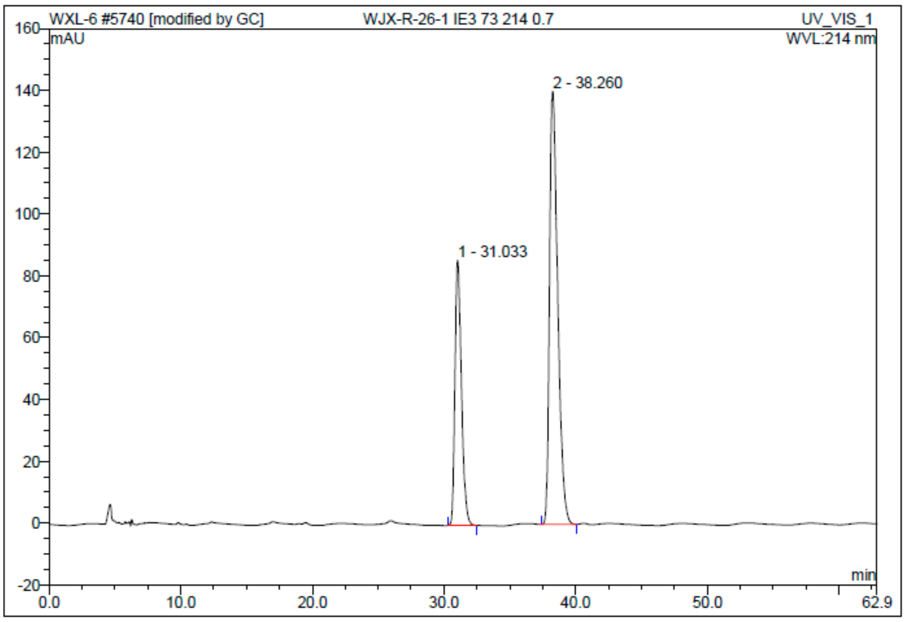

No.	Ret.Time min	Peak Name	Height mAU	Area mAU*min	Rel.Area %	Amount	Type
1	31.03	n.a.	85.813	49.050	32.58	n.a.	BMB
2	38.26	n.a.	140.193	101.491	67.42	n.a.	BMB
Total:			226.007	150.540	100.00	0.000	

Figure S108. The HPLC data of **1q** in Scheme 2.

a

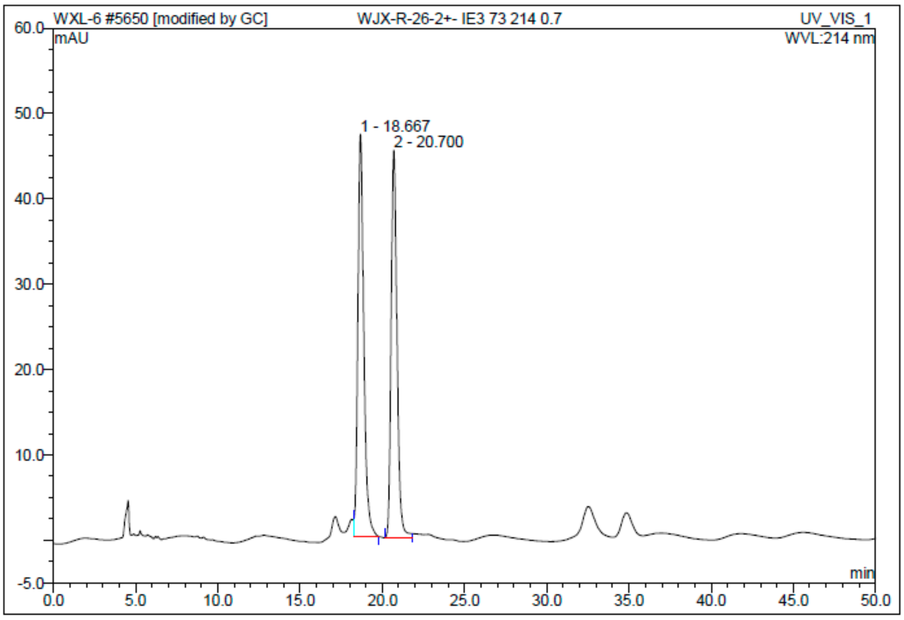

No.	Ret.Time min	Peak Name	Height mAU	Area mAU*min	Rel.Area %	Amount	Type
1	18.67	n.a.	47.138	19.796	51.27	n.a.	MB*
2	20.70	n.a.	45.426	18.812	48.73	n.a.	BM *
Total:			92.564	38.608	100.00	0.000	

b

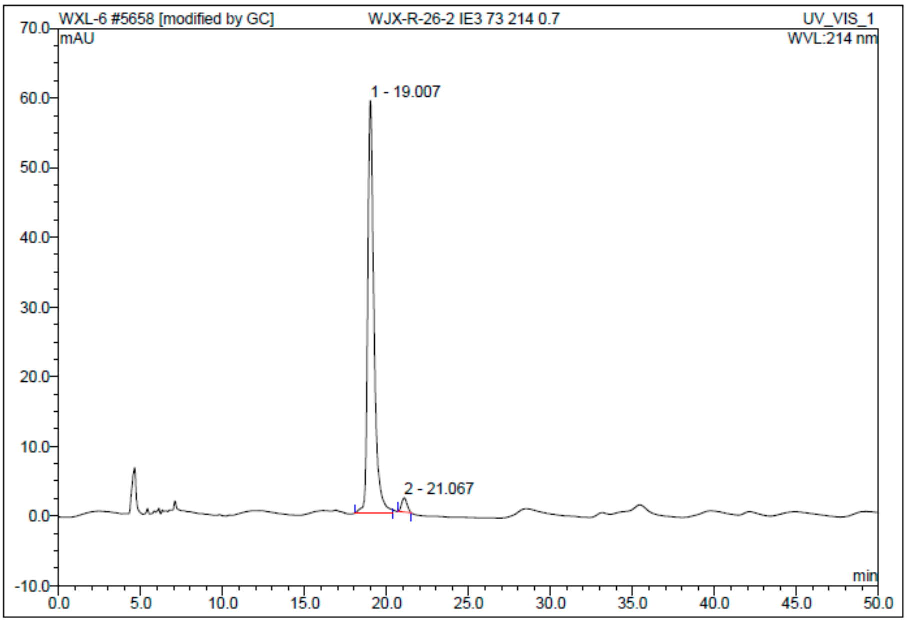

No.	Ret.Time min	Peak Name	Height mAU	Area mAU*min	Rel.Area %	Amount	Type
1	19.01	n.a.	59.220	26.439	97.16	n.a.	BM *
2	21.07	n.a.	2.037	0.773	2.84	n.a.	BMB*
Total:			61.257	27.212	100.00	0.000	

Figure S109. The HPLC data of **2q** in Scheme 2.

a

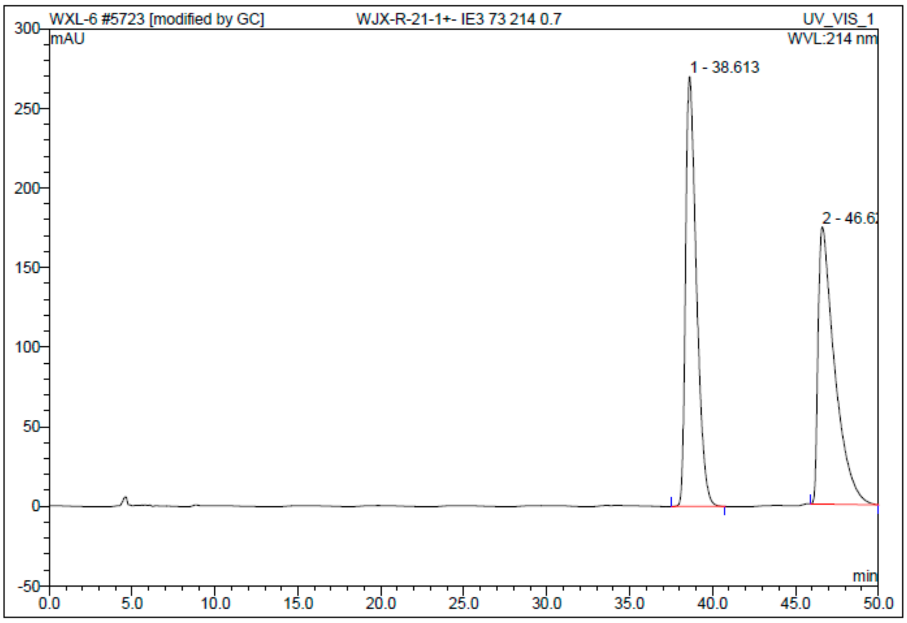

No.	Ret.Time min	Peak Name	Height mAU	Area mAU*min	Rel.Area %	Amount	Type
1	38.61	n.a.	270.072	206.010	50.44	n.a.	BMB
2	46.63	n.a.	173.979	202.446	49.56	n.a.	BMB
Total:			444.052	408.456	100.00	0.000	

b

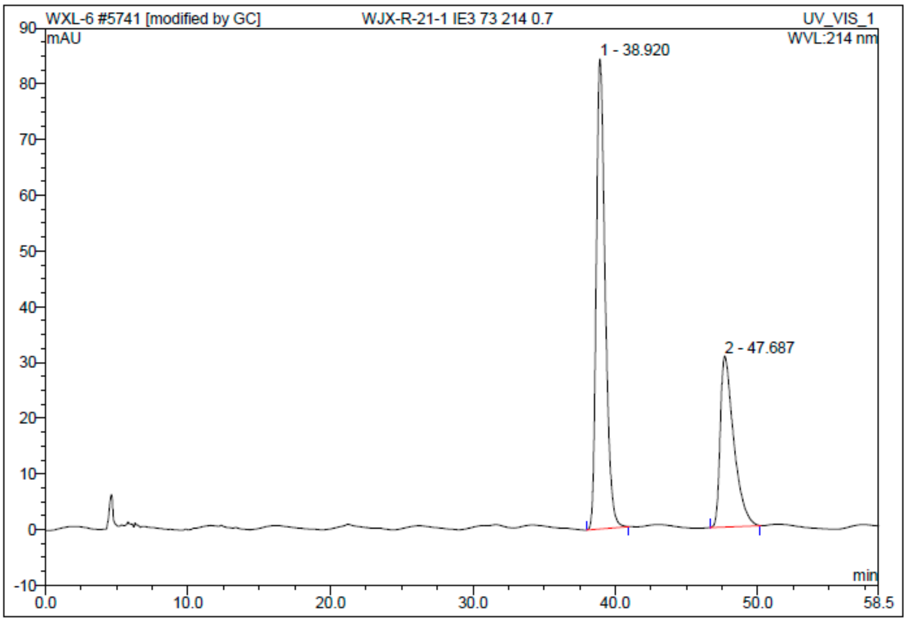

No.	Ret.Time min	Peak Name	Height mAU	Area mAU*min	Rel.Area %	Amount	Type
1	38.92	n.a.	84.369	61.380	64.47	n.a.	BMB
2	47.69	n.a.	30.701	33.828	35.53	n.a.	BMB
Total:			115.070	95.208	100.00	0.000	

Figure S110. The HPLC data of **1r** in Scheme 2.

a

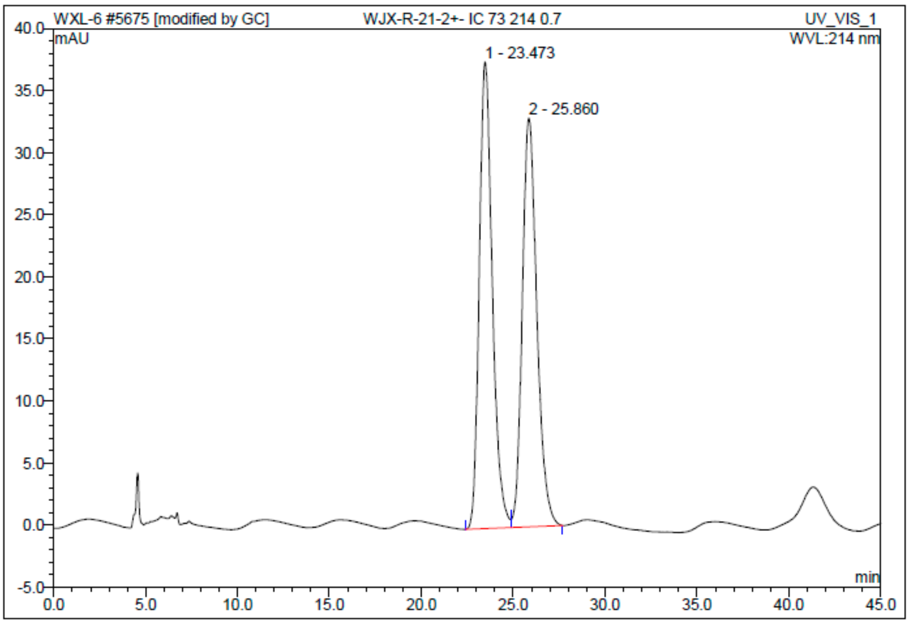

No.	Ret.Time min	Peak Name	Height mAU	Area mAU*min	Rel.Area %	Amount	Type
1	23.47	n.a.	37.587	31.001	50.58	n.a.	BM
2	25.86	n.a.	32.936	30.292	49.42	n.a.	MB
Total:			70.523	61.294	100.00	0.000	

b

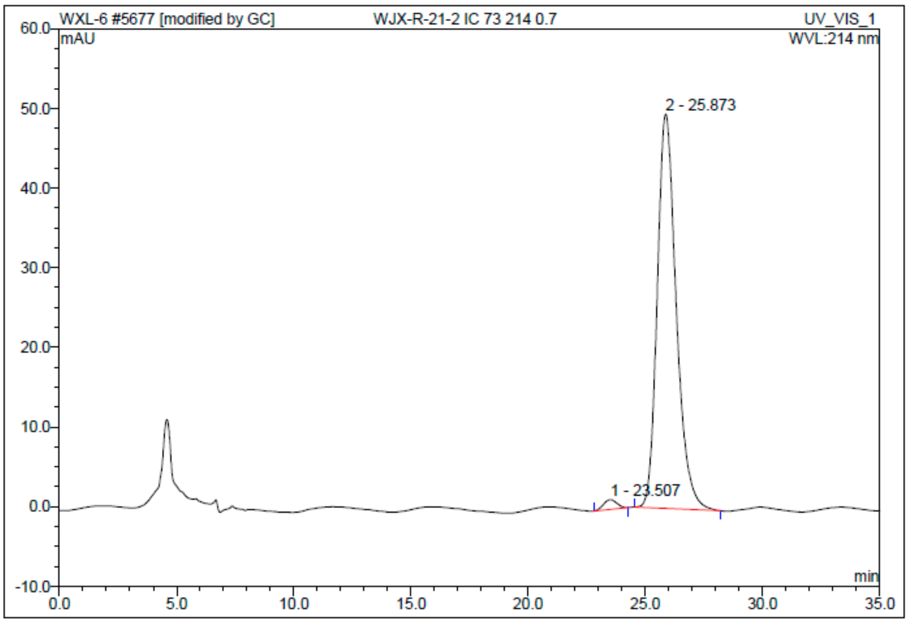

No.	Ret.Time min	Peak Name	Height mAU	Area mAU*min	Rel.Area %	Amount	Type
1	23.51	n.a.	1.225	0.802	1.70	n.a.	BMB
2	25.87	n.a.	49.517	46.435	98.30	n.a.	BMB
Total:			50.742	47.238	100.00	0.000	

Figure S111. The HPLC data of **2r** in Scheme 2.

a

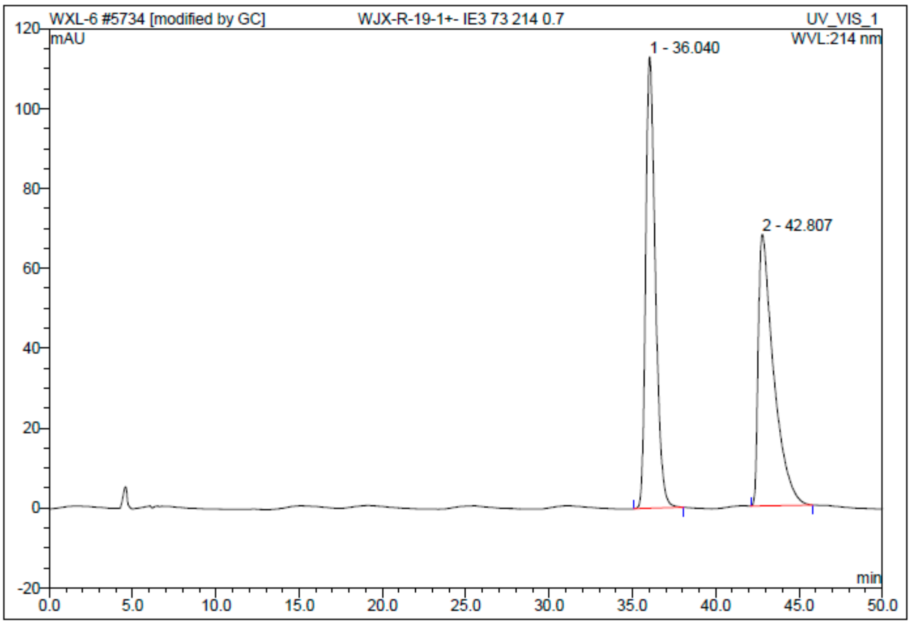

No.	Ret.Time min	Peak Name	Height mAU	Area mAU*min	Rel.Area %	Amount	Type
1	36.04	n.a.	113.024	76.115	51.10	n.a.	BMB
2	42.81	n.a.	67.961	72.844	48.90	n.a.	BMB
Total:			180.985	148.959	100.00	0.000	

b

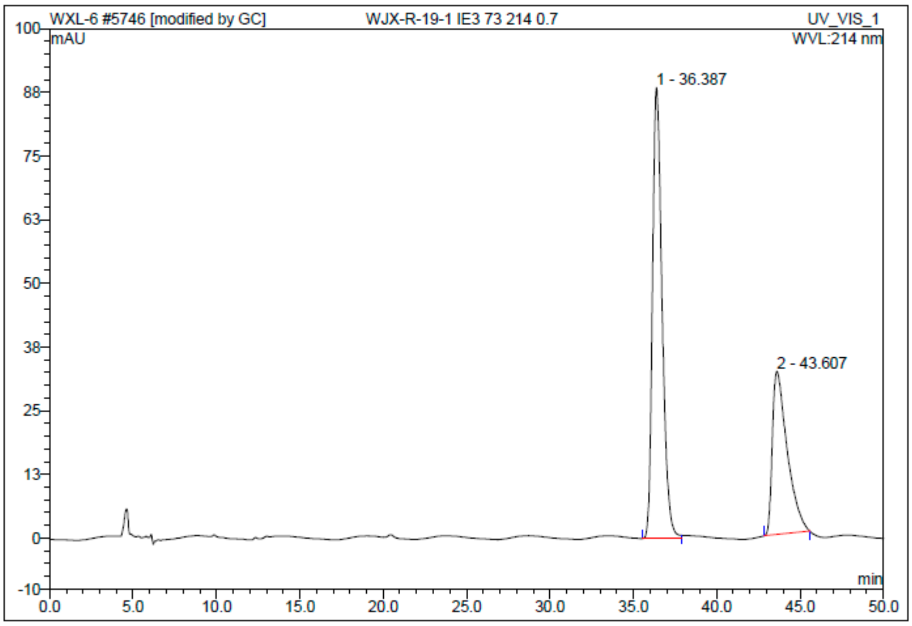

No.	Ret.Time min	Peak Name	Height mAU	Area mAU*min	Rel.Area %	Amount	Type
1	36.39	n.a.	88.542	59.336	64.22	n.a.	BM *
2	43.61	n.a.	32.028	33.058	35.78	n.a.	BMB*
Total:			120.570	92.394	100.00	0.000	

Figure S112. The HPLC data of **1s** in Scheme 2.

a

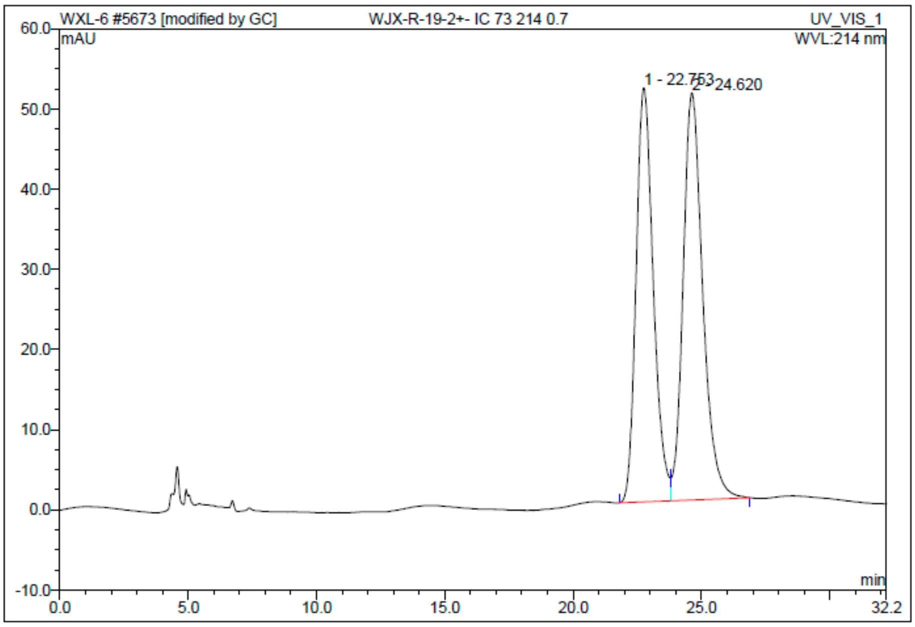

No.	Ret.Time min	Peak Name	Height mAU	Area mAU*min	Rel.Area %	Amount	Type
1	22.75	n.a.	51.685	40.111	47.10	n.a.	BM
2	24.62	n.a.	50.813	45.048	52.90	n.a.	MB
Total:			102.498	85.159	100.00	0.000	

b

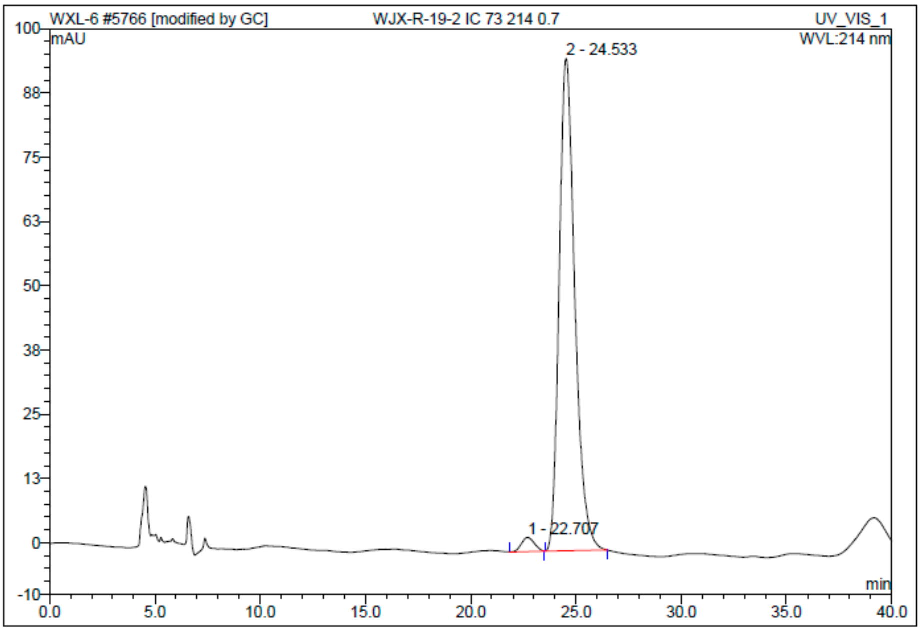

No.	Ret.Time min	Peak Name	Height mAU	Area mAU*min	Rel.Area %	Amount	Type
1	22.71	n.a.	2.760	1.969	2.33	n.a.	BMB*
2	24.53	n.a.	95.755	82.712	97.67	n.a.	BMB*
Total:			98.514	84.680	100.00	0.000	

Figure S113. The HPLC data of **2s** in Scheme 2.

a

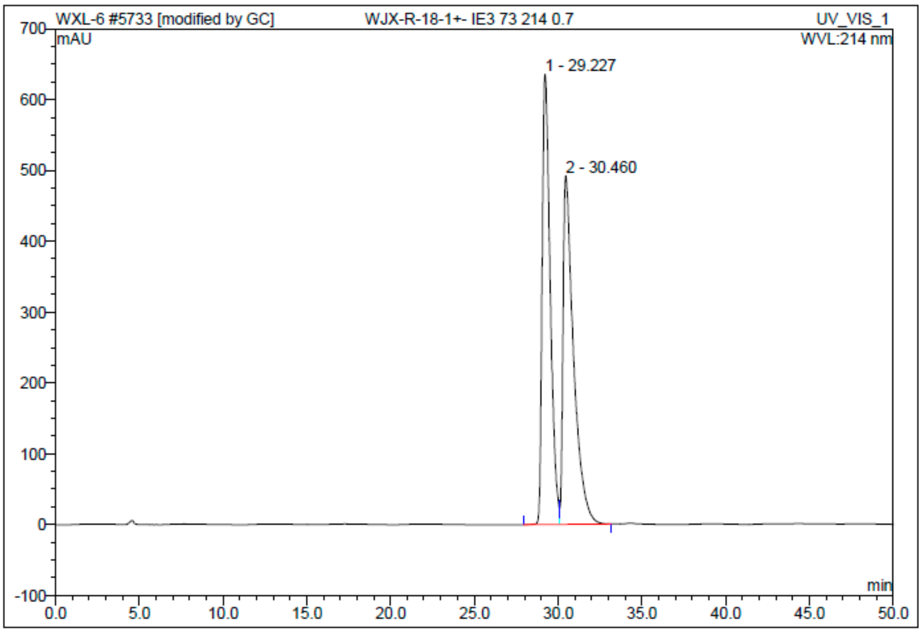

No.	Ret.Time min	Peak Name	Height mAU	Area mAU*min	Rel.Area %	Amount	Type
1	29.23	n.a.	635.628	353.020	49.61	n.a.	BM
2	30.46	n.a.	491.814	358.576	50.39	n.a.	MB
Total:			1127.442	711.596	100.00	0.000	

b

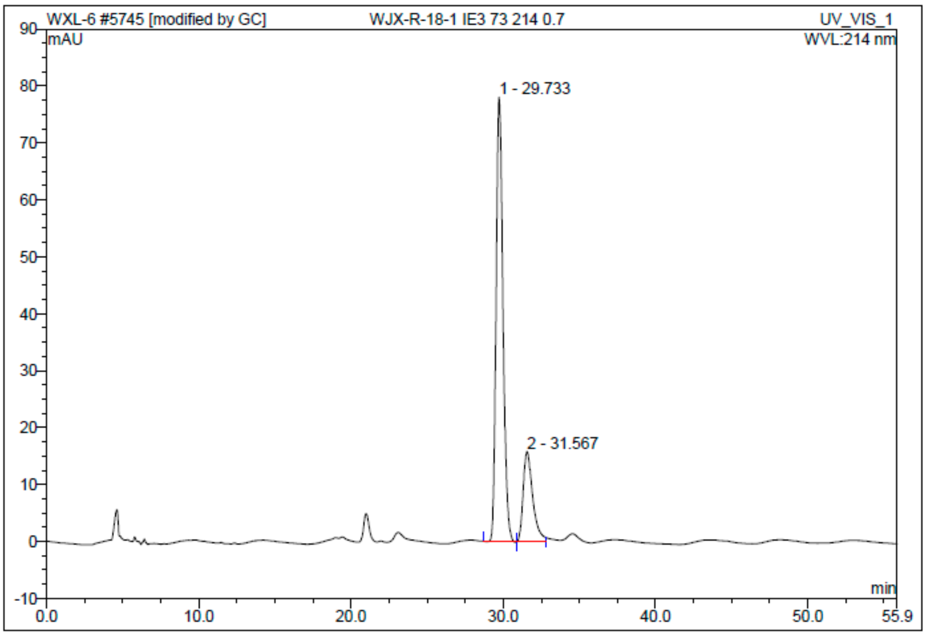

No.	Ret.Time min	Peak Name	Height mAU	Area mAU*min	Rel.Area %	Amount	Type
1	29.73	n.a.	78.146	40.797	77.83	n.a.	Mb*
2	31.57	n.a.	15.891	11.618	22.17	n.a.	bM *
Total:			94.036	52.414	100.00	0.000	

Figure S114. The HPLC data of **1t** in Scheme 2.

a

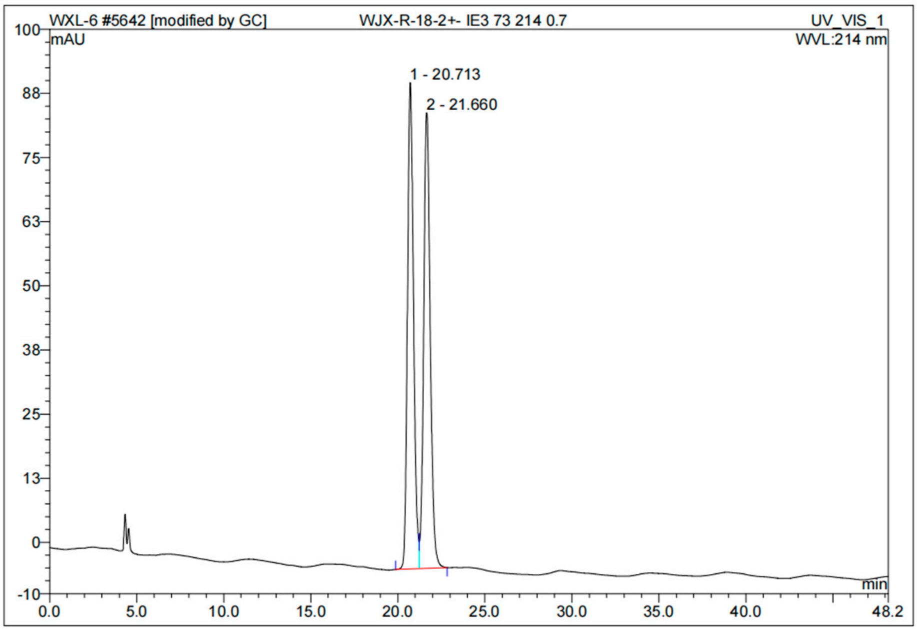

No.	Ret.Time min	Peak Name	Height mAU	Area mAU*min	Rel.Area %	Amount	Type
1	20.71	n.a.	94.790	38.067	49.71	n.a.	BM
2	21.66	n.a.	88.790	38.510	50.29	n.a.	MB
Total:			183.580	76.576	100.00	0.000	

b

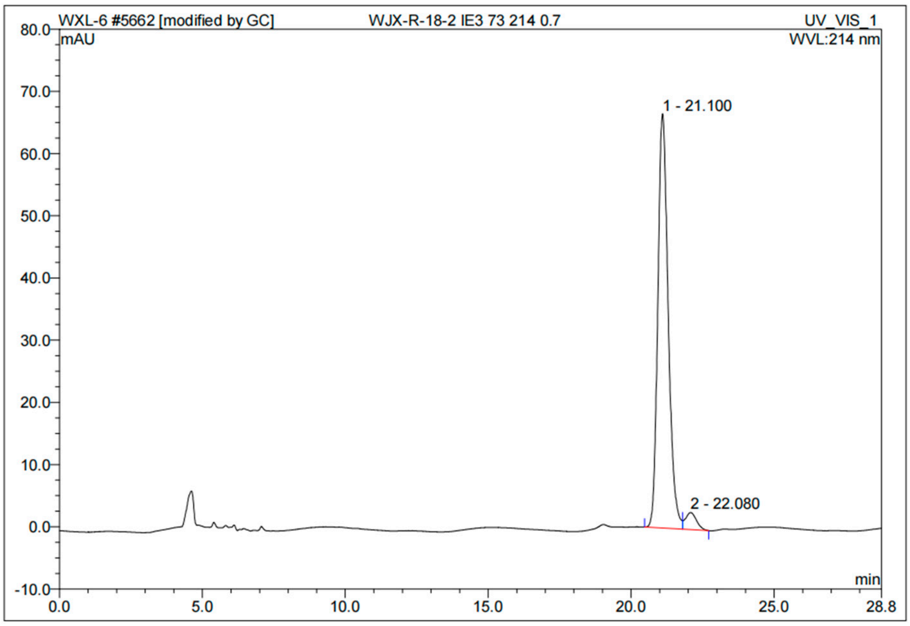

No.	Ret.Time min	Peak Name	Height mAU	Area mAU*min	Rel.Area %	Amount	Type
1	21.10	n.a.	66.633	27.982	95.77	n.a.	BM
2	22.08	n.a.	2.746	1.236	4.23	n.a.	MB
Total:			69.379	29.218	100.00	0.000	

Figure S115. The HPLC data of **2t** in Scheme 2.

a

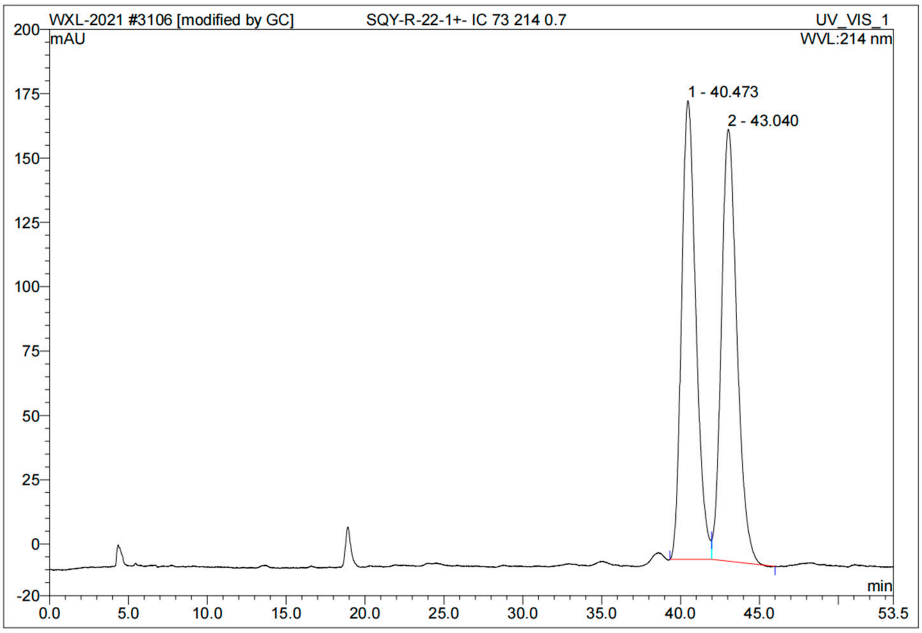

No.	Ret.Time min	Peak Name	Height mAU	Area mAU*min	Rel.Area %	Amount	Type
1	40.47	n.a.	178.262	188.165	49.45	n.a.	BM *
2	43.04	n.a.	167.891	192.340	50.55	n.a.	MB*
Total:			346.153	380.505	100.00	0.000	

b

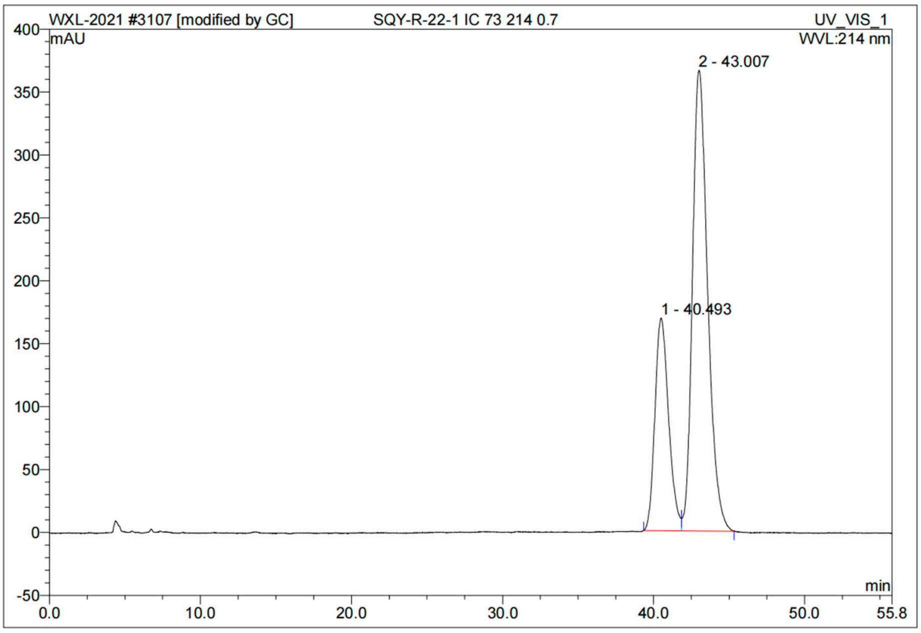

No.	Ret.Time min	Peak Name	Height mAU	Area mAU*min	Rel.Area %	Amount	Type
1	40.49	n.a.	169.153	177.644	29.33	n.a.	BM
2	43.01	n.a.	366.302	427.950	70.67	n.a.	MB
Total:			535.455	605.594	100.00	0.000	

Figure S116. The HPLC data of **1u** in Scheme 2.

a

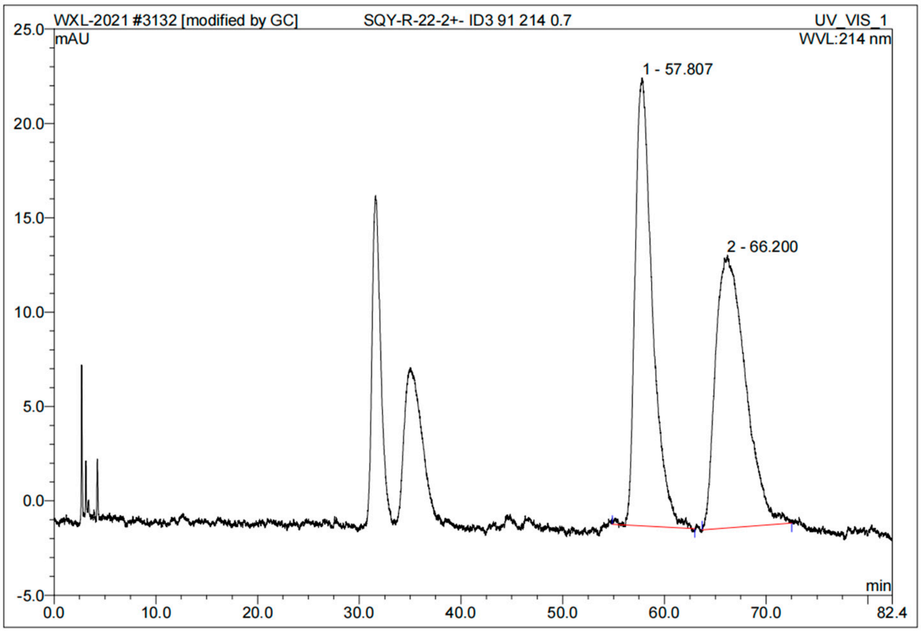

No.	Ret.Time min	Peak Name	Height mAU	Area mAU*min	Rel.Area %	Amount	Type
1	57.81	n.a.	23.736	46.860	48.73	n.a.	BMB*
2	66.20	n.a.	14.469	49.299	51.27	n.a.	BMB*
Total:			38.205	96.160	100.00	0.000	

b

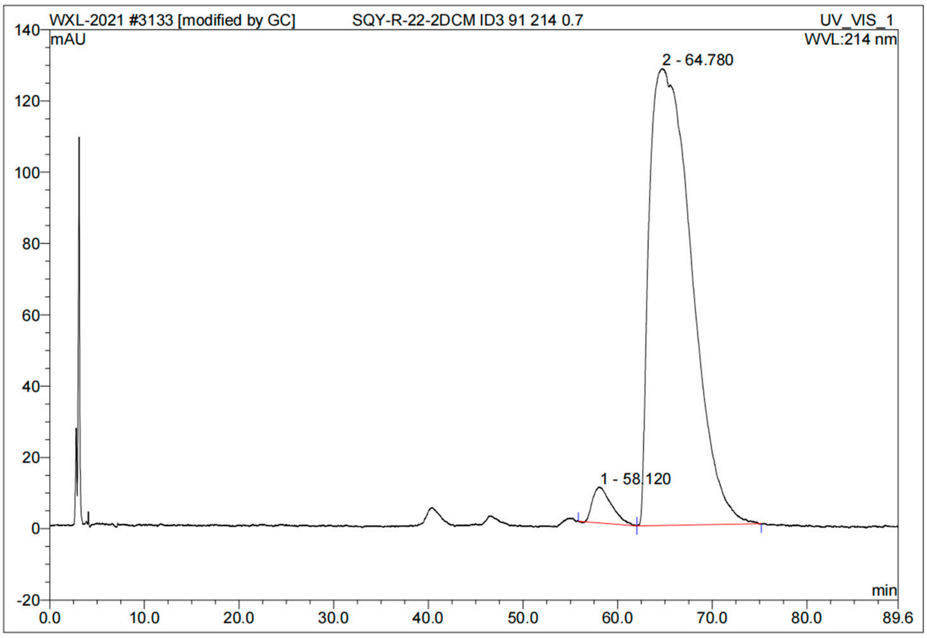

No.	Ret.Time min	Peak Name	Height mAU	Area mAU*min	Rel.Area %	Amount	Type
1	58.12	n.a.	10.069	23.204	3.39	n.a.	BMb*
2	64.78	n.a.	128.193	661.629	96.61	n.a.	bMB*
Total:			138.262	684.833	100.00	0.000	

Figure S117. The HPLC data of **2u** in Scheme 2.

a

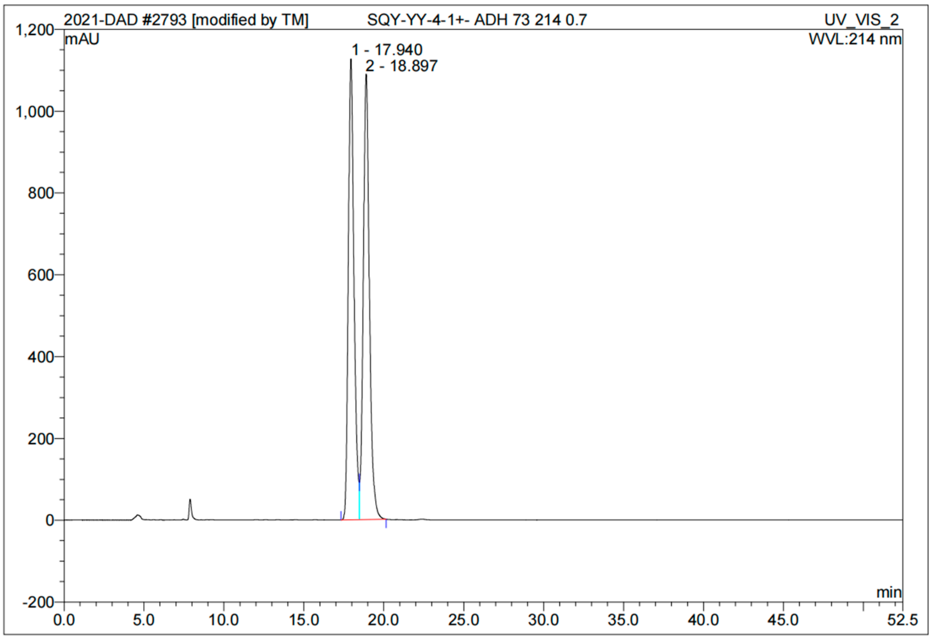

No.	Ret.Time min	Peak Name	Height mAU	Area mAU*min	Rel.Area %	Amount	Type
1	17.94	n.a.	1127.317	466.937	48.98	n.a.	BM
2	18.90	n.a.	1089.283	486.368	51.02	n.a.	MB
Total:			2216.600	953.304	100.00	0.000	

b

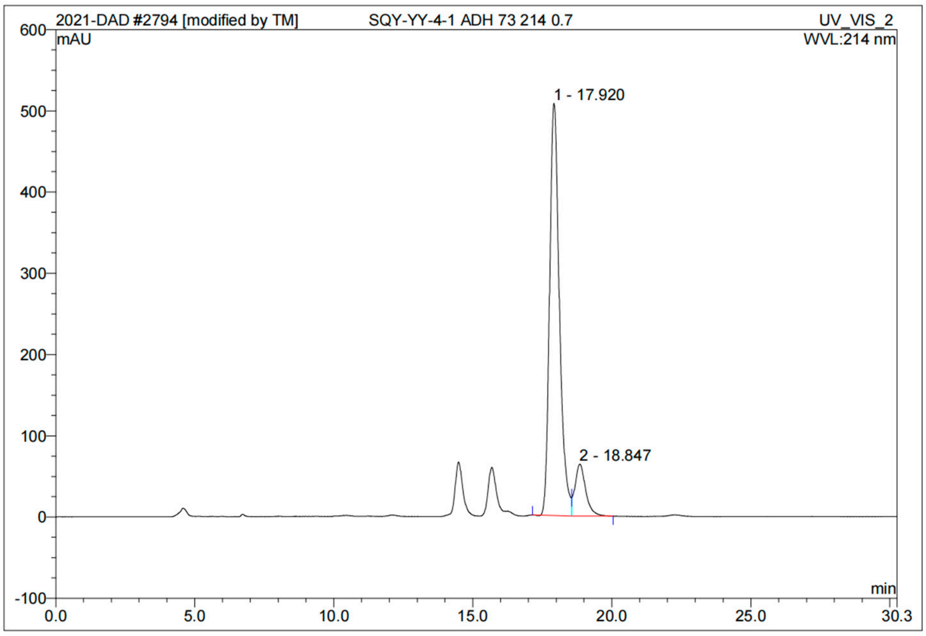

No.	Ret.Time min	Peak Name	Height mAU	Area mAU*min	Rel.Area %	Amount	Type
1	17.92	n.a.	507.757	207.452	87.80	n.a.	BM *
2	18.85	n.a.	64.114	28.813	12.20	n.a.	MB*
Total:			571.871	236.265	100.00	0.000	

Figure S118. The HPLC data of **1v** in Scheme 2.

a

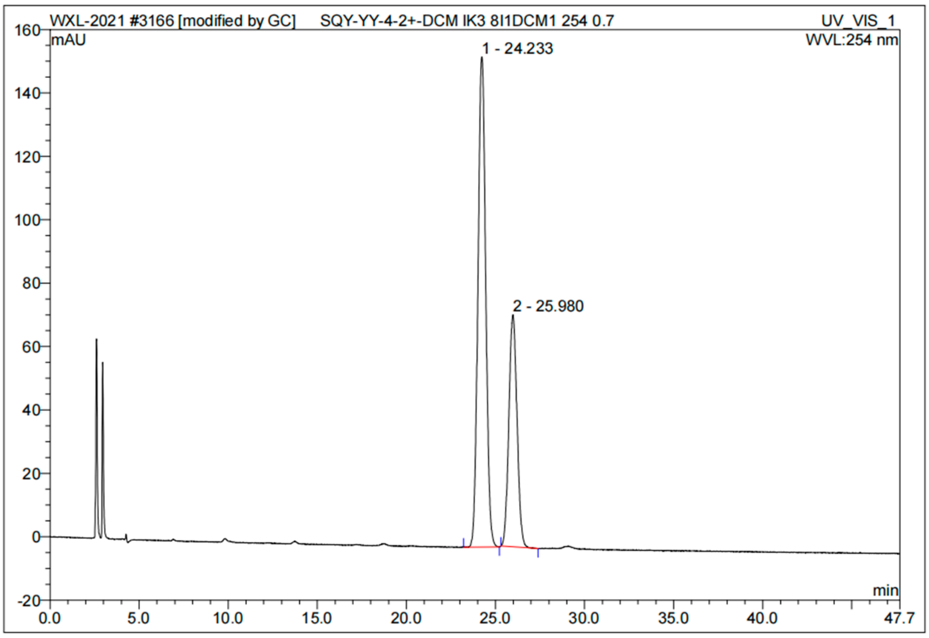

No.	Ret.Time min	Peak Name	Height mAU	Area mAU*min	Rel.Area %	Amount	Type
1	24.23	n.a.	154.699	77.890	66.66	n.a.	BMB*
2	25.98	n.a.	73.227	38.963	33.34	n.a.	BMB*
Total:			227.926	116.853	100.00	0.000	

b

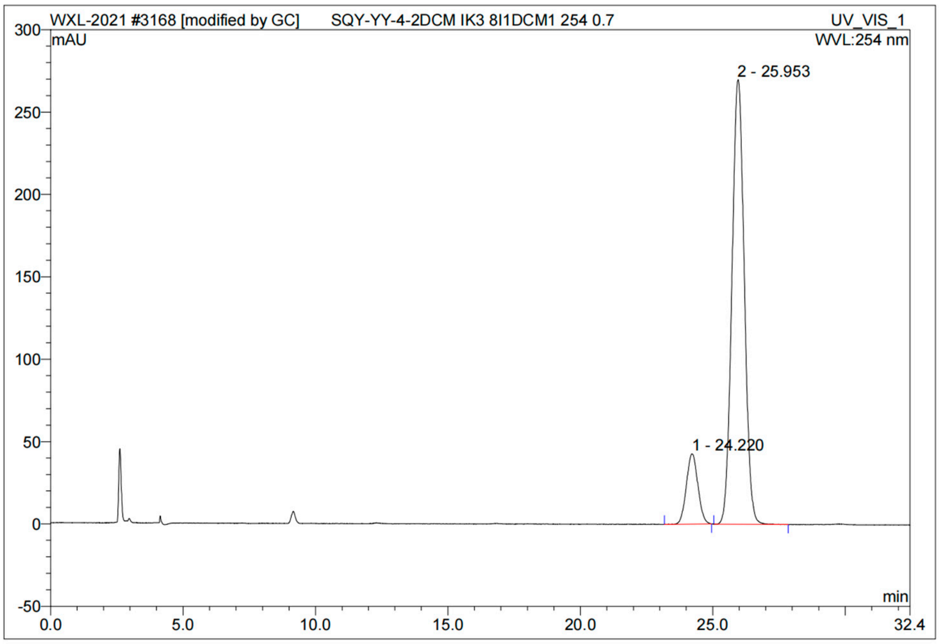

No.	Ret.Time min	Peak Name	Height mAU	Area mAU*min	Rel.Area %	Amount	Type
1	24.22	n.a.	42.802	21.470	12.82	n.a.	BMB*
2	25.95	n.a.	269.892	146.018	87.18	n.a.	BMB*
Total:			312.694	167.488	100.00	0.000	

Figure S119. The HPLC data of **2v** in Scheme 2.

a

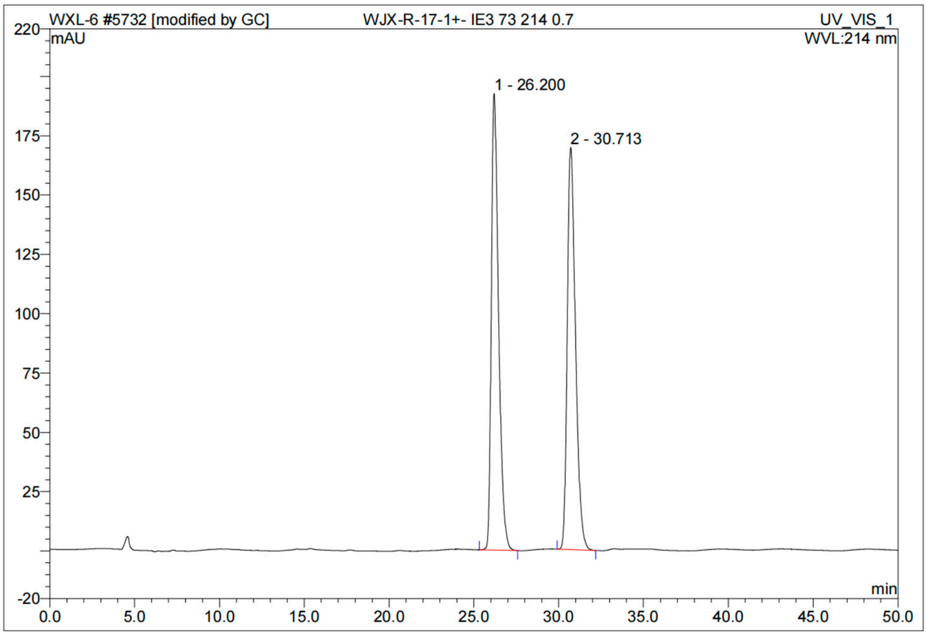

No.	Ret.Time min	Peak Name	Height mAU	Area mAU*min	Rel.Area %	Amount	Type
1	26.20	n.a.	192.547	95.390	50.01	n.a.	BMB
2	30.71	n.a.	169.622	95.350	49.99	n.a.	BMB
Total:			362.169	190.739	100.00	0.000	

b

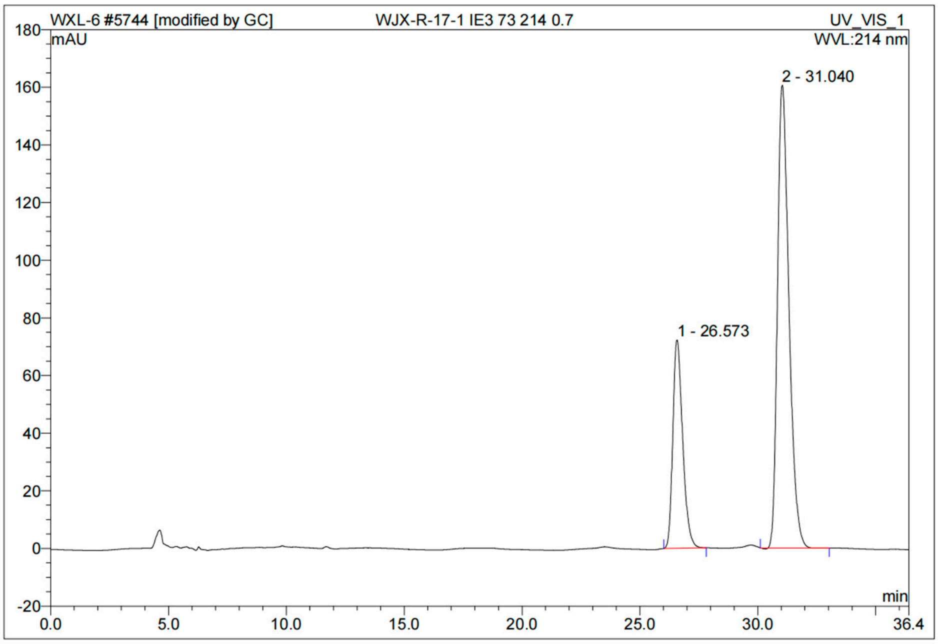

No.	Ret.Time min	Peak Name	Height mAU	Area mAU*min	Rel.Area %	Amount	Type
1	26.57	n.a.	72.262	34.965	27.91	n.a.	BMB*
2	31.04	n.a.	160.638	90.296	72.09	n.a.	BMB*
Total:			232.900	125.261	100.00	0.000	

Figure S120. The HPLC data of **1w** in Scheme 2.

a

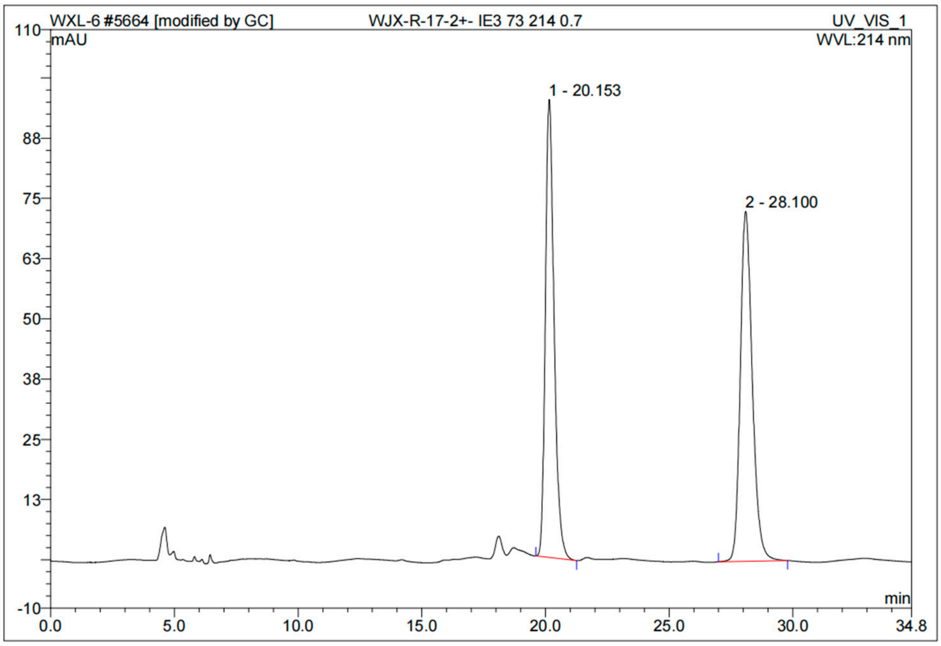

No.	Ret.Time min	Peak Name	Height mAU	Area mAU*min	Rel.Area %	Amount	Type
1	20.15	n.a.	95.080	38.738	48.64	n.a.	BMB*
2	28.10	n.a.	72.649	40.899	51.36	n.a.	BMB
Total:			167.729	79.637	100.00	0.000	

b

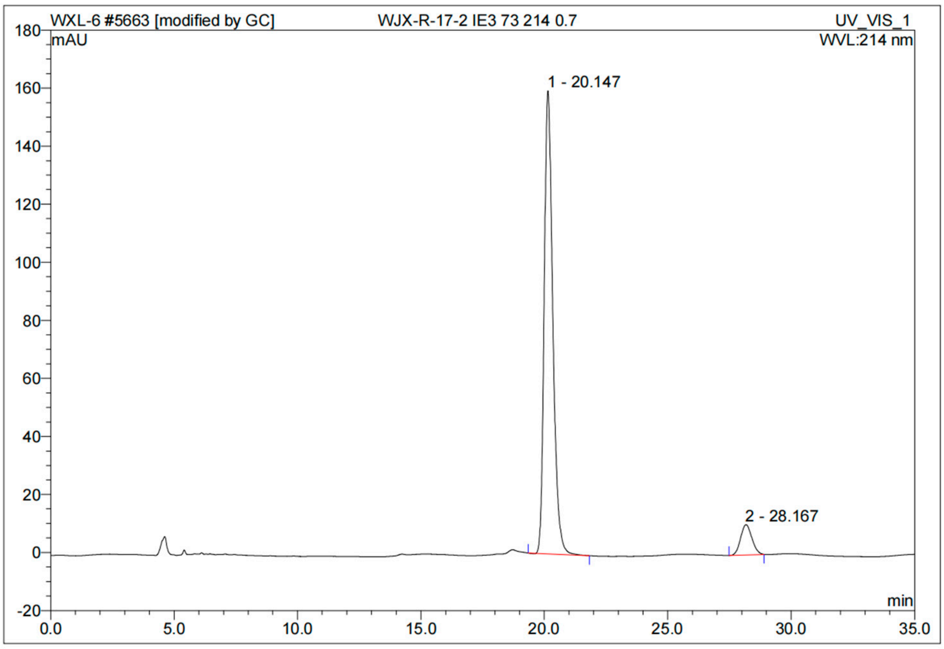

No.	Ret.Time min	Peak Name	Height mAU	Area mAU*min	Rel.Area %	Amount	Type
1	20.15	n.a.	159.625	64.527	92.09	n.a.	BMB*
2	28.17	n.a.	10.411	5.542	7.91	n.a.	BMB*
Total:			170.036	70.069	100.00	0.000	

Figure S121. The HPLC data of **2w** in Scheme 2.

a

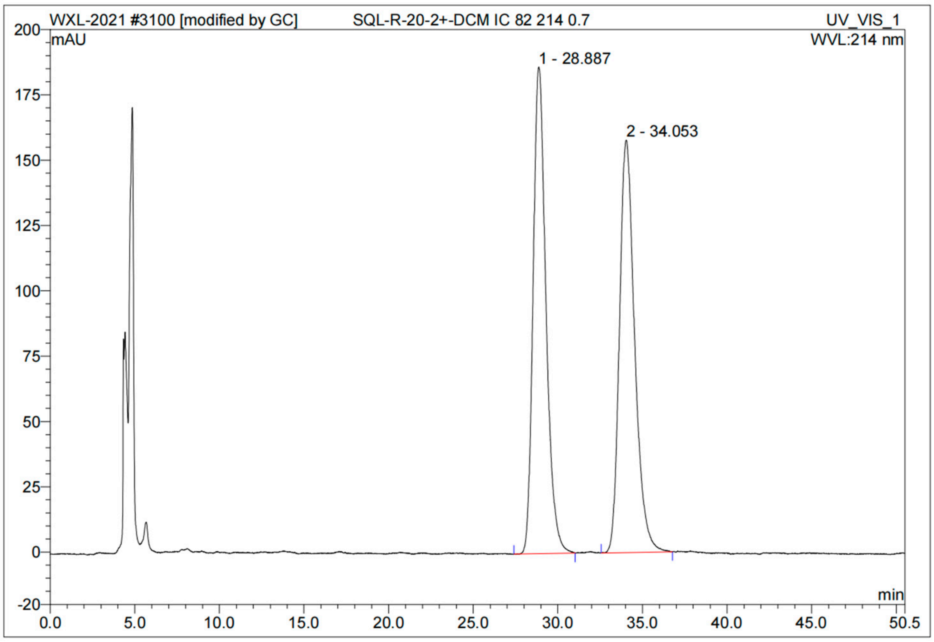

No.	Ret.Time min	Peak Name	Height mAU	Area mAU*min	Rel.Area %	Amount	Type
1	28.89	n.a.	186.293	164.003	49.96	n.a.	BMB*
2	34.05	n.a.	157.927	164.269	50.04	n.a.	BMB*
Total:			344.220	328.272	100.00	0.000	

b

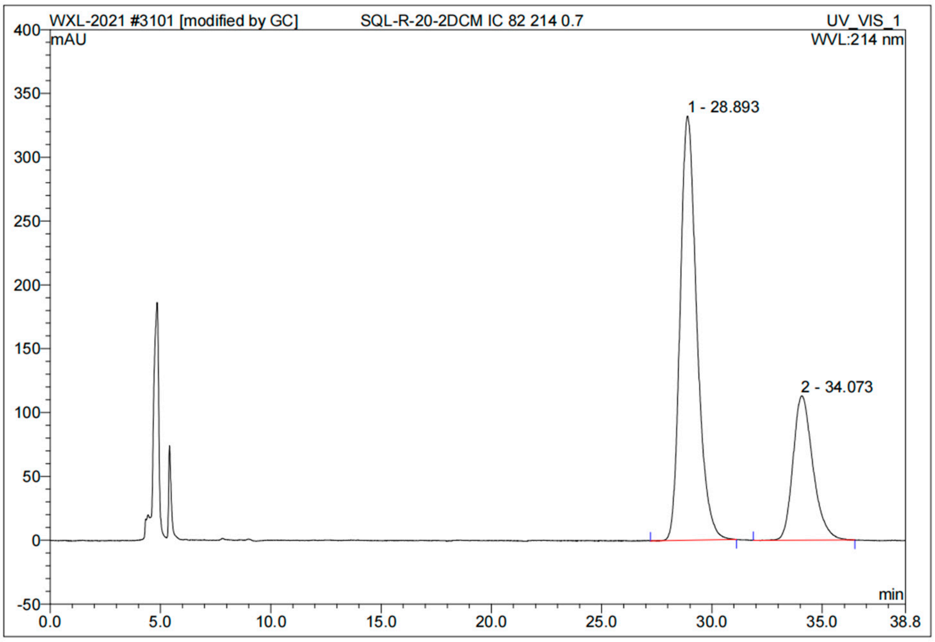

No.	Ret.Time min	Peak Name	Height mAU	Area mAU*min	Rel.Area %	Amount	Type
1	28.89	n.a.	332.585	291.835	71.08	n.a.	BMB*
2	34.07	n.a.	113.273	118.741	28.92	n.a.	BMB*
Total:			445.859	410.576	100.00	0.000	

Figure S122. The HPLC data of **1x** in Scheme 2.

a

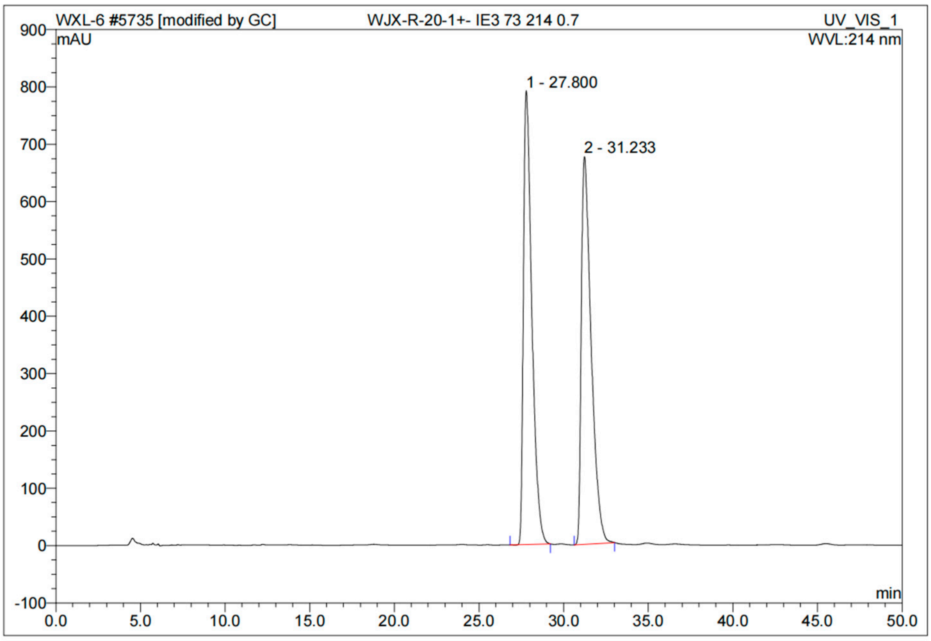

No.	Ret.Time min	Peak Name	Height mAU	Area mAU*min	Rel.Area %	Amount	Type
1	27.80	n.a.	791.678	455.772	49.98	n.a.	BMB*
2	31.23	n.a.	676.558	456.121	50.02	n.a.	BMB*
Total:			1468.237	911.893	100.00	0.000	

b

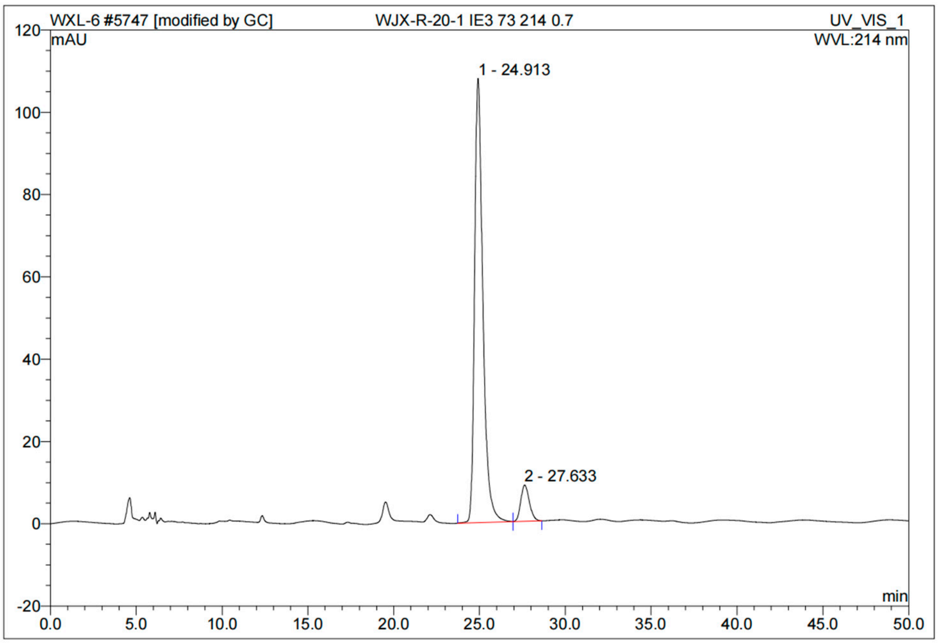

No.	Ret.Time min	Peak Name	Height mAU	Area mAU*min	Rel.Area %	Amount	Type
1	24.91	n.a.	107.967	60.603	92.17	n.a.	BM
2	27.63	n.a.	8.897	5.145	7.83	n.a.	MB
Total:			116.864	65.748	100.00	0.000	

Figure S123. The HPLC data of **2x** in Scheme 2.

7. The HPLC data of **3** in Scheme 3.

a

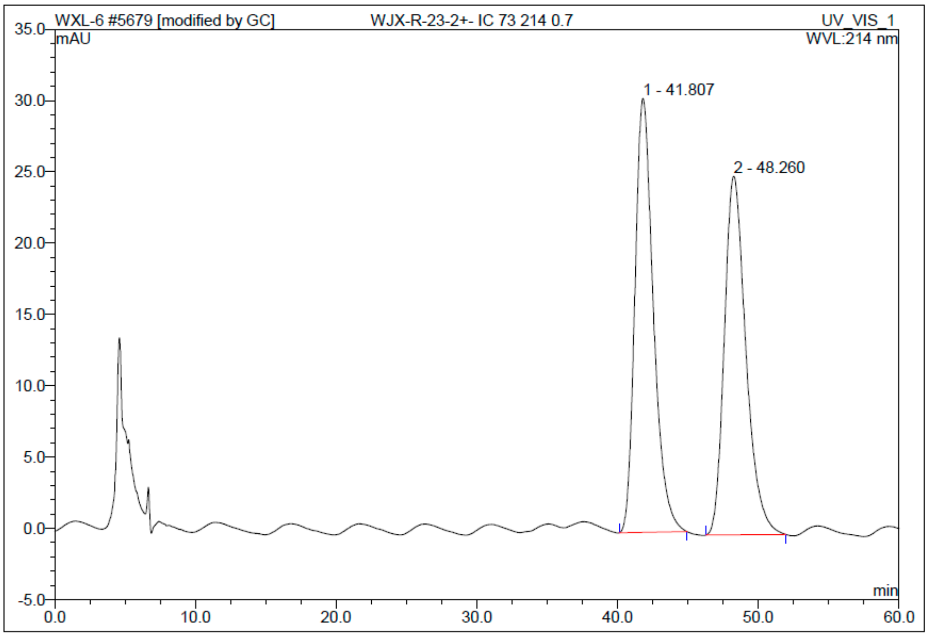

No.	Ret.Time min	Peak Name	Height mAU	Area mAU*min	Rel.Area %	Amount	Type
1	41.81	n.a.	30.449	46.326	50.88	n.a.	BMB
2	48.26	n.a.	25.135	44.720	49.12	n.a.	BMB
Total:			55.584	91.047	100.00	0.000	

b

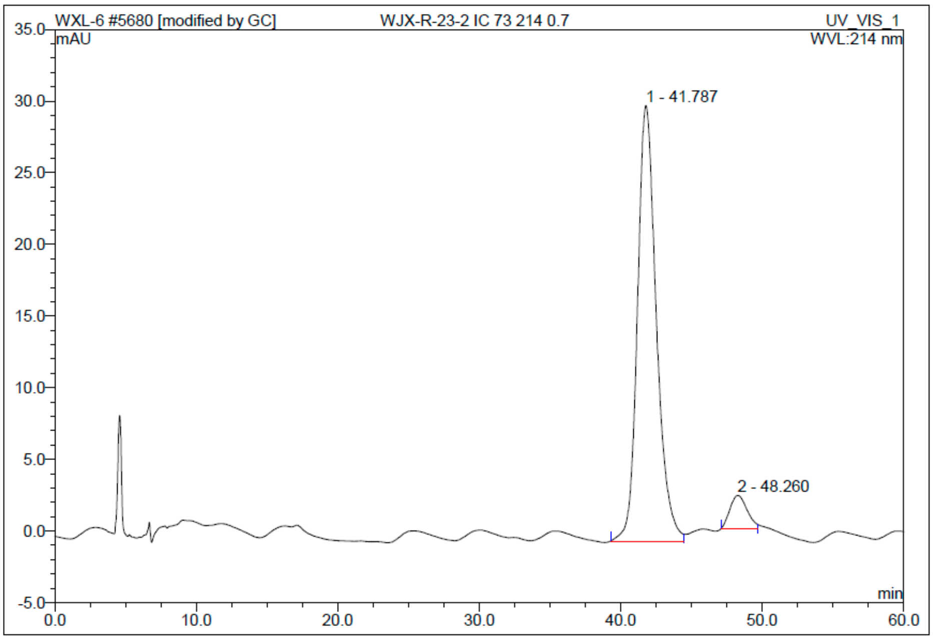

No.	Ret.Time min	Peak Name	Height mAU	Area mAU*min	Rel.Area %	Amount	Type
1	41.79	n.a.	30.401	47.035	93.57	n.a.	BM *
2	48.26	n.a.	2.315	3.234	6.43	n.a.	BM *
Total:			32.715	50.268	100.00	0.000	

Figure S124. The HPLC data of **3** in Scheme 3.

a

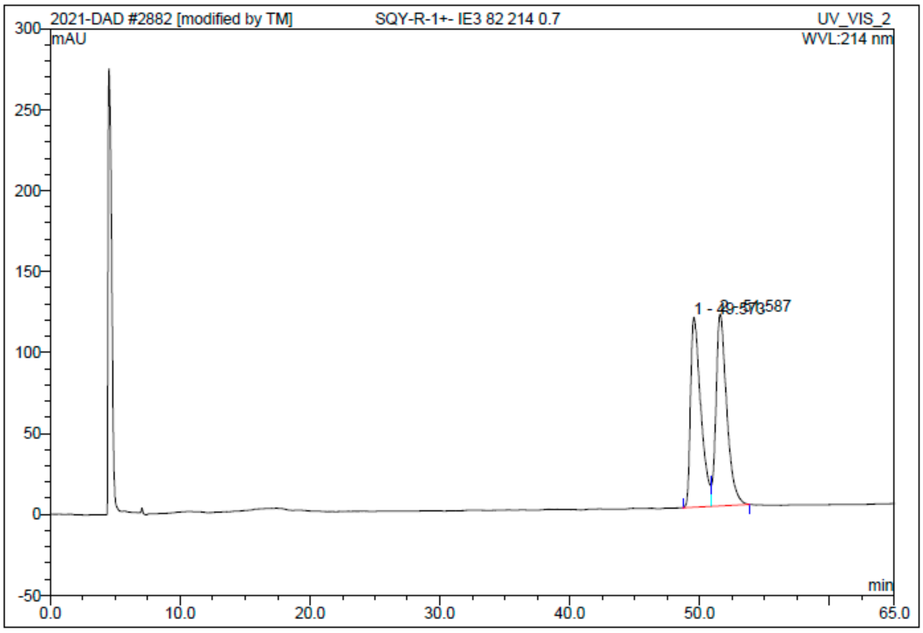

No.	Ret.Time min	Peak Name	Height mAU	Area mAU*min	Rel.Area %	Amount	Type
1	49.57	n.a.	117.267	107.068	48.45	n.a.	BM
2	51.59	n.a.	118.407	113.926	51.55	n.a.	MB
Total:			235.674	220.995	100.00	0.000	

b

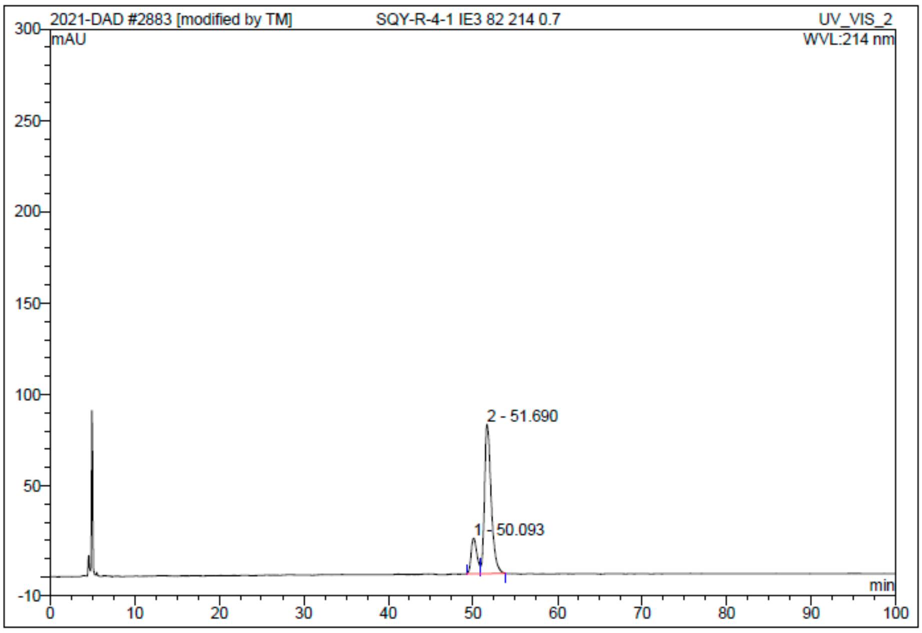

No.	Ret.Time min	Peak Name	Height mAU	Area mAU*min	Rel.Area %	Amount	Type
1	50.09	n.a.	19.370	15.584	17.26	n.a.	BM
2	51.69	n.a.	81.758	74.695	82.74	n.a.	MB
Total:			101.128	90.279	100.00	0.000	

Figure S125. The HPLC data of **1v** in Scheme 3.

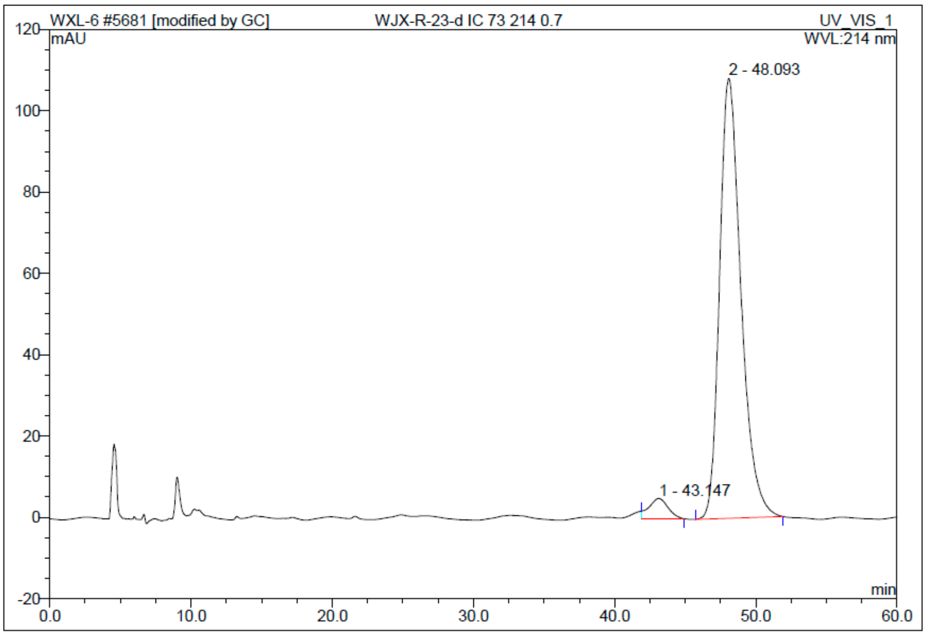

No.	Ret.Time min	Peak Name	Height mAU	Area mAU*min	Rel.Area %	Amount	Type
1	43.15	n.a.	4.952	7.824	3.95	n.a.	MB*
2	48.09	n.a.	108.196	190.098	96.05	n.a.	BMB
Total:			113.148	197.922	100.00	0.000	

Figure S126. The HPLC data of **4** in Scheme 3.
